# PEPbench—Open, Reproducible, and Systematic Benchmarking of Automated Pre‐Ejection Period Extraction Algorithms

**DOI:** 10.1111/psyp.70176

**Published:** 2025-11-11

**Authors:** Robert Richer, Julia Jorkowitz, Sebastian Stühler, Luca Abel, Miriam Kurz, Marie Oesten, Stefan G. Griesshammer, Nils C. Albrecht, Arne Küderle, Christoph Ostgathe, Alexander Kölpin, Tobias Steigleder, Nicolas Rohleder, Bjoern M. Eskofier

**Affiliations:** ^1^ Machine Learning and Data Analytics Lab (MaD Lab), Department Artificial Intelligence in Biomedical Engineering (AIBE) Friedrich‐Alexander‐Universität Erlangen‐Nürnberg (FAU) Erlangen Germany; ^2^ Chair of Health Psychology, Department of Psychology Friedrich‐Alexander‐Universität Erlangen‐Nürnberg (FAU) Erlangen Germany; ^3^ Department for Palliative Medicine University Hospital Erlangen Erlangen Germany; ^4^ Institute of High‐Frequency Technology Technische Universität Hamburg (TUHH) Hamburg Germany; ^5^ Translational Digital Health Group, Institute of AI for Health, Helmholtz Zentrum München – German Research Center for Environmental Health Neuherberg Germany

**Keywords:** algorithm benchmarking, B‐point, *dZ*/*dt*, ECG, ICG, open science, PEP, Q‐peak

## Abstract

The pre‐ejection period (PEP) is a widely used cardiac parameter in psychophysiology that reflects the duration between the onset of ventricular depolarization and the opening of the aortic valve. PEP is often used as a marker of cardiac sympathetic nervous system (SNS) activity, particularly in within‐subject comparisons under similar hemodynamic conditions. While many algorithms for automated PEP extraction from electrocardiography (ECG) and impedance cardiography (ICG) signals (more precisely, its first derivative, *dZ*/*dt*) have been proposed in literature, they have not been systematically benchmarked. This lack of standardized algorithm comparisons originates from the absence of open‐source algorithms and annotated datasets for evaluating PEP extraction algorithms. To address this issue, we introduce *PEPbench*, an open‐source Python package with different Q‐peak and B‐point detection algorithms from literature that can be combined to create comprehensive PEP extraction pipelines, and a standardized framework for evaluating PEP extraction algorithms. We use *PEPbench* to systematically compare 108 different algorithm combinations. All combinations are evaluated on two datasets with manually annotated Q‐peaks and B‐points, which we make publicly available as the first datasets with reference PEP annotations. Our results show that the algorithms can differ vastly in their performance and that B‐point detection algorithms introduce a considerable amount of error. Thus, we suggest that automated PEP extraction algorithms should be used with caution on a beat‐to‐beat level as their error rates are relatively high. This highlights the need for open and reproducible benchmarking frameworks for PEP extraction algorithms to improve the quality of research findings in the field of psychophysiology. With *PEPbench*, we aim to take a first step toward this goal and encourage other researchers to engage in the evaluation of PEP extraction algorithms by contributing algorithms, data, and annotations. We hope to establish a community‐driven platform, fostering innovation and collaboration in the field of psychophysiology and beyond.

## Impact Statement

This study introduces *PEPbench*, the first open‐source framework for systematically benchmarking pre‐ejection period (PEP) extraction algorithms, addressing a critical gap in the field. By providing standardized datasets with annotated ECG and *dZ*/*dt* signals, and enabling the evaluation of different PEP extraction approaches, our *PEPbench* framework sets a foundation for reproducible, systematic comparisons in psychophysiological research. This work fosters transparency, innovation, and collaboration, advancing the accuracy and reliability of psychophysiological studies.

**Abbreviations**
AE: absolute error
ANS: autonomic nervous system
ARE: absolute relative error
BMI: body mass index
CRC: Collaborative Research Center
DWT: discrete wavelet transform
*dZ*/*dt*
: first derivative of the thoracic impedance signal (*Z*_0_) acquired through ICG
*E*
: error
ECG: electrocardiogram
f‐TSST: friendly Trier Social Stress Test
HR: heart rate
HRV: heart rate variability
ICG: impedance cardiography
LVET: left ventricular ejection time
MAD: median absolute deviation
MAE: mean absolute error
MARE: mean absolute relative error
MDD: major depressive disorder
ME: mean error
PEP: pre‐ejection period
PNS: parasympathetic nervous system
RMS: root‐mean‐square
SNS: sympathetic nervous system
TFM: Task Force Monitor
TSST: Trier Social Stress Test
TWA: T‐wave amplitude

## 1 Introduction

Since its first systematic description (Harris et al. 1967; Newlin and Levenson 1979), the pre‐ejection period (PEP) has become a widely used hemodynamic parameter in psychophysiology, cardiovascular research, and clinical diagnostics (Albinet et al. 2024; Forouzanfar et al. 2019; Schächinger et al. 2001; Sherwood et al. 1986). It is a systolic time interval that reflects myocardial contractility and is defined as the time interval between the onset of ventricular depolarization and the opening of the aortic valve (Newlin and Levenson 1979). Because PEP is influenced by beta‐adrenergic stimulation and generally not affected by parasympathetic activity, it is often used to infer changes in sympathetic nervous system (SNS) activation, with shorter PEP values commonly interpreted as reflecting increased cardiac sympathetic drive (Drost et al. 2022; Sherwood et al. 1990). However, this interpretation is only valid under stable physiological conditions, as PEP is also sensitive to changes in cardiac preload and afterload. For example, during postural shifts such as head‐up tilt, PEP may increase despite higher sympathetic activity due to vascular influences (Cacioppo et al. 1994; Sherwood et al. 1990). Therefore, PEP should only be used to infer SNS changes when comparing measurements taken under similar conditions. Despite these limitations, PEP is still a valuable measure, particularly when compared to heart rate variability (HRV), which reflects input from both branches of the autonomic nervous system (Berntson et al. 1991; Obrist et al. 1974) and which primarily indexes cardiac parasympathetic (vagal) activity (Berntson et al. 1997; Laborde et al. 2017; Quigley et al. 2024).

For this reason, PEP is frequently used in studies of acute stress (Pilz et al. 2023; Weissman 2021), reward processing (Brenner and Beauchaine 2011; Chen et al. 2024), and pharmacological interventions (Clark et al. 2015), as well as in clinical populations such as individuals with Type D personality (Kupper et al. 2013) or major depressive disorder (Bair et al. 2021), and has been explored as an indicator of fetal health (Evers 1980; Organ et al. 1980).

PEP can be measured non‐invasively by simultaneously recording the heart's electrical activity via electrocardiography (ECG) and thoracic impedance via impedance cardiography (ICG) (Berntson et al. 2004). The time interval between the Q‐wave onset from the ECG as a start point (reflecting the onset of ventricular depolarization; Berntson et al. 2004) and the B‐point from the first derivative (*dZ*/*dt*) of the thoracic impedance signal (*Z*_0_) from the ICG as an endpoint (reflecting the opening of the aortic valve; Lozano et al. 2007) is determined. While this procedure may sound straightforward, it presents several challenges. Even though the start and endpoints of the PEP are well‐defined, different methods exist to determine these points (Forouzanfar et al. 2018). The Q‐wave *onset* is frequently used as the start point, but other methods like the Q‐wave *peak* (i.e., R‐wave onset) have been proposed since the Q‐wave onset can be difficult to detect, especially in noisy signals (Berntson et al. 2004). Similarly, the B‐point is often a subtle inflection signal before the upstroke in the *dZ*/*dt* signal, which can be challenging to discern (Lozano et al. 2007). Factors like poor electrode contact, patient movement, respiration, and individual anatomical differences can alter the *dZ*/*dt* waveform, complicating B‐point detection (Lozano et al. 2007). Furthermore, the morphology of the *dZ*/*dt* waveform can vary significantly between individuals, as well as under different physiological conditions, affecting the reliability of automated algorithms for detecting the B‐point (Lozano et al. 2007; Sherwood et al. 1990).

Researchers often rely on manual B‐point annotations by trained experts to address these issues. However, this approach is time‐consuming, resource‐intensive, and subject to inter‐rater variability, making it impractical for large‐scale studies or real‐time applications (Riese et al. 2003). Automated algorithms utilizing signal processing techniques have been developed to facilitate fiducial point detection in the ECG and *dZ*/*dt* signals and to streamline the measurement process. However, some of these algorithms use ensemble averaging approaches to reduce noise and artifacts (Cieslak et al. 2018; Kelsey and Guethlein 1990; Riese et al. 2003). While Cieslak et al. (2018) made commendable efforts to support the research community by openly sharing and documenting their code, their ensemble averaging approach is not suitable for beat‐to‐beat analysis (Forouzanfar et al. 2018).

Over the past decades, researchers have proposed various beat‐to‐beat B‐point extraction algorithms. Most approaches determine the B‐point based on certain conditions (e.g., finding extrema or zero‐crossings) in the *dZ*/*dt* signal or one of its derivatives (Árbol et al. 2017; Bagal et al. 2017; Carvalho et al. 2011; Debski et al. 1993; DeMarzo and Lang 1996; Drost et al. 2022; Forouzanfar et al. 2018; Naidu et al. 2014; Ono et al. 2004; Pale et al. 2021; Sherwood et al. 1990; Stern et al. 1985), while others transform the *dZ*/*dt* signal through weighted window functions that emphasize specific portions of the waveform before the B‐point search (Miljković and Šekara 2022), segment the *dZ*/*dt* signal through curve fitting before applying matched filters (Nagel et al. 1989), or use adaptive search windows based on the ECG R‐peaks and S‐waves, respectively (Karpiel et al. 2022). Furthermore, different time‐frequency (Wang et al. 1995) or wavelet‐based B‐point detection approaches were proposed (Hu et al. 2014; Nagel et al. 1989; Naidu et al. 2014; Shuguang et al. 2005). In contrast to these predominantly signal‐processing‐based approaches, other researchers proposed data‐driven methods that determine the B‐point location based on linear/quadratic regression (e.g., Lozano et al. 2007) or more complex machine learning models (Sheikh et al. 2022). However, these are often evaluated on private datasets without comparisons to other algorithms, restricting their broader applicability.

In addition, a variety of different, mostly wavelet‐ or machine learning‐based, algorithms for ECG delineation exist (Banerjee 2019; Chen and Maharatna 2020; Li et al. 1995; Sabherwal et al. 2019; Saini et al. 2014; Shivappriya et al. 2006). However, most of them don't specifically detect the Q‐wave onset, but rather segment the entire QRS complex, the P, or T waves, respectively. Other algorithms were proposed to directly locate or estimate the location of the Q‐wave onset or the Q‐peak (e.g., Berntson et al. 2004; Martinez et al. 2004; Van Lien et al. 2013).

Taken together, this yields an overwhelming number of different algorithm combinations for PEP estimation, requiring a systematic evaluation to determine the best‐performing approach. Attempts to compare different B‐point extraction algorithms were made in the past (Árbol et al. 2017; Forouzanfar et al. 2018), but they did not provide open‐source code or annotated datasets, leaving the field without a standardized and reproducible evaluation framework.

Moreover, a straightforward way for researchers to access and apply these algorithms is missing, further limiting their use in empirical studies. Commercial systems like Biopac and MindWare already offer integrated PEP extraction algorithms used in research and clinical practice (e.g., Bagley and El‐Sheikh 2014; Bair et al. 2021; Chen et al. 2024; Clark et al. 2015; Murch et al. 2020). While these tools are widely used, their proprietary nature often limits transparency, hindering independent validation and generalizability across populations and recording conditions. Notably, MindWare provides several options for B‐point detection along with documentation linking these options to published algorithms (Debski et al. 1993; Lozano et al. 2007; Sherwood et al. 1990). However, the full implementation details and access to the underlying source code are still restricted.

Publicly available and annotated datasets are essential for evaluating PEP extraction algorithms as they serve as the basis for standardized evaluation protocols that can enable accurate performance assessment, addressing the lack of transparency and hindering reproducibility in PEP research. To the best of our knowledge, only two datasets with annotated *dZ*/*dt* signals for B‐point detection are publicly available (Miljković and Šekara 2023; Pale et al. 2021), but none with synchronized and annotated ECG and *dZ*/*dt* signals for PEP estimation. Similar to ECG processing advancements driven by open datasets like the MIT‐BIH Arrhythmia Database (Moody and Mark 2001) and initiatives like PhysioNet (Goldberger et al. 2000), standardized PEP benchmarking frameworks can accelerate progress in psychophysiology, enabling the establishment of challenges like the PhysioNet Challenges which demonstrate the value of community‐driven platforms (e.g., Moody 2002, 2008; Moody et al. 2006).

Building on our prior initiatives for gait analysis benchmarking (Küderle et al. 2024), this paper introduces *PEPbench*, an open framework for evaluating PEP extraction algorithms that can serve as a starting point for further endeavors to advance the field of psychophysiology.

## 2 Methods

**FIGURE 1:**
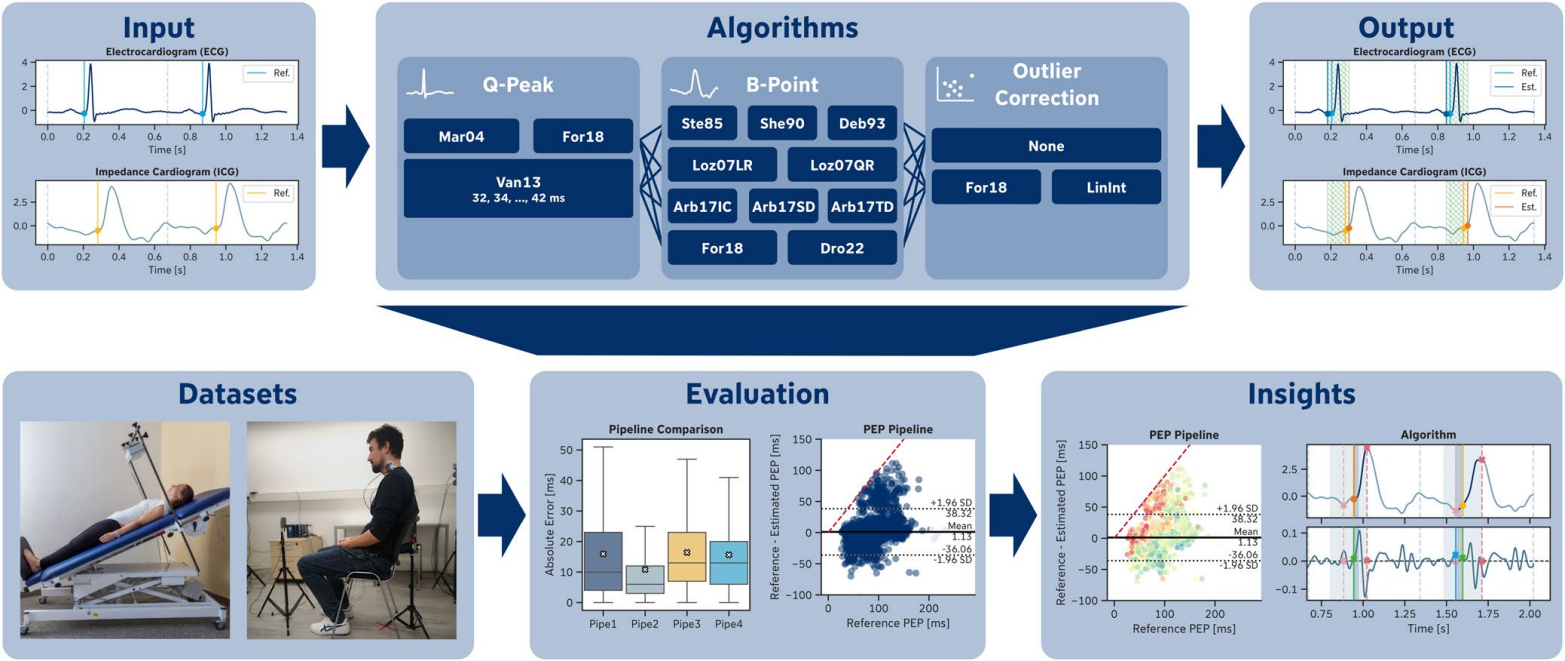
Overview of the key contributions. This paper presents the *PEPbench* framework, which provides a set of publicly available algorithms for Q‐peak and B‐point detection from electrocardiogram (ECG) and the first derivative of the impedance cardiogram signal (*dZ*/*dt*) input signals that can be combined to build pre‐ejection period (PEP) extraction pipelines for automated PEP extraction (top). Furthermore, *PEPbench* offers a standardized evaluation framework for algorithm comparison, along with various functions for data analysis and visualization. Using this framework, we systematically compare different algorithms on two newly released, publicly available datasets containing annotated ECG and *dZ*/*dt* signals with expert‐annotated Q‐peaks and B‐points (bottom).

For the purpose of systematically comparing different PEP extraction pipelines, we used two datasets from two separate studies, which are described in detail in Section 2.2. We manually annotated the relevant fiducial points of the PEP in the ECG and *dZ*/*dt* signals of both datasets, which were used as ground truth for evaluating the automated PEP extraction pipelines. After preprocessing the data, we applied various PEP extraction pipelines, each consisting of three steps: a *Q‐peak* extraction algorithm applied to the ECG signal, a *B‐point* extraction algorithm applied to the *dZ*/*dt* signal, and an optional *outlier correction* algorithm applied to the extracted B‐point values, as suggested by previous research (Forouzanfar et al. 2018, 2019). Finally, the different PEP extraction pipelines were evaluated using a standardized evaluation framework. All these components are integrated into our Python package, *PEPbench*, which is available on GitHub (https://github.com/empkins/pepbench), while the two datasets are available on the Open Science Framework (OSF) platform (EmpkinS Dataset: https://doi.org/10.17605/OSF.IO/SH3XN, Guardian Dataset: https://doi.org/10.17605/OSF.IO/GYH75). The entire process is summarized in Figure 1.

### 2.1 Overview of the *PEPbench* Framework

The *PEPbench* framework is a Python package designed for evaluating PEP extraction pipelines. It is modular and extensible, enabling the easy integration of new PEP extraction and outlier correction algorithms. The package is built on top of the domain‐agnostic *tpcp* package (Küderle et al. 2023), which provides framework‐independent tools for algorithm development and evaluation. The *PEPbench* framework comprises several main components:

- **Datasets**: This module provides data loader classes for the datasets used in our evaluations, which are reusable code structures (known as *classes* in Python) that define how datasets are accessed. Each data loader class includes methods (also known as *functions*) for loading raw data, manual annotations, and metadata. New datasets can be integrated by implementing a new data loader class using the *tpcp* package.
- **Algorithms**: This module includes three different algorithms for Q‐peak extraction from the ECG signal, twelve algorithms for B‐point extraction from the *dZ*/*dt* signal, and two algorithms for correcting outliers in the extracted B‐points. Additionally, helper algorithms are provided for cardiac cycle segmentation and C‐point detection. Each algorithm is implemented as a separate class and inherits from a common base class, which defines a common interface and ensures that all algorithms follow the same structure and usage pattern. This consistent design allows each algorithm to be accessed, configured, and used in the same standardized way. The algorithms can be run independently or as part of a PEP extraction pipeline. New algorithms can also be integrated by creating a new algorithm class using the *tpcp* package.
- **Pipelines**: This module provides a framework for building PEP extraction pipelines. In this context, a *pipeline* refers to a structured sequence of processing steps applied to physiological signal data. Pipelines are composed of a Q‐peak extraction algorithm, a B‐point extraction algorithm, and an optional outlier correction algorithm. Users can select from the implemented algorithms or use custom algorithms. The pipeline class includes methods for applying pipelines to data and storing both the final results and intermediate processing steps.
- **Evaluation**: This component offers a standardized evaluation framework for running and assessing PEP extraction pipelines on specific datasets. Each unique combination of a PEP extraction pipeline and dataset is referred to as a “challenge.” From each challenge, a set of evaluation metrics on different aggregation levels is computed and stored in a structured format for further analysis and comparison.
- **Plotting**: This module includes a set of functions for visualizing data and annotations, outputs from individual algorithms, and evaluation results.

### 2.2 Dataset Descriptions

To systematically compare different PEP extraction pipelines, we used two datasets from two different studies involving healthy participants. The first dataset was collected in the context of the Collaborative Research Center (CRC) *Empatho‐Kinesthetic Sensor Technology* (*EmpkinS*) (SFB 1483) at Friedrich‐Alexander‐Universität Erlangen‐Nürnberg (FAU) to explore the use of contactless radar technology for the assessment of acute psychosocial stress. The second dataset was collected in the context of the *Guarded by Advanced Radar Technology‐Based Diagnostics Applied in Palliative and Intensive Care Nursing* (*Guardian*) project at the University Hospital Erlangen to explore the use of contactless radar technology for monitoring patients in palliative care.

In addition to these two datasets described in more detail in the following, we also implemented two other datasets into the PEPbench framework: the *ReBeatICG* dataset (Pale et al. 2021) and the *TimeWindowICG* dataset (Miljković and Šekara 2023). Both datasets provide synchronized ECG and *dZ*/*dt* recordings of participants undergoing different psychophysiological manipulations. While B‐points were manually annotated in both datasets, no Q‐peaks were annotated in the corresponding ECG data. Furthermore, the annotations were only performed by a single annotator. Due to these limitations in annotation completeness, and since discussing the results of these additional datasets in detail would go beyond the scope of this manuscript, we chose not to include them in the core analyses. However, we provide their integration within the PEPbench framework, together with the analysis scripts, to support future benchmarking extensions and cross‐study comparisons.

#### 2.2.1 EmpkinS Dataset

In this dataset, we recorded *n* = 15 healthy participants (60% female) with a mean age of 23.1 ± 2.6 years and a mean body mass index (BMI) of 21.9 ± 2.4 kg/m^2^ (M ± SD) between December 2022 and May 2023. Participants were screened for eligibility via an online questionnaire and were excluded if they reported any of the following, as outlined in previous studies (Richer et al. 2024): (1) an age below 18 years or above 50 years, (2) non‐German native language, (3) a BMI below 18 or above 30, (4) the current presence of a physical or mental illness, (5) medication intake (such as beta‐blockers, antidepressants, or glucocorticoids), (6) smoking or use of other drugs, or (7) previous experience with stress tests.

Because of associations between the menstrual cycle and stress reactivity, we only included female participants who were not using hormonal contraceptives and were in the early follicular phase of their menstrual cycle, according to self‐report (Kirschbaum et al. 1999). Eligible participants were invited to the EmpkinS Lab (FAU, Erlangen), where the study procedure was explained in detail, and written informed consent was obtained. The study was approved by the local ethics committee of FAU (protocol #493_20 B) and was conducted in accordance with the Declaration of Helsinki.

The tests were conducted between 1:00 p.m. and 7:00 p.m. to minimize the impact of circadian rhythms on stress reactivity (Smyth et al. 1997). Participants were instructed to abstain from consuming alcohol on the preceding day and the day of testing, to wake at least 3 h before the testing, and to refrain from eating and engaging in heavy physical activity at least 1 h prior to the study. Each participant underwent a standardized laboratory stress induction protocol and a stress‐free control condition in randomized order on two consecutive days. The stress induction consisted of a modified version of the Trier Social Stress Test (TSST; Kirschbaum et al. 1993). The TSST is a widely used standardized acute psychosocial stress induction protocol that includes a mock job interview (referred to as *Interview*) and a mental arithmetic task (referred to as *Mental Arithmetic*). Each phase lasts 5 min and is conducted in front of a two‐person evaluation panel providing social‐evaluative feedback. Before the task, participants were given 5 min to prepare for the upcoming job interview. For our experiment, we expected the TSST to modulate the PEP through increased SNS activity during acute psychosocial stress exposure, as shown in previous work (Seddon et al. 2020; Yim et al. 2015).

As a control condition, we used a modified version of the friendly TSST (f‐TSST; Wiemers et al. 2013), designed to be similar to the TSST but without inducing acute psychosocial stress. Analogous to the TSST, the f‐TSST consists of a 5‐min *Interview* and a 5‐min *Mental Arithmetic* phase; however, the evaluation panel provides only friendly and positive feedback.

In this study, we modified both the TSST and f‐TSST protocols to make them more suitable for radar‐based stress assessment by introducing speech pauses of 1 min before the *Interview* and after the *Mental Arithmetic* phases of the (f‐)TSST (referred to as *Pause 1* and *Pause 5*, respectively). Additionally, we added 30‐s speech pauses after 2.5 min of the *Interview* and *Mental Arithmetic* phases (*Pause 2* and *Pause 4*) and between the two phases (*Pause 3*) (see Figure S1a). These pauses were intended to improve the signal quality of the radar recordings by reducing artifacts caused by speech and body movements (Herzer et al. 2022).

Throughout the entire (f‐)TSST, we acquired synchronized ECG and ICG recordings using a *Biopac MP 160* system (Biopac, Goleta, CA, USA) equipped with *ECG100C* and *NICO100C* amplifiers, respectively. The electrodes were attached before participants filled out state questionnaires before the (f‐)TSST, which occurred at least 10 min before the (f‐)TSST start to allow for the stabilization of the electrode‐skin interface, as recommended by Sherwood et al. (1990). The ECG was acquired using a standard three‐lead, one‐channel configuration with adhesive disposable Ag/AgCl electrodes placed on the chest according to a modified Lead II of Einthoven's triangle. For the ICG, a configuration with four electrode pairs was used: two pairs were placed laterally on the neck and two pairs were placed laterally on the torso at the level of the xiphoid process. The electrodes within each pair were placed 3 cm apart. To measure the baseline thoracic impedance (*Z*_0_) and its first derivative (*dZ*/*dt*), we induced a current with an RMS amplitude of 400 μA and a frequency of 50 kHz through the current (outer) electrodes and measured the voltage across the voltage (inner) electrodes. Both signals were recorded with a sampling rate of 1000 Hz using the provided *AcqKnowledge* software and stored as .*acq* files.

#### 2.2.2 Guardian Dataset

This dataset is a subset of a larger dataset previously published (Schellenberger et al. 2020). The subset used in this work consists of *n* = 24 participants (50% female) with a mean age of 31.2 ± 11.0  years and a mean BMI of 23.8 ± 3.5 kg/m^2^ (M ± SD). Participants were invited to the Palliative Care Unit of the University Hospital Erlangen between February and July 2018, where the study procedure was explained in detail and written consent was obtained before testing commenced. The study was approved by the local ethics committee of FAU (protocol #85_15B) and conducted in accordance with relevant guidelines and regulations. Before testing, participants were screened for their health status by a physician, who measured heart rate, blood pressure, and heart sounds. Only participants with values within clinically acceptable ranges and without detected abnormalities were included in the study. Eligible participants were then asked to lie down on a tilt table in a supine position, and electrodes for ECG and ICG recordings were attached before they were asked to relax for 10 min to allow for the stabilization of the electrode‐skin interface, as recommended by Sherwood et al. (1990). Afterwards, the measurement protocol started, which consisted of five phases (Schellenberger et al. 2020; Figure S1b):

1. **Resting**: The 10‐min electrode stabilization period was followed by another 10‐min resting period in which participants were instructed to lie still and breathe regularly.
2. **Valsalva maneuver**: Participants performed the Valsalva maneuver, which involves generating intrathoracic pressure by forcefully attempting to exhale against a closed glottis for 20 s, thereby preventing actual airflow. Afterwards, participants breathed normally for 5 min. This procedure was repeated three times. The Valsalva maneuver is known to induce baroreflex‐mediated sympathetic activation and to transient changes in cardiac preload due to increased intrathoracic pressure, meaning that related changes in PEP reflect a combination of sympathetic and pre‐load‐related effects rather than purely SNS‐mediated influences (Gorlin et al. 1957; Sharpey‐Schafer 1955).
3. **Holding breath**: Participants first inhaled and held their breath as long as possible. After the first breath‐hold period, they exhaled, breathed normally for about 1 min, and then exhaled and held their breath again.
4. **Tilt table up**: Participants were tilted from a supine to a head‐up position of 70°, where they remained for 10 min. This procedure induces sympathetic activation due to the orthostatic challenge as well as a reduction in venous return and cardiac preload (Kenny et al. 1986; Van Zanten et al. 2024).
5. **Tilt table level**: Finally, participants were tilted back to the supine position (0°) and remained in this position for another 10 min.

Throughout the study, synchronized ECG and ICG recordings were obtained using a *Task Force Monitor (TFM) 3040i* system (CNSystems Medizintechnik GmbH, Graz, Austria). The ECG was recorded using a four‐lead configuration with adhesive disposable Ag/AgCl electrodes placed on the chest to record Lead I and Lead II according to Einthoven's triangle, from which the Lead II configuration was used for further processing and analyses. The ICG was recorded using a four‐electrode configuration: one partial band electrode was placed at the nape of the neck close to the glottis, two partial band electrodes were placed laterally on the thorax close to the xiphoid process, and one neutral spot electrode was placed on the left leg, following manufacturer recommendations (Fortin et al. 2006; Schellenberger et al. 2020). From these measurements, the baseline thoracic impedance (*Z*_0_) and its first derivative (*dZ*/*dt*) were computed.

The ECG signal was recorded at a sampling rate of 1000 Hz, while the *dZ*/*dt* signal was recorded at 500 Hz using the *Task Force Monitor* software and stored as .mat files. The ECG signal was downsampled to 500 Hz to match the sampling rate of the *dZ*/*dt* signal.

### 2.3 Preprocessing

After recording the data, we preprocessed the acquired signals to remove artifacts such as baseline drifts and power‐line interference. The ECG signal was filtered using a fifth‐order Butterworth band‐pass filter with cutoff frequencies of 0.67 and 45 Hz (Makowski et al. 2021). The *dZ*/*dt* signal was filtered using a fourth‐order Butterworth band‐pass filter with cutoff frequencies of 0.5 and 25 Hz, as proposed by Forouzanfar et al. (2019).

Following filtering, we segmented the time‐series data into individual cardiac cycles to facilitate the subsequent manual annotation of fiducial points. First, we detected the R‐peaks in the ECG signal using the *NeuroKit2* Python package (Makowski et al. 2021). The R‐peaks were then used to segment the ECG and *dZ*/*dt* signals into individual cardiac cycles based on the preceding RR interval. The start of the current cardiac cycle was defined as the R‐peak of the current cardiac cycle minus 35% of the preceding RR interval, and the end was defined as the R‐peak of the current cardiac cycle plus 65% of the preceding RR interval. This segmentation process divided the ECG and *dZ*/*dt* signals into individual cardiac cycles, which were subsequently manually annotated by trained experts.

### 2.4 Manual PEP Annotation

For data annotation, we used the *MaD GUI*, an open‐source Python package for annotating time‐series sensor data (Ollenschläger et al. 2022). The *MaD GUI* provides a graphical user interface for visualizing and annotating time‐series data and can be flexibly adapted to various data formats and annotation tasks by implementing data loader and annotation handler classes. Within the *MaD GUI*, the pre‐processed ECG and *dZ*/*dt* signals were plotted together in a joint view (Figure S2). Additionally, pre‐computed cardiac cycle borders for the ECG and *dZ*/*dt* signals, as described in the previous section, were overlaid to aid the annotation process.

Given the extensive number of cardiac cycles in both datasets, subsets from each phase were randomly selected for manual annotation. For the *EmpkinS Dataset*, random subsets of 30 s were selected for the *Preparation*, *Interview*, and *Mental Arithmetic* phases, while 10‐s subsets were selected for the *Pause 1* and *Pause 5* phases of the (f‐)TSST. For the *Guardian Dataset*, random subsets of 60 s were selected for all phases (*Resting*, *Valsalva*, *Holding Breath*, *Tilt Table Up*, and *Tilt Table Level*). In total, 5116 cardiac cycles (average 341 ± 72 cycles per participant) were annotated for the *EmpkinS Dataset*, and 6795 cardiac cycles (average 283 ± 62 cycles per participant) were annotated for the *Guardian Dataset* (Table 1a and 1b).

**TABLE 1a:** Total number of manually annotated cardiac cycles (CCs) in the *EmpkinS Dataset*, split by condition and phase.

EmpkinS Dataset		
	# of CCs	
Phase/Condition	f‐TSST	TSST
Preparation	625	695
Pause 1	224	233
Interview	686	793
Mental Arithmetic	690	767
Pause 5	192	211
Total	2417	2699

**TABLE 1b:** Total number of manually annotated cardiac cycles (CCs) in the *Guardian Dataset*, split by phase.

Guardian Dataset	
Phase	# of CCs
Resting	1308
Valsalva	1347
Holding Breath	1436
Tilt Up	1486
Tilt Level	1218
Total	6795

In these selected subsets, two independent, trained annotators manually marked the fiducial points of the PEP in both signals. The annotation guidelines were as follows:

1. **ECG signal**: We annotated the Q‐wave *peak* as the start point of the PEP instead of the Q‐wave *onset* based on the findings by Berntson et al. (2004) who reported that the Q‐wave *peak* offers greater reliability in annotation compared to the Q‐wave *onset*, which can be difficult to detect, particularly in noisy signals or Lead II electrode configurations. Thus, we consistently annotated the Q‐wave *peak* (further referred to as Q‐*peak*) as the start point because using the Q‐wave onset whenever present and the Q‐wave peak otherwise can induce large intra‐ and inter‐subject variations in the annotation process, which should be avoided (Berntson et al. 2004).
2. ***dZ*** **/** ***dt*** **signal**: We annotated the B‐points according to the recommendations of Nagel et al. (1989) and Sherwood et al. (1990) which were summarized into a decision tree for visual B‐point detection by Árbol et al. (2017) In short, this decision tree guides the annotator through the *dZ*/*dt* signal and indicates where to localize the onset of the rapid up‐slope of the B‐point, depending on the shape of the wave, taking into consideration that it may be preceded by an incisive notch, a plateau, an inflection point, a sharp change in the gradient of the graph, or even the lack of any identifiable mark (Árbol et al. 2017). Furthermore, the examples of challenging *dZ*/*dt* signal patterns presented by Forouzanfar et al. (2018) were considered in the annotation process. As additional visual guidance, we used the simultaneously plotted ECG signal and used the Q‐peak as the lower limit for the B‐point annotation to ensure that the annotated B‐points were always occurring *after* the corresponding annotated Q‐peak in the ECG signal.

If one of the annotators could not identify the fiducial points in a cardiac cycle, the cycle was marked as *Artifact* and was excluded from further analysis. Finally, all manual annotations were exported as .csv files, together with the automatically extracted cardiac cycle borders, and were used as ground truth for the evaluation of the automated PEP extraction algorithms.

### 2.5 PEP Extraction Pipelines

#### 2.5.1 Overview of Algorithms

In this work, we selected algorithms for PEP extraction from ECG and *dZ*/*dt* signals that were previously proposed in the literature. We primarily focused on algorithms that follow a signal processing‐based approach instead of a data‐driven approach (e.g., utilizing machine learning) to ensure a fair comparison of the algorithms and an objective evaluation of their performance across different datasets.

In total, we implemented three different Q‐peak extraction algorithms and twelve different B‐point extraction algorithms, which are summarized in Tables 2 and 3, respectively. Additionally, we implemented two outlier correction algorithms for correcting B‐point outliers and one *dummy* outlier correction algorithm that passes through the input data unchanged, corresponding to a “no correction” scenario. The outlier correction algorithms are summarized in Table 4. The outliers were identified according to Forouzanfar et al. (2018), who defined B‐point outliers as values that were more than three times the median absolute deviation away from the median of stationarized B‐point values. The stationarization was performed by subtracting the low‐pass filtered B‐point values from the original B‐point values, where the low‐pass filter was a fourth‐order Butterworth filter with a cut‐off frequency of 0.1 Hz.

**TABLE 2:** Overview of Q‐peak detection algorithms implemented in pepbench which are evaluated in this work.

Original publication	Abbr.	Algorithm Name in *PEPbench* (QPeakExtraction{*name*})	Description
Martinez et al. (2004)	Mar04	Martinez2004Neurokit	Discrete Wavelet Transform (DWT)‐based algorithm for ECG waveform delineation, implemented in *neurokit2* Python library (Makowski et al. 2021).
Van Lien et al. (2013)	Van13 (t ms)	VanLien2013	Fixed time interval t (original publication: 40 ms) subtracted from the R‐peak; *This publication*: different time intervals $t\in \left\{32,34,36,38,40,42\right\}\;\mathrm{ms}$.
Forouzanfar et al. (2018)	For18	Forouzanfar2018	Last sample before the R‐peak that is below a certain threshold ($-1.2\cdot R/f_{s}$), where R is the amplitude of the R‐peak and *f*_s_ is the sampling frequency of the ECG signal; *This publication*: a scaling factor is used instead of *f*_s_ due to different sampling rates of datasets compared to the original publication (2000 Hz).

**TABLE 3:** Overview of B‐point detection algorithms implemented in pepbench which are evaluated in this work.

Original publication	Abbr.	Algorithm name in *PEPbench* (BPointExtraction{*name*})	Description
Stern et al. (1985)	Ste85	Stern1985	Last local minimum of dZ/dt signal before C‐point.
Sherwood et al. (1990)	She90	Sherwood1990	Last zero‐crossing of dZ/dt signal before C‐point.
Debski et al. (1993)^a^	Deb93	Debski1993SecondDerivative	Last local minimum of $d^{2}Z/dt^{2}$ signal before C‐point.
Lozano et al. (2007)	Loz07LR	Lozano2007LinearRegression	Linear regression model based on the interval between ECG R‐peak and *dZ*/*dt* C‐point: B=0.55·RC+4.45B, where B is the location of the B‐point and RC is the time interval between R‐peak and C‐point.
	Loz07QR	Lozano2007QuadraticRegression	Quadratic regression model based on the interval between ECG R‐peak and *dZ*/*dt* C‐point: $B=-0.0032\cdot RC^{2}+1.233\cdot \mathrm{RC}-31.59$, where B is the location of the B‐point and RC is the time interval between R‐peak and C‐point.
Árbol et al. (2017)	Arb17IC	Arbol2017IsoelectricChanges	Last crossing of dZ/dt signal through the mean of the dZ/dt signal in cardiac cycle [referred to by Árbol et al. (2017) as “isoelectric crossings”] before C‐point.
	Arb17SD	Arbol2017SecondDerivative	Maximum of $d^{2}Z/dt^{2}$ signal in a 50 ms window starting 150 ms before the C‐point.
	Arb17TD	Arbol2017ThirdDerivative	Maximum of $d^{3}Z/dt^{3}$ signal within 300 ms before the C‐point.^b^
Forouzanfar et al. (2018)	For18	Forouzanfar2018	Last zero‐crossing or last local maximum of $d^{3}Z/dt^{3}$ signal in the first third of the most significant monotonically increasing segment between A‐point (local minimum in window before C‐point with window size of one‐third of the preceding RR‐interval) and C‐point that fulfills certain conditions.
Pale et al. (2021)	Pal21	Pale2021	Search window based on C‐point location and C‐point amplitude in the *dZ*/*dt* signal. B‐point is identified as either (1) the local minimum closest to the C‐point or (2) the first point at which the slope of the *dZ*/*dt* signal exceeds a defined threshold. If no condition is fulfilled, the search is repeated with a relaxed slope threshold. If still unsuccessful, the global minimum of the *dZ*/*dt* signal within the cardiac cycle before the C‐point is returned.
Drost et al. (2022)	Dro22	Drost2022	Point with maximum y‐distance between dZ/dt signal and a straight line connecting the C‐point and the point in the dZ/dt signal 150 ms before the C‐point.
Miljković and Šekara (2022)	Mil22	Miljkovic2022	B‐point is identified by applying a weighted time window to the *dZ*/*dt* signal segment preceding the C‐point. The transformation should enhance the characteristic shape of the B‐point, facilitating its detection based on the amplified features in the resulting signal.

^a^ In Debski et al. (1993), a second B‐point detection algorithm was proposed that uses the last maximum of the $d^{3}Z/dt^{3}$ signal before the C‐point. In *PEPbench*, we only implemented the second derivative‐based algorithm since a third‐derivative‐based algorithm was also proposed later by Árbol et al. (2017) with the only difference being the window size for searching the local maximum (see above).

^b^ In DeMarzo and Lang (1996), an algorithm was proposed that detects the B‐point as the point “where the slope towards the right exceeds the slope towards the left by the greatest value.” This is almost equivalent to finding the maximum change rate of the curvature, which corresponds to finding the maximum of the $d^{3}Z/dt^{3}$ signal before the C‐point as proposed later by Árbol et al. (2017). In *PEPbench*, we only implemented the version by Árbol et al. (2017).

**TABLE 4:** Overview of B‐point outlier correction algorithms implemented in pepbench which are evaluated in this work. B‐point outliers were defined according to the procedure described by Forouzanfar et al. (2018).

Original publication	Abbr.	Algorithm name in *PEPbench* (QPeakExtraction{*name*})	Description
n/a	None	Dummy	Dummy outlier correction algorithm that passes through the input data unchanged.
This publication	LinInt	LinearInterpolation	Linear interpolation to correct B‐point outliers, defined as more than three times the Median Absolute Deviation (MAD) away from the median of the stationarized B‐points.
Forouzanfar et al. (2018)	For18	Forouzanfar2018	Forward and backward autoregressive models to correct B‐point outlier, defined as more than three times the Median Absolute Deviation (MAD) away from the median of the stationarized B‐points.

We used all algorithms with their default parameters, as proposed in the original publications, with some exceptions. For the Q‐peak and B‐point detection algorithms proposed by Forouzanfar et al. (2018), we did not use the sampling frequency of the recorded *dZ*/*dt* signals as scaling factors for computing signal amplitude‐based thresholds, since the sampling frequency is unrelated to the signal amplitude and different sampling rates would lead to different thresholds for the same signal while the signal amplitude remains constant. Thus, we considered it inappropriate to scale the signal amplitudes by the sampling frequency of the signal. Instead, we introduced a tunable *scaling factor* parameter that can be set externally and is independent of the sampling frequency of the signal. By default, this scaling factor is set to 2000, which corresponds to the sampling frequency of the *dZ*/*dt* signal in the original publication by Forouzanfar et al. (2018). In addition, we used different versions of the Q‐peak detection algorithm by Van Lien et al. (2013) which consists of a fixed time interval (original publication: 40 ms) subtracted from the R‐peak to determine the Q‐peak as the Q–R interval is expected to be stable over time, even if it varies across individuals (Van Lien et al. 2013). We tested different time intervals of $t\in \left\{32,34,36,38,40,42\right\}\;\mathrm{ms}$ to evaluate the impact of the time interval on the Q‐peak detection performance.

Many B‐point detection algorithms require the C‐point as a reference point, which is defined as the maximum of the dZ/dt signal within a cardiac cycle (also referred to as $\mathrm{dZ}/dt_{\max}$; Sherwood et al. 1990). Thus, we implemented a C‐point extraction algorithm based on the peak detection algorithm provided by the *scipy* Python package (Virtanen et al. 2020) (CPointExtractionScipyFindPeaks). Since the find_peaks method can detect multiple peaks within a cardiac cycle, we selected the peak with the smallest absolute difference to the mean R–C interval of the three preceding cardiac cycles as the C‐point. If no peak was detected, the C‐point was set to a missing value (*NaN*).

#### 2.5.2 Dealing With Missing Fiducial Points

Some of the algorithms fail to detect fiducial points when certain conditions are not met, such as when there are no local minima in the specified search interval of the dZ/dt signal. In these cases, the resulting fiducial points are set to a missing value (*NaN*).

#### 2.5.3 Combining Algorithms to Pipelines

The combination of a Q‐peak extraction algorithm, a B‐point extraction algorithm, and an optional outlier correction algorithm forms a *PEP extraction pipeline*. When applying this pipeline to the data, the signals are first segmented into individual cardiac cycles using the same method as described in Section 2.4, and C‐points are extracted from the *dZ*/*dt* signal. Both the Q‐peak extraction and B‐point extraction algorithms receive the segmented cardiac cycle borders as input, while the B‐point extraction algorithm additionally receives the C‐points as input. Afterwards, the outlier correction algorithm is applied to the extracted B‐point values (if specified). Finally, the PEP is computed for each cardiac cycle as follows:

$$\mathrm{PEP}=\mathrm{loc}\left(B‐\text{point}\right)-\mathrm{loc}\left(Q‐\text{peak}\right)$$

where $\mathrm{loc}\left(Q‐\text{peak}\right)$ and $\mathrm{loc}\left(B‐\text{point}\right)$ denote the locations of the Q‐point and B‐point, respectively, within the cardiac cycle in milliseconds.

Since the Q‐peak and B‐point extraction algorithms operate independently on the ECG and *dZ*/*dt* signals, respectively, the PEP extraction pipelines can yield negative PEP values if a B‐point is detected before the Q‐peak. To handle these cases, we introduce a parameter to the PepExtractionPipeline class, allowing configuration of whether negative PEP values should be set to 0 or to a missing value (*NaN*, the default).

### 2.6 Evaluation

#### 2.6.1 Creating Evaluation Challenges

By combining all possible algorithm combinations, we created a total of 108 individual PEP extraction pipelines. By including the different time intervals for the (Van Lien et al. 2013) Q‐peak detection algorithm, we increased the number of pipelines to 288, which we evaluated individually on the two datasets introduced in Section 2.2. The evaluation of the different PEP extraction pipelines is based on a standardized evaluation framework implemented in the *PEPbench* package, which allows the execution of various *challenges* on the two benchmark datasets.

Besides evaluating entire PEP extraction pipelines, which consist of a Q‐peak extraction algorithm, a B‐point extraction algorithm, and an optional outlier correction algorithm, we also examined the individual algorithms (Q‐peak extraction and B‐point extraction with outlier correction) separately. For this, we implemented PEP pipelines that use an automated extraction algorithm for one signal (ECG or *dZ*/*dt*) and the manual annotations for the other signal, respectively. This setup enabled the evaluation of the individual algorithms in isolation.

#### 2.6.2 PEPbench Evaluation Framework

For each challenge, the evaluation framework computes a set of evaluation metrics on different aggregation levels. These metrics are calculated with reference to the manually annotated PEP values and the automatically extracted PEP values from the different PEP extraction pipelines. Initially, the detected cardiac cycles were matched with the manually annotated cardiac cycles based on the borders of the cycles. A detected cardiac cycle was considered a match if the start and end borders were within a tolerance of 100 ms centered around the start and end borders of the manually annotated cardiac cycle. Evaluation metrics were computed based on the matched cardiac cycles (i.e., true positives). Unmatched cardiac cycles, which generally occurred at the beginning and end of the recordings, were considered false positives and excluded from the evaluation.

Subsequently, the Error (*E*), Absolute Error (AE), and Absolute Relative Error (ARE) were computed for each sample (i.e., each cardiac cycle). In these formulas, PEP_ref_ represents the *reference*, that is, the manually annotated PEP, and PEP_est_ represents the *estimated*, that is, automatically extracted PEP.

$$\begin{array}{c} E={\mathrm{PEP}}_{\mathrm{ref}}-{\mathrm{PEP}}_{\mathrm{est}}\\ \mathrm{AE}=\left|{\mathrm{PEP}}_{\mathrm{ref}}-{\mathrm{PEP}}_{\mathrm{est}}\right|\\ \mathrm{ARE}=\left|\frac{{\mathrm{PEP}}_{\mathrm{ref}}-{\mathrm{PEP}}_{\mathrm{est}}}{{\mathrm{PEP}}_{\mathrm{ref}}}\right| \end{array}$$

#### 2.6.3 Evaluation Metric Aggregations

Besides storing the per‐sample evaluation metrics for further analysis, we also computed aggregated evaluation metrics on single *datapoints*, which we refer to as all cardiac cycles for one condition and phase (*EmpkinS Dataset*) or all cardiac cycles of one participant for one phase (*Guardian Dataset*). For the error metrics, we computed the mean value over all cardiac cycles of one datapoint, resulting in mean error (ME), mean absolute error (MAE), and mean absolute relative error (MARE) metrics per datapoint.

In addition to the *per‐datapoint* aggregation, we directly aggregated the *per‐sample* error metrics by computing the mean and standard deviation over *all* cardiac cycles of the dataset without first aggregating them on the datapoint level. This approach ensures that each cardiac cycle is weighted equally in the evaluation process, allowing a direct comparison of the error metrics between different pipelines without weighting biases due to different numbers of cardiac cycles per datapoint. In the results section, we will, unless stated otherwise, report on the per‐sample error metrics.

Finally, we computed the number of *invalid* PEP values, that is, cardiac cycles where a pipeline failed to extract a valid PEP, as well as the *total* number of PEP per datapoint. We aggregated these values over the entire datasets by summing them up to obtain the total number of valid and invalid PEP values that were automatically extracted by the respective algorithm.

#### 2.6.4 Inter‐Rater Agreement

Since cardiac cycles were annotated by two individual raters, we computed inter‐rater reliability using the intraclass correlation coefficient $\mathrm{ICC}\left(3,2\right)$, which assesses the absolute agreement between two fixed raters averaging across measurements (McGraw and Wong 1996) to compare the consistency between annotators.

Because averaging the locations of fiducial points across raters is conceptually inappropriate (as it would result in non‐physiological intermediary values), we chose to report the results based on the annotations from Annotator 1. To still account for the potential influence of inter‐annotator variability, we showcase the effect of different annotations on the best‐performing individual algorithm and pipeline, respectively. Additionally, we investigated differences in algorithm estimation errors between cardiac cycles with high annotation agreement (i.e., both annotations differed not more than 5 ms), medium agreement (between 5 and 10 ms), and low agreement (larger than 10 ms).

### 2.7 Availability of Code and Data

The *PEPbench* framework is available on GitHub (https://github.com/empkins/pepbench) under the MIT license. This repository contains the source code of the Python package, as well as the code for all experiments and evaluations presented in this paper and the Supporting Information. Furthermore, the repository contains the documentation of the package, including a detailed description of the implemented algorithms and evaluation approach, as well as a user guide for contributing to the *PEPbench* framework. This includes guidance on adding new algorithms or datasets, as well as instructions for manually annotating the fiducial points using the MaD GUI (Ollenschläger et al. 2022).

The two datasets used in this experiment are available on the Open Science Framework (OSF) platform (*EmpkinS Dataset*: https://doi.org/10.17605/OSF.IO/SH3XN, *Guardian Dataset*: https://doi.org/10.17605/OSF.IO/GYH75).

## 3 Results

We structure the presentation of our results as follows: First, we summarize the manually annotated PEP values of both datasets to demonstrate the data basis of the reference values for the subsequent algorithm benchmarking. Afterwards, we present the results of the individual fiducial point extraction algorithms (*Q‐peak* or *B‐point* extraction algorithms, respectively) as outlined in Section 2.6. Finally, we present and evaluate the performance of combined PEP extraction pipelines.

### 3.1 Manually Annotated Reference Pre‐Ejection Period (PEP)

**FIGURE 2:**
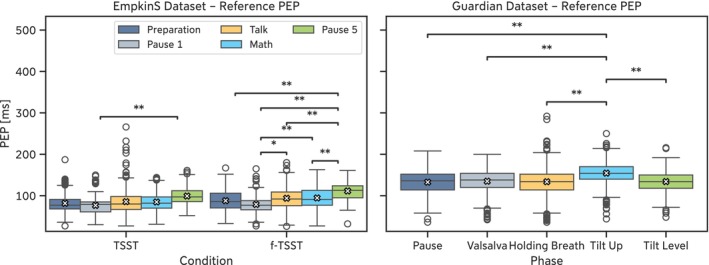
Reference PEP values for the *EmpkinS Dataset* (left) and the *Guardian Dataset* (right). Mean values are denoted by the white cross. Statistical tests were computed between the different phases using Wilcoxon signed‐rank tests with Bonferroni correction. Statistical significance is denoted as: *p < 0.05, **p < 0.01.

From the *EmpkinS Dataset*, 116 of the 5116 manually annotated cardiac cycles (2.3%) were labeled as artifacts by Annotator 1, resulting in 5000 cardiac cycles for further analysis. The average heart rate over the entire dataset was 101.5 ± 24.3 beats per minute (bpm), ranging between 47.2 bpm and 157.9 bpm. The average PEP over the entire dataset was 88.4 ± 25.0 ms, with differences between the individual phases in both conditions (Figure 2, Table 5a and 5b).

**TABLE 5a:** Summary of reference PEP values (as mean ± standard deviation [M ± SD]) for the different conditions and phases of the *EmpkinS Dataset*. The range is provided as [min, max].

EmpkinS Dataset				
Phase/Condition	f‐TSST		TSST	
	M±SD [ms]	Range [ms]	M±SD [ms]	Range [ms]
Preparation	87.95 ± 24.36	[33, 167]	81.69 ± 21.45	[27, 187]
Pause 1	78.85 ± 20.67	[27, 165]	76.61 ± 21.79	[30, 150]
Interview	93.88 ± 24.67	[26, 180]	85.70 ± 28.38	[27, 266]
Mental Arithmetic	94.59 ± 25.70	[27, 163]	84.87 ± 20.62	[31, 144]
Pause 5	111.49 ± 21.28	[32, 161]	99.21 ± 22.55	[52, 161]

**TABLE 5b:** Summary of reference PEP values (as mean ± standard deviation [M ± SD]) for the different phases of the *Guardian Dataset*. The range is provided as [min, max].

Guardian Dataset		
Phase	M ± SD [ms]	Range [ms]
Resting	132.71 ± 25.41	[36, 208]
Valsalva	134.99 ± 25.28	[42, 200]
Holding Breath	133.76 ± 30.32	[36, 292]
Tilt Up	154.49 ± 22.92	[44, 250]
Tilt Level	134.16 ± 23.87	[48, 216]

Similarly, 184 of the 6795 cardiac cycles (2.7%) in the *Guardian Dataset* were labeled as artifacts by Annotator 1, resulting in 6611 cardiac cycles for further analysis. For the *Guardian Dataset*, the average heart rate was 67.6 ± 13.4 bpm, ranging between 39.1 and 138.3 bpm. The average PEP over the entire dataset was 138.4 ± 27.1 ms. Similar to the *EmpkinS Dataset*, the PEP differed between the phases of the protocol in the *Guardian Dataset* due to the different interventions (Figure 2, Table 5a).

Annotator 2 labeled 368 out of 5116 cardiac cycles (7.2%) for the *EmpkinS Dataset* as artifacts and 101 out of 6795 cardiac cycles (1.4%) for the *Guardian Dataset*. Overall, the agreement between both annotators was excellent for both modalities and datasets (*EmpkinS Dataset*: ICC(3,2) = 0.997, 95% CI [1.00, 1.00] for Q‐peak annotations, ICC(3,2) = 0.986, 95% CI [0.99, 0.99] for B‐point annotations; *Guardian Dataset*: ICC(3,2) = 0.999, 95% CI [1.00, 1.00] for Q‐peak annotations, ICC(3,2) = 0.983, 95% CI [0.98, 0.98] for B‐point annotations). For the *EmpkinS Dataset*, the average annotation difference was −0.7 ± 4.5 ms with a range from −27.0 to +31.0 ms for the Q‐peaks and 4.6 ± 14.9 ms with a range of −60.0 to +88.0 ms for the B‐points. A total of 88.0% annotated Q‐peaks showed *high* annotation agreements, that is, both annotations differed by no more than 5 ms, while 6.2% and 5.8% showed *medium* (5–10 ms difference) and *low* (more than 10 ms difference) agreements, respectively. In contrast, 60.0% of the B‐point annotations showed *high* agreement (21.9% *medium*, 18.1% *low*). For the *Guardian Dataset*, the average annotations difference was −0.4 ± 3.0 ms with a range from −26.0 to +16.0 ms for the Q‐peaks and 3.6 ± 16.2 ms with a range of −96.0 to +128.0 ms for the B‐points. For the Q‐peaks annotations, 96.6% showed *high* annotation agreement (1.9% *medium*, 1.5% *low*), while 72.9% of the B‐point annotations showed *high* agreement (13.6% *medium*, 13.5% *low*).

More detailed information on the reference PEP values can be found in the Supporting Information (*EmpkinS Dataset*: Figures S3 and S4; *Guardian Dataset*: Figures S36 and S37).

### 3.2 Q‐peak Extraction Algorithms

**FIGURE 3:**
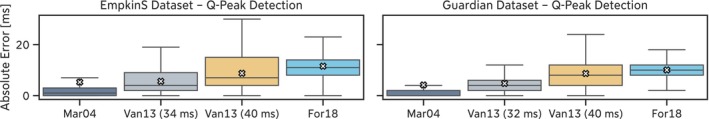
Absolute error of selected Q‐peak extraction algorithms on the *EmpkinS Dataset* (left) and the *Guardian Dataset* (right). For better visualization outliers exceeding 1.5 × IQR are not shown. The full boxplot including outliers can be found in the Supporting Information (Figures S5 and S38). Mean values are denoted by the white cross.

The Q‐peak extraction algorithms were separately evaluated on the *EmpkinS Dataset* and the *Guardian Dataset*. The results of the individual algorithms are summarized in Tables 5a and 6. For the *EmpkinS Dataset*, the *Mar04* (Martinez et al. 2004) Q‐peak extraction algorithm achieved the lowest MAE (5.3 ± 9.5 ms), followed by the *Van13* (Van Lien et al. 2013) algorithm with time differences t=34ms and t=32ms subtracted from the R‐peak, respectively (34 ms: 5.6 ± 4.4 ms; 32 ms: 5.7 ± 3.4 ms). Using the time difference originally proposed by Van Lien et al. (2013) (t=40ms) resulted in a higher MAE (8.8 ± 6.2 ms). While the algorithm by Martinez et al. (2004) achieved the lowest MAE, it exhibited higher error variance, greater sensitivity to outliers (Figure 3, left), and the highest number of missed Q‐peaks (Table 6).

**TABLE 6:** Results of the Q‐peak extraction algorithms on the *EmpkinS Dataset*.

Q‐peak algorithm	MAE (ms)	ME (ms)	MARE (%)	Invalid PEPs
Mar04	5.3 ± 9.5	−4.4 ± 10.0	6.8 ± 13.3	145 (2.9%)
Van13 (34 ms)	5.6 ± 4.4	−2.4 ± 6.7	7.4 ± 7.1	0 (0.0%)
Van13 (32 ms)	5.7 ± 3.5	−0.4 ± 6.7	7.3 ± 5.6	0 (0.0%)
Van13 (36 ms)	6.0 ± 5.3	−4.4 ± 6.7	8.1 ± 8.6	0 (0.0%)
Van13 (38 ms)	7.2 ± 5.8	−6.4 ± 6.7	9.6 ± 9.6	0 (0.0%)
Van13 (40 ms)	8.8 ± 6.2	−8.4 ± 6.7	11.6 ± 10.5	0 (0.0%)
Van13 (42 ms)	10.6 ± 6.4	−10.4 ± 6.7	13.9 ± 11.2	0 (0.0%)
For18	11.5 ± 5.9	11.3 ± 6.4	13.8 ± 7.1	3 (0.1%)

*Note:* The algorithms are sorted by the mean absolute error (MAE) in ascending order.

Similar results were observed for the *Guardian Dataset*, where the *Mar04* (Martinez et al. 2004) algorithm also achieved the lowest MAE (4.2 ± 12.4 ms), followed by the *Van13* (Van Lien et al. 2013) algorithm with t=32ms and t=34ms, respectively (t=32ms: 4.8 ± 5.3 ms; t=34ms: 5.0 ± 5.7 ms) (Figure 3, right, Table 7).

**TABLE 7:** Results of the Q‐peak extraction algorithms on the *Guardian Dataset*.

Q‐peak algorithm	MAE (ms)	ME (ms)	MARE (%)	Invalid PEPs
Mar04	4.2 ± 12.4	−3.1 ± 12.7	3.0 ± 9.2	113 (1.7%)
Van13 (32 ms)	4.8 ± 5.3	−0.5 ± 7.1	3.8 ± 5.1	0 (0.0%)
Van13 (34 ms)	5.0 ± 5.7	−2.5 ± 7.1	4.0 ± 5.5	0 (0.0%)
Van13 (36 ms)	5.7 ± 6.2	−4.5 ± 7.1	4.6 ± 6.0	0 (0.0%)
Van13 (38 ms)	7.0 ± 6.7	−6.5 ± 7.1	5.6 ± 6.4	0 (0.0%)
Van13 (40 ms)	8.7 ± 6.9	−8.5 ± 7.1	6.9 ± 6.8	0 (0.0%)
For18	10.1 ± 4.6	9.6 ± 5.4	7.5 ± 4.7	0 (0.0%)
Van13 (42 ms)	10.6 ± 7.0	−10.5 ± 7.1	8.3 ± 7.0	0 (0.0%)

*Note:* The algorithms are sorted by the mean absolute error (MAE) in ascending order.

Across both datasets, the *For18* algorithm proposed by Forouzanfar et al. (2018) performed considerably worse compared to the other algorithms. In comparison to all other algorithms, we observed that the *For18* algorithm tended to estimate Q‐peak locations too late, resulting in an underestimation of the PEP and, thus, in a positive mean error. In contrast, all other algorithms showed a negative mean error, indicating an overestimation of the PEP due to early Q‐peak detection (Tables 6 and 7).

A deeper investigation into the performance of the algorithms suggests that the *Mar04* (Martinez et al. 2004) algorithm is more susceptible to deviations in the ECG morphology, as the MAE of the algorithm varied more between participants compared to the other algorithms (see Supporting Information, *EmpkinS Dataset*: Table S2, Figures S6–S11; *Guardian Dataset*: Table S16, Figures S39–S44). This participant‐level variability likely reflects differences in ECG signal characteristics such as QRS amplitude, noise levels, and waveform shape, which can vary due to factors like body composition, electrode placement, or individual cardiac physiology. In comparison, the *Van13* algorithm proposed by Van Lien et al. (2013) demonstrated a more stable performance across participants. However, the approach of subtracting a fixed time difference from the R‐peak to estimate the Q‐peak location resulted in increasing estimation errors with higher heart rates in the *EmpkinS Dataset* (r=0.42,p<0.001) but not in the *Guardian Dataset* (r=0.01,p=0.234) (see Supporting Information, *EmpkinS Dataset*: Figures S12 and S13; *Guardian Dataset*: Figures S45 and S46).

Comparing the algorithm performance metrics between the different reference annotations showed small differences. For the *Mar04* algorithm, the estimation difference between both annotators was 5.3 ± 9.5 ms versus 5.8 ± 9.4 ms in the *EmpkinS Dataset* and 4.2 ± 12.4 ms versus 5.1 ± 12.7 ms in the *Guardian Dataset* (other algorithms: see Tables S3 and S17). For Q‐peaks with high annotation agreement, the MAE was considerably lower compared to Q‐peaks with medium or low agreement for both datasets (Tables S4 and S18). Across all algorithms, the overall average absolute difference between both annotators was 0.4 ± 0.3 ms for the *EmpkinS Dataset* and 0.4 ± 0.3 ms for the *Guardian Dataset*.

### 3.3 B‐point Extraction Algorithms

**FIGURE 4:**
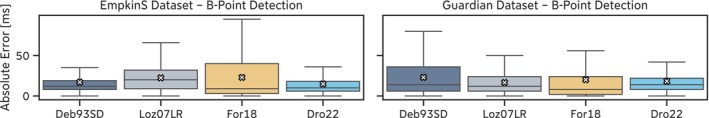
Absolute error of selected B‐point extraction algorithms (without outlier correction) on the *EmpkinS Dataset* (left) and the *Guardian Dataset* (right). For better visualization outliers exceeding 1.5 × IQR are not shown. The full boxplot including outliers can be found in the Supporting Information (Figures S14 and S47). Mean values are denoted by the white cross.

For the *EmpkinS Dataset*, the *Dro22* (Drost et al. 2022) algorithm achieved the lowest MAE (14.9 ± 14.7 ms), followed by the *Deb93SD* (Debski et al. 1993) algorithm (17.1 ± 16.8 ms) and the *Mil22* (Miljković and Šekara 2022) algorithm (19.8 ± 20.4 ms; Table 8, Figure 4, left). Compared to the other two algorithms, the *Deb93SD* algorithm failed more frequently to detect the B‐point, resulting in a higher number of invalid PEPs. The highest MAE was observed for the *Arb17SD* algorithm (Árbol et al. 2017) at 41.9 ± 21.2 ms, along with a MARE of 47.6% ± 22.8%. The *Pal21* (Pale et al. 2021) algorithm demonstrated the highest rate of invalid PEPs, failing to detect the B‐point in 29.2% of cardiac cycles. For the *Guardian Dataset*, the *Loz07LR* (Lozano et al. 2007) algorithm performed best, yielding the lowest MAE (16.7 ± 14.8 ms), followed by the *Dro22* algorithm (18.2 ± 15.2 ms) and the *For18* (Forouzanfar et al. 2018) algorithm (20.1 ± 29.5 ms; Table 9, Figure 4, right). Although the *Deb93SD* algorithm showed the second‐lowest MAE on the *EmpkinS Dataset*, its performance was poorer on the *Guardian Dataset*. However, it exhibited fewer invalid PEPs (8.2% vs. 5.8%). Overall, the number of invalid PEPs was higher in the *Guardian Dataset* than in the *EmpkinS Dataset*, regardless of the algorithm. The highest percentage of invalid PEPs was recorded for the *Pal21* algorithm (Pale et al. 2021), which failed to detect the B‐point in 37.7% of cardiac cycles. Detailed reasons for invalid PEPs are presented in Table 10.

**TABLE 8:** Results of the B‐point extraction algorithms (without outlier correction) on the *EmpkinS Dataset*.

B‐point algorithm	MAE (ms)	ME (ms)	MARE (%)	Invalid PEPs
Dro22	14.9 ± 14.7	−10.1 ± 18.4	18.8 ± 19.9	56 (1.1%)
Deb93SD	17.1 ± 16.8	5.3 ± 23.4	21.8 ± 26.6	411 (8.2%)
Mil22	19.8 ± 20.4	−2.2 ± 28.3	24.3 ± 26.6	190 (3.8%)
Loz07QR	21.6 ± 15.0	−16.9 ± 20.2	30.5 ± 30.6	39 (0.8%)
Loz07LR	22.4 ± 16.1	−20.2 ± 18.8	31.9 ± 31.9	29 (0.6%)
For18	22.9 ± 26.5	21.2 ± 27.9	25.8 ± 28.1	299 (6.0%)
Arb17TD	23.7 ± 18.4	−19.7 ± 22.6	30.5 ± 29.4	393 (7.9%)
She90	23.8 ± 14.7	−11.6 ± 25.4	30.0 ± 22.6	291 (5.8%)
Arb17IC	24.4 ± 16.0	−9.9 ± 27.4	30.6 ± 22.8	136 (2.7%)
Ste85	26.2 ± 30.9	24.1 ± 32.6	28.8 ± 32.5	792 (15.8%)
Pal21	37.5 ± 25.3	−14.3 ± 42.9	46.5 ± 34.1	1458 (29.2%)
Arb17SD	41.9 ± 21.2	39.2 ± 25.9	47.6 ± 22.8	286 (5.7%)

*Note:* The metrics were computed only on valid PEP values, that is, cardiac cycles for which a valid B‐point could be detected. Invalid PEPs (e.g., due to physiologically implausible locations or failed detections) were excluded from the metric calculations and are reported separately in the last column. Algorithms are sorted by mean absolute error (MAE) in ascending order.

**TABLE 9:** Results of the B‐point extraction algorithms (without outlier correction) on the Guardian Dataset.

B‐point algorithm	MAE (ms)	ME (ms)	MARE (%)	Invalid PEPs
Loz07LR	16.7 ± 14.8	−4.1 ± 21.9	14.4 ± 19.2	379 (5.7%)
Dro22	18.2 ± 15.2	−14.8 ± 18.6	15.5 ± 19.0	384 (5.8%)
For18	20.1 ± 29.5	11.2 ± 33.9	14.8 ± 22.5	572 (8.7%)
Ste85	21.5 ± 33.7	18.7 ± 35.4	16.0 ± 25.2	554 (8.4%)
Deb93SD	23.0 ± 23.9	−9.2 ± 31.8	19.1 ± 25.0	385 (5.8%)
Mil22	24.3 ± 29.7	9.4 ± 37.2	17.9 ± 22.0	395 (6.0%)
Loz07QR	27.1 ± 18.7	20.2 ± 25.9	19.4 ± 14.8	383 (5.8%)
Arb17IC	32.8 ± 22.5	4.2 ± 39.6	25.3 ± 19.7	388 (5.9%)
She90	33.3 ± 22.5	4.4 ± 39.9	25.7 ± 19.8	424 (6.4%)
Arb17TD	34.0 ± 34.6	1.3 ± 48.5	26.7 ± 28.9	1209 (18.3%)
Arb17SD	43.1 ± 21.9	38.0 ± 30.0	31.5 ± 15.5	392 (5.9%)
Pal21	50.6 ± 40.5	3.6 ± 64.8	39.6 ± 33.6	2494 (37.7%)

*Note:* The metrics were computed only on valid PEP values, that is, cardiac cycles for which a valid B‐point could be detected. Invalid PEPs (e.g., due to physiologically implausible locations or failed detections) were excluded from the metric calculations and are reported separately in the last column. Algorithms are sorted by mean absolute error (MAE) in ascending order.

**TABLE 10:** Overview of invalid PEP values for B‐point detection algorithms and the underlying reasons.

Reason	Inv. B Window	Neg. PEP		No C		No Iso. Cross.	No Loc. Min.		No Mon. Incr.		No Z‐Cross.	
Algorithm/Dataset	Guardian	EmpkinS	Guardian	EmpkinS	Guardian	EmpkinS	EmpkinS	Guardian	EmpkinS	Guardian	EmpkinS	Guardian
Arb17IC	0	116	21	5	367	15	0	0	0	0	0	0
Arb17SD	0	281	25	5	367	0	0	0	0	0	0	0
Arb17TD	2	388	840	5	367	0	0	0	0	0	0	0
Deb93SD	0	0	0	5	367	0	406	18	0	0	0	0
Dro22	0	51	17	5	367	0	0	0	0	0	0	0
For18	0	254	81	14	478	0	0	0	27	7	0	0
Loz07LR	0	24	12	5	367	0	0	0	0	0	0	0
Loz07QR	0	34	16	5	367	0	0	0	0	0	0	0
Mil22	0	185	28	5	367	0	0	0	0	0	0	0
Pal21	0	1453	2127	5	367	0	0	0	0	0	0	0
She90	0	0	0	5	367	0	0	0	0	0	286	57
Ste85	0	765	185	5	367	0	22	2	0	0	0	0

Abbreviations: Inv. B Window = Invalid B‐point search window determined, Neg. PEP = negative PEP computed (i.e., B‐point was detected before reference Q‐peak), No C = No C‐point detected, No Iso. Cross. = No isoelectric crossing before C‐point detected, No Loc. Min. = No local minimum detected within, No Mon. Incr. = No monotonically increasing segment detected, No Z‐Cross. = No zero crossings detected.

For both datasets, there was no observed influence of individual participants, experimental phases, heart rate, BMI, or age on algorithm performance (Supporting Information, *EmpkinS Dataset*: Table S5, Figures S15–S21; *Guardian Dataset*: Table S19, Figures S48–S54).

**FIGURE 5:**
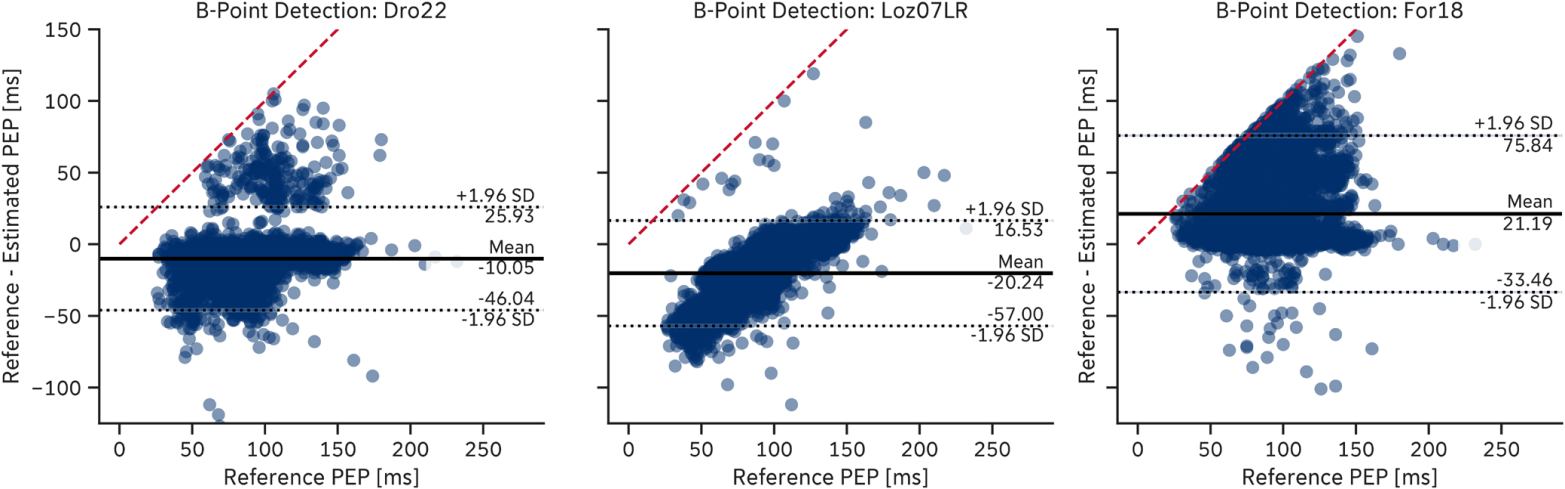
Residual plots of selected B‐point extraction algorithms on the *EmpkinS* Dataset. The red dashed line indicates the upper estimation error limit given by the location of the reference Q‐peaks.

A more detailed analysis revealed that the regression‐based B‐point extraction algorithms proposed by Lozano et al. (2007) (*Loz07LR* and *Loz07QR*) exhibited error dependency on the reference PEP. Shorter PEP values led to overestimation of the B‐point location, whereas longer PEP values resulted in underestimation (Figure 5). More generally, some algorithms tended to estimate the B‐point location too early (e.g., *For18*, *Ste85*, *Arb17SD*), resulting in *positive* mean errors, while others, such as *Dro22* and *Loz07LR*, estimated it too late, leading to *negative* mean errors. Notably, some algorithms, including the *For18* algorithm, detected the B‐point so early that it was clipped by the Q‐peak location, thereby imposing an upper limit on estimation error determined by the reference PEP values (e.g., *EmpkinS Dataset*: Figure 5, red dashed line). This early detection also led to a general underestimation of PEP (Tables 8 and 9). Conversely, the lower estimation error limit for most algorithms was determined by the detected C‐point location.

The effect of different annotators on the B‐point algorithm detection performance was small, with an overall average absolute difference across all algorithms of 3.0 ± 1.4 ms for the *EmpkinS Dataset* and 1.6 ± 1.3 ms for the *Guardian Dataset*. For the *Dro22* algorithm, the estimation difference between both annotators was 14.9 ± 14.7 ms versus 17.4 ± 15.1 ms in the *EmpkinS Dataset* and 18.2 ± 15.2 ms versus 20.4 ± 19.7 ms in the *Guardian Dataset* (other algorithms: see Tables S7 and S21). Similar to the Q‐peak detection, B‐points with high annotation agreement showed a considerably lower MAE compared to B‐points with medium or low agreement for both datasets (Tables S8 and S22).

### 3.4 Effect of B‐point Outlier Correction

Applying different outlier correction algorithms to B‐point outlier candidates reduced error metrics across several algorithms (e.g., *Ste85*, *She90*, *Arb17SD*, *For18*, and *Mil22*).

**FIGURE 6:**
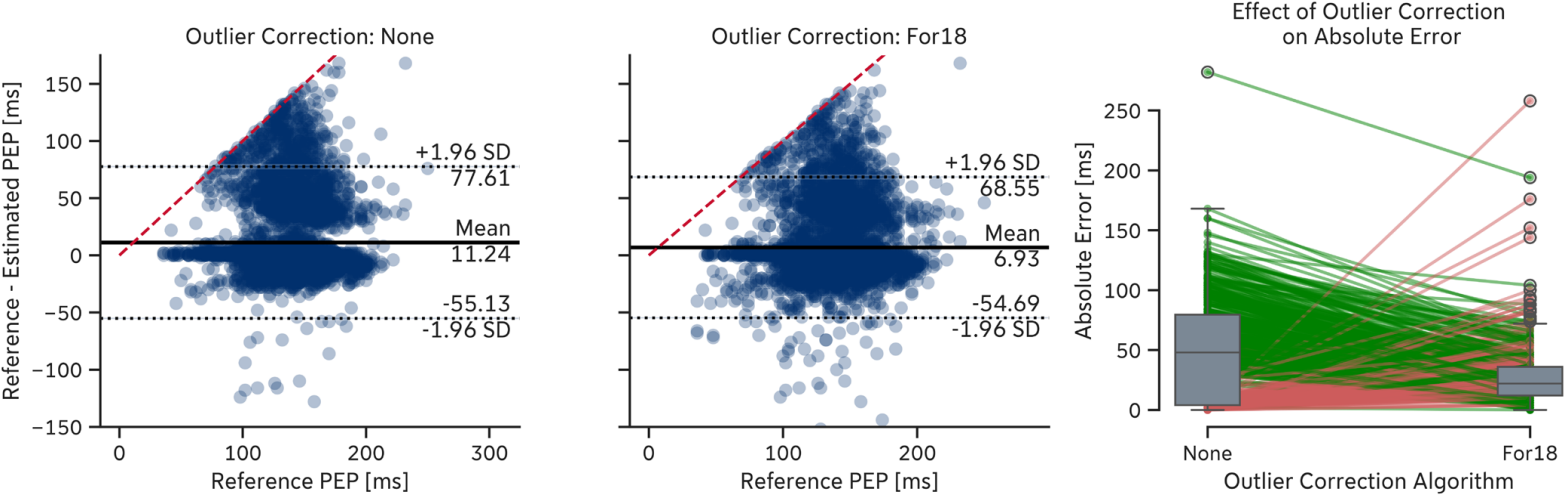
Effect of outlier correction on the performance of the *For18* algorithm for the Guardian Dataset. Left: Residual plot without outlier correction. Center: Residual plot with outlier correction according to Forouzanfar et al. (2018). Right: Paired plot of the PEP values extracted by the *For18* algorithm without and with outlier correction. Green lines indicate a reduction in AE after outlier correction, red lines an increase. PEPs not affected by outlier correction are not shown for better visualization.

For the *For18* algorithm, applying the *LinInt* outlier correction algorithm to the extracted B‐points improved the MAE from 22.9 ± 26.5 ms to 21.4 ± 25.6 ms on the *EmpkinS Dataset* (Table 11; Supporting Information, Figures S22–S25) and from 20.1 ± 29.5 ms to 18.3 ± 26.1 ms on the *Guardian Dataset* (Table 12; Supporting Information, Figures S55–S58). Using the *For18* outlier correction algorithm on the extracted B‐points of the *For18* algorithm showed a similar improvement in MAE to 21.8 ± 25.7 ms on the *EmpkinS Dataset* and to 18.5 ± 26.4 ms on the *Guardian Dataset* (Figure 6, left and center). For the *Guardian Dataset*, 5.9% of all pre‐ejection periods (PEPs) exhibited a reduction in absolute error (AE) after outlier correction, 3.2% exhibited an increase in AE, and 91.0% were unaffected by the outlier correction algorithm (Figure 6, right).

**TABLE 11:** MAE of the outlier correction algorithms on the B‐point extraction algorithms on the *EmpkinS Dataset*.

B‐point algorithm	Outlier correction algorithm		
	None	LinInt	For18
Dro22	**14.9 ± 14.7**	15.4 ± 14.8	15.2 ± 14.7
Deb93SD	17.1 ± 16.8	17.1 ± 17.3	**16.9 ± 17.1**
Mil22	19.8 ± 20.4	19.1 ± 19.0	**19.0 ± 18.9**
Loz07QR	**21.6 ± 15.0**	22.6 ± 16.5	22.5 ± 16.4
Loz07LR	**22.4 ± 16.1**	23.7 ± 18.3	23.6 ± 18.2
For18	22.9 ± 26.5	**21.4 ± 25.6**	21.8 ± 25.7
Arb17TD	**23.7 ± 18.4**	25.2 ± 18.8	25.0 ± 18.7
She90	23.8 ± 14.7	23.5 ± 15.5	**23.3 ± 15.3**
Arb17IC	24.4 ± 16.0	23.4 ± 15.3	**23.3 ± 15.3**
Ste85	26.2 ± 30.9	**24.2 ± 28.9**	24.5 ± 29.0
Pal21	**37.5 ± 25.3**	38.0 ± 25.0	37.8 ± 24.8
Arb17SD	41.9 ± 21.2	**38.8 ± 20.2**	39.4 ± 20.4

*Note:* The algorithms are sorted by the AE in ascending order. The lowest MAE values per algorithm are highlighted in bold. MAE values are provided in milliseconds (M ± SD).

**TABLE 12:** MAE of the outlier correction algorithms on the B‐point extraction algorithms on the *Guardian Dataset*.

B‐point algorithm	Outlier correction algorithm		
	None	LinInt	For18
Loz07LR	**16.7 ± 14.8**	17.2 ± 15.9	17.1 ± 15.8
Ste85	21.5 ± 33.7	**17.6 ± 28.2**	18.3 ± 29.2
Dro22	**18.2 ± 15.2**	19.4 ± 16.2	19.1 ± 16.0
For18	20.1 ± 29.5	**18.3 ± 26.1**	18.5 ± 26.4
Mil22	24.3 ± 29.7	**22.2 ± 26.9**	22.5 ± 27.2
Deb93SD	**23.0 ± 23.9**	24.3 ± 24.5	23.6 ± 24.1
Loz07QR	27.1 ± 18.7	**26.4 ± 18.3**	26.4 ± 18.3
Arb17IC	32.8 ± 22.5	**31.3 ± 21.7**	31.4 ± 21.8
She90	33.3 ± 22.5	31.3 ± 21.5	**31.2 ± 21.5**
Arb17TD	34.0 ± 34.6	29.5 ± 27.9	**29.1 ± 28.2**
Arb17SD	43.1 ± 21.9	**41.3 ± 22.1**	41.7 ± 22.0
Pal21	50.6 ± 40.5	**48.1 ± 37.6**	49.1 ± 38.5

*Note:* The algorithms are sorted by the AE in ascending order. The lowest MAE values per algorithm are highlighted in bold. MAE values are provided in milliseconds (M ± SD).

Although these improvements were evident for the *For18* algorithm, the outlier correction algorithms showed inconsistent effects on the performance of other B‐point extraction algorithms (*Deb93SD*, *Loz07QR*, and *Arb17TD*) and consistently worsened the performance of the *Dro22* and *Loz07LR* algorithms (Tables 11 and 12). For the *Dro22* algorithm, the *For18* outlier correction algorithm slightly increased the MAE from 14.9 ± 14.7 ms to 15.2 ± 14.7 ms on the *EmpkinS Dataset* and from 18.2 ± 15.2 ms to 19.1 ± 16.0 ms on the *Guardian Dataset*. This effect is also reflected in the percentage of PEPs affected by the outlier correction algorithm, where only 2.7% (*EmpkinS Dataset*) and 1.2% (*Guardian Dataset*) of PEPs showed a reduction in AE after outlier correction, while 7.7% (*EmpkinS Dataset*) and 6.4% (*Guardian Dataset*) of PEPs showed an increase in AE.

Despite these inconsistencies, the outlier correction algorithms consistently reduced the number of invalid PEPs for all algorithms on both datasets, thereby improving the robustness of the algorithms (Supporting Information, *EmpkinS Dataset*: Table S6, *Guardian Dataset*: Table S20).

### 3.5 Combined PEP Extraction Pipelines

When combining the Q‐peak and B‐point extraction algorithms into a joint pipeline to estimate the PEP, the lowest MAE (10.8 ± 15.6 ms) on the *EmpkinS Dataset* was achieved by a pipeline consisting of the *For18* Q‐peak extraction algorithm and the *Dro22* B‐point extraction algorithm, without outlier correction applied. This pipeline (*For18 | Dro22 | None*) is referred to as the *Overall Lowest Error Pipeline* as it achieved the combined PEP extraction pipeline's overall lowest MAE. The same pipeline also attained the lowest MAE on the *Guardian Dataset* (11.6 ± 15.9 ms) (Table 13, Supporting Information, Tables S9 and S23). We did not observe consistent systematic errors, such as biases across participants, experimental phases, heart rate, age, or BMI, for both datasets (Supporting Information, *EmpkinS Dataset*: Figures S27–S33; *Guardian Dataset*: Figures S60–S66). Similar to the individual algorithms, the pipeline performances vary between participants (Supporting Information, *EmpkinS Dataset*: Table S10, *Guardian Dataset*: Table S24).

**TABLE 13:** Results of selected combined PEP extraction pipelines per dataset.

Dataset	Type	Q‐peak algorithm	B‐point algorithm	Outlier correction	MAE (ms)	ME (ms)	MARE (%)	Invalid PEPs
EmpkinS	OLE	For18	Dro22	None	10.8 ± 15.6	1.1 ± 19.0	13.0 ± 18.4	68 (1.4%)
	ILE	Mar04	Dro22	None	18.9 ± 17.6	−14.5 ± 21.5	24.3 ± 26.1	200 (4.0%)
Guardian	OLE	For18	Dro22	None	11.6 ± 15.9	−5.0 ± 19.0	10.1 ± 17.8	385 (5.8%)
	ILE	Mar04	Loz07LR	None	18.3 ± 16.6	−6.5 ± 23.8	15.7 ± 20.6	481 (7.3%)

*Note:* The different pipeline types are: OLE = overall lowest error, that is, the combined PEP pipelines with the overall lowest MAE on the EmpkinS Dataset and the Guardian Dataset, respectively; ILE = individually lowest error, that is, the combined PEP pipeline consisting of the algorithms with the individually lowest MAE. Results of all PEP pipelines are available in the Supporting Information, Tables S11 (EmpkinS Dataset) and S25 (Guardian Dataset).

On both datasets, the *Overall Lowest Error Pipeline* outperformed the *Individually Lowest Error Pipeline*, which comprised the algorithms with the lowest MAE values when evaluated separately on Q‐peaks and B‐points. On the *EmpkinS Dataset*, the *Individually Lowest Error Pipeline* was *(Mar04 | Dro22 | None)* with an MAE of 18.9 ± 17.6 ms (Table 13, Supporting Information, Figures S34 and S35). On the *Guardian Dataset*, the *Individually Lowest Error Pipeline* was *(Mar04 | Loz07LR | None)* with an MAE of 18.3 ± 16.6 ms (Table 13, Supporting Information, Figures S67 and S68).

**FIGURE 7:**
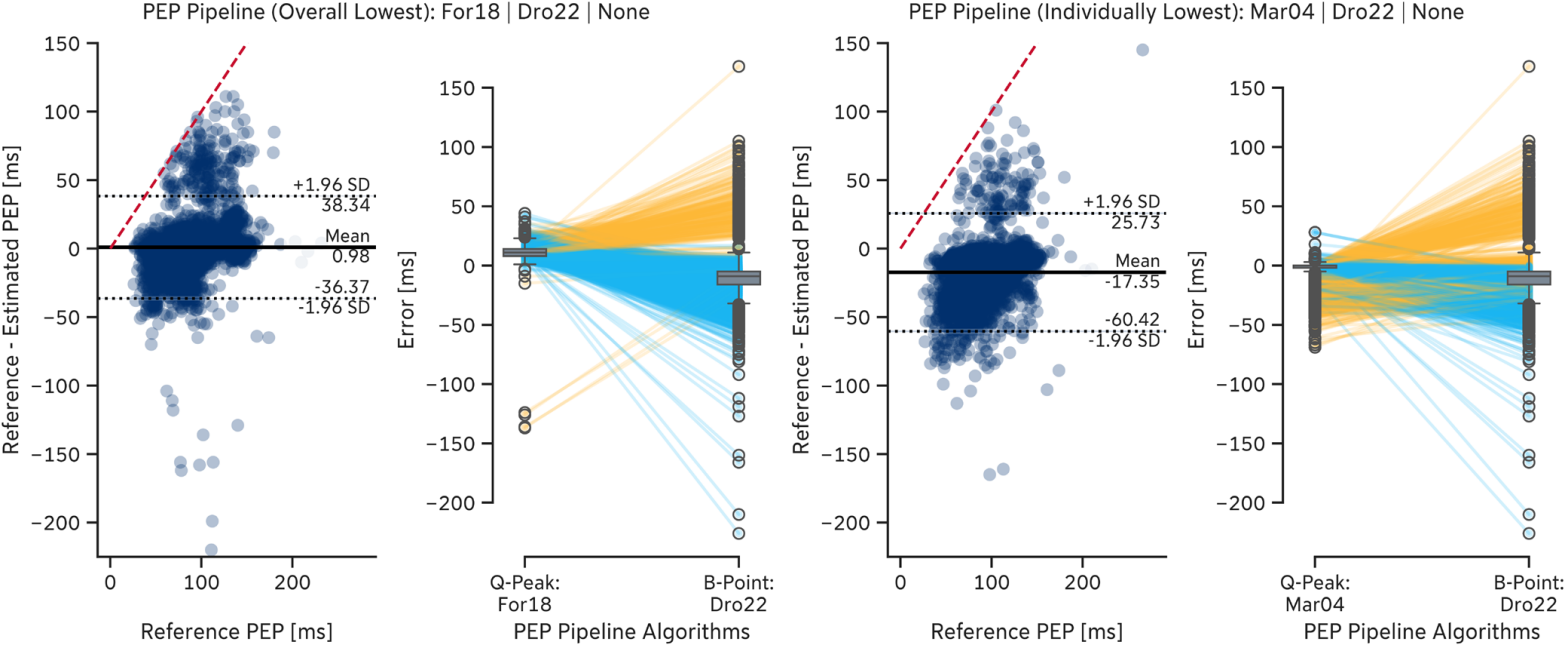
Evaluation results of the combined PEP extraction pipelines on the *EmpkinS Dataset*. Left: Overall Lowest Error Pipeline, that is, pipeline with the overall lowest MAE; Right: Individually Lowest Error Pipeline, that is, pipeline consisting of the algorithms with the individually lowest MAE. For each pipeline, residual plots (left) indicate the error between the estimated and reference PEP values and paired plots (right) illustrate the error propagation between the Q‐peak and B‐point extraction algorithms. Yellow lines indicate a positive change between the Q‐peak and B‐point extraction algorithm, while blue lines indicate a negative change, respectively.

**FIGURE 8:**
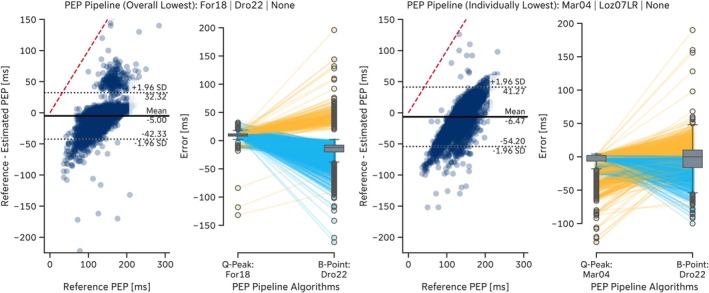
Evaluation results of the combined PEP extraction pipelines on the *Guardian Dataset*. Left: Overall Lowest Error Pipeline, that is, pipeline with the overall lowest MAE; Right: Individually Lowest Error Pipeline, that is, pipeline consisting of the algorithms with the individually lowest MAE. For each pipeline, residual plots (left) indicate the error between the estimated and reference PEP values and paired plots (right) illustrate the error propagation between the Q‐peak and B‐point extraction algorithms. Yellow lines indicate a positive change between the Q‐peak and B‐point extraction algorithm, while blue lines indicate a negative change, respectively.

A deeper analysis of error propagation between the Q‐peak and B‐point extraction algorithms revealed that individual estimation errors seem to be partially compensated within the combined pipeline. The *For18* Q‐peak extraction algorithm tends to estimate Q‐peak locations too late, resulting in an underestimation of the PEP and a positive error bias. Conversely, the *Dro22* B‐point extraction algorithm tends to estimate B‐point locations too late, leading to PEP overestimation and a negative error bias (Figures 7 and 8). In contrast, both algorithms in the *Individually Lowest Error Pipelines* exhibited positive error biases, leading to error accumulation within the combined pipeline.

For both the *Overall Lowest Error Pipeline* and the *Individually Lowest Error Pipeline*, applying outlier correction algorithms did not enhance the performance of the combined PEP extraction pipelines for most cases (Tables S11 and S25). However, the outlier correction algorithms did reduce the number of invalid PEP values, improving the pipelines' robustness.

Similar to the individual algorithms, the combined PEP pipelines exhibit only small differences when using the annotations performed by the second annotator. For the *Overall Lowest Error Pipeline*, the difference was 10.8 ± 15.6 ms versus 10.7 ± 14.1 ms for the *EmpkinS Dataset* and 11.6 ± 15.9 ms versus 13.5 ± 19.2 ms for the *Guardian Dataset*, respectively (Tables S12 and S26). Cardiac cycles with high agreement between the two annotators showed a considerably lower MAE compared to cardiac cycles with medium or low agreement for both Q‐peaks and B‐points and both datasets, respectively (Tables S13, S14, S27, and S28).

## 4 Discussion

In this paper, we introduced *PEPbench*, an open‐source Python package containing various algorithms for automated beat‐to‐beat PEP extraction from ECG and *dZ*/*dt* signals. Along with the package, we presented a systematic comparison of these algorithms on two different datasets, which we made publicly available, to facilitate the evaluation and comparison of PEP extraction algorithms. In the following, we discuss the key findings of our work, as well as the implications and limitations of our study and outline future research directions.

### 4.1 The Datasets Offer a First Testbed for Benchmarking PEP Extraction Algorithms

The two datasets used for evaluating PEP extraction algorithms differ in study design, measurement device, and participant characteristics, providing a useful variability of conditions for our initial benchmarking efforts.

While limited in overall size and diversity, both datasets include distinct (psycho‐)physiological manipulations that elicited changes in the measured PEP, as reflected by a broad distribution of PEP values across experimental phases. The average PEP values were within the normal physiological range (60–170 ms) in all different phases of both datasets (Árbol et al. 2017; Hodges et al. 1972; Houtveen et al. 2005; Weissler et al. 1968).

In the *EmpkinS Dataset*, the average heart rate was considerably higher than in the *Guardian Dataset* and the average PEP was shorter. This can be attributed to the different participant characteristics and experimental conditions in the two studies. In the *EmpkinS Dataset*, participants were, on average, almost 8 years younger, which can have an impact on both heart rate and PEP (Cybulski 1996). Furthermore, measurements in the *EmpkinS Dataset* were taken in a standing position. In contrast, measurements in the *Guardian Dataset* were conducted in a supine position, which can influence cardiovascular dynamics due to the orthostatic stress response (Hnatkova et al. 2019; Van Zanten et al. 2024). Lastly, the psychosocial stress induction in the *EmpkinS Dataset*, characterized by elements such as social‐evaluative threat and lack of control, elicits complex autonomic response patterns involving both sympathetic and parasympathetic modulation. In contrast, the Valsalva maneuver and tilt‐table tests employed in the *Guardian Dataset* typically produce more phasic, short‐lived cardiovascular responses. These responses involve both sympathetic activation and pronounced mechanical effects on cardiac preload and afterload, which can also influence PEP. Accordingly, changes in PEP during these tests should not be interpreted as purely SNS‐mediated. These physiological differences likely contributed to the observed variance in PEP across datasets and are relevant for interpreting algorithm performance under different autonomic response profiles (Goldstein et al. 2011). These contextual differences provide a useful contrast in autonomic response profiles for testing algorithm robustness.

In summary, the datasets used in our work for benchmarking PEP extraction algorithms are a good starting point for evaluating algorithm performance on different populations and recording conditions. The datasets come with manual annotations of the Q‐peak and B‐point, making them suitable for validating and comparing beat‐to‐beat PEP extraction algorithms. Due to the large number of total cardiac cycles in the datasets, we only annotated subsets of the data, which may limit the generalizability of our results to the entire datasets. Nonetheless, the segments were selected randomly while considering the distribution of the data in the different phases of the experiment, which should ensure that the annotated segments are representative of the entire dataset. However, we acknowledge that the current datasets do not capture the full range of participant characteristics or measurement contexts, particularly those involving seated recordings, older adults, or clinical populations. These limitations underscore the need for future contributions that extend the benchmark to more diverse datasets.

To strengthen the reliability of the ground truth annotations, we added a second independent annotator who annotated both datasets. The inter‐rater agreement was very high for Q‐peaks and B‐points, and the overall algorithm performance results were consistent across both annotators. Although small differences between annotators were observed, they did not substantially affect the overall benchmarking conclusions. Cardiac cycles with high agreement between the two annotators showed markedly lower errors than those with medium or low agreement, underscoring the importance of annotator consistency for algorithm evaluation. These findings also point to the influence of signal quality on both manual annotation and algorithm performance: Cardiac cycles with lower inter‐rater agreement likely reflect noisy or ambiguous waveform morphology, which poses challenges for both human raters and automated algorithms. Improving signal quality, through sensor design, preprocessing, or artifact rejection, remains a key avenue for reducing estimation errors and improving the reliability of PEP extraction methods.

By publishing the annotated datasets under an open‐source license, we aim to provide a robust and transparent foundation for future research in PEP extraction, which can build upon our work by manually annotating more data segments in our datasets or even adding new datasets to the benchmarking framework. We further encourage the research community to not only contribute additional annotations or datasets, but also to clearly document their scoring approaches, so that any resulting biases can be understood in context. This can help to continuously improve the benchmarking framework and facilitate more reliable, generalizable comparisons across PEP extraction methods.

Another potential limitation of our annotation approach arises from the visual guidance provided during the manual labeling process. Specifically, the simultaneous plotting of the ECG signal and the use of the Q‐peak as a lower limit for the B‐point annotation might have unintentionally introduced bias. While this method ensured that only physiologically plausible B‐points were annotated in the *dZ*/*dt* signal, as the annotated B‐points consistently followed their corresponding Q‐peaks in the ECG signal, it also meant that the rater viewed both signals simultaneously. This approach is standard practice in impedance cardiography, as the Q‐peak and B‐point are physiologically linked through the sequence of ventricular depolarization and aortic valve opening. Although concurrent visualization could, in principle, introduce a perceptual coupling bias, it reflects the natural temporal dependence of these events and supports physiologically valid identification of the B‐point. Therefore, we consider this procedure appropriate and consistent with established guidelines (2017).

### 4.2 Q‐peak Detection Algorithms Are Robust Across Datasets

In both datasets, the Q‐peak detection algorithms proved to be robust and showed similar performance. The Q‐peak detection algorithm *Mar04*, proposed by Martinez et al. (2004), showed the best performance in terms of MAE on both datasets, indicating that it can deal with the variability across different populations and recording conditions. With 2.9% (*EmpkinS Dataset*) and 1.7% (*Guardian Dataset*) of cardiac cycles containing Q‐peak detection errors, the *Mar04* algorithm showed reliable performance in both datasets. However, it is more prone to differences in the ECG waveform morphology, which is reflected in a higher standard deviation compared to the other algorithms due to higher inter‐participant variability. Changes to the ECG waveform morphology can occur due to factors like electrode placement, patient movement, or individual anatomical differences. Similarly, the pre‐processing steps applied to the ECG signal, such as filtering, baseline correction, or artifact removal, can also affect Q‐peak detection performance. In our work, we applied the same pre‐processing steps to the ECG signals in both datasets, which are commonly used in the literature (Happold et al. 2021; Makowski et al. 2021; Richer et al. 2022). However, the impact of different filtering approaches on Q‐peak detection performance has not been investigated in this work and remains an open question for future research. Using our *PEPbench* framework, researchers can easily implement pre‐processing approaches and compare their impact on Q‐peak detection performance across different datasets.

Besides the *Mar04* algorithm, the other algorithms also showed good performance in terms of MAE on both datasets. On both datasets, the *Van13* algorithm (Van Lien et al. 2013) achieved a comparable MAE to the *Mar04* algorithm with similar best‐performing time intervals t as parameters (34 ms for the *EmpkinS Dataset* and 32 ms for the *Guardian Dataset*), both of which are lower than the originally proposed time interval of t=40ms (Van Lien et al. 2013). This time interval in the original publication was chosen to localize the Q‐wave *onset* in the ECG signal. In contrast, we decided to use and manually annotate the Q‐wave *peak* as the start point of the PEP in our work, which is more robust and easier to detect than the Q‐wave onset (Berntson et al. 2004) but occurs slightly later in the ECG signal. This can explain the shorter time interval t for the Q‐peak detection algorithms in our work compared to the original publication. The time difference between the Q‐wave onset and the Q‐wave peak typically ranges from 10 to 20 ms, depending on heart rate and individual morphology (Berntson et al. 2004). This aligns well with the observed reduction in the optimal time interval t in our study.

The *Van13* algorithm had a lower standard deviation compared to the *Mar04* algorithm, indicating that it is less sensitive to changes in the ECG waveform morphology since it relies on detecting the R‐peak, the most prominent and easiest‐to‐detect feature in the ECG signal, with many algorithms available for its reliable detection (Makowski et al. 2021). However, detecting the R‐peak and subtracting a fixed time interval t to localize the Q‐peak can introduce a systematic bias in the PEP values, which needs to be considered when using this algorithm (Berntson et al. 2004). Especially in the *EmpkinS Dataset*, which has a larger heart rate range compared to the *Guardian Dataset*, the *Van13* algorithm showed a higher error dependency on heart rate, which can be attributed to the fixed time interval t used for Q‐peak localization.

In comparison to the *Mar04* and *Van13* algorithms, the *For18* algorithm had a higher MAE on both datasets and was the only algorithm with a positive mean error, indicating that the Q‐peak location was estimated too late in the cardiac cycle. Since the Q‐wave *onset* occurs *before* the Q‐wave *peak*, the estimations by the *For18* algorithm reflect the actual start of the PEP worse than the other algorithms.

In summary, the robust performance of the Q‐peak detection algorithms across datasets indicates that the choice of algorithm for Q‐peak detection has a minor impact on PEP extraction accuracy. However, the impact still needs to be considered when analyzing PEP data extracted with automated algorithms. In future work, the impact of different pre‐processing steps on Q‐peak detection performance should be further investigated.

### 4.3 B‐point Detection Remains a Major Source of Error in Automatic PEP Extraction

B‐point detection has long been recognized as a major source of error and variability in automatic PEP extraction, and it is widely considered the primary challenge in ensuring reliable PEP measurements (Cieslak et al. 2018; Kelsey and Guethlein 1990; Sherwood et al. 1990). Our results support and extend this understanding by systematically comparing B‐point detection algorithms across datasets and identifying approaches that address this key limitation. Among the tested algorithms, the *Dro22* B‐point detection algorithm (Drost et al. 2022) emerged as the most robust, achieving the best performance on the *EmpkinS Dataset* and the second‐best performance on the *Guardian Dataset* in terms of MAE. The *Dro22* algorithm showed consistent behavior across different datasets, participants, and experimental phases. Additionally, no systematic errors were observed that were dependent on heart rate or the reference PEP values. This suggests that the *Dro22* algorithm is the most suitable candidate for automated PEP extraction, as it effectively handles the variability in the *dZ*/*dt* waveform morphology across different populations and recording conditions. Unlike most other algorithms, which primarily rely on the detection of extrema in the *dZ*/*dt* signal or its derivatives, the *Dro22* algorithm identifies the B‐point based on a geometric approach. Specifically, it locates the point with the maximum distance along the y‐axis between the *dZ*/*dt* signal and a straight line connecting the C‐point (the maximum of the *dZ*/*dt* signal within the cardiac cycle) and the point on the *dZ*/*dt* signal 150 ms earlier. This method appears to be more robust to noise and artifacts in the *dZ*/*dt* signal, which can interfere with the reliable detection of extrema.

Another set of algorithms that did not rely on extrema detection was proposed by (Lozano et al. 2007). These models employ linear and quadratic regression techniques to detect the B‐point based on the C‐point and the ECG R‐peak. The regression coefficients were derived from prior datasets and are applied as fixed constants to new data, rather than recalculating them for each individual or dataset. This means the approach assumes that a single set of constants is sufficiently generalizable across different populations and recording conditions. The linear regression model (*Loz07LR*) showed good performance on the *EmpkinS Dataset* (ranked fourth) and achieved the lowest MAE on the *Guardian Dataset*. However, the purely data‐driven approach of the *Loz07LR* algorithm might be less robust to PEP values that are at the extrema of the physiological range. This could explain its higher error rates in the *EmpkinS Dataset* compared to the *Guardian Dataset*, where PEP values were shorter, and heart rates were higher. This finding is supported by the linear relationship between error and reference PEP values for the *Loz07LR* algorithm in the *EmpkinS Dataset* (Figure 5). Future work could explore adapting the regression constants to specific populations or recording contexts and systematically investigate the effects of such adjustments on detection accuracy.

Most B‐point extraction algorithms we investigated require the C‐point to be detected first, which then serves as the basis for the subsequent B‐point detection algorithms. In our work, we used an automatic C‐point detection algorithm based on a peak detection algorithm from the *scipy* package. Even though this approach has been shown to be reliable for detecting the C‐point since it is the most prominent fiducial point in the dZ/dt signal, it can still introduce errors in the PEP extraction process, as detecting the C‐point incorrectly can influence the subsequent B‐point detection. To eliminate this potential source of error, future work should consider manual verification of the C‐point location and compare it to the performance of the automatic detection algorithm.

As part of our attempt to improve the performance of the B‐point detection algorithms, we also implemented two different outlier correction algorithms that aim to correct the B‐point location either through linearly interpolating the B‐point location of outliers based on the neighboring B‐point locations (*LinInt*) or through an auto‐regressive model (*For18*). Applying outlier correction resulted in an improvement in the MAE for seven out of the twelve B‐point detection algorithms on the *EmpkinS Dataset* and for nine out of the twelve algorithms on the *Guardian Dataset*. Even though the *For18* auto‐regressive model is more complex (both in terms of algorithmic and computational complexity) than the *LinInt*, it performed similarly compared to the *LinInt* algorithm. Even in combination with the *For18* B‐point detection algorithm, as originally published by Forouzanfar et al. (2018), the *LinInt* outlier correction approach showed better performance in terms of MAE on both datasets. Despite the outlier correction algorithms not significantly improving the performance of the B‐point detection algorithms, they can still be useful to correct the B‐point location in cases where the B‐point detection algorithm fails to detect the B‐point correctly, effectively reducing the number of invalid PEP values. Thus, the outlier correction algorithms can be considered a valuable post‐processing step to improve the overall reliability of the extracted PEP for a given dataset by identifying and correcting invalid PEP values.

Comparing the two datasets revealed that the PEP extraction failed in considerably more cardiac cycles in the *Guardian Dataset* compared to the *EmpkinS Dataset*, resulting in more invalid PEP values. One possible explanation for this might be the greater amount of movement elicited by the physiological manipulations, such as during tilt‐table transitions or the initiation of the Valsalva maneuver, which can affect signal quality, especially in the dZ/dt signal. Investigating whether invalid detections systematically coincide with these movement‐intensive moments would be highly relevant for future work. However, the current analysis is limited in this regard, as only randomly selected subsets of each experimental phase were annotated, making it difficult to compare the timing of invalid detections with specific events within the phase, such as movement onset.

An important finding is that the impact of outlier correction varied considerably across algorithms in our evaluation. In some cases, outlier correction even led to reduced performance. This highlights that the effectiveness of outlier correction is not universal and may strongly depend on the specific error patterns and characteristics of each algorithm. A more detailed, algorithm‐specific analysis of when and how outlier correction provides benefits, or when it may even introduce additional errors, is highly relevant and should be investigated in future research. Given the scope of this manuscript, such in‐depth analyses were beyond our focus, but we encourage further work to systematically investigate when and how to apply outlier correction in PEP extraction pipelines or even exploring the use of novel outlier correction algorithms.

In general, the B‐point detection algorithms showed considerably higher error rates and error variability compared to those of the Q‐peak detection algorithms. Even though the errors may appear low for the best‐performing algorithms (14.9 and 16.7 ms, respectively), the relative errors are considerable (18.8% and 14.4%, respectively). Since the physiological range of the PEP is relatively small (60–170 ms; Hodges et al. 1972), this can have a significant impact on the interpretation of the PEP values. This is particularly relevant in studies that rely on high temporal precision, such as beat‐to‐beat analyses or investigations aiming to detect subtle, short‐term changes in sympathetic cardiac control. As summarized by Albinet et al. (2024), even an error of approximately 15 ms can considerably influence the interpretation of psychophysiological findings, especially in settings with small expected effects. Therefore, while the *Dro22* algorithm demonstrated the most robust performance in our benchmark, its residual error must still be carefully considered when selecting PEP extraction methods for highly sensitive analyses.

The B‐point detection errors can be attributed to the nature of the B‐point, which is often a subtle inflection signal before the upstroke in the dZ/dt signal that can be difficult to detect due to noise, artifacts, or changes in the dZ/dt waveform morphology (Lozano et al. 2007). This confirms collective findings from previous work indicating that B‐point detection is a major source of error in automated PEP extraction and needs to be improved to achieve reliable beat‐to‐beat PEP values. However, the B‐point detection algorithms are also more challenging to develop compared to the Q‐peak detection algorithms due to the complexity of the dZ/dt signal and the variability in the dZ/dt waveform morphology. Furthermore, the extraction task is generally difficult due to the small time intervals involved. Assuming a sampling rate of 500 Hz, as present in the *Guardian Dataset*, misdetecting the B‐point by a single sample already leads to a 2 ms error in the PEP value, which translates to a relative error of 1.2%–2.7%, given the physiological range of the PEP (75–170 ms). While this sampling rate is sufficient for most applications, including our work, future work should re‐investigate the performance of the B‐point detection algorithms on datasets with higher sampling rates to eliminate this potential source of error.

### 4.4 Separate Algorithm Evaluation Helps to Understand the Individual Contributions to the Overall PEP Extraction Accuracy

By evaluating the Q‐peak and B‐point detection algorithms separately, instead of only evaluating combined PEP extraction pipelines as a whole, we were able to gain a deeper understanding of the individual contributions of the algorithms to the overall PEP extraction accuracy. We observed that the best‐performing PEP extraction pipelines were different from constructing a PEP extraction pipeline consisting of the *individually* best‐performing Q‐peak and B‐point detection algorithms. The PEP extraction pipelines that achieved the lowest mean absolute error (MAE) on both datasets were combinations of algorithms that both over‐ or underestimated the location of the Q‐peak and B‐point, respectively, for example, a combination of algorithms with negative mean error for the Q‐peak detection and positive mean error for the B‐point detection (or vice versa). Such combinations reduce the overall error of the PEP extraction pipeline, resulting in values that are closer to the reference PEP. However, this does not necessarily mean that the extracted PEP values accurately represent the true physiological PEP. Thus, it is important to consider the individual contributions of the Q‐peak and B‐point detection algorithms to the overall PEP.

In our evaluation, we mainly focused on beat‐to‐beat PEP extraction as this is the most fine‐grained level of analysis, allowing us to obtain the most detailed insights into the algorithm performances, but also representing the most challenging task for automated algorithms. However, in many applications, it may not be necessary to extract PEP on a beat‐to‐beat basis. Instead, extracting PEP from ensemble‐averaged data or averaging PEP values over specific time windows or experimental phases with shared variance (e.g., during the same task or physiological state) may be sufficient. Such approaches can reduce the impact of random errors in the Q‐peak and B‐point detection algorithms and thereby improve the reliability of the extracted PEP values. This is supported by our results, which showed that many PEP pipelines achieved a lower mean error than the mean absolute error when aggregated, suggesting that the errors in the Q‐peak and B‐point detection algorithms are not systematic and can cancel each other out when averaging across multiple cardiac cycles or epochs (Table 13).

### 4.5 Open Science‐Driven Algorithm Benchmarking Can Advance Psychophysiological Research

When comparing the rankings of the B‐point detection algorithms on the two datasets, we observed that the algorithms with the lowest MAE were not always the most recently published ones. This contrasts with trends in other fields, where novel algorithms often outperform older ones in terms of accuracy, reliability, or computational efficiency (Smith et al. 2013). For the B‐point detection algorithms we evaluated, this pattern did not hold, suggesting that the systematic comparison of PEP extraction algorithms has been underdeveloped, leaving substantial room for improvement.

Additionally, our benchmarking approach demonstrated that the best‐performing algorithms are not necessarily the most complex or computationally expensive ones. For example, the *For18* algorithm can be considered the most complex algorithm we investigated, since the B‐point detection is based on a set of complex rules derived from the dZ/dt signal and its derivatives (Forouzanfar et al. 2018). However, it performed worse than other algorithms that detect the B‐point based on simpler principles. In many cardiac cycles, the B‐points were even detected at the borders of the search range, which is the default fallback if no reliable B‐point candidate can be detected. This leads to a high standard deviation for the *For18* algorithm as well as to many cardiac cycles that were at the upper estimation limit of the B‐point detection range. This indicates that the complexity of the algorithm does not necessarily lead to better performance, but that the robustness of the algorithm to noise and artifacts in the dZ/dt signal is more important for reliable B‐point detection. Thus, future work should not only focus on developing more complex algorithms but especially on algorithms that handle noise and artifacts in the dZ/dt signal well.

This emphasizes the importance of establishing such an endeavor to drive progress in the field of psychophysiology using the principles of open science. To the best of our knowledge, our work is the first to establish such a comprehensive benchmarking framework for PEP extraction algorithms and the first time that different algorithms have been systematically compared. Furthermore, this is the first time that the implementation of these algorithms has been made publicly available, allowing researchers to reproduce our results and build upon our work, and the first time that datasets containing ECG and dZ/dt signals with manual event annotations have been made publicly available.

We acknowledge that the current version of *PEPbench* includes only two datasets with specific experimental conditions (e.g., supine and standing positions), which clearly do not represent the full range of contexts typically encountered in psychophysiological research. However, our primary goal was to introduce *PEPbench* as a technically robust, open‐source framework for the systematic and reproducible benchmarking of PEP extraction algorithms. Rather than aiming to draw definitive physiological conclusions, our work intends to highlight the variability in algorithm performance and demonstrate the urgent need for standardized, transparent evaluation tools. The modular and extensible design of *PEPbench* ensures that additional datasets can be readily integrated in the future to enhance generalizability and coverage, as demonstrated with the integration of the publicly available *ReBeatICG* (Pale et al. 2021) and *TimeWindowICG* (Miljković and Šekara 2023) datasets.

## 5 Conclusion and Outlook

In this paper, we introduced *PEPbench* as the first open‐source, reproducible, and systematic benchmarking framework for beat‐to‐beat PEP extraction algorithms. The framework provides the most comprehensive collection of algorithms for Q‐wave detection, B‐point detection, and B‐point outlier correction from the literature, which can all be accessed through a unified Python interface. *PEPbench* also includes two publicly available datasets with annotated ECG and dZ/dt signals for the evaluation and comparison of PEP extraction algorithms, as well as a set of evaluation metrics and visualization tools for assessing algorithm performance. Through the systematic comparison of algorithms on these datasets, we gained insights into the strengths and limitations of different methods and identified the most promising algorithm combinations for PEP extraction. Our benchmarking approach also demonstrated that, even though many different algorithm combinations have been proposed in the literature so far, most of them should be used with caution for automated PEP extraction on a beat‐to‐beat level as their error rates are still relatively high.

Thus, our work can also have important implications for clinical settings beyond the methodological contributions. In particular, the errors in B‐point detection, even when using the most robust algorithms, can influence the interpretation of cardiac sympathetic activity, especially in applications where precision and reliability are critical because subtle changes are assessed. It is therefore essential to recognize that while automated algorithms facilitate large‐scale and efficient PEP extraction, careful quality control and an awareness of potential algorithmic limitations remain necessary. Further improvements in detection accuracy, as well as context‐specific validation studies, will be crucial for safely translating these methods into applied or clinical use. Taken together, these limitations might contribute to the current situation of why many researchers continue to use manual annotation, often in combination with (semi‐)automated methods, to balance efficiency with reliability.

In the future, we aim to improve the framework by including additional datasets with manual annotations as well as further algorithms for PEP extraction from the literature. Furthermore, we plan to extend this type of benchmarking framework beyond PEP extraction to support the evaluation of algorithms for other cardiac parameters that are of interest in psychophysiological research, such as the left ventricular ejection time (LVET; Tavakolian 2016) or T‐wave amplitude (TWA; Drost et al. 2022). This way, we hope to establish a community‐driven platform for the development, evaluation, and comparison of algorithms for cardiac parameter extraction, which will foster innovation and collaboration in the field of psychophysiology and beyond. Combining this with other established psychophysiological measures, such as RMSSD or HF‐HRV as examples for vagally‐mediated HRV indices, could further enhance the framework's ability to assess both branches of the autonomic nervous system in an integrated manner, when the conditions are appropriate for making such inferences. This would support the development of more comprehensive pipelines for autonomic assessment, facilitating a better understanding of the dynamic interplay between sympathetic and parasympathetic activity.

Most importantly, however, we hope that we will not contribute to this endeavor alone. Through the release of *PEPbench*, we hope to encourage other researchers to contribute their own algorithms to the framework, share their data and annotations, and engage in the evaluation and comparison of algorithms for cardiac parameter extraction. By providing a common ground for the evaluation and comparison of PEP extraction algorithms and beyond, we aim to accelerate the development of new methods, enhance the accuracy and reliability of existing ones, and ultimately improve the quality of research findings in the field of psychophysiology through a more transparent and collaborative approach.

## Author Contributions

**Robert Richer:** conceptualization, data curation, formal analysis, investigation, methodology, software, validation, visualization, writing – original draft, writing – review and editing. **Julia Jorkowitz:** data curation, formal analysis, investigation, software, validation, visualization, writing – original draft, writing – review and editing. **Sebastian Stühler:** data curation, formal analysis, investigation, methodology, software, validation, visualization, writing – original draft, writing – review and editing. **Luca Abel:** conceptualization, data curation, formal analysis, methodology, software, validation, writing – original draft, writing – review and editing. **Miriam Kurz:** data curation, investigation, methodology, resources, writing – review and editing. **Marie Oesten:** data curation, methodology, writing – review and editing. **Stefan G. Griesshammer:** data curation, resources, writing – review and editing. **Nils C. Albrecht:** data curation, writing – review and editing. **Arne Küderle:** methodology, software, validation, visualization, writing – review and editing. **Christoph Ostgathe:** conceptualization, funding acquisition, resources, supervision, writing – review and editing. **Alexander Kölpin:** conceptualization, funding acquisition, supervision, writing – review and editing. **Tobias Steigleder:** conceptualization, funding acquisition, investigation, resources, supervision, writing – review and editing. **Nicolas Rohleder:** conceptualization, funding acquisition, investigation, resources, supervision, writing – review and editing. **Bjoern M. Eskofier:** conceptualization, funding acquisition, investigation, supervision, writing – review and editing.

## Ethics Statement

Both studies in this manuscript were approved by the local ethics committee of FAU (protocol #493_20 B and #264_17 B) and were conducted in accordance with the Declaration of Helsinki.

## Conflicts of Interest

The authors declare no conflicts of interest.

## Supporting information


**Data S1:** psyp70176‐sup‐0001‐supinfo.pdf.

## Data Availability

The two datasets used in this experiment are available on the Open Science Framework (OSF) platform (EmpkinS Dataset: https://doi.org/10.17605/OSF.IO/SH3XN, Guardian Dataset: https://doi.org/10.17605/OSF.IO/GYH75). The source code of the pepbench framework is available on GitHub (https://github.com/empkins/pepbench).
