# Supplementary material for: PEPbench—Open, Reproducible, and Systematic Benchmarking of Automated Pre‐Ejection Period Extraction Algorithms
Source: Psychophysiology. 2025 Nov 11;62(11):e70176. doi: 10.1111/psyp.70176 (PMC12605688; doi:10.1111/psyp.70176)
Supplement: Supplementary file 1 — Data S1: psyp70176‐sup‐0001‐supinfo.pdf. [file PSYP-62-e70176-s001.pdf]

Supplementary Material for Paper "PEPbench – Open, Reproducible, and Systematic Benchmarking of Automated Pre-Ejection Period Extraction Algorithms"

R. Richer, J. Jorkowitz, S. Stühler, L. Abel, M. Kurz, M. Oesten, S. Griesshammer, N. C. Albrecht, A. Küderle, C. Ostgathe, A. Kölpin, T. Steigleder, N. Rohleder, B. M. Eskofier

Contents

1 Overview of Study Procedures **3**

2 Manual Annotation of the PEP using the *MaD GUI* software **3**

3 *EmpkinS Dataset* **4**
3.1 Reference PEP Values . . . 4
3.2 Q-Peak Detection . . . 5
3.2.1 Residual Plots – Overall and Detailed . . . 6
3.2.2 Effect of ECG Waveform on Q-Peak Detection Performance . . . 8
3.2.3 Effect of Heart Rate on Q-Peak Detection Error . . . 9
3.2.4 Effect of Annotations on Q-Peak Detection Error . . . 10
3.2.5 Effect of Annotation Agreement on Q-Peak Detection Error . . . 10
3.3 B-Point Detection . . . 11
3.3.1 Residual Plots – Overall and Detailed . . . 12
3.3.2 Effect of Heart Rate on B-Point Detection Error . . . 13
3.3.3 Effect of Outlier Correction on B-Point Detection Performance . . . 15
3.3.4 Effect of Annotations on B-Point Detection Error . . . 18
3.3.5 Effect of Annotation Agreement on B-Point Detection Error . . . 19
3.4 Combined PEP Pipelines . . . 20
3.4.1 Results of all PEP Pipelines . . . 20
3.4.2 Absolute Error . . . 26
3.4.3 Residual Plots – Overall and Detailed . . . 27
3.4.4 Effect of Heart Rate on PEP Pipeline Extraction Error . . . 28
3.4.5 Effect of Outlier Correction on PEP Pipeline Performance . . . 30
3.5 Individually Lowest vs. Overall Lowest PEP Pipelines . . . 32
3.5.1 Effect of Annotations on B-Point Detection Error . . . 33
3.5.2 Effect of Annotation Agreement on PEP Pipeline Detection Error . . . 42

4 *Guardian Dataset* **43**
4.1 Reference PEP Values for the *Guardian Dataset* . . . 43
4.2 Q-Peak Detection . . . 44
4.2.1 Residual Plots – Overall and Detailed . . . 45
4.2.2 Effect of ECG Waveform on Q-Peak Detection Performance . . . 47
4.2.3 Effect of Heart Rate on Q-Peak Detection Error . . . 48
4.2.4 Effect of Annotations on Q-Peak Detection Error . . . 49
4.2.5 Effect of Annotation Agreement on Q-Peak Detection Error . . . 49
4.3 B-Point Detection . . . 50
4.3.1 Residual Plots – Overall and Detailed . . . 51
4.3.2 Effect of Heart Rate on B-Point Detection Error . . . 53
4.3.3 Effect of Outlier Correction on B-Point Detection Performance . . . 54
4.3.4 Effect of Annotations on B-Point Detection Error . . . 57
4.3.5 Effect of Annotation Agreement on B-Point Detection Error . . . 58
4.4 Combined PEP Pipelines . . . 59

4.4.1 Results of all PEP Pipelines . . . 59
4.4.2 Absolute Error . . . 65
4.4.3 Residual Plots – Overall and Detailed . . . 66
4.4.4 Effect of Heart Rate on PEP Pipeline Extraction Error . . . 68
4.4.5 Effect of Outlier Correction on PEP Pipeline Performance . . . 69
4.5 Individually Lowest vs. Overall Lowest PEP Pipelines . . . 71
4.5.1 Effect of Annotations on B-Point Detection Error . . . 72
4.5.2 Effect of Annotation Agreement on PEP Pipeline Detection Error . . . 81

1 Overview of Study Procedures

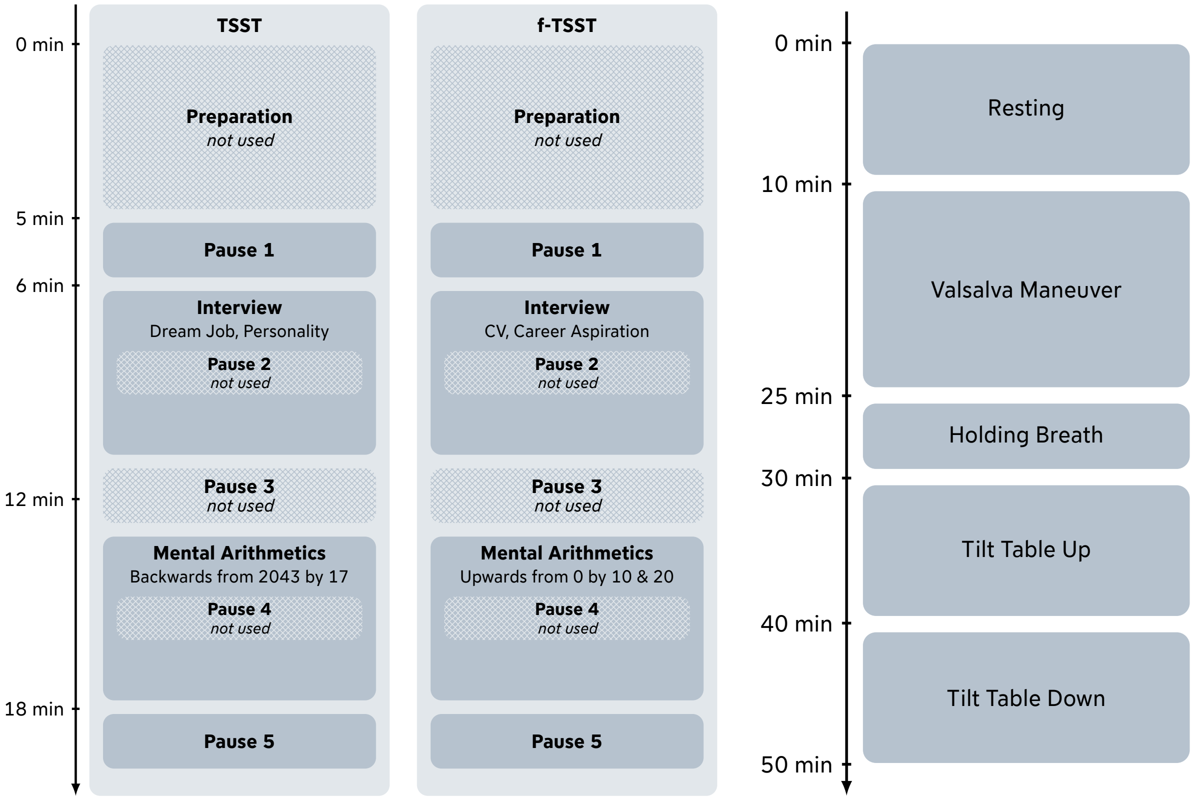

(a) EmpkinS Dataset (b) Guardian Dataset

Figure S1: Overview of the study procedures for the data recording of the *EmpkinS* and *Guardian* datasets.

2 Manual Annotation of the PEP using the *MaD GUI* software

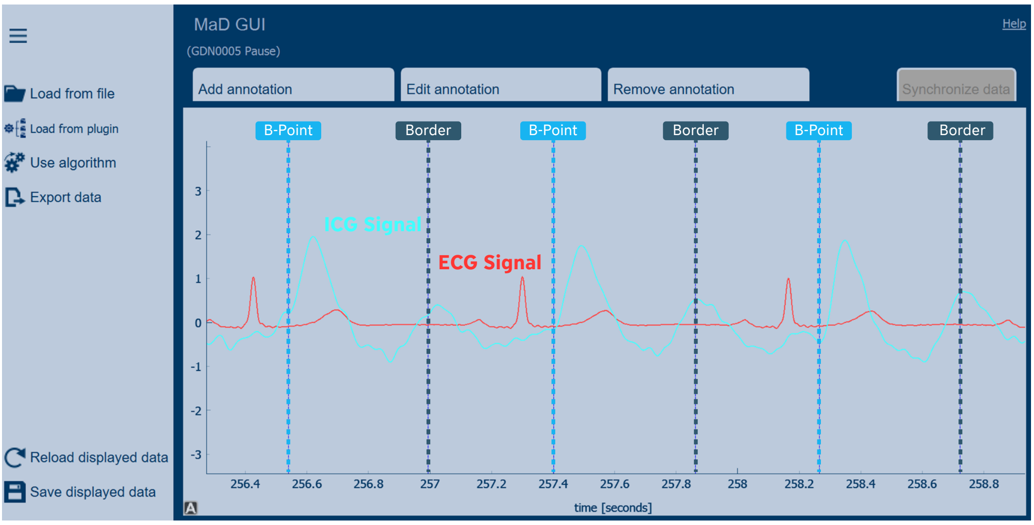

Figure S2: Screenshot of the *MaD GUI* [1] software for manual annotation of the PEP. The software allows the user to simultaneously load the ECG (red) and ICG (cyan) signals, to zoom in and out, and to manually label the Q-peaks (*not displayed here*) and B-points (blue) in the signals. The borders of the cardiac cycles (grey) are detected during the pre-processing step and automatically added by the software to facilitate the labeling process.

3 *EmpkinS Dataset*

3.1 Reference PEP Values

Table S1: Summary of reference pre-ejection period values for the different conditions and phases of the *EmpkinS Dataset*. The range is provided as [min, max].

Condition	Phase	M ± SD [ms]	Range [ms]
TSST	Preparation	81.69 ± 21.45	[27, 187]
	Pause 1	76.61 ± 21.79	[30, 150]
	Talk	85.70 ± 28.38	[27, 266]
	Math	84.87 ± 20.62	[31, 144]
	Pause 5	99.21 ± 22.55	[52, 161]
f-TSST	Preparation	87.95 ± 24.36	[33, 167]
	Pause 1	78.85 ± 20.67	[27, 165]
	Talk	93.88 ± 24.67	[26, 180]
	Math	94.59 ± 25.70	[27, 163]
	Pause 5	111.49 ± 21.28	[32, 161]

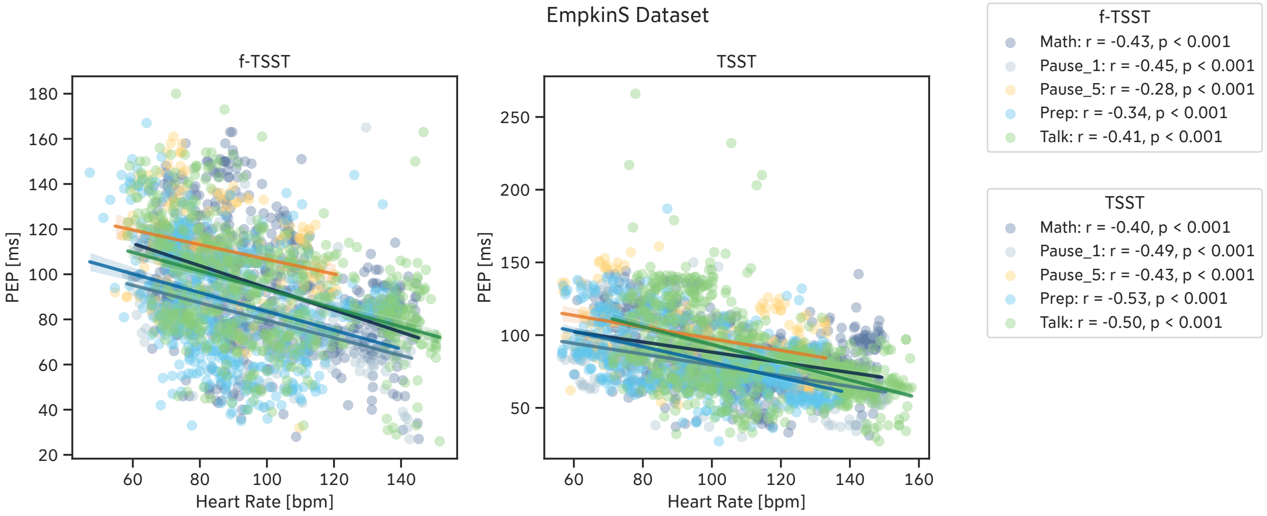

Figure S3: Relationship between Reference PEP and Heart Rate for the *EmpkinS Dataset*. The black line represents the linear regression fit with the corresponding 95% confidence interval.

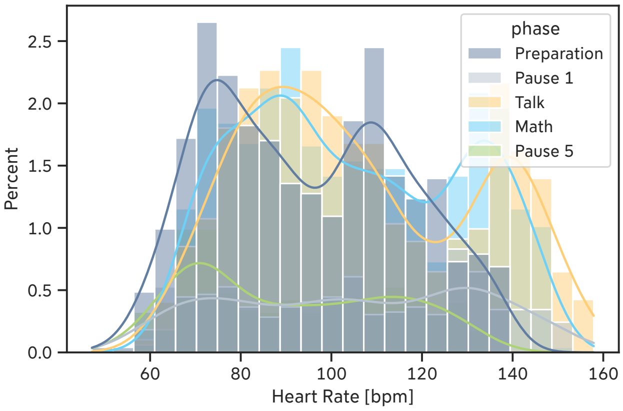

Figure S4: Distribution of heart rate values in the *EmpkinS Dataset*, divided by phase.

3.2 Q-Peak Detection

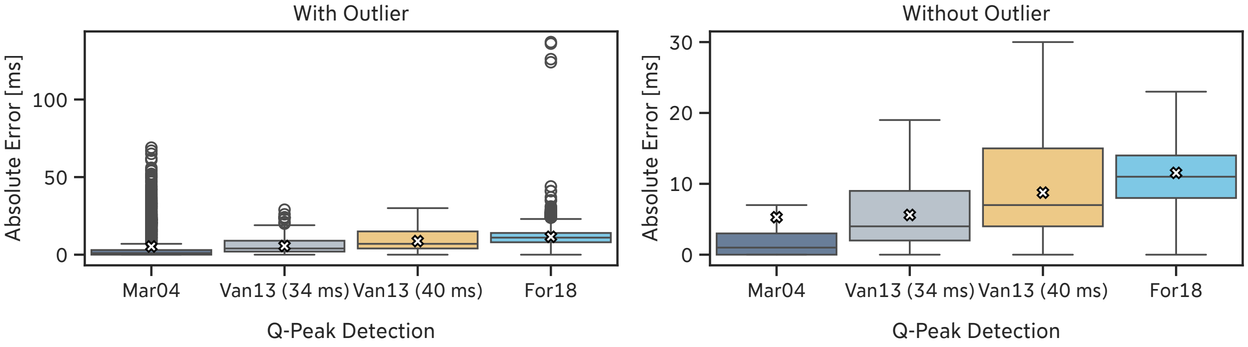

Figure S5: Absolute error of selected Q-peak extraction algorithms on the *EmpkinS Dataset* with outliers (left) and without outliers (right). Mean values are denoted by the white cross.

Table S2: Mean Abolute Error of selected Q-peak extraction algorithms on the *EmpkinS Dataset* per participant. The values with the highest errors are highlighted in red.

	Absolute Error [ms]							
**Q-peak Algorithm**	**Mar04**		**Van13 (34 ms)**		**Van13 (40 ms)**		**For18**	
	**Mean**	**SD**	**Mean**	**SD**	**Mean**	**SD**	**Mean**	**SD**
Participant								
VP_001	19.97	10.78	3.97	3.38	6.82	5.06	12.61	4.82
VP_002	1.62	5.27	6.07	3.19	11.26	4.39	10.07	2.72
VP_003	5.25	7.57	4.86	3.56	8.59	5.44	9.02	3.64
VP_004	4.70	4.81	8.45	2.42	4.86	4.92	22.16	9.35
VP_005	9.83	11.64	5.86	4.50	9.19	6.59	10.73	4.72
VP_020	6.77	10.17	3.80	2.56	5.46	4.40	13.33	10.57
VP_022	4.38	11.86	2.48	1.93	8.12	2.27	11.59	7.58
VP_023	9.86	11.43	6.29	1.72	2.98	5.63	13.40	4.64
VP_026	1.59	3.02	3.98	4.07	9.14	4.63	12.48	3.07
VP_027	2.50	6.98	1.06	0.76	5.14	0.98	7.36	0.76
VP_028	1.93	5.17	9.82	5.36	15.47	5.78	9.65	3.50
VP_029	5.23	10.59	4.14	2.74	9.15	3.28	11.12	3.99
VP_030	0.89	1.13	11.92	1.79	17.90	1.92	7.71	1.18
VP_031	2.68	6.48	3.79	4.05	6.52	5.53	14.56	3.86
VP_032	5.79	11.24	4.38	2.81	2.47	2.14	9.88	2.64

3.2.1 Residual Plots – Overall and Detailed

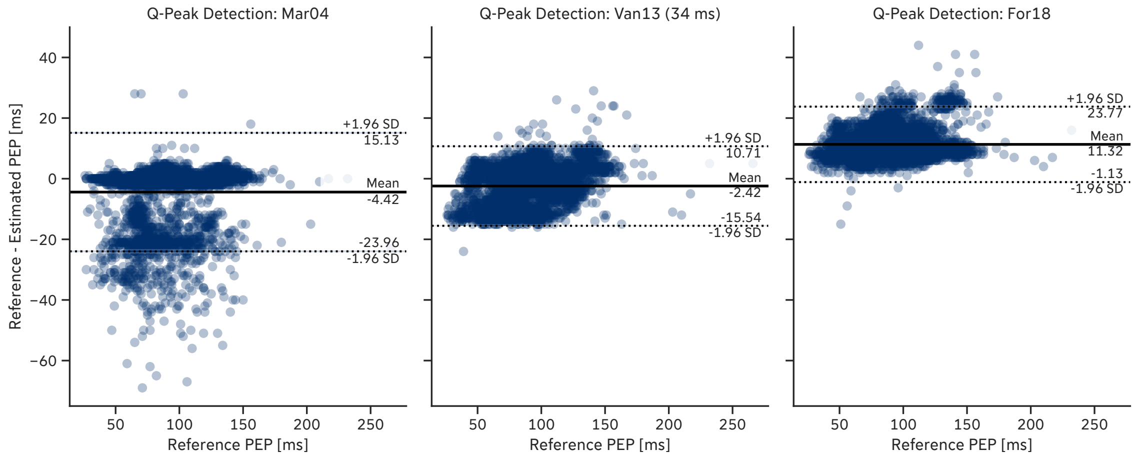

Figure S6: Residual plots of selected Q-peak extraction algorithms on the *EmpkinS Dataset*.

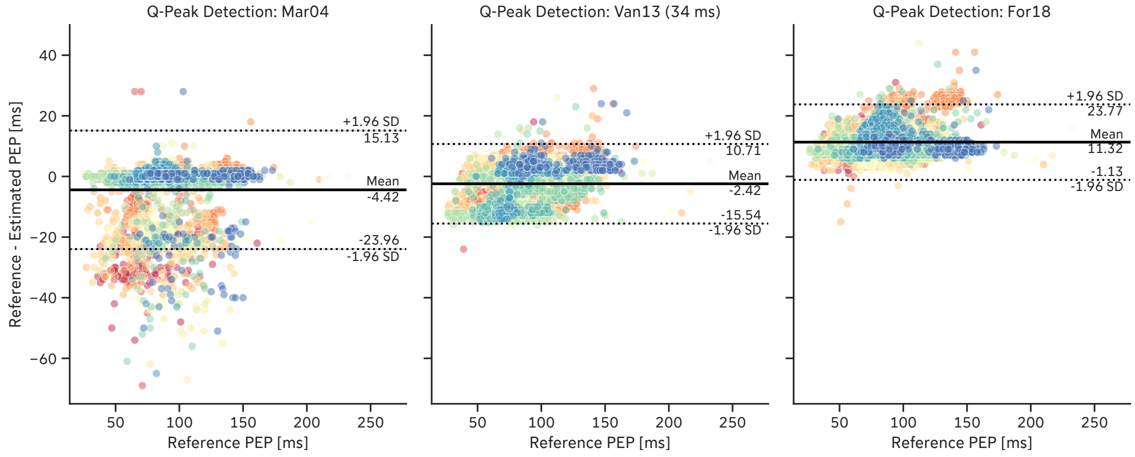

Figure S7: Residual plots of selected Q-peak extraction algorithms on the *EmpkinS Dataset*. Each participant is represented by a different color.

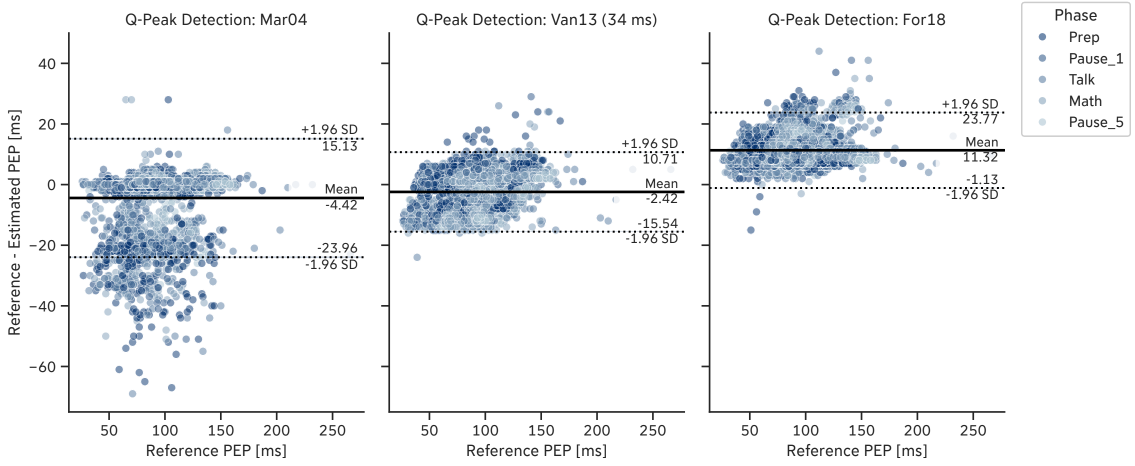

Figure S8: Residual plots of selected Q-peak extraction algorithms on the *EmpkinS Dataset*. Each phase is represented by a different color.

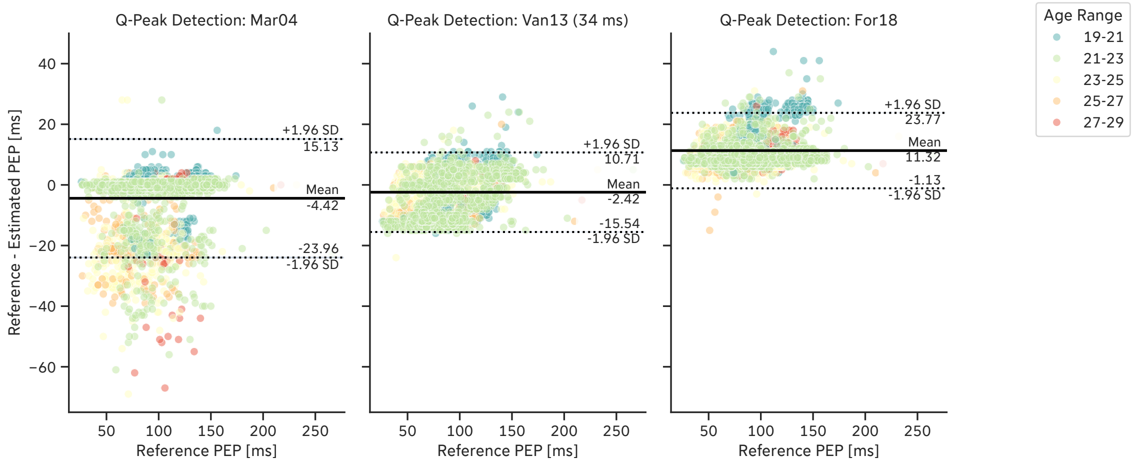

Figure S9: Residual plots of selected Q-peak extraction algorithms on the *EmpkinS Dataset*. Each age range is represented by a different color.

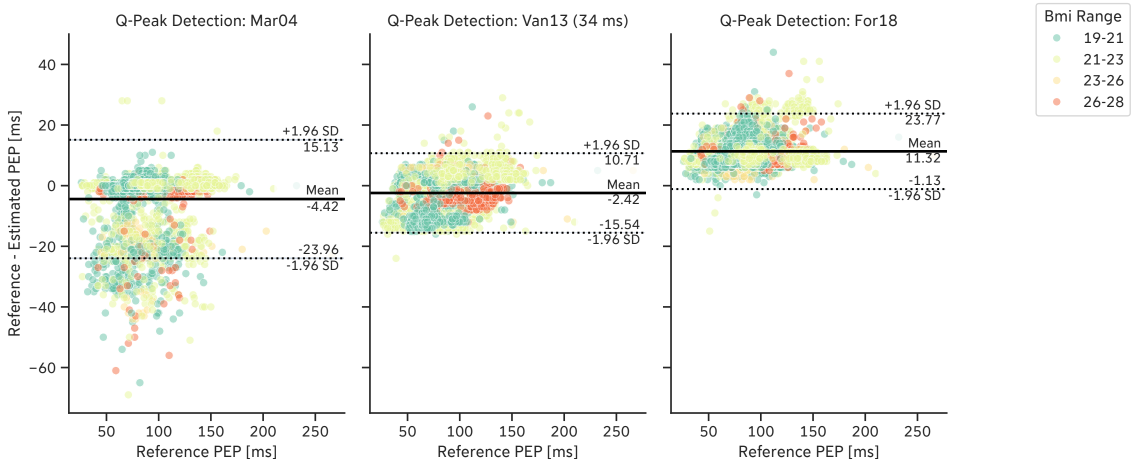

Figure S10: Residual plots of selected Q-peak extraction algorithms on the *EmpkinS Dataset*. Each BMI range is represented by a different color.

3.2.2 Effect of ECG Waveform on Q-Peak Detection Performance

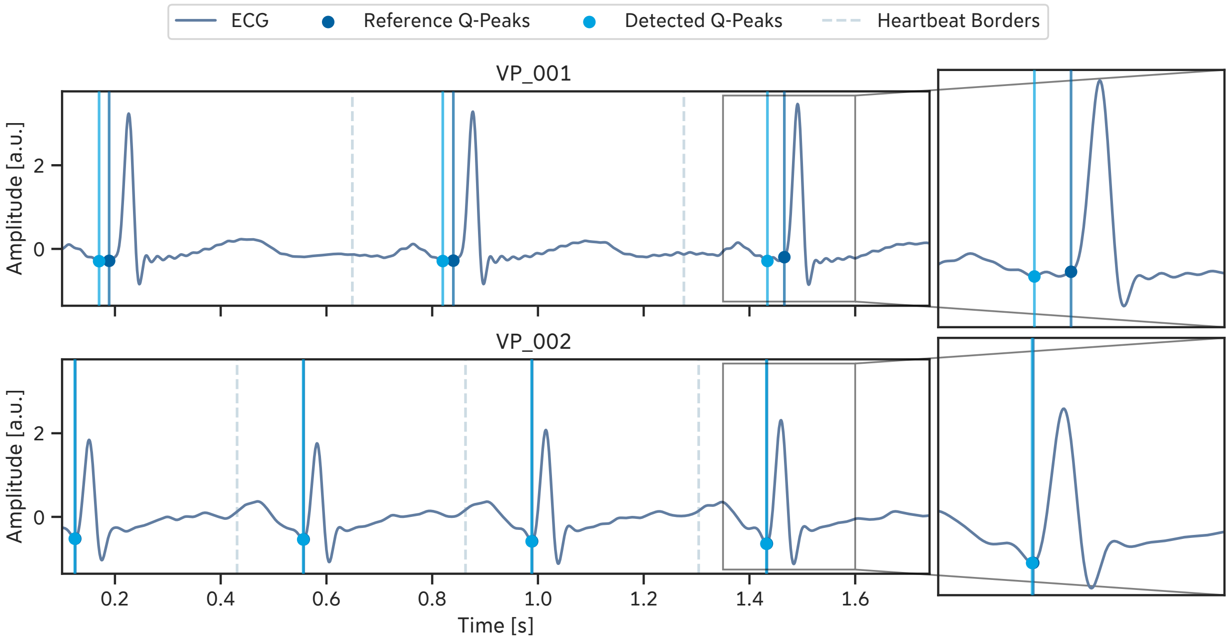

Figure S11: Examples of ECG waveforms from two participants of the *EmpkinS Dataset* and the effect of the ECG waveform on the Q-peak detection performance. The Q-peak extraction was performed using the *Mar04* algorithm [2].

3.2.3 Effect of Heart Rate on Q-Peak Detection Error

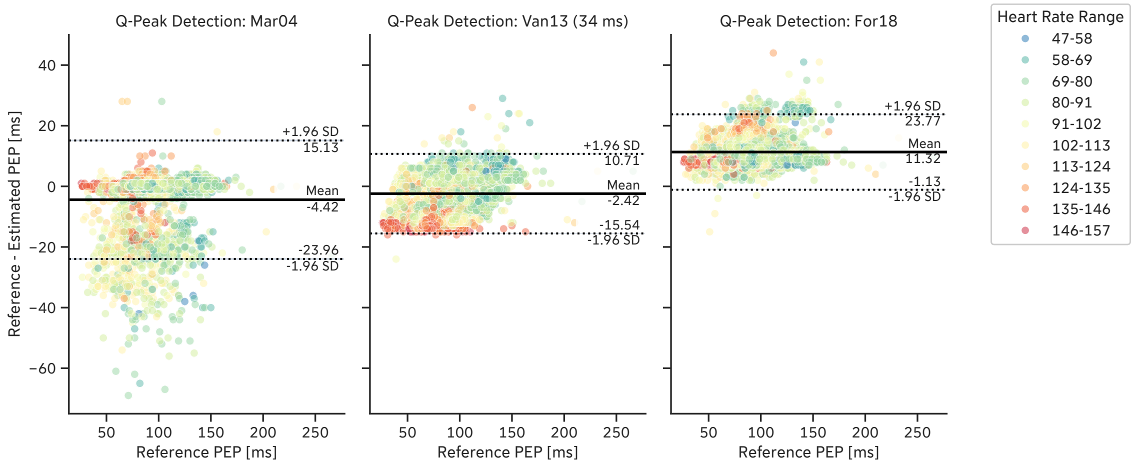

Figure S12: Residual plots of selected Q-peak extraction algorithms on the *EmpkinS Dataset*. Each heart rate bin is represented by a different color.

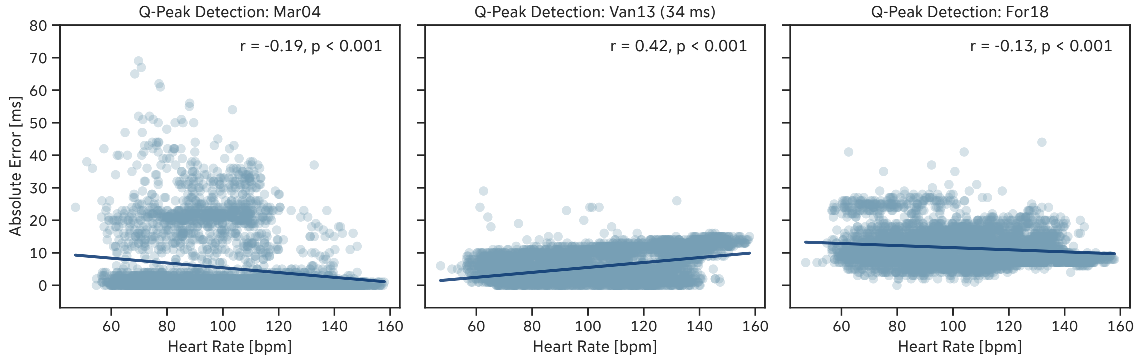

Figure S13: Regression plot between heart rate and absolute error of selected Q-peak extraction algorithms on the *EmpkinS Dataset*.

3.2.4 Effect of Annotations on Q-Peak Detection Error

Table S3: Error metrics of Q-peak extraction algorithms on the *EmpkinS Dataset* for different annotators and MAE difference between both annotators. MAE = Mean Absolute Error, ME = Mean Error.

	Annotator 1				Annotator 2				Annotator Difference	
	MAE [ms]		ME [ms]		MAE [ms]		ME [ms]		MAE [ms]	
	Mean	SD	Mean	SD	Mean	SD	Mean	SD	Mean	SD
**Q-peak Algorithm**										
Mar04	5.29	9.54	−4.42	9.97	5.77	9.42	−5.23	9.73	0.48	−0.12
Van13 (34 ms)	5.60	4.38	−2.42	6.69	5.05	4.15	−2.89	5.86	−0.55	−0.24
Van13 (32 ms)	5.69	3.54	−0.42	6.69	4.83	3.44	−0.89	5.86	−0.86	−0.11
Van13 (36 ms)	6.04	5.27	−4.42	6.69	5.88	4.88	−4.89	5.86	−0.17	−0.40
Van13 (38 ms)	7.20	5.84	−6.42	6.69	7.28	5.38	−6.89	5.86	0.08	−0.47
Van13 (40 ms)	8.76	6.24	−8.42	6.69	9.04	5.64	−8.89	5.86	0.28	−0.60
Van13 (42 ms)	10.57	6.45	−10.42	6.69	10.98	5.70	−10.89	5.86	0.41	−0.75
For18	11.54	5.94	11.32	6.35	10.85	3.93	10.79	4.09	−0.69	−2.01

3.2.5 Effect of Annotation Agreement on Q-Peak Detection Error

Table S4: Effect of annotation agreement on the absolute error (AE) of selected Q-peak extraction algorithm (Mar04) on the *EmpkinS Dataset*. Annotation agreements: *high*: [0 ms, 4 ms], *medium*: [5 ms, 10 ms], *low*: ≥ 11 ms

	Annotator 1		Annotator 2	
	Mean	SD	Mean	SD
Agreement Bins				
high	4.73	9.09	5.00	9.15
medium	10.86	11.60	9.63	9.87
low	8.88	11.29	13.74	8.52

3.3 B-Point Detection

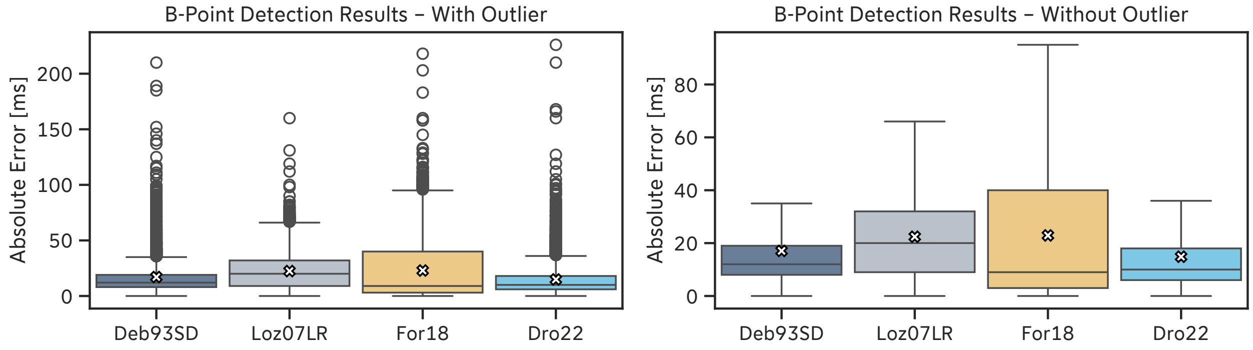

Figure S14: Absolute error of selected B-point extraction algorithms on the *EmpkinS Dataset* with outliers (left) and without outliers (right). Mean values are denoted by the white cross.

Table S5: Mean Abolute Error of selected B-point extraction algorithms on the *EmpkinS Dataset* per participant. The values with the highest errors are highlighted in red.

	Absolute Error [ms]							
**B-point Algorithm**	**Deb93SD**		**Dro22**		**For18**		**Loz07LR**	
**Outlier Correction Algorithm**	**None**		**None**		**None**		**None**	
	**Mean**	**SD**	**Mean**	**SD**	**Mean**	**SD**	**Mean**	**SD**
Participant								
VP_001	19.11	16.98	16.64	13.05	23.18	24.16	25.87	15.10
VP_002	15.14	13.72	13.69	10.16	37.97	25.17	24.06	12.52
VP_003	18.59	16.88	13.82	14.56	16.37	18.83	24.39	15.72
VP_004	15.35	13.36	16.09	15.78	24.62	24.49	15.08	14.94
VP_005	17.59	14.93	15.04	14.19	23.86	24.37	27.25	19.01
VP_020	23.41	26.07	20.67	16.34	31.02	26.60	35.91	23.28
VP_022	14.19	12.24	17.77	13.48	29.55	24.03	15.60	14.29
VP_023	15.71	17.07	15.26	13.89	10.12	21.56	27.65	12.79
VP_026	14.91	12.18	9.75	8.88	5.49	12.83	20.06	7.22
VP_027	18.91	24.78	11.36	23.07	14.38	26.18	11.25	16.45
VP_028	22.28	22.29	15.80	19.01	40.83	34.10	23.33	18.52
VP_029	18.91	17.99	20.85	17.41	39.13	29.73	23.45	17.04
VP_030	16.32	13.96	9.43	9.11	11.72	18.03	19.12	10.08
VP_031	12.69	7.13	15.03	10.30	19.81	24.30	21.45	8.99
VP_032	15.29	13.57	16.39	13.65	22.44	27.09	18.88	16.41

3.3.1 Residual Plots – Overall and Detailed

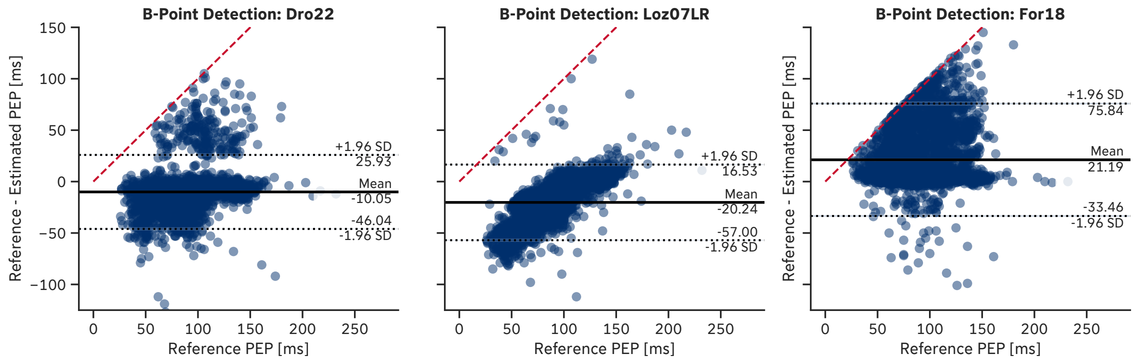

Figure S15: Residual plots of selected B-point extraction algorithms on the *EmpkinS Dataset*. The red dashed line indicates the upper estimation error limit given by the location of the reference Q-peaks.

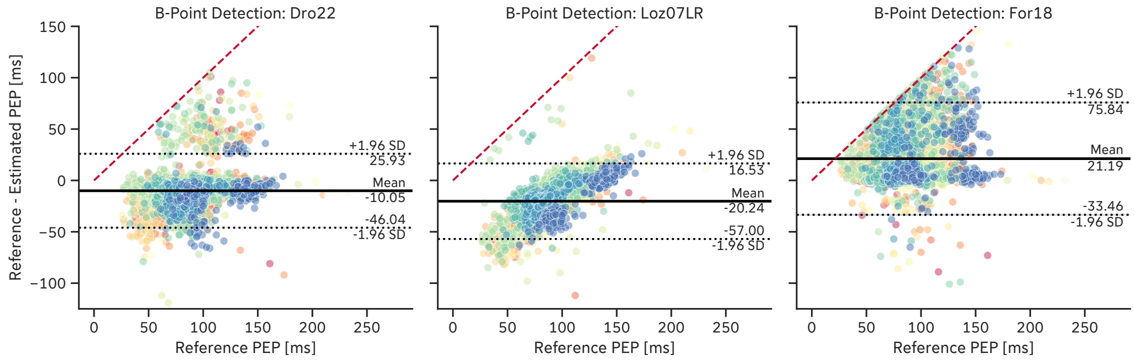

Figure S16: Residual plots of selected B-point extraction algorithms on the *EmpkinS Dataset*. Each participant is represented by a different color. The red dashed line indicates the upper estimation error limit given by the location of the reference Q-peaks.

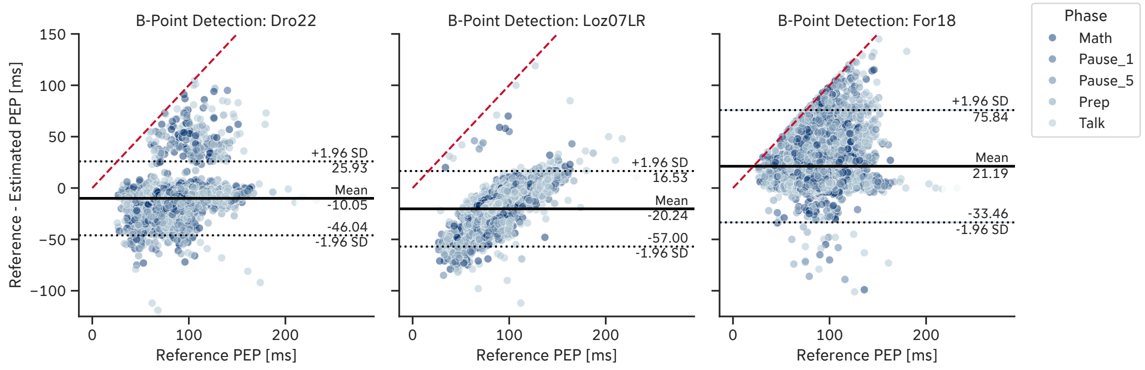

Figure S17: Residual plots of selected B-point extraction algorithms on the *EmpkinS Dataset*. Each phase is represented by a different color. The red dashed line indicates the upper estimation error limit given by the location of the reference Q-peaks.

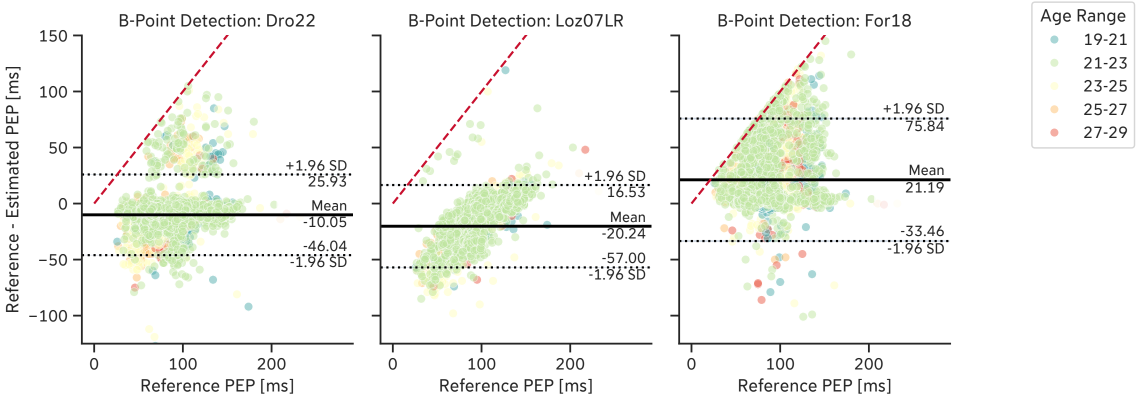

Figure S18: Residual plots of selected B-point extraction algorithms on the *EmpkinS Dataset*. Each age range is represented by a different color. The red dashed line indicates the upper estimation error limit given by the location of the reference Q-peaks.

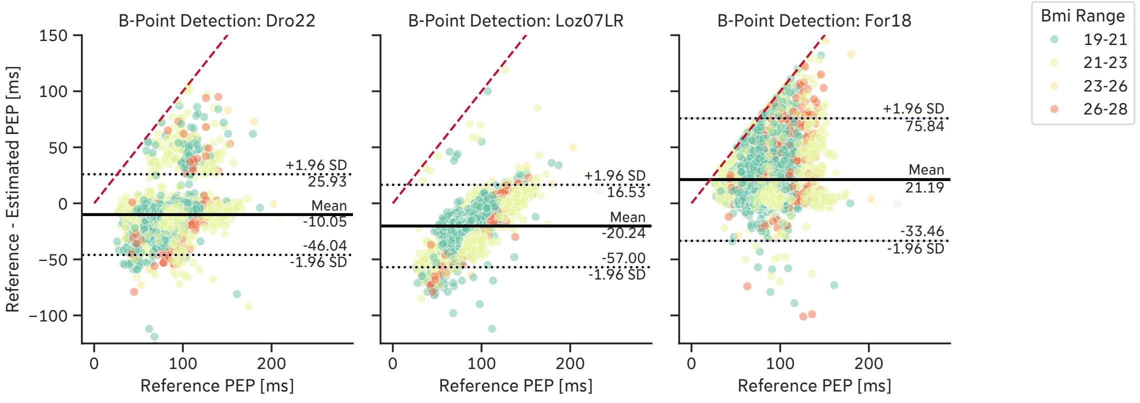

Figure S19: Residual plots of selected B-point extraction algorithms on the *EmpkinS Dataset*. Each BMI range is represented by a different color. The red dashed line indicates the upper estimation error limit given by the location of the reference Q-peaks.

3.3.2 Effect of Heart Rate on B-Point Detection Error

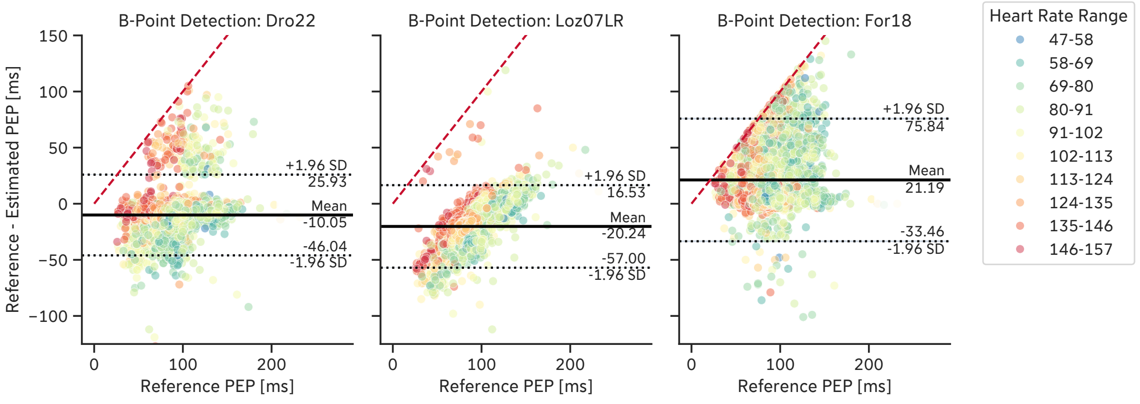

Figure S20: Residual plots of selected B-point extraction algorithms on the *EmpkinS Dataset*. Each heart rate bin is represented by a different color. The red dashed line indicates the upper estimation error limit given by the location of the reference Q-peaks.

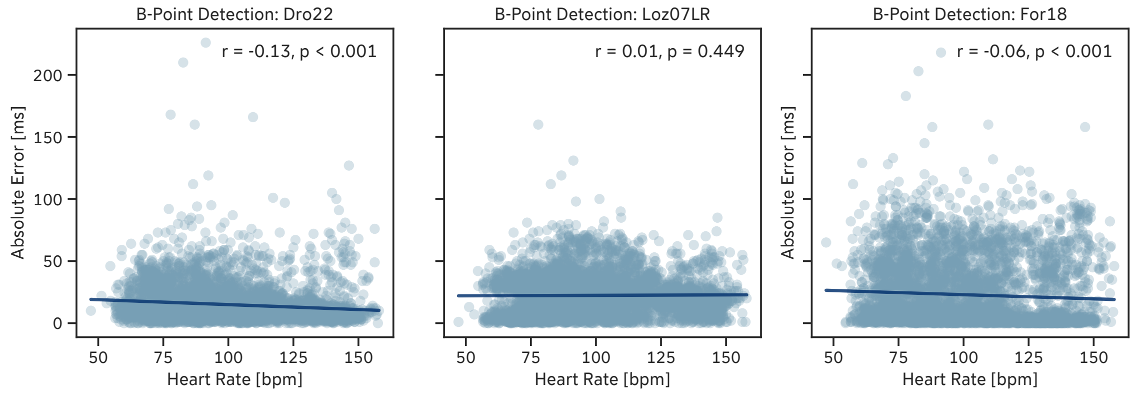

Figure S21: Regression plot between heart rate and absolute error of selected B-point extraction algorithms on the *EmpkinS Dataset*.

3.3.3 Effect of Outlier Correction on B-Point Detection Performance

Table S6: Effect of Outlier Correction algorithms on the B-point extraction algorithms for the *EmpkinS Dataset*. The algorithms are sorted by the Mean Absolute Error (MAE) in ascending order. Resuls highlighted in green indicate an improvement of the metric through outlier correction, red indicate no improvement.

		**MAE [ms]**		**ME [ms]**		**MARE [%]**		**Valid PEPs**	**Invalid PEPs**	**Total PEPs**
		**Mean**	**SD**	**Mean**	**SD**	**Mean**	**SD**	**Total**	**Total**	**Total**
B-Point Detection	Outlier Correction									
Dro22	None	14.86	14.74	−10.05	18.36	18.84	19.93	4944	56	5000
	For18	15.23	14.73	−12.50	17.10	19.79	22.00	4955	45	5000
	LinInt	15.39	14.81	−12.84	17.07	20.02	22.21	4955	45	5000
Deb93SD	For18	16.94	17.11	1.20	24.04	22.86	28.76	4955	45	5000
	None	17.06	16.82	5.31	23.36	21.77	26.62	4589	411	5000
	LinInt	17.12	17.25	0.72	24.29	23.16	29.09	4955	45	5000
Mil22	For18	19.01	18.88	−5.81	26.16	24.02	26.87	4891	109	5000
	LinInt	19.13	19.04	−6.68	26.15	24.26	27.29	4903	97	5000
	None	19.79	20.39	−2.18	28.34	24.28	26.57	4810	190	5000
For18	LinInt	21.43	25.55	17.19	28.58	24.30	27.97	4818	182	5000
	For18	21.77	25.70	17.78	28.61	24.62	27.93	4816	184	5000
	None	22.91	26.49	21.19	27.88	25.75	28.07	4701	299	5000
Loz07QR	None	21.64	15.04	−16.94	20.19	30.50	30.60	4961	39	5000
	For18	22.51	16.37	−18.49	20.81	31.61	31.85	4961	39	5000
	LinInt	22.59	16.51	−18.61	20.90	31.73	32.02	4961	39	5000
Loz07LR	None	22.41	16.11	−20.24	18.76	31.87	31.86	4971	29	5000
	For18	23.59	18.22	−21.65	20.49	33.36	34.46	4971	29	5000
	LinInt	23.68	18.33	−21.75	20.58	33.47	34.58	4971	29	5000
Arb17IC	For18	23.30	15.28	−12.97	24.66	29.74	23.46	4938	62	5000
	LinInt	23.45	15.28	−13.39	24.58	29.92	23.50	4938	62	5000
	None	24.35	15.96	−9.93	27.37	30.57	22.79	4864	136	5000
She90	For18	23.31	15.31	−14.31	23.94	29.77	24.12	4956	44	5000
	LinInt	23.52	15.50	−14.78	23.98	30.11	24.64	4956	44	5000
	None	23.79	14.68	−11.60	25.44	29.97	22.63	4709	291	5000
Arb17TD	None	23.72	18.36	−19.75	22.57	30.48	29.36	4607	393	5000
	For18	25.04	18.72	−22.73	21.47	32.76	31.17	4830	170	5000
	LinInt	25.23	18.77	−23.36	21.05	33.11	31.43	4840	160	5000
Ste85	LinInt	24.19	28.93	20.26	31.80	26.98	31.02	4429	571	5000
	For18	24.47	28.99	21.00	31.59	27.25	31.05	4425	575	5000
	None	26.25	30.91	24.14	32.58	28.82	32.52	4208	792	5000
Pal21	None	37.48	25.32	−14.34	42.90	46.48	34.15	3542	1458	5000
	LinInt	37.76	24.76	−16.26	42.13	46.91	34.50	3893	1107	5000
	For18	38.01	25.01	−15.56	42.76	47.13	34.34	3858	1142	5000
Arb17SD	LinInt	38.79	20.17	36.05	24.74	44.46	22.21	4835	165	5000
	For18	39.37	20.17	36.65	24.78	45.06	22.14	4814	186	5000
	None	41.90	21.19	39.18	25.87	47.55	22.79	4714	286	5000

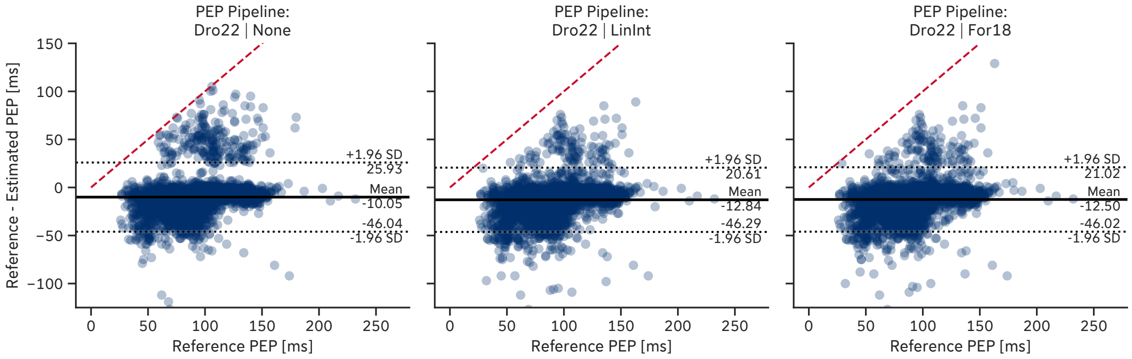

Figure S22: Effect of outlier correction (*LinInt*, *For18*) on the absolute error of the *Dro22* [3] B-point detection algorithm for on the *EmpkinS Dataset*. The red dashed line indicates the upper estimation error limit given by the location of the reference Q-peaks.

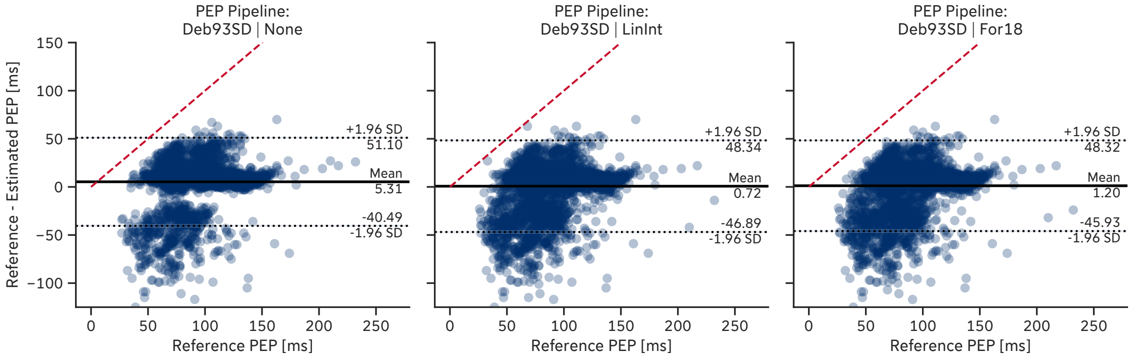

Figure S23: Effect of outlier correction (*LinInt*, *For18*) on the absolute error of the *Deb93SD* [4] B-point detection algorithm for on the *EmpkinS Dataset*. The red dashed line indicates the upper estimation error limit given by the location of the reference Q-peaks.

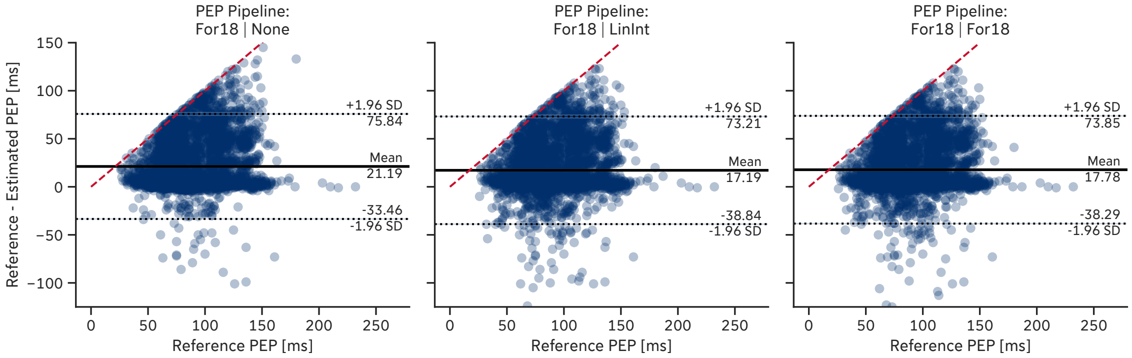

Figure S24: Effect of outlier correction (*LinInt*, *For18*) on the absolute error of the *For18* [5] B-point detection algorithm for on the *EmpkinS Dataset*. The red dashed line indicates the upper estimation error limit given by the location of the reference Q-peaks.

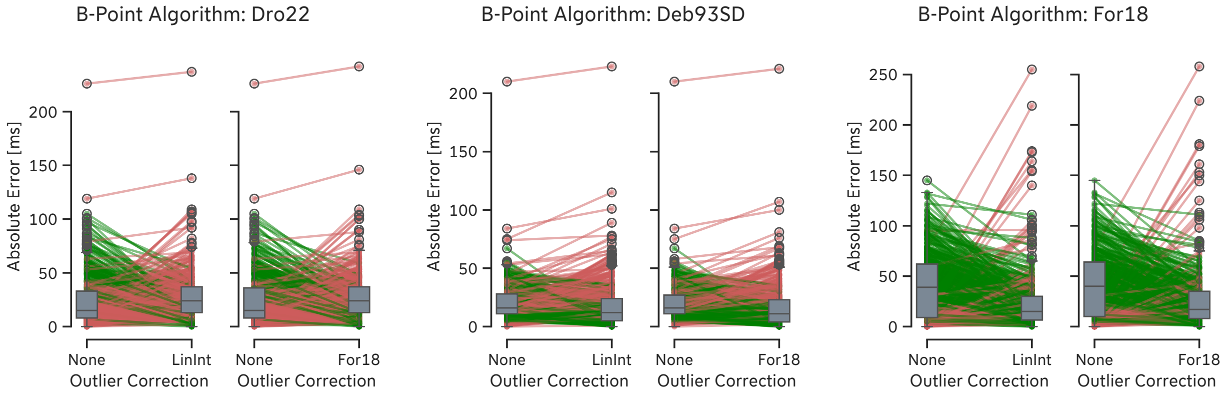

(a) *Dro22* [3] (b) *Deb93SD* [4] (c) *For18* [5]

Figure S25: Paired plots of the improvements in the absolute error due to outlier correction for the selected B-point detection algorithms on the *EmpkinS Dataset*. Green lines indicate an improvement (i.e., reduction in the absolute error) while red lines indicate a deterioration (i.e., increase in the absolute error).

3.3.4 Effect of Annotations on B-Point Detection Error

Table S7: Error metrics of B-point extraction algorithms on the *EmpkinS Dataset* for different annotators and MAE difference between both annotators. MAE = Mean Absolute Error, ME = Mean Error.

		Annotator 1				Annotator 2				Annotator Difference	
		MAE [ms]		ME [ms]		MAE [ms]		ME [ms]		MAE [ms]	
B-point Algorithm	Outlier Correction Algorithm	Mean	SD	Mean	SD	Mean	SD	Mean	SD	Mean	SD
Dro22	None	14.86	14.74	−10.05	18.36	17.43	15.08	−15.55	17.02	2.58	0.34
	For18	15.23	14.73	−12.50	17.10	18.46	15.85	−17.66	16.74	3.23	1.12
	LinInt	15.39	14.81	−12.84	17.07	18.66	16.04	−17.93	16.85	3.27	1.23
Deb93SD	For18	16.94	17.11	1.20	24.04	19.06	18.97	−3.73	26.64	2.13	1.87
	None	17.06	16.82	5.31	23.36	18.68	18.04	0.75	25.96	1.62	1.22
	LinInt	17.12	17.25	0.72	24.29	19.26	19.14	−4.18	26.83	2.14	1.89
Mil22	For18	19.01	18.88	−5.81	26.16	19.86	19.23	−11.01	25.36	0.85	0.35
	LinInt	19.13	19.04	−6.68	26.15	20.19	19.49	−11.82	25.46	1.07	0.45
	None	19.79	20.39	−2.18	28.34	19.88	20.41	−7.39	27.52	0.09	0.02
For18	LinInt	21.43	25.55	17.19	28.58	17.69	23.76	12.50	26.86	−3.74	−1.79
Loz07QR	None	21.64	15.04	−16.94	20.19	26.13	18.51	−22.92	22.36	4.49	3.46
For18	For18	21.77	25.70	17.78	28.61	17.93	23.88	12.98	26.90	−3.84	−1.82
Loz07LR	None	22.41	16.11	−20.24	18.76	27.17	19.51	−25.98	21.06	4.76	3.40
Loz07QR	For18	22.51	16.37	−18.49	20.81	26.75	18.93	−23.85	22.47	4.23	2.57
	LinInt	22.59	16.51	−18.61	20.90	26.81	18.98	−23.94	22.49	4.22	2.47
For18	None	22.91	26.49	21.19	27.88	18.96	24.61	16.27	26.46	−3.95	−1.88
Arb17IC	For18	23.30	15.28	−12.97	24.66	26.05	15.63	−18.52	24.08	2.74	0.35
She90	For18	23.31	15.31	−14.31	23.94	26.20	15.62	−19.48	23.48	2.89	0.31
Arb17IC	LinInt	23.45	15.28	−13.39	24.58	26.27	15.63	−18.86	24.06	2.82	0.35
She90	LinInt	23.52	15.50	−14.78	23.98	26.52	15.84	−19.94	23.59	3.00	0.33
Loz07LR	For18	23.59	18.22	−21.65	20.49	28.04	20.72	−26.97	22.10	4.45	2.50
	LinInt	23.68	18.33	−21.75	20.58	28.10	20.70	−27.03	22.07	4.42	2.37
Arb17TD	None	23.72	18.36	−19.75	22.57	27.78	20.52	−25.65	23.12	4.06	2.16
She90	None	23.79	14.68	−11.60	25.44	26.30	15.21	−16.83	25.30	2.51	0.54
Ste85	LinInt	24.19	28.93	20.26	31.80	20.01	27.58	16.56	29.78	−4.18	−1.35
Arb17IC	None	24.35	15.96	−9.93	27.37	26.79	16.07	−15.49	27.13	2.44	0.11
Ste85	For18	24.47	28.99	21.00	31.59	20.19	27.65	17.23	29.58	−4.27	−1.34
Arb17TD	For18	25.04	18.72	−22.73	21.47	29.52	20.86	−28.08	22.76	4.47	2.14
	LinInt	25.23	18.77	−23.36	21.05	29.85	20.92	−28.68	22.50	4.62	2.15
Ste85	None	26.25	30.91	24.14	32.58	21.70	29.80	20.29	30.78	−4.54	−1.11
Pal21	None	37.48	25.32	−14.34	42.90	37.65	25.69	−17.62	42.04	0.17	0.37
	LinInt	37.76	24.76	−16.26	42.13	38.24	25.12	−19.71	41.29	0.48	0.36
	For18	38.01	25.01	−15.56	42.76	38.48	25.30	−18.93	41.98	0.47	0.29
Arb17SD	LinInt	38.79	20.17	36.05	24.74	36.25	19.25	30.56	27.39	−2.55	−0.93
	For18	39.37	20.17	36.65	24.78	36.84	19.22	31.18	27.47	−2.53	−0.95
	None	41.90	21.19	39.18	25.87	39.17	20.16	33.58	28.52	−2.73	−1.03

3.3.5 Effect of Annotation Agreement on B-Point Detection Error

Table S8: Effect of annotation agreement on the absolute error (AE) of selected B-point extraction algorithm (Dro22) on the *EmpkinS Dataset*. Annotation agreements: *high*: [0 ms, 4 ms], *medium*: [5 ms, 10 ms], *low*: $\geq$ 11 ms.

Agreement Bins	**Annotator 1**		**Annotator 2**	
	**Mean**	**SD**	**Mean**	**SD**
high	13.13	12.05	13.24	10.96
medium	15.79	16.65	17.87	13.56
low	19.24	17.28	30.66	19.70

3.4 Combined PEP Pipelines

3.4.1 Results of all PEP Pipelines

Table S9: Results of combined PEP extraction pipelines on the *EmpkinS Dataset*. The pipelines are sorted by the MAE in ascending order.

Q-Peak Detection	B-Point Detection	Outlier Correction	**MAE [ms]**	**ME [ms]**	**MARE [%]**	**Invalid PEPs**
For18	Dro22	None	10.8±15.6	1.1±19.0	13.0±18.4	68 (1.4 %)
		For18	10.9±14.7	−1.2±18.2	13.6±19.4	49 (1.0 %)
		LinInt	10.9±14.7	−1.5±18.3	13.7±19.6	48 (1.0 %)
Van13 (42 ms)	Deb93SD	None	14.5±19.6	−5.1±23.9	19.2±32.0	411 (8.2 %)
Van13 (40 ms)	Deb93SD	None	14.5±19.1	−3.1±23.9	19.1±31.1	411 (8.2 %)
Van13 (38 ms)	Deb93SD	None	14.9±18.7	−1.1±23.9	19.2±30.2	411 (8.2 %)
Van13 (36 ms)	Deb93SD	None	15.4±18.2	0.9±23.9	19.7±29.3	411 (8.2 %)
Van13 (32 ms)	Dro22	None	15.6±14.8	−10.4±18.9	20.5±21.0	51 (1.0 %)
Van13 (38 ms)	Deb93SD	For18	15.8±19.3	−5.2±24.4	22.0±33.5	45 (0.9 %)
Van13 (40 ms)	Deb93SD	For18	15.8±19.9	−7.2±24.4	22.2±34.7	45 (0.9 %)
For18	Arb17TD	None	16.0±17.6	−8.8±22.0	21.0±26.3	417 (8.3 %)
Van13 (32 ms)	Dro22	For18	16.0±14.9	−12.9±17.7	21.4±23.2	44 (0.9 %)
Van13 (36 ms)	Deb93SD	For18	16.0±18.6	−3.2±24.4	21.9±32.4	45 (0.9 %)
Van13 (42 ms)	Deb93SD	For18	16.1±20.5	−9.2±24.4	22.8±35.9	45 (0.9 %)
Van13 (38 ms)	Deb93SD	LinInt	16.1±19.5	−5.7±24.7	22.4±34.0	45 (0.9 %)
Van13 (40 ms)	Deb93SD	LinInt	16.2±20.1	−7.7±24.7	22.7±35.1	45 (0.9 %)
Van13 (34 ms)	Deb93SD	None	16.2±17.8	2.9±23.9	20.3±28.4	411 (8.2 %)
Van13 (32 ms)	Dro22	LinInt	16.2±15.0	−13.2±17.7	21.7±23.5	44 (0.9 %)
Van13 (36 ms)	Deb93SD	LinInt	16.3±18.9	−3.7±24.7	22.4±32.8	45 (0.9 %)
For18	Loz07LR	None	16.4±13.9	−9.0±19.6	22.8±26.7	35 (0.7 %)
Van13 (34 ms)	Deb93SD	For18	16.4±18.0	−1.2±24.4	22.1±31.2	45 (0.9 %)
Van13 (42 ms)	Deb93SD	LinInt	16.5±20.8	−9.7±24.7	23.3±36.3	44 (0.9 %)
Van13 (34 ms)	Deb93SD	LinInt	16.7±18.2	−1.7±24.7	22.5±31.6	45 (0.9 %)
For18	Loz07QR	None	16.8±13.7	−5.7±20.9	22.4±25.2	46 (0.9 %)
Van13 (32 ms)	Deb93SD	For18	17.1±17.4	0.8±24.4	22.6±30.0	45 (0.9 %)
For18	Arb17TD	For18	17.1±18.1	−11.6±22.0	22.9±28.2	183 (3.7 %)
Van13 (32 ms)	Deb93SD	None	17.2±17.3	4.9±23.9	21.3±27.4	411 (8.2 %)
For18	Arb17TD	LinInt	17.2±18.1	−12.1±21.8	23.1±28.4	171 (3.4 %)
Van13 (34 ms)	Dro22	None	17.2±14.6	−12.3±18.9	22.5±21.5	50 (1.0 %)
Van13 (32 ms)	Deb93SD	LinInt	17.3±17.6	0.3±24.6	23.0±30.3	46 (0.9 %)
For18	Loz07QR	For18	17.3±14.9	−7.2±21.7	23.2±26.8	46 (0.9 %)
	Loz07LR	For18	17.4±16.2	−10.4±21.3	24.1±29.7	35 (0.7 %)
	Loz07QR	LinInt	17.4±15.1	−7.4±21.8	23.3±26.9	46 (0.9 %)
	Loz07LR	LinInt	17.4±16.3	−10.5±21.4	24.2±29.9	35 (0.7 %)
Van13 (34 ms)	Dro22	For18	17.6±14.9	−14.9±17.7	23.5±23.9	44 (0.9 %)
Mar04	Deb93SD	None	17.8±18.5	1.2±25.7	22.7±29.9	545 (10.9 %)
Van13 (34 ms)	Dro22	LinInt	17.8±15.1	−15.2±17.7	23.8±24.1	44 (0.9 %)
For18	She90	For18	17.9±17.3	−3.0±24.7	22.2±22.7	47 (0.9 %)
		LinInt	17.9±17.3	−3.5±24.7	22.3±23.2	48 (1.0 %)
	Arb17IC	LinInt	18.0±17.6	−2.1±25.1	22.2±22.6	68 (1.4 %)
		For18	18.1±17.6	−1.7±25.1	22.3±22.5	68 (1.4 %)
Mar04	Deb93SD	For18	18.5±19.4	−3.4±26.6	25.1±33.4	190 (3.8 %)
		LinInt	18.7±19.7	−3.9±26.9	25.5±33.7	190 (3.8 %)
Van13 (36 ms)	Dro22	None	18.8±14.4	−14.3±18.9	24.6±21.9	50 (1.0 %)
For18	She90	None	18.9±18.0	−0.2±26.1	22.9±22.2	294 (5.9 %)
Mar04	Dro22	None	18.9±17.6	−14.5±21.5	24.3±26.1	200 (4.0 %)
Van13 (36 ms)	Dro22	For18	19.3±15.0	−16.9±17.7	25.7±24.5	44 (0.9 %)
Mar04	Dro22	For18	19.4±18.1	−17.0±20.4	25.4±28.2	189 (3.8 %)
For18	Arb17IC	None	19.4±19.3	1.2±27.4	23.5±22.9	153 (3.1 %)
Van13 (36 ms)	Dro22	LinInt	19.5±15.1	−17.2±17.7	26.0±24.7	43 (0.9 %)
Mar04	Dro22	LinInt	19.6±18.2	−17.3±20.4	25.7±28.4	189 (3.8 %)

Continued on next page

Table S9: Results of combined PEP extraction pipelines on the *EmpkinS Dataset*. The pipelines are sorted by the MAE in ascending order.

Q-Peak Detection	B-Point Detection	Outlier Correction	**MAE [ms]**	**ME [ms]**	**MARE [%]**	**Invalid PEPs**
Van13 (38 ms)	Dro22	None	20.5±14.3	−16.3±18.9	26.8±22.3	50 (1.0 %)
		For18	21.1±15.0	−18.9±17.7	27.9±25.1	44 (0.9 %)
Van13 (40 ms)	For18	LinInt	21.2±23.6	10.1±30.1	24.6±26.2	80 (1.6 %)
Van13 (42 ms)	For18	LinInt	21.3±23.2	8.3±30.4	24.8±25.9	66 (1.3 %)
Van13 (38 ms)	Dro22	LinInt	21.3±15.2	−19.2±17.8	28.2±25.3	42 (0.8 %)
Van13 (42 ms)	For18	For18	21.4±23.2	8.9±30.3	24.8±25.6	66 (1.3 %)
Van13 (40 ms)	For18	For18	21.4±23.6	10.7±30.1	24.7±26.0	78 (1.6 %)
Van13 (38 ms)	For18	LinInt	21.4±24.0	11.9±29.9	24.7±26.4	96 (1.9 %)
		For18	21.6±24.1	12.5±29.9	24.8±26.2	96 (1.9 %)
Mar04	For18	LinInt	21.7±24.9	12.9±30.4	24.8±27.8	297 (5.9 %)
Van13 (36 ms)	For18	LinInt	21.9±24.4	13.7±29.7	25.1±26.6	106 (2.1 %)
Mar04	For18	For18	22.0±25.0	13.5±30.5	25.1±27.7	299 (6.0 %)
Van13 (36 ms)	For18	For18	22.1±24.4	14.3±29.7	25.3±26.4	107 (2.1 %)
Van13 (40 ms)	Dro22	None	22.3±14.1	−18.3±18.9	29.0±22.8	50 (1.0 %)
Van13 (34 ms)	For18	LinInt	22.5±24.5	15.4±29.4	25.6±26.6	128 (2.6 %)
Van13 (42 ms)	For18	None	22.5±23.6	12.9±30.0	25.8±24.7	137 (2.7 %)
Van13 (40 ms)	For18	None	22.6±24.2	14.6±29.7	25.8±25.3	156 (3.1 %)
Van13 (34 ms)	For18	For18	22.7±24.5	16.0±29.4	25.8±26.5	130 (2.6 %)
Van13 (38 ms)	For18	None	22.8±24.6	16.2±29.3	25.9±25.6	185 (3.7 %)
Van13 (40 ms)	Dro22	For18	22.9±15.1	−20.9±17.7	30.2±25.7	43 (0.9 %)
Van13 (32 ms)	Loz07QR	None	22.9±15.3	−17.3±21.5	32.6±32.4	37 (0.7 %)
Mar04	For18	None	23.0±25.4	17.1±29.7	26.0±26.9	405 (8.1 %)
Van13 (40 ms)	Dro22	LinInt	23.1±15.2	−21.2±17.8	30.5±25.9	42 (0.8 %)
Van13 (32 ms)	For18	LinInt	23.3±24.7	17.2±29.3	26.5±26.7	144 (2.9 %)
Van13 (36 ms)	For18	None	23.3±25.0	18.0±29.1	26.5±26.1	200 (4.0 %)
Van13 (32 ms)	Loz07LR	None	23.5±16.0	−20.7±19.5	33.8±33.5	30 (0.6 %)
	Arb17IC	For18	23.5±15.3	−13.3±24.8	30.7±25.0	58 (1.2 %)
	For18	For18	23.6±24.8	17.8±29.2	26.7±26.6	145 (2.9 %)
	She90	For18	23.6±15.6	−14.7±24.2	30.8±25.9	41 (0.8 %)
For18	Deb93SD	LinInt	23.7±14.6	12.0±25.1	29.7±23.3	50 (1.0 %)
Van13 (32 ms)	Arb17IC	LinInt	23.7±15.4	−13.7±24.7	30.9±25.2	57 (1.1 %)
For18	Deb93SD	For18	23.7±14.5	12.5±24.8	29.7±23.0	48 (1.0 %)
Van13 (32 ms)	Loz07QR	For18	23.8±16.6	−18.9±22.0	33.7±33.7	37 (0.7 %)
	She90	LinInt	23.8±15.8	−15.1±24.2	31.2±26.4	41 (0.8 %)
	Loz07QR	LinInt	23.9±16.7	−19.0±22.2	33.8±33.8	37 (0.7 %)
Van13 (34 ms)	For18	None	24.0±25.2	19.6±28.7	27.1±26.3	228 (4.6 %)
Van13 (32 ms)	Arb17TD	None	24.0±18.4	−19.8±22.9	31.6±30.3	395 (7.9 %)
Van13 (42 ms)	Dro22	None	24.0±13.9	−20.3±18.9	31.2±23.3	50 (1.0 %)
Van13 (32 ms)	She90	None	24.1±14.9	−11.8±25.7	30.9±24.3	291 (5.8 %)
Van13 (34 ms)	Loz07QR	None	24.2±15.8	−19.3±21.5	34.4±33.6	37 (0.7 %)
For18	Mil22	LinInt	24.4±52.0	−1.7±57.4	24.0±26.0	127 (2.5 %)
		For18	24.5±51.7	−0.9±57.2	23.9±25.7	143 (2.9 %)
Mar04	Ste85	LinInt	24.5±27.6	15.9±33.3	27.6±30.1	651 (13.0 %)
Van13 (32 ms)	Arb17IC	None	24.6±16.0	−10.2±27.6	31.6±24.2	128 (2.6 %)
Mar04	Ste85	For18	24.7±27.7	16.7±33.1	27.8±30.0	653 (13.1 %)
Van13 (42 ms)	Dro22	For18	24.7±15.1	−22.9±17.7	32.5±26.3	43 (0.9 %)
Van13 (32 ms)	Loz07LR	For18	24.7±18.2	−22.1±21.3	35.3±36.1	30 (0.6 %)
Van13 (34 ms)	Arb17IC	For18	24.7±15.3	−15.3±24.8	32.4±25.8	57 (1.1 %)
Van13 (32 ms)	Loz07LR	LinInt	24.8±18.3	−22.2±21.4	35.4±36.3	30 (0.6 %)
	For18	None	24.9±25.4	21.3±28.5	28.0±26.5	252 (5.0 %)
Van13 (34 ms)	She90	For18	24.9±15.7	−16.7±24.2	32.5±26.7	40 (0.8 %)
	Arb17IC	LinInt	24.9±15.4	−15.7±24.7	32.6±25.9	57 (1.1 %)
Van13 (42 ms)	Dro22	LinInt	25.0±15.2	−23.2±17.8	32.8±26.5	42 (0.8 %)
Van13 (34 ms)	Loz07LR	None	25.0±16.5	−22.7±19.5	35.8±34.6	30 (0.6 %)
	Loz07QR	For18	25.1±17.1	−20.9±22.0	35.6±34.8	37 (0.7 %)

Continued on next page

Table S9: Results of combined PEP extraction pipelines on the *EmpkinS Dataset*. The pipelines are sorted by the MAE in ascending order.

Q-Peak Detection	B-Point Detection	Outlier Correction	**MAE [ms]**	**ME [ms]**	**MARE [%]**	**Invalid PEPs**
	She90	LinInt	25.1±15.9	−17.1±24.3	32.9±27.2	40 (0.8 %)
	Loz07QR	LinInt	25.2±17.2	−21.0±22.2	35.7±34.9	37 (0.7 %)
	She90	None	25.2±14.7	−13.8±25.7	32.5±25.0	291 (5.8 %)
Van13 (32 ms)	Arb17TD	For18	25.4±18.8	−22.9±21.8	34.0±32.2	167 (3.3 %)
Mar04	Loz07QR	None	25.5±19.0	−21.4±23.4	35.9±37.2	184 (3.7 %)
Van13 (36 ms)	Loz07QR	None	25.5±16.3	−21.3±21.5	36.3±34.7	37 (0.7 %)
Van13 (32 ms)	Arb17TD	LinInt	25.5±18.9	−23.6±21.2	34.3±32.5	161 (3.2 %)
For18	Mil22	None	25.6±50.6	2.9±56.7	25.1±25.5	240 (4.8 %)
Van13 (32 ms)	Mil22	For18	25.7±52.9	−11.8±57.6	26.9±29.2	93 (1.9 %)
For18	Deb93SD	None	25.7±14.4	16.7±24.3	30.9±21.8	412 (8.2 %)
Van13 (34 ms)	Arb17IC	None	25.7±15.7	−12.1±27.6	33.1±24.8	126 (2.5 %)
	Arb17TD	None	25.8±18.4	−21.7±23.1	33.8±30.9	390 (7.8 %)
Van13 (32 ms)	Mil22	LinInt	25.8±53.2	−12.7±57.8	27.1±29.7	84 (1.7 %)
Van13 (36 ms)	Arb17IC	For18	26.0±15.4	−17.3±24.8	34.1±26.6	57 (1.1 %)
	Ste85	LinInt	26.2±28.3	18.0±34.0	29.7±30.5	434 (8.7 %)
	Arb17IC	LinInt	26.2±15.5	−17.7±24.7	34.3±26.7	57 (1.1 %)
	She90	For18	26.2±15.8	−18.7±24.2	34.3±27.5	40 (0.8 %)
Van13 (34 ms)	Loz07LR	For18	26.2±18.6	−24.1±21.3	37.3±37.1	30 (0.6 %)
Van13 (32 ms)	Mil22	None	26.2±51.6	−7.6±57.4	27.2±28.6	168 (3.4 %)
Van13 (38 ms)	Ste85	LinInt	26.2±28.2	16.8±34.7	29.9±30.6	380 (7.6 %)
Van13 (34 ms)	Loz07LR	LinInt	26.3±18.8	−24.2±21.4	37.4±37.3	30 (0.6 %)
Mar04	Ste85	None	26.4±29.4	20.1±34.0	29.2±31.0	851 (17.0 %)
Van13 (36 ms)	She90	None	26.4±14.7	−15.8±25.7	34.2±25.8	291 (5.8 %)
		LinInt	26.4±16.0	−19.1±24.3	34.7±28.0	40 (0.8 %)
	Loz07QR	For18	26.5±17.6	−22.9±22.0	37.5±35.9	37 (0.7 %)
Mar04	Loz07QR	For18	26.5±20.2	−23.0±24.1	37.1±38.4	184 (3.7 %)
Van13 (34 ms)	Mil22	For18	26.5±53.0	−13.6±57.7	28.0±30.0	83 (1.7 %)
	Ste85	LinInt	26.5±28.4	19.5±33.6	29.9±30.5	474 (9.5 %)
Van13 (40 ms)	Ste85	LinInt	26.5±28.0	15.6±35.2	30.4±30.7	324 (6.5 %)
Van13 (36 ms)	Ste85	For18	26.5±28.5	19.0±33.9	30.0±30.6	422 (8.4 %)
Van13 (38 ms)	Ste85	For18	26.5±28.4	17.7±34.6	30.1±30.6	371 (7.4 %)
Van13 (36 ms)	Loz07QR	LinInt	26.5±17.7	−23.0±22.2	37.6±36.0	37 (0.7 %)
Mar04	Loz07QR	LinInt	26.5±20.4	−23.1±24.2	37.2±38.5	184 (3.7 %)
	Arb17IC	For18	26.6±18.3	−17.4±27.1	34.4±30.0	204 (4.1 %)
Van13 (36 ms)	Loz07LR	None	26.6±17.0	−24.6±19.8	38.0±35.6	28 (0.6 %)
Van13 (34 ms)	Mil22	LinInt	26.6±53.4	−14.6±57.8	28.3±30.4	75 (1.5 %)
Mar04	She90	For18	26.7±18.5	−18.8±26.4	34.7±30.8	189 (3.8 %)
	Loz07LR	None	26.7±19.9	−24.6±22.4	37.6±38.2	171 (3.4 %)
Van13 (40 ms)	Ste85	For18	26.7±28.1	16.5±35.1	30.5±30.6	316 (6.3 %)
Mar04	Arb17IC	LinInt	26.8±18.3	−17.8±27.1	34.7±30.1	204 (4.1 %)
Van13 (34 ms)	Ste85	For18	26.8±28.6	20.4±33.4	30.2±30.5	469 (9.4 %)
Van13 (36 ms)	Arb17IC	None	26.9±15.5	−14.1±27.7	34.7±25.5	124 (2.5 %)
Mar04	She90	None	26.9±17.5	−16.0±27.8	34.6±29.3	426 (8.5 %)
Van13 (32 ms)	Ste85	LinInt	26.9±28.5	20.8±33.2	30.1±30.2	525 (10.5 %)
Van13 (34 ms)	Mil22	None	26.9±51.7	−9.3±57.5	28.2±29.2	155 (3.1 %)
Van13 (38 ms)	Loz07QR	None	26.9±16.7	−23.3±21.5	38.3±35.8	37 (0.7 %)
Mar04	She90	LinInt	26.9±18.7	−19.3±26.5	35.0±31.2	189 (3.8 %)
Van13 (42 ms)	Ste85	LinInt	27.0±27.7	14.4±35.9	31.1±30.5	272 (5.4 %)
		For18	27.1±27.8	15.2±35.7	31.1±30.4	265 (5.3 %)
Van13 (34 ms)	Arb17TD	For18	27.2±18.9	−24.8±21.9	36.2±32.8	165 (3.3 %)
Van13 (32 ms)	Ste85	For18	27.3±28.6	21.8±33.0	30.5±30.3	517 (10.3 %)
Van13 (38 ms)	Arb17IC	For18	27.3±15.5	−19.3±24.8	35.9±27.5	56 (1.1 %)
Van13 (34 ms)	Arb17TD	LinInt	27.3±19.0	−25.5±21.4	36.5±33.2	158 (3.2 %)
Mar04	Arb17IC	None	27.4±18.3	−14.1±29.7	35.0±29.0	274 (5.5 %)
Van13 (36 ms)	Mil22	For18	27.4±53.1	−15.5±57.7	29.4±30.7	74 (1.5 %)

Continued on next page

Table S9: Results of combined PEP extraction pipelines on the *EmpkinS Dataset*. The pipelines are sorted by the MAE in ascending order.

Q-Peak Detection	B-Point Detection	Outlier Correction	**MAE [ms]**	**ME [ms]**	**MARE [%]**	**Invalid PEPs**
Van13 (38 ms)	Arb17IC	LinInt	27.5±15.6	−19.7±24.7	36.1±27.5	56 (1.1 %)
	She90	For18	27.5±16.0	−20.7±24.2	36.1±28.4	40 (0.8 %)
		None	27.6±14.7	−17.8±25.7	35.8±26.7	291 (5.8 %)
Van13 (36 ms)	Arb17TD	None	27.6±18.3	−23.6±23.3	36.1±31.4	385 (7.7 %)
	Mil22	LinInt	27.6±53.5	−16.4±57.9	29.7±31.1	68 (1.4 %)
Van13 (38 ms)	She90	LinInt	27.8±16.1	−21.1±24.3	36.5±28.9	40 (0.8 %)
Van13 (36 ms)	Loz07LR	For18	27.8±19.2	−26.0±21.5	39.5±38.1	28 (0.6 %)
	Mil22	None	27.9±52.1	−11.3±58.0	29.4±29.8	143 (2.9 %)
Mar04	Arb17TD	None	27.9±21.4	−23.8±25.8	36.1±35.3	510 (10.2 %)
Van13 (38 ms)	Loz07QR	For18	27.9±18.0	−24.9±22.0	39.6±36.9	37 (0.7 %)
Van13 (36 ms)	Loz07LR	LinInt	27.9±19.3	−26.1±21.6	39.6±38.2	28 (0.6 %)
Mar04	Loz07LR	For18	27.9±21.8	−26.1±24.0	39.2±40.6	171 (3.4 %)
Van13 (38 ms)	Loz07QR	LinInt	28.0±18.2	−25.0±22.2	39.7±37.1	37 (0.7 %)
Mar04	Loz07LR	LinInt	28.0±21.9	−26.2±24.1	39.3±40.7	171 (3.4 %)
Van13 (38 ms)	Arb17IC	None	28.1±15.5	−16.0±27.9	36.4±26.3	118 (2.4 %)
Mar04	Mil22	None	28.2±51.9	−12.2±57.8	29.7±32.7	319 (6.4 %)
		For18	28.2±53.4	−16.5±58.1	30.1±34.1	244 (4.9 %)
Van13 (38 ms)	Loz07LR	None	28.2±17.4	−26.6±19.9	40.1±36.5	27 (0.5 %)
Mar04	Mil22	LinInt	28.4±53.8	−17.4±58.3	30.4±34.6	233 (4.7 %)
Van13 (36 ms)	Ste85	None	28.4±30.1	22.4±34.8	31.8±31.8	625 (12.5 %)
Van13 (38 ms)	Ste85	None	28.4±30.0	21.3±35.4	32.0±31.7	564 (11.3 %)
Van13 (40 ms)	Loz07QR	None	28.4±17.1	−25.3±21.5	40.4±36.9	37 (0.7 %)
Van13 (38 ms)	Mil22	For18	28.5±53.2	−17.3±57.8	30.9±31.3	68 (1.4 %)
Van13 (40 ms)	Ste85	None	28.7±29.6	20.2±35.9	32.5±31.7	495 (9.9 %)
	Arb17IC	For18	28.7±15.7	−21.2±24.8	37.7±28.3	55 (1.1 %)
Van13 (38 ms)	Mil22	LinInt	28.8±53.6	−18.3±58.0	31.3±31.8	62 (1.2 %)
Van13 (34 ms)	Ste85	None	28.8±30.4	23.8±34.4	32.1±31.8	676 (13.5 %)
Van13 (40 ms)	Arb17IC	LinInt	28.9±15.8	−21.7±24.8	38.0±28.4	55 (1.1 %)
Van13 (38 ms)	Mil22	None	28.9±52.2	−13.1±58.2	30.8±30.3	131 (2.6 %)
Van13 (40 ms)	She90	None	28.9±14.8	−19.8±25.7	37.6±27.6	291 (5.8 %)
		For18	28.9±16.3	−22.7±24.2	38.0±29.3	40 (0.8 %)
Van13 (36 ms)	Arb17TD	For18	29.0±18.9	−26.8±22.0	38.5±33.4	164 (3.3 %)
		LinInt	29.2±19.1	−27.5±21.4	38.8±33.8	158 (3.2 %)
Van13 (42 ms)	Ste85	None	29.2±29.4	19.2±36.7	33.2±31.4	427 (8.5 %)
Van13 (40 ms)	She90	LinInt	29.2±16.4	−23.1±24.3	38.4±29.8	40 (0.8 %)
Van13 (32 ms)	Ste85	None	29.3±30.5	25.0±34.0	32.4±31.7	735 (14.7 %)
Mar04	Arb17TD	For18	29.3±22.1	−27.1±24.7	38.4±37.5	297 (5.9 %)
Van13 (40 ms)	Arb17IC	None	29.4±15.5	−17.9±27.9	38.1±27.1	116 (2.3 %)
	Loz07QR	For18	29.5±18.4	−26.9±22.0	41.7±37.9	37 (0.7 %)
Van13 (38 ms)	Arb17TD	None	29.5±18.3	−25.5±23.6	38.4±32.0	377 (7.5 %)
	Loz07LR	For18	29.5±19.5	−28.0±21.6	41.7±39.0	27 (0.5 %)
Mar04	Arb17TD	LinInt	29.5±22.2	−27.8±24.4	38.9±37.8	289 (5.8 %)
Van13 (40 ms)	Loz07QR	LinInt	29.5±18.6	−27.0±22.2	41.8±38.1	37 (0.7 %)
Van13 (38 ms)	Loz07LR	LinInt	29.6±19.6	−28.1±21.7	41.8±39.1	27 (0.5 %)
Van13 (40 ms)	Mil22	For18	29.7±53.3	−19.3±57.9	32.4±31.9	64 (1.3 %)
		None	29.9±52.1	−14.9±58.2	32.2±30.7	124 (2.5 %)
	Loz07LR	None	30.0±17.8	−28.6±19.9	42.4±37.5	26 (0.5 %)
For18	For18	LinInt	30.0±24.5	26.9±27.9	34.2±26.1	315 (6.3 %)
Van13 (40 ms)	Mil22	LinInt	30.0±53.6	−20.2±58.0	32.9±32.4	58 (1.2 %)
Van13 (42 ms)	Loz07QR	None	30.0±17.5	−27.3±21.5	42.6±37.9	37 (0.7 %)
	Arb17IC	For18	30.1±16.0	−23.2±25.0	39.6±29.3	53 (1.1 %)
	She90	None	30.2±15.1	−21.8±25.7	39.3±28.6	291 (5.8 %)
	Arb17IC	LinInt	30.3±16.1	−23.6±24.9	39.8±29.4	53 (1.1 %)
	She90	For18	30.3±16.6	−24.7±24.2	39.9±30.3	40 (0.8 %)
For18	For18	For18	30.4±24.6	27.4±27.8	34.7±26.0	317 (6.3 %)

Continued on next page

Table S9: Results of combined PEP extraction pipelines on the *EmpkinS Dataset*. The pipelines are sorted by the MAE in ascending order.

Q-Peak Detection	B-Point Detection	Outlier Correction	**MAE [ms]**	**ME [ms]**	**MARE [%]**	**Invalid PEPs**
Van13 (42 ms)	Arb17IC	None	30.7±15.7	−19.8±28.2	39.9±28.0	110 (2.2 %)
	She90	LinInt	30.7±16.7	−25.1±24.3	40.3±30.7	40 (0.8 %)
Van13 (38 ms)	Arb17TD	For18	30.9±19.0	−28.8±22.0	40.8±34.0	162 (3.2 %)
Van13 (42 ms)	Mil22	For18	30.9±53.3	−21.2±57.9	34.1±32.5	59 (1.2 %)
	Loz07QR	For18	31.1±18.8	−28.9±22.0	43.8±38.9	37 (0.7 %)
Van13 (38 ms)	Arb17TD	LinInt	31.1±19.1	−29.5±21.5	41.1±34.4	155 (3.1 %)
Van13 (42 ms)	Loz07QR	LinInt	31.2±19.0	−29.0±22.2	44.0±39.0	37 (0.7 %)
	Mil22	None	31.2±52.5	−17.0±58.7	33.8±31.2	116 (2.3 %)
Van13 (40 ms)	Loz07LR	For18	31.2±19.8	−30.0±21.6	43.9±39.9	26 (0.5 %)
Van13 (42 ms)	Mil22	LinInt	31.3±53.7	−22.2±58.1	34.6±33.0	54 (1.1 %)
Van13 (40 ms)	Loz07LR	LinInt	31.3±19.9	−30.1±21.7	44.1±40.0	26 (0.5 %)
	Arb17TD	None	31.4±18.2	−27.4±23.8	40.7±32.5	373 (7.5 %)
Van13 (42 ms)	Arb17SD	LinInt	31.4±18.8	26.5±25.2	36.3±20.4	53 (1.1 %)
For18	For18	None	31.6±24.8	30.3±26.4	35.9±25.5	485 (9.7 %)
Van13 (42 ms)	Loz07LR	None	31.7±18.0	−30.5±20.0	44.7±38.4	24 (0.5 %)
For18	Ste85	LinInt	31.8±27.4	28.7±30.6	35.5±27.9	796 (15.9 %)
Van13 (42 ms)	Arb17SD	For18	32.1±18.8	27.3±25.2	37.0±20.4	61 (1.2 %)
For18	Ste85	For18	32.4±27.5	29.6±30.5	36.1±28.0	799 (16.0 %)
Van13 (40 ms)	Arb17TD	For18	32.8±19.0	−30.7±22.2	43.1±34.6	159 (3.2 %)
	Arb17SD	LinInt	32.8±19.2	28.5±25.2	37.7±20.5	61 (1.2 %)
	Arb17TD	LinInt	33.0±19.2	−31.4±21.7	43.5±35.0	152 (3.0 %)
Van13 (42 ms)	Loz07LR	For18	33.0±20.1	−32.0±21.7	46.3±40.8	24 (0.5 %)
		LinInt	33.1±20.2	−32.1±21.8	46.4±40.9	24 (0.5 %)
	Arb17TD	None	33.3±18.1	−29.2±24.3	43.1±33.0	363 (7.3 %)
Van13 (40 ms)	Arb17SD	For18	33.5±19.2	29.2±25.2	38.4±20.5	70 (1.4 %)
For18	Ste85	None	34.2±29.0	32.2±31.2	37.7±28.7	1062 (21.2 %)
Van13 (38 ms)	Arb17SD	LinInt	34.2±19.5	30.4±25.1	39.2±20.7	72 (1.4 %)
Van13 (42 ms)	Arb17TD	For18	34.7±19.0	−32.6±22.5	45.5±35.2	152 (3.0 %)
	Arb17SD	None	34.8±19.9	30.2±26.4	40.1±21.5	100 (2.0 %)
Van13 (38 ms)	Arb17SD	For18	34.9±19.5	31.1±25.1	39.9±20.7	80 (1.6 %)
Van13 (42 ms)	Arb17TD	LinInt	34.9±19.2	−33.3±22.0	45.9±35.7	147 (2.9 %)
Van13 (36 ms)	Arb17SD	LinInt	35.7±19.9	32.3±25.0	40.7±20.9	81 (1.6 %)
Van13 (40 ms)	Arb17SD	None	36.2±20.3	32.1±26.4	41.5±21.6	111 (2.2 %)
Van13 (36 ms)	Arb17SD	For18	36.3±19.9	33.0±25.1	41.5±21.0	91 (1.8 %)
Mar04	Arb17SD	LinInt	36.6±20.5	31.6±27.6	42.5±23.6	294 (5.9 %)
For18	Pal21	LinInt	36.7±48.7	−12.7±59.6	40.9±32.0	1247 (24.9 %)
		For18	37.1±49.0	−12.0±60.3	41.4±31.9	1281 (25.6 %)
Van13 (34 ms)	Arb17SD	LinInt	37.2±20.3	34.2±25.0	42.2±21.2	94 (1.9 %)
Mar04	Arb17SD	For18	37.2±20.5	32.2±27.7	43.1±23.6	312 (6.2 %)
For18	Pal21	None	37.3±50.6	−11.5±61.8	41.2±31.4	1598 (32.0 %)
Van13 (38 ms)	Arb17SD	None	37.6±20.6	33.9±26.3	43.0±21.8	130 (2.6 %)
Van13 (34 ms)	Arb17SD	For18	37.8±20.3	34.9±25.0	43.0±21.2	105 (2.1 %)
Van13 (32 ms)	Arb17SD	LinInt	38.7±20.7	36.1±24.9	43.8±21.5	109 (2.2 %)
Van13 (36 ms)	Arb17SD	None	39.1±21.0	35.8±26.2	44.5±22.0	144 (2.9 %)
Van13 (32 ms)	Arb17SD	For18	39.4±20.8	36.8±25.0	44.6±21.5	120 (2.4 %)
Mar04	Arb17SD	None	39.7±21.4	34.9±28.6	45.7±24.0	390 (7.8 %)
Van13 (34 ms)	Arb17SD	None	40.5±21.4	37.6±26.1	45.9±22.2	174 (3.5 %)
Van13 (32 ms)	Arb17SD	None	42.0±21.8	39.5±26.1	47.4±22.4	202 (4.0 %)
	Pal21	LinInt	43.4±49.7	−20.7±62.7	50.0±36.8	1106 (22.1 %)
		None	43.7±51.6	−19.2±64.9	49.9±36.3	1454 (29.1 %)
		For18	43.7±49.9	−20.1±63.3	50.2±36.5	1143 (22.9 %)
Van13 (34 ms)	Pal21	LinInt	44.6±50.0	−22.0±63.3	51.5±37.5	1078 (21.6 %)
		None	44.8±52.0	−20.6±65.5	51.3±37.1	1429 (28.6 %)
		For18	44.9±50.3	−21.5±63.9	51.7±37.3	1117 (22.3 %)
Mar04	Pal21	None	45.1±52.0	−22.3±65.2	51.6±40.0	1516 (30.3 %)

Continued on next page

Table S9: Results of combined PEP extraction pipelines on the *EmpkinS Dataset*. The pipelines are sorted by the MAE in ascending order.

Q-Peak Detection	B-Point Detection	Outlier Correction	**MAE [ms]**	**ME [ms]**	**MARE [%]**	**Invalid PEPs**
		LinInt	45.2±50.1	−23.8±63.2	52.2±40.5	1160 (23.2 %)
		For18	45.3±50.3	−23.2±63.7	52.2±40.3	1196 (23.9 %)
Van13 (36 ms)	Pal21	LinInt	45.8±50.2	−23.2±63.9	53.0±38.3	1050 (21.0 %)
		None	45.9±52.1	−21.7±66.0	52.7±37.9	1402 (28.0 %)
		For18	46.0±50.4	−22.7±64.3	53.1±38.0	1090 (21.8 %)
Van13 (38 ms)	Pal21	LinInt	47.0±50.2	−24.5±64.3	54.6±38.9	1024 (20.5 %)
		None	47.1±52.1	−22.9±66.4	54.2±38.5	1375 (27.5 %)
		For18	47.2±50.4	−24.0±64.8	54.7±38.7	1063 (21.3 %)
For18	Arb17SD	LinInt	48.0±22.5	46.7±25.2	54.1±23.7	297 (5.9 %)
Van13 (40 ms)	Pal21	LinInt	48.4±50.2	−25.6±64.9	56.3±39.5	992 (19.8 %)
		None	48.4±52.1	−24.0±67.0	55.8±39.1	1346 (26.9 %)
		For18	48.5±50.4	−25.2±65.3	56.3±39.3	1036 (20.7 %)
For18	Arb17SD	For18	48.6±22.5	47.2±25.3	54.7±23.7	325 (6.5 %)
Van13 (42 ms)	Pal21	LinInt	49.9±50.2	−26.5±65.6	58.0±40.0	955 (19.1 %)
		For18	49.9±50.4	−26.0±66.0	58.1±39.9	996 (19.9 %)
		None	50.0±52.1	−24.7±67.8	57.7±39.5	1305 (26.1 %)
For18	Arb17SD	None	50.7±23.5	49.3±26.3	56.4±24.1	492 (9.8 %)

3.4.2 Absolute Error

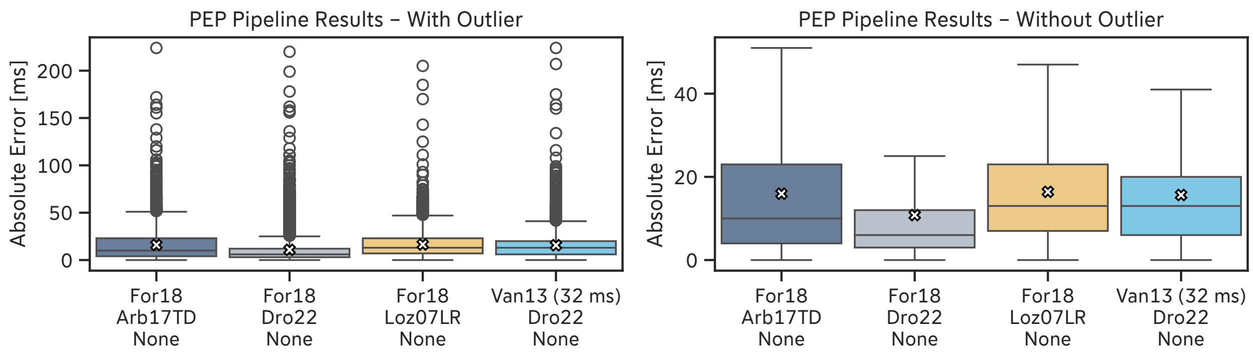

Figure S26: Absolute error of the selected PEP pipelines on the *EmpkinS Dataset* with outliers (left) and without outliers (right). Mean values are denoted by the white cross.

Table S10: Mean Abolute Error of selected PEP extraction pipelines on the *EmpkinS Dataset* per participant. The values with the highest errors are highlighted in red.

	Absolute Error [ms]							
**Q-peak Algorithm**					**For18**		**Van13 (32 ms)**	
**B-point Algorithm**	**Arb17TD**		**Dro22**		**Loz07LR**		**Dro22**	
**Outlier Correction Algorithm**	**None**		**None**		**None**		**None**	
	**Mean**	**SD**	**Mean**	**SD**	**Mean**	**SD**	**Mean**	**SD**
Participant								
VP_001	20.30	17.60	11.94	15.15	17.35	12.48	16.13	14.73
VP_002	15.02	14.44	6.93	8.31	16.12	10.14	16.85	10.67
VP_003	18.68	14.19	9.16	12.96	19.38	12.40	14.89	15.46
VP_004	18.28	16.55	18.92	19.32	19.86	14.56	15.69	15.50
VP_005	17.14	17.82	11.73	14.43	21.13	14.91	15.92	13.90
VP_020	25.35	23.71	17.02	21.20	27.06	24.38	19.02	16.78
VP_022	16.93	17.53	11.39	14.44	12.91	12.21	17.93	13.84
VP_023	15.25	17.10	8.19	14.31	15.38	14.00	10.79	14.61
VP_026	9.67	10.72	6.76	8.87	8.83	6.96	10.81	9.68
VP_027	10.27	21.33	9.11	23.00	10.84	14.64	9.17	23.35
VP_028	11.54	17.00	14.28	20.92	19.21	15.11	20.11	15.88
VP_029	25.90	24.90	15.48	17.79	19.87	11.45	21.59	17.38
VP_030	9.62	10.49	5.59	7.63	13.11	8.82	19.02	8.01
VP_031	15.12	12.37	9.26	9.35	9.71	7.51	13.67	10.39
VP_032	15.94	17.20	10.70	14.24	19.12	9.99	11.79	13.99

3.4.3 Residual Plots – Overall and Detailed

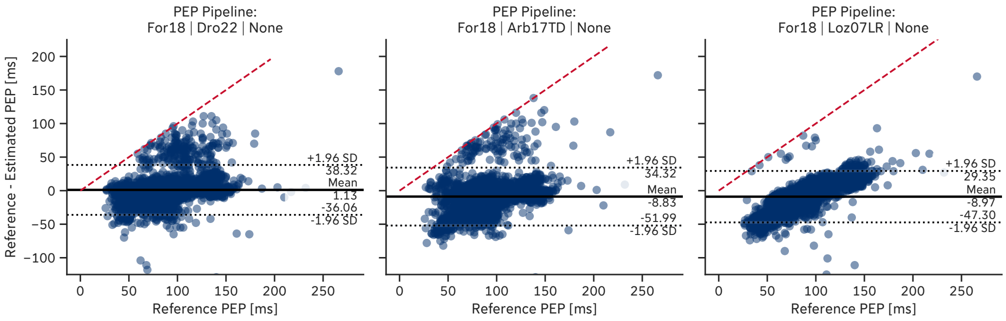

Figure S27: Residual plots of the selected PEP pipelines on the *EmpkinS Dataset*.

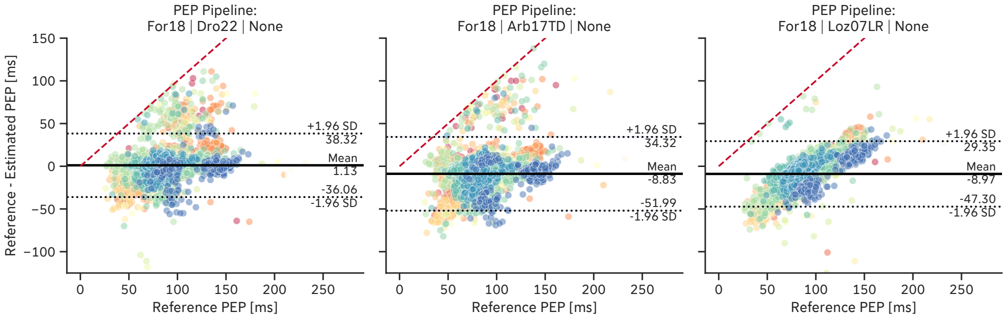

Figure S28: Residual plots of the selected PEP pipelines on the *EmpkinS Dataset*. Each participant is represented by a different color.

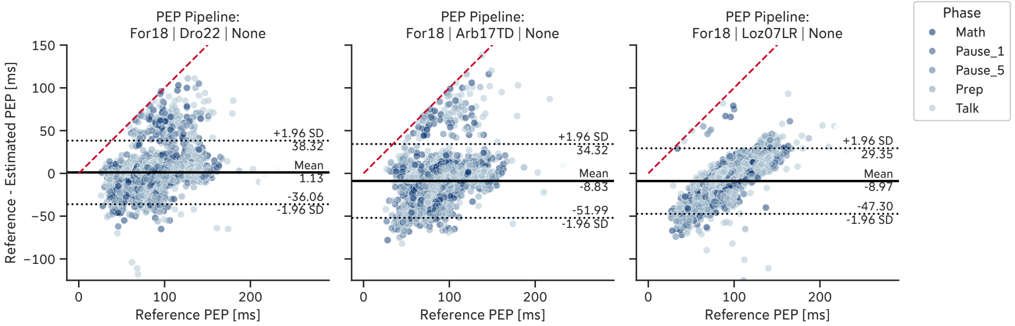

Figure S29: Residual plots of the selected PEP pipelines on the *EmpkinS Dataset*. Each phase is represented by a different color.

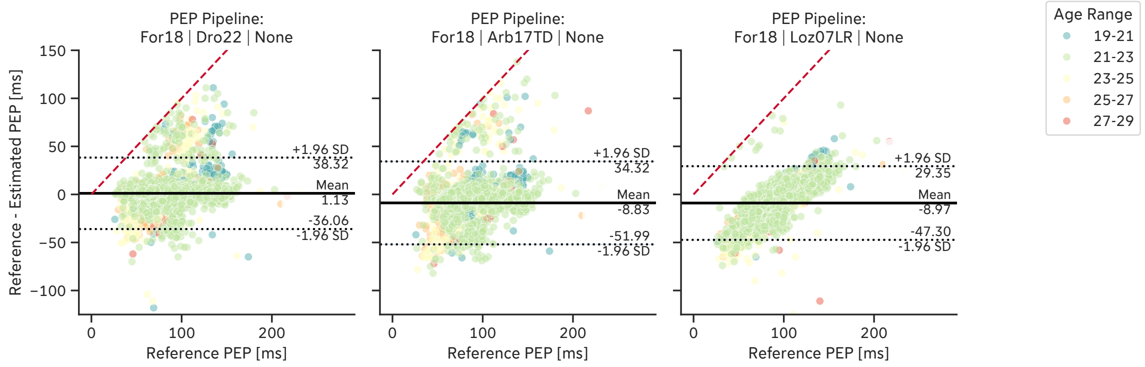

Figure S30: Residual plots of selected PEP pipelines algorithms on the *EmpkinS Dataset*. Each age range is represented by a different color. The red dashed line indicates the upper estimation error limit given by the location of the reference Q-peaks.

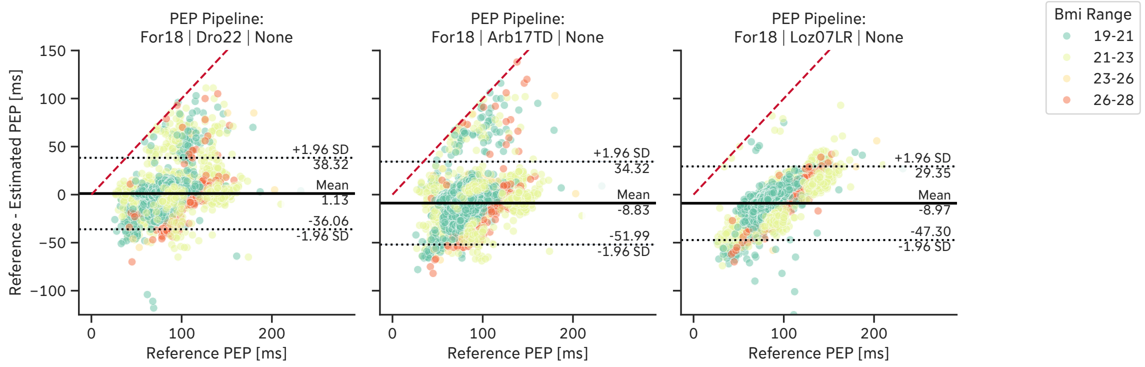

Figure S31: Residual plots of selected PEP pipelines on the *EmpkinS Dataset*. Each BMI range is represented by a different color. The red dashed line indicates the upper estimation error limit given by the location of the reference Q-peaks.

3.4.4 Effect of Heart Rate on PEP Pipeline Extraction Error

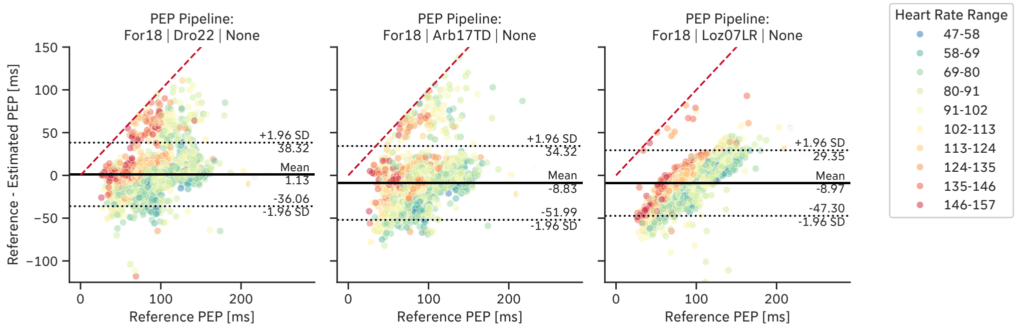

Figure S32: Residual plots of the selected PEP pipelines on the *EmpkinS Dataset*. Each heart rate bin is represented by a different color.

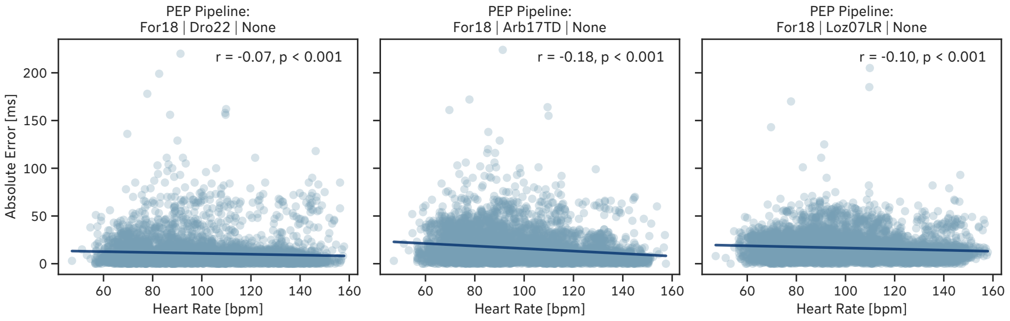

Figure S33: Regression plot between heart rate and absolute error of the selected PEP pipelines on the *EmpkinS Dataset*.

3.4.5 Effect of Outlier Correction on PEP Pipeline Performance

Table S11: Mean Absolute Error (MAE) of the outlier correction algorithms for the different B-point extraction algorithms on the *EmpkinS Dataset*. MAE values are provided in milliseconds as ($M \pm SD$).

Q-peak Algorithm	B-point Algorithm	Outlier Correction Algorithm For18	LinInt	None
For18	Arb17IC	18.1±17.6	**18.0±17.6**	19.4±19.3
	Arb17SD	48.6±22.5	**48.0±22.5**	50.7±23.5
	Arb17TD	17.1±18.1	17.2±18.1	**16.0±17.6**
	Deb93SD	**23.7±14.5**	23.7±14.6	25.7±14.4
	Dro22	10.9±14.7	10.9±14.7	**10.8±15.6**
	For18	30.4±24.6	**30.0±24.5**	31.6±24.8
	Loz07LR	17.4±16.2	17.4±16.3	**16.4±13.9**
	Loz07QR	17.3±14.9	17.4±15.1	**16.8±13.7**
	She90	**17.9±17.3**	17.9±17.3	18.9±18.0
	Ste85	32.4±27.5	**31.8±27.4**	34.2±29.0
Mar04	Arb17IC	**26.6±18.3**	26.8±18.3	27.4±18.3
	Arb17SD	37.2±20.5	**36.6±20.5**	39.7±21.4
	Arb17TD	29.3±22.1	29.5±22.2	**27.9±21.4**
	Deb93SD	18.5±19.4	18.7±19.7	**17.8±18.5**
	Dro22	19.4±18.1	19.6±18.2	**18.9±17.6**
	For18	22.0±25.0	**21.7±24.9**	23.0±25.4
	Loz07LR	27.9±21.8	28.0±21.9	**26.7±19.9**
	Loz07QR	26.5±20.2	26.5±20.4	**25.5±19.0**
	She90	**26.7±18.5**	26.9±18.7	26.9±17.5
	Ste85	24.7±27.7	**24.5±27.6**	26.4±29.4
Van13 (32 ms)	Arb17IC	**23.5±15.3**	23.7±15.4	24.6±16.0
	Arb17SD	39.4±20.8	**38.7±20.7**	42.0±21.8
	Arb17TD	25.4±18.8	25.5±18.9	**24.0±18.4**
	Deb93SD	**17.1±17.4**	17.3±17.6	17.2±17.3
	Dro22	16.0±14.9	16.2±15.0	**15.6±14.8**
	For18	23.6±24.8	**23.3±24.7**	24.9±25.4
	Loz07LR	24.7±18.2	24.8±18.3	**23.5±16.0**
	Loz07QR	23.8±16.6	23.9±16.7	**22.9±15.3**
	She90	**23.6±15.6**	23.8±15.8	24.1±14.9
	Ste85	27.3±28.6	**26.9±28.5**	29.3±30.5
Van13 (34 ms)	Arb17IC	**24.7±15.3**	24.9±15.4	25.7±15.7
	Arb17SD	37.8±20.3	**37.2±20.3**	40.5±21.4
	Arb17TD	27.2±18.9	27.3±19.0	**25.8±18.4**
	Deb93SD	16.4±18.0	16.7±18.2	**16.2±17.8**
	Dro22	17.6±14.9	17.8±15.1	**17.2±14.6**
	For18	22.7±24.5	**22.5±24.5**	24.0±25.2
	Loz07LR	26.2±18.6	26.3±18.8	**25.0±16.5**
	Loz07QR	25.1±17.1	25.2±17.2	**24.2±15.8**
	She90	**24.9±15.7**	25.1±15.9	25.2±14.7
	Ste85	26.8±28.6	**26.5±28.4**	28.8±30.4
Van13 (36 ms)	Arb17IC	**26.0±15.4**	26.2±15.5	26.9±15.5
	Arb17SD	36.3±19.9	**35.7±19.9**	39.1±21.0
	Arb17TD	29.0±18.9	29.2±19.1	**27.6±18.3**
	Deb93SD	16.0±18.6	16.3±18.9	**15.4±18.2**
	Dro22	19.3±15.0	19.5±15.1	**18.8±14.4**
	For18	22.1±24.4	**21.9±24.4**	23.3±25.0
	Loz07LR	27.8±19.2	27.9±19.3	**26.6±17.0**
	Loz07QR	26.5±17.6	26.5±17.7	**25.5±16.3**
	She90	**26.2±15.8**	26.4±16.0	26.4±14.7

Continued on next page

Table S11: Mean Absolute Error (MAE) of the outlier correction algorithms for the different B-point extraction algorithms on the *EmpkinS Dataset*. MAE values are provided in milliseconds as (M ± SD).

**Q-peak Algorithm**	**B-point Algorithm**	**Outlier Correction Algorithm** For18	**LinInt**	**None**
	Ste85	26.5±28.5	**26.2±28.3**	28.4±30.1
Van13 (38 ms)	Arb17IC	**27.3±15.5**	27.5±15.6	28.1±15.5
	Arb17SD	34.9±19.5	**34.2±19.5**	37.6±20.6
	Arb17TD	30.9±19.0	31.1±19.1	**29.5±18.3**
	Deb93SD	15.8±19.3	16.1±19.5	**14.9±18.7**
	Dro22	21.1±15.0	21.3±15.2	**20.5±14.3**
	For18	21.6±24.1	**21.4±24.0**	22.8±24.6
	Loz07LR	29.5±19.5	29.6±19.6	**28.2±17.4**
	Loz07QR	27.9±18.0	28.0±18.2	**26.9±16.7**
	She90	**27.5±16.0**	27.8±16.1	27.6±14.7
	Ste85	26.5±28.4	**26.2±28.2**	28.4±30.0
Van13 (40 ms)	Arb17IC	**28.7±15.7**	28.9±15.8	29.4±15.5
	Arb17SD	33.5±19.2	**32.8±19.2**	36.2±20.3
	Arb17TD	32.8±19.0	33.0±19.2	**31.4±18.2**
	Deb93SD	15.8±19.9	16.2±20.1	**14.5±19.1**
	Dro22	22.9±15.1	23.1±15.2	**22.3±14.1**
	For18	21.4±23.6	**21.2±23.6**	22.6±24.2
	Loz07LR	31.2±19.8	31.3±19.9	**30.0±17.8**
	Loz07QR	29.5±18.4	29.5±18.6	**28.4±17.1**
	She90	**28.9±16.3**	29.2±16.4	28.9±14.8
	Ste85	26.7±28.1	**26.5±28.0**	28.7±29.6
Van13 (42 ms)	Arb17IC	**30.1±16.0**	30.3±16.1	30.7±15.7
	Arb17SD	32.1±18.8	**31.4±18.8**	34.8±19.9
	Arb17TD	34.7±19.0	34.9±19.2	**33.3±18.1**
	Deb93SD	16.1±20.5	16.5±20.8	**14.5±19.6**
	Dro22	24.7±15.1	25.0±15.2	**24.0±13.9**
	For18	21.4±23.2	**21.3±23.2**	22.5±23.6
	Loz07LR	33.0±20.1	33.1±20.2	**31.7±18.0**
	Loz07QR	31.1±18.8	31.2±19.0	**30.0±17.5**
	She90	30.3±16.6	30.7±16.7	**30.2±15.1**
	Ste85	27.1±27.8	**27.0±27.7**	29.2±29.4

3.5 Individually Lowest vs. Overall Lowest PEP Pipelines

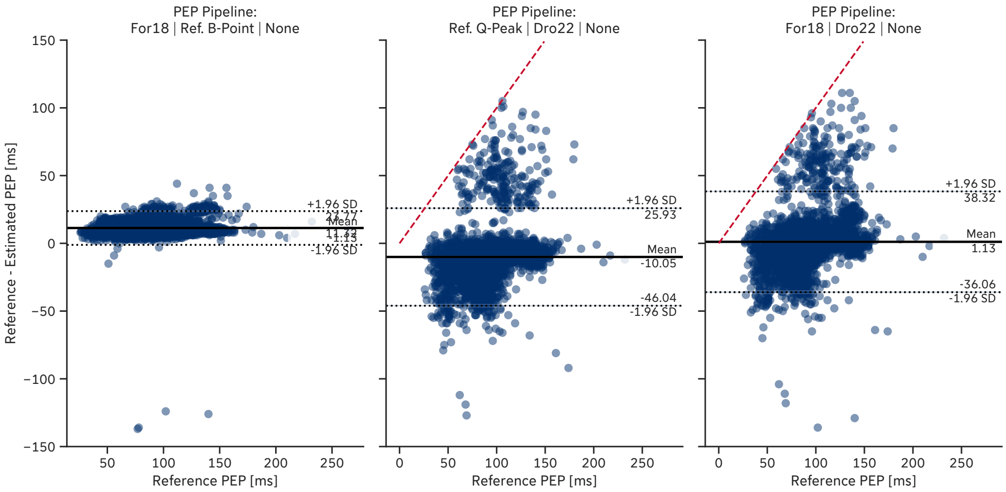

Figure S34: Residual plot of the single PEP pipeline extraction steps of the *individually lowest* PEP pipeline (i.e., the PEP pipeline consisting of the algorithms with the individually lowest MAE) on the *EmpkinS Dataset*.

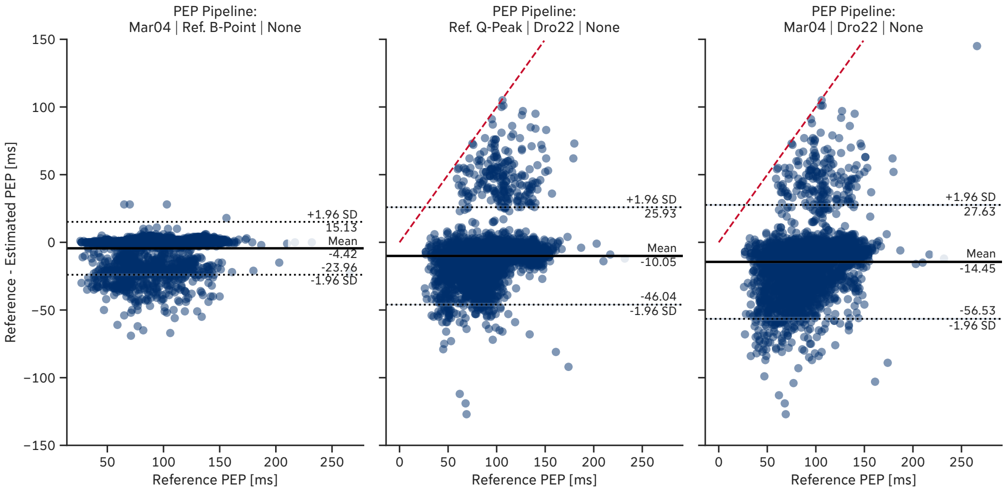

Figure S35: Residual plot of the single PEP pipeline extraction steps of the *overall lowest* PEP pipeline (i.e., the PEP pipeline with the overall lowest MAE) on the *EmpkinS Dataset*.

3.5.1 Effect of Annotations on B-Point Detection Error

Table S12: Error metrics of PEP extraction pipelines on the *EmpkinS Dataset* for different annotators and MAE difference between both annotators. MAE = Mean Absolute Error, ME = Mean Error.

			Annotator 1				Annotator 2				Ann. Diff.	
			MAE [ms]		ME [ms]		MAE [ms]		ME [ms]		MAE [ms]	
Q-peak Algorithm	B-point Algorithm	Outlier Correction Algorithm	Mean	SD	Mean	SD	Mean	SD	Mean	SD	Mean	SD
		None	10.81	15.64	1.13	18.97	10.68	14.14	−4.80	17.06	−0.13	−1.49
For18	Dro22	For18	10.86	14.69	−1.18	18.23	11.42	14.27	−6.86	16.94	0.56	−0.42
		LinInt	10.90	14.73	−1.51	18.26	11.58	14.41	−7.13	17.06	0.68	−0.32
Van13 (42 ms)	Deb93SD	None	14.47	19.62	−5.06	23.85	16.94	22.50	−10.12	26.28	2.47	2.88
Van13 (40 ms)	Deb93SD	None	14.54	19.15	−3.06	23.85	16.75	21.82	−8.12	26.28	2.21	2.67
Van13 (38 ms)	Deb93SD	None	14.87	18.68	−1.06	23.85	16.83	21.10	−6.12	26.28	1.96	2.42
Van13 (36 ms)	Deb93SD	None	15.43	18.21	0.94	23.85	17.15	20.34	−4.12	26.28	1.72	2.13
Van13 (32 ms)	Dro22	None	15.64	14.77	−10.36	18.86	18.32	15.18	−16.39	17.24	2.68	0.40
Van13 (38 ms)	Deb93SD	For18	15.82	19.27	−5.22	24.38	18.64	22.05	−10.60	26.86	2.82	2.79
Van13 (40 ms)	Deb93SD	For18	15.84	19.89	−7.22	24.38	18.94	22.84	−12.60	26.86	3.10	2.95
For18	Arb17TD	None	15.95	17.56	−8.83	22.02	18.95	19.50	−15.10	22.61	3.00	1.94
Van13 (32 ms)	Dro22	For18	16.02	14.88	−12.88	17.67	19.37	15.97	−18.53	16.93	3.34	1.09
Van13 (36 ms)	Deb93SD	For18	16.03	18.64	−3.22	24.38	18.57	21.22	−8.60	26.86	2.54	2.58
Van13 (42 ms)	Deb93SD	For18	16.09	20.50	−9.22	24.38	19.47	23.57	−14.60	26.86	3.38	3.07
Van13 (38 ms)	Deb93SD	LinInt	16.13	19.50	−5.69	24.66	18.96	22.25	−11.06	27.07	2.83	2.76
Van13 (40 ms)	Deb93SD	LinInt	16.17	20.14	−7.69	24.66	19.28	23.05	−13.06	27.07	3.11	2.92
Van13 (34 ms)	Deb93SD	None	16.18	17.77	2.94	23.85	17.70	19.55	−2.12	26.28	1.52	1.78
Van13 (32 ms)	Dro22	LinInt	16.20	14.98	−13.22	17.67	19.59	16.17	−18.81	17.07	3.39	1.19
Van13 (36 ms)	Deb93SD	LinInt	16.31	18.85	−3.69	24.66	18.87	21.42	−9.06	27.07	2.55	2.56
For18	Loz07LR	None	16.41	13.92	−8.97	19.55	19.51	17.02	−15.22	20.95	3.11	3.10
Van13 (34 ms)	Deb93SD	For18	16.43	18.05	−1.22	24.38	18.72	20.36	−6.60	26.86	2.29	2.31
Van13 (42 ms)	Deb93SD	LinInt	16.47	20.80	−9.67	24.70	19.83	23.79	−15.06	27.07	3.36	2.99
Van13 (34 ms)	Deb93SD	LinInt	16.68	18.23	−1.69	24.66	18.99	20.53	−7.06	27.07	2.31	2.30
For18	Loz07QR	None	16.76	13.67	−5.69	20.86	19.32	16.32	−12.16	22.17	2.56	2.65
Van13 (32 ms)	Deb93SD	For18	17.06	17.43	0.78	24.38	19.08	19.45	−4.60	26.86	2.02	2.02
For18	Arb17TD	For18	17.12	18.06	−11.56	22.05	20.53	19.92	−17.38	22.72	3.41	1.85
Van13 (32 ms)	Deb93SD	None	17.16	17.29	4.94	23.85	18.46	18.71	−0.12	26.28	1.31	1.41
For18	Arb17TD	LinInt	17.17	18.09	−12.15	21.78	20.77	19.96	−17.95	22.53	3.60	1.88

Continued on next page

Table S12: Error metrics of PEP extraction pipelines on the *EmpkinS Dataset* for different annotators and MAE difference between both annotators. MAE = Mean Absolute Error, ME = Mean Error.

			Annotator 1				Annotator 2				Ann. Diff.	
			MAE [ms]		ME [ms]		MAE [ms]		ME [ms]		MAE [ms]	
			Mean	SD	Mean	SD	Mean	SD	Mean	SD	Mean	SD
Q-peak Algorithm	B-point Algorithm	Outlier Correction Algorithm										
Van13 (34 ms)	Dro22	None	17.19	14.64	−12.34	18.91	20.15	15.14	−18.39	17.24	2.96	0.50
Van13 (32 ms)	Deb93SD	LinInt	17.26	17.58	0.29	24.64	19.33	19.61	−5.06	27.07	2.07	2.03
For18	Loz07QR	For18	17.29	14.92	−7.24	21.65	19.75	16.69	−13.09	22.30	2.46	1.77
	Loz07LR	For18	17.35	16.18	−10.38	21.33	20.26	18.34	−16.20	22.01	2.91	2.16
	Loz07QR	LinInt	17.36	15.06	−7.36	21.77	19.80	16.73	−13.18	22.32	2.44	1.67
	Loz07LR	LinInt	17.41	16.32	−10.49	21.44	20.29	18.33	−16.27	21.98	2.88	2.01
Van13 (34 ms)	Dro22	For18	17.61	14.95	−14.88	17.67	21.24	16.04	−20.53	16.93	3.63	1.09
Mar04	Deb93SD	None	17.79	18.52	1.20	25.66	19.50	20.68	−4.07	28.13	1.71	2.16
Van13 (34 ms)	Dro22	LinInt	17.81	15.06	−15.22	17.67	21.47	16.24	−20.81	17.07	3.66	1.17
For18	She90	For18	17.90	17.26	−2.99	24.68	19.12	16.19	−8.67	23.51	1.23	−1.07
		LinInt	17.92	17.32	−3.48	24.68	19.34	16.33	−9.13	23.61	1.42	−0.99
	Arb17IC	LinInt	18.01	17.58	−2.11	25.08	19.22	16.50	−8.07	24.02	1.22	−1.07
		For18	18.06	17.58	−1.69	25.14	19.13	16.48	−7.73	24.04	1.08	−1.10
Mar04	Deb93SD	For18	18.52	19.45	−3.38	26.65	21.05	22.09	−9.02	29.15	2.53	2.64
		LinInt	18.73	19.66	−3.86	26.88	21.26	22.30	−9.49	29.32	2.53	2.64
Van13 (36 ms)	Dro22	None	18.83	14.45	−14.34	18.91	22.05	15.07	−20.39	17.24	3.22	0.62
For18	She90	None	18.90	18.01	−0.19	26.11	19.76	16.90	−6.00	25.30	0.86	−1.11
Mar04	Dro22	None	18.94	17.64	−14.45	21.47	22.42	18.16	−20.69	20.10	3.48	0.52
Van13 (36 ms)	Dro22	For18	19.31	14.98	−16.88	17.67	23.17	16.05	−22.53	16.93	3.86	1.07
Mar04	Dro22	For18	19.43	18.06	−16.97	20.39	23.54	19.17	−22.86	19.97	4.11	1.10
For18	Arb17IC	None	19.45	19.32	1.16	27.39	20.30	18.05	−4.91	26.72	0.85	−1.27
Van13 (36 ms)	Dro22	LinInt	19.52	15.14	−17.20	17.74	23.40	16.25	−22.81	17.07	3.88	1.11
Mar04	Dro22	LinInt	19.61	18.16	−17.31	20.37	23.75	19.34	−23.14	20.07	4.14	1.18
Van13 (38 ms)	Dro22	None	20.53	14.26	−16.34	18.91	23.96	14.99	−22.39	17.24	3.43	0.73
		For18	21.06	15.01	−18.88	17.67	25.12	16.05	−24.53	16.93	4.06	1.05
Van13 (40 ms)	For18	LinInt	21.23	23.61	10.08	30.11	18.60	21.85	4.92	28.27	−2.63	−1.77
Van13 (42 ms)	For18	LinInt	21.29	23.20	8.30	30.37	19.14	21.45	3.14	28.58	−2.15	−1.74
Van13 (38 ms)	Dro22	LinInt	21.31	15.19	−19.17	17.81	25.35	16.26	−24.81	17.07	4.04	1.07
Van13 (42 ms)	For18	For18	21.41	23.21	8.89	30.30	19.19	21.48	3.63	28.58	−2.21	−1.73
Van13 (40 ms)	For18	For18	21.41	23.65	10.70	30.06	18.72	21.90	5.44	28.29	−2.70	−1.75
Van13 (38 ms)	For18	LinInt	21.43	24.02	11.87	29.92	18.30	22.29	6.70	28.05	−3.13	−1.73
		For18	21.64	24.06	12.47	29.86	18.43	22.35	7.19	28.06	−3.21	−1.71
Mar04	For18	LinInt	21.72	24.89	12.85	30.43	18.63	23.18	7.55	28.76	−3.10	−1.71
Van13 (36 ms)	For18	LinInt	21.90	24.35	13.72	29.74	18.35	22.67	8.56	27.88	−3.55	−1.68
Mar04	For18	For18	22.02	24.99	13.47	30.47	18.81	23.30	8.05	28.85	−3.21	−1.69
Van13 (36 ms)	For18	For18	22.14	24.39	14.31	29.67	18.51	22.75	9.05	27.90	−3.63	−1.65

Continued on next page

Table S12: Error metrics of PEP extraction pipelines on the *EmpkinS Dataset* for different annotators and MAE difference between both annotators. MAE = Mean Absolute Error, ME = Mean Error.

			Annotator 1				Annotator 2				Ann. Diff.	
			MAE [ms]		ME [ms]		MAE [ms]		ME [ms]		MAE [ms]	
**Q-peak Algorithm**	**B-point Algorithm**	**Outlier Correction Algorithm**	**Mean**	**SD**	**Mean**	**SD**	**Mean**	**SD**	**Mean**	**SD**	**Mean**	**SD**
Van13 (40 ms)	Dro22	None	22.27	14.07	−18.34	18.91	25.88	14.92	−24.39	17.24	3.61	0.84
Van13 (34 ms)	For18	LinInt	22.46	24.49	15.42	29.44	18.58	22.78	10.26	27.55	−3.87	−1.71
Van13 (42 ms)	For18	None	22.52	23.63	12.87	30.00	20.05	21.60	7.40	28.53	−2.46	−2.03
Van13 (40 ms)	For18	None	22.57	24.20	14.59	29.70	19.62	22.18	9.12	28.18	−2.95	−2.02
Van13 (34 ms)	For18	For18	22.71	24.55	16.00	29.37	18.77	22.86	10.74	27.56	−3.94	−1.68
Van13 (38 ms)	For18	None	22.77	24.58	16.21	29.32	19.36	22.72	10.76	27.85	−3.41	−1.86
Van13 (40 ms)	Dro22	For18	22.87	15.07	−20.86	17.75	27.07	16.05	−26.53	16.93	4.20	0.99
Van13 (32 ms)	Loz07QR	None	22.94	15.29	−17.31	21.46	27.27	18.73	−23.78	23.00	4.33	3.45
Mar04	For18	None	23.00	25.41	17.09	29.71	19.64	23.49	11.50	28.37	−3.37	−1.92
Van13 (40 ms)	Dro22	LinInt	23.12	15.20	−21.17	17.81	27.31	16.26	−26.81	17.07	4.19	1.06
Van13 (32 ms)	For18	LinInt	23.31	24.74	17.24	29.30	19.16	22.95	12.05	27.37	−4.15	−1.79
Van13 (36 ms)	For18	None	23.35	25.02	18.00	29.11	19.51	23.22	12.55	27.61	−3.84	−1.80
Van13 (32 ms)	Loz07LR	None	23.52	16.00	−20.68	19.53	28.40	19.36	−26.87	21.43	4.87	3.36
	Arb17IC	For18	23.54	15.33	−13.29	24.76	26.60	15.70	−19.36	24.08	3.06	0.37
	For18	For18	23.60	24.79	17.83	29.22	19.39	23.04	12.54	27.38	−4.20	−1.75
	She90	For18	23.62	15.59	−14.67	24.20	26.82	15.77	−20.31	23.56	3.20	0.18
For18	Deb93SD	LinInt	23.66	14.57	12.02	25.06	23.87	14.29	6.61	27.02	0.20	−0.27
Van13 (32 ms)	Arb17IC	LinInt	23.67	15.41	−13.70	24.70	26.82	15.76	−19.68	24.09	3.14	0.35
For18	Deb93SD	For18	23.72	14.48	12.51	24.82	23.86	14.16	7.08	26.83	0.14	−0.33
Van13 (32 ms)	Loz07QR	For18	23.80	16.60	−18.86	22.05	27.88	19.15	−24.71	23.10	4.08	2.55
	She90	LinInt	23.82	15.77	−15.14	24.23	27.14	15.97	−20.78	23.66	3.32	0.20
	Loz07QR	LinInt	23.89	16.74	−18.97	22.15	27.94	19.20	−24.80	23.12	4.06	2.46
Van13 (34 ms)	For18	None	23.98	25.22	19.63	28.73	19.84	23.41	14.17	27.22	−4.14	−1.80
Van13 (32 ms)	Arb17TD	None	24.02	18.41	−19.84	22.86	28.47	20.53	−26.22	23.34	4.45	2.12
Van13 (42 ms)	Dro22	None	24.04	13.91	−20.34	18.91	27.80	14.86	−26.39	17.24	3.76	0.95
Van13 (32 ms)	She90	None	24.06	14.90	−11.84	25.70	26.81	15.29	−17.65	25.32	2.75	0.40
Van13 (34 ms)	Loz07QR	None	24.16	15.79	−19.31	21.46	28.75	19.16	−25.78	23.00	4.58	3.37
For18	Mil22	LinInt	24.42	51.98	−1.72	57.41	18.33	16.91	−1.45	24.90	−6.09	−35.07
		For18	24.46	51.68	−0.93	57.17	18.25	16.79	−0.64	24.79	−6.21	−34.89
Mar04	Ste85	LinInt	24.47	27.63	15.95	33.28	20.70	26.26	11.52	31.39	−3.76	−1.37
Van13 (32 ms)	Arb17IC	None	24.63	16.02	−10.15	27.57	27.28	16.12	−16.30	27.17	2.64	0.11
Mar04	Ste85	For18	24.69	27.66	16.73	33.09	20.85	26.27	12.25	31.22	−3.84	−1.39

Continued on next page

Table S12: Error metrics of PEP extraction pipelines on the *EmpkinS Dataset* for different annotators and MAE difference between both annotators. MAE = Mean Absolute Error, ME = Mean Error.

			Annotator 1				Annotator 2				Ann. Diff.	
			MAE [ms]		ME [ms]		MAE [ms]		ME [ms]		MAE [ms]	
Q-peak Algorithm	B-point Algorithm	Outlier Correction Algorithm	Mean	SD	Mean	SD	Mean	SD	Mean	SD	Mean	SD
Van13 (42 ms)	Dro22	For18	24.70	15.08	−22.86	17.75	29.04	16.05	−28.53	16.93	4.34	0.97
Van13 (32 ms)	Loz07LR	For18	24.70	18.20	−22.09	21.29	29.27	20.62	−27.85	22.50	4.58	2.42
Van13 (34 ms)	Arb17IC	For18	24.74	15.33	−15.27	24.78	28.07	15.77	−21.34	24.11	3.33	0.44
Van13 (32 ms)	Loz07LR	LinInt	24.78	18.34	−22.20	21.39	29.32	20.61	−27.92	22.48	4.54	2.27
	For18	None	24.87	25.43	21.34	28.46	20.51	23.62	15.89	26.95	−4.36	−1.81
Van13 (34 ms)	She90	For18	24.88	15.67	−16.65	24.23	28.30	15.87	−22.31	23.56	3.43	0.20
	Arb17IC	LinInt	24.89	15.39	−15.70	24.70	28.28	15.82	−21.68	24.09	3.39	0.42
Van13 (42 ms)	Dro22	LinInt	24.96	15.21	−23.17	17.81	29.28	16.26	−28.81	17.07	4.32	1.05
Van13 (34 ms)	Loz07LR	None	24.99	16.48	−22.68	19.53	30.06	19.72	−28.87	21.43	5.07	3.24
	Loz07QR	For18	25.07	17.10	−20.86	22.05	29.39	19.56	−26.71	23.10	4.32	2.46
	She90	LinInt	25.11	15.85	−17.12	24.26	28.64	16.08	−22.78	23.66	3.53	0.23
	Loz07QR	LinInt	25.16	17.25	−20.97	22.15	29.46	19.62	−26.80	23.12	4.30	2.37
	She90	None	25.19	14.74	−13.84	25.70	28.18	15.26	−19.65	25.32	2.99	0.52
Van13 (32 ms)	Arb17TD	For18	25.39	18.80	−22.88	21.79	30.27	20.76	−28.80	22.76	4.88	1.96
Mar04	Loz07QR	None	25.46	18.96	−21.40	23.44	30.45	22.21	−28.16	25.05	4.99	3.25
Van13 (36 ms)	Loz07QR	None	25.50	16.26	−21.31	21.46	30.30	19.56	−27.78	23.00	4.81	3.29
Van13 (32 ms)	Arb17TD	LinInt	25.50	18.85	−23.60	21.18	30.57	20.89	−29.43	22.47	5.07	2.03
For18	Mil22	None	25.60	50.64	2.86	56.68	19.27	18.65	2.78	26.67	−6.33	−31.99
Van13 (32 ms)	Mil22	For18	25.68	52.87	−11.80	57.58	20.89	18.62	−11.62	25.46	−4.79	−34.25
For18	Deb93SD	None	25.69	14.37	16.65	24.27	25.38	13.16	11.55	26.15	−0.31	−1.22
Van13 (34 ms)	Arb17IC	None	25.73	15.75	−12.12	27.63	28.61	15.97	−18.29	27.18	2.87	0.22
	Arb17TD	None	25.80	18.41	−21.74	23.07	30.37	20.57	−28.11	23.56	4.57	2.16
Van13 (32 ms)	Mil22	LinInt	25.82	53.23	−12.72	57.77	21.24	18.92	−12.43	25.59	−4.58	−34.31
Van13 (36 ms)	Arb17IC	For18	25.99	15.39	−17.27	24.78	29.56	15.88	−23.34	24.11	3.57	0.48
	Ste85	LinInt	26.16	28.28	18.05	34.03	21.87	26.84	13.82	31.74	−4.29	−1.44
	Arb17IC	LinInt	26.17	15.45	−17.70	24.70	29.79	15.93	−23.68	24.09	3.62	0.47
	She90	For18	26.18	15.79	−18.65	24.23	29.82	16.04	−24.31	23.56	3.64	0.24
Van13 (34 ms)	Loz07LR	For18	26.19	18.64	−24.09	21.29	30.95	20.97	−29.85	22.50	4.76	2.32
Van13 (32 ms)	Mil22	None	26.23	51.61	−7.58	57.40	20.99	19.71	−7.90	27.69	−5.24	−31.90
Van13 (38 ms)	Ste85	LinInt	26.24	28.22	16.83	34.67	22.30	26.73	12.62	32.45	−3.94	−1.50
Van13 (34 ms)	Loz07LR	LinInt	26.28	18.78	−24.20	21.39	31.01	20.95	−29.92	22.48	4.73	2.18
Mar04	Ste85	None	26.38	29.39	20.14	33.98	22.27	28.14	15.53	32.35	−4.11	−1.25
Van13 (36 ms)	She90	None	26.38	14.68	−15.84	25.70	29.58	15.32	−21.65	25.32	3.20	0.65
		LinInt	26.44	15.96	−19.12	24.26	30.16	16.25	−24.78	23.66	3.72	0.29
	Loz07QR	For18	26.45	17.58	−22.86	22.05	30.98	19.95	−28.71	23.10	4.53	2.37
Mar04	Loz07QR	For18	26.46	20.24	−23.00	24.10	31.18	22.63	−29.11	25.24	4.72	2.39
Van13 (34 ms)	Mil22	For18	26.47	53.00	−13.62	57.66	22.03	19.00	−13.38	25.84	−4.44	−34.01
	Ste85	LinInt	26.48	28.45	19.50	33.63	21.91	26.98	15.22	31.25	−4.57	−1.46

Continued on next page

Table S12: Error metrics of PEP extraction pipelines on the *EmpkinS Dataset* for different annotators and MAE difference between both annotators. MAE = Mean Absolute Error, ME = Mean Error.

Q-peak Algorithm	B-point Algorithm	Outlier Correction Algorithm	Annotator 1 MAE [ms] Mean	Annotator 1 MAE [ms] SD	Annotator 1 ME [ms] Mean	Annotator 1 ME [ms] SD	Annotator 2 MAE [ms] Mean	Annotator 2 MAE [ms] SD	Annotator 2 ME [ms] Mean	Annotator 2 ME [ms] SD	Ann. Diff. MAE [ms] Mean	Ann. Diff. MAE [ms] SD
Van13 (40 ms)	Ste85	LinInt	26.49	27.97	15.60	35.23	23.05	26.46	11.46	33.17	−3.44	−1.51
Van13 (36 ms)	Ste85	For18	26.50	28.47	19.00	33.94	22.11	27.00	14.70	31.66	−4.39	−1.46
Van13 (38 ms)	Ste85	For18	26.50	28.39	17.75	34.55	22.47	26.84	13.44	32.32	−4.04	−1.56
Van13 (36 ms)	Loz07QR	LinInt	26.54	17.73	−22.97	22.15	31.04	20.01	−28.80	23.12	4.51	2.28
Mar04	Loz07QR	LinInt	26.55	20.37	−23.12	24.20	31.25	22.67	−29.20	25.26	4.70	2.30
	Arb17IC	For18	26.57	18.26	−17.40	27.14	30.12	18.89	−23.67	26.52	3.55	0.63
Van13 (36 ms)	Loz07LR	None	26.60	17.05	−24.62	19.80	31.79	20.04	−30.85	21.45	5.19	3.00
Van13 (34 ms)	Mil22	LinInt	26.64	53.37	−14.56	57.84	22.41	19.29	−14.21	25.93	−4.23	−34.08
Mar04	She90	For18	26.68	18.48	−18.85	26.42	30.34	18.99	−24.71	25.90	3.66	0.52
	Loz07LR	None	26.69	19.91	−24.61	22.42	32.08	22.79	−31.13	24.07	5.40	2.88
Van13 (40 ms)	Ste85	For18	26.69	28.10	16.49	35.07	23.13	26.48	12.24	32.96	−3.56	−1.62
Mar04	Arb17IC	LinInt	26.76	18.31	−17.84	27.08	30.38	18.92	−24.02	26.52	3.61	0.61
Van13 (34 ms)	Ste85	For18	26.79	28.56	20.36	33.45	22.12	27.09	16.01	31.09	−4.67	−1.47
Van13 (36 ms)	Arb17IC	None	26.88	15.55	−14.08	27.68	29.99	15.90	−20.25	27.24	3.11	0.35
Mar04	She90	None	26.90	17.45	−16.03	27.77	30.17	18.20	−22.02	27.51	3.27	0.74
Van13 (32 ms)	Ste85	LinInt	26.91	28.50	20.85	33.19	22.13	26.97	16.53	30.72	−4.78	−1.53
Van13 (34 ms)	Mil22	None	26.91	51.67	−9.34	57.51	21.99	19.91	−9.63	28.06	−4.92	−31.76
Van13 (38 ms)	Loz07QR	None	26.92	16.71	−23.31	21.46	31.93	19.90	−29.78	23.00	5.02	3.19
Mar04	She90	LinInt	26.94	18.69	−19.32	26.49	30.69	19.23	−25.18	26.03	3.75	0.54
Van13 (42 ms)	Ste85	LinInt	26.97	27.71	14.38	35.90	23.95	26.04	10.23	33.87	−3.02	−1.67
		For18	27.11	27.82	15.25	35.73	23.99	26.06	10.99	33.67	−3.13	−1.76
Van13 (34 ms)	Arb17TD	For18	27.19	18.90	−24.83	21.91	32.17	20.83	−30.75	22.89	4.98	1.93
Van13 (32 ms)	Ste85	For18	27.29	28.64	21.76	33.04	22.42	27.14	17.39	30.61	−4.87	−1.51
Van13 (38 ms)	Arb17IC	For18	27.31	15.50	−19.26	24.80	31.08	16.04	−25.34	24.11	3.77	0.53
Van13 (34 ms)	Arb17TD	LinInt	27.33	19.01	−25.53	21.37	32.49	20.97	−31.38	22.60	5.16	1.96
Mar04	Arb17IC	None	27.36	18.31	−14.15	29.73	30.55	18.74	−20.52	29.38	3.19	0.42
Van13 (36 ms)	Mil22	For18	27.43	53.11	−15.46	57.74	23.28	19.18	−15.25	26.03	−4.15	−33.93
Van13 (38 ms)	Arb17IC	LinInt	27.50	15.57	−19.68	24.73	31.32	16.09	−25.68	24.09	3.81	0.52
	She90	For18	27.53	15.99	−20.65	24.23	31.35	16.26	−26.31	23.56	3.83	0.27
		None	27.61	14.71	−17.84	25.70	31.00	15.47	−23.65	25.32	3.39	0.76
Van13 (36 ms)	Arb17TD	None	27.62	18.35	−23.64	23.26	32.27	20.55	−30.07	23.66	4.65	2.20
	Mil22	LinInt	27.63	53.48	−16.43	57.91	23.68	19.46	−16.10	26.09	−3.95	−34.01
Van13 (38 ms)	She90	LinInt	27.82	16.14	−21.12	24.26	31.72	16.46	−26.78	23.66	3.90	0.32

Continued on next page

Table S12: Error metrics of PEP extraction pipelines on the *EmpkinS Dataset* for different annotators and MAE difference between both annotators. MAE = Mean Absolute Error, ME = Mean Error.

			**Annotator 1**				**Annotator 2**				**Ann. Diff.**	
			**MAE [ms]**		**ME [ms]**		**MAE [ms]**		**ME [ms]**		**MAE [ms]**	
**Q-peak Algorithm**	**B-point Algorithm**	**Outlier Correction Algorithm**	**Mean**	**SD**	**Mean**	**SD**	**Mean**	**SD**	**Mean**	**SD**	**Mean**	**SD**
Van13 (36 ms)	Loz07LR	For18	27.83	19.15	−26.03	21.54	32.70	21.27	−31.84	22.53	4.87	2.11
	Mil22	None	27.87	52.14	−11.31	58.03	23.11	19.90	−11.46	28.26	−4.77	−32.24
Mar04	Arb17TD	None	27.91	21.36	−23.82	25.85	32.88	23.42	−30.33	26.64	4.97	2.06
Van13 (38 ms)	Loz07QR	For18	27.91	18.03	−24.86	22.05	32.63	20.28	−30.71	23.10	4.72	2.26
Van13 (36 ms)	Loz07LR	LinInt	27.92	19.28	−26.13	21.64	32.75	21.25	−31.90	22.50	4.83	1.97
Mar04	Loz07LR	For18	27.94	21.79	−26.06	24.00	33.03	23.93	−32.15	25.10	5.09	2.14
Van13 (38 ms)	Loz07QR	LinInt	28.00	18.18	−24.97	22.15	32.70	20.34	−30.80	23.12	4.70	2.16
Mar04	Loz07LR	LinInt	28.03	21.89	−26.17	24.08	33.09	23.90	−32.21	25.07	5.06	2.02
Van13 (38 ms)	Arb17IC	None	28.12	15.49	−15.97	27.86	31.41	15.94	−22.19	27.36	3.28	0.45
Mar04	Mil22	None	28.16	51.89	−12.20	57.77	23.69	22.36	−12.54	30.06	−4.47	−29.54
		For18	28.20	53.42	−16.47	58.12	24.04	22.08	−16.33	28.26	−4.17	−31.34
Van13 (38 ms)	Loz07LR	None	28.25	17.43	−26.60	19.85	33.58	20.32	−32.83	21.52	5.34	2.90
Mar04	Mil22	LinInt	28.41	53.79	−17.35	58.31	24.43	22.41	−17.18	28.36	−3.98	−31.38
Van13 (36 ms)	Ste85	None	28.42	30.14	22.42	34.84	23.83	28.98	18.03	32.90	−4.60	−1.16
Van13 (38 ms)	Ste85	None	28.43	29.97	21.26	35.42	24.21	28.70	16.89	33.54	−4.21	−1.27
Van13 (40 ms)	Loz07QR	None	28.43	17.11	−25.31	21.46	33.63	20.20	−31.78	23.00	5.20	3.09
Van13 (38 ms)	Mil22	For18	28.51	53.20	−17.33	57.82	24.69	19.34	−17.10	26.29	−3.82	−33.86
Van13 (40 ms)	Ste85	None	28.65	29.61	20.16	35.94	24.98	28.25	15.87	34.22	−3.67	−1.36
	Arb17IC	For18	28.66	15.71	−21.24	24.84	32.62	16.25	−27.34	24.11	3.96	0.55
Van13 (38 ms)	Mil22	LinInt	28.76	53.57	−18.31	57.98	25.12	19.64	−17.96	26.35	−3.64	−33.93
Van13 (34 ms)	Ste85	None	28.78	30.36	23.76	34.43	23.87	29.17	19.30	32.38	−4.91	−1.18
Van13 (40 ms)	Arb17IC	LinInt	28.87	15.78	−21.66	24.77	32.87	16.30	−27.67	24.10	4.00	0.52
Van13 (38 ms)	Mil22	None	28.87	52.17	−13.07	58.17	24.44	20.03	−13.19	28.72	−4.43	−32.14
Van13 (40 ms)	She90	None	28.87	14.84	−19.84	25.70	32.45	15.70	−25.65	25.32	3.57	0.85
		For18	28.91	16.26	−22.65	24.23	32.92	16.53	−28.31	23.56	4.00	0.28
Van13 (36 ms)	Arb17TD	For18	29.02	18.94	−26.81	21.95	34.08	20.87	−32.72	22.93	5.06	1.93
		LinInt	29.18	19.06	−27.53	21.37	34.40	21.00	−33.38	22.60	5.23	1.94
Van13 (42 ms)	Ste85	None	29.20	29.36	19.16	36.72	25.98	27.85	14.86	35.07	−3.22	−1.51
Van13 (40 ms)	She90	LinInt	29.23	16.40	−23.12	24.26	33.29	16.72	−28.78	23.66	4.07	0.32
Van13 (32 ms)	Ste85	None	29.26	30.47	25.04	34.03	24.14	29.25	20.53	31.89	−5.12	−1.22
Mar04	Arb17TD	For18	29.27	22.13	−27.09	24.75	34.59	23.97	−33.15	25.93	5.33	1.83
Van13 (40 ms)	Arb17IC	None	29.36	15.48	−17.92	27.93	32.81	16.03	−24.19	27.36	3.45	0.54
	Loz07QR	For18	29.46	18.43	−26.86	22.05	34.34	20.59	−32.71	23.10	4.88	2.16
Van13 (38 ms)	Arb17TD	None	29.50	18.27	−25.48	23.56	34.22	20.54	−31.88	24.01	4.72	2.27
	Loz07LR	For18	29.50	19.50	−28.01	21.59	34.50	21.53	−33.82	22.59	5.00	2.03

Continued on next page

Table S12: Error metrics of PEP extraction pipelines on the *EmpkinS Dataset* for different annotators and MAE difference between both annotators. MAE = Mean Absolute Error, ME = Mean Error.

			Annotator 1				Annotator 2				Ann. Diff.	
			MAE [ms]		ME [ms]		MAE [ms]		ME [ms]		MAE [ms]	
Q-peak Algorithm	B-point Algorithm	Outlier Correction Algorithm	Mean	SD	Mean	SD	Mean	SD	Mean	SD	Mean	SD
Mar04	Arb17TD	LinInt	29.53	22.22	−27.77	24.38	35.00	24.08	−33.76	25.78	5.47	1.86
Van13 (40 ms)	Loz07QR	LinInt	29.54	18.59	−26.97	22.15	34.41	20.65	−32.80	23.12	4.87	2.06
Van13 (38 ms)	Loz07LR	LinInt	29.59	19.62	−28.11	21.69	34.56	21.51	−33.88	22.56	4.96	1.89
Van13 (40 ms)	Mil22	For18	29.68	53.27	−19.26	57.86	26.13	19.40	−19.04	26.39	−3.55	−33.87
		None	29.93	52.15	−14.93	58.25	25.76	19.89	−15.10	28.83	−4.17	−32.26
	Loz07LR	None	29.96	17.76	−28.58	19.91	35.42	20.55	−34.81	21.57	5.46	2.80
For18	For18	LinInt	29.96	24.52	26.85	27.89	25.21	21.96	21.77	25.38	−4.76	−2.55
Van13 (40 ms)	Mil22	LinInt	29.99	53.64	−20.23	58.03	26.60	19.70	−19.90	26.46	−3.38	−33.94
Van13 (42 ms)	Loz07QR	None	30.00	17.49	−27.31	21.46	35.38	20.46	−33.78	23.00	5.37	2.97
	Arb17IC	For18	30.07	16.02	−23.19	24.97	34.21	16.56	−29.31	24.20	4.14	0.54
	She90	None	30.17	15.07	−21.84	25.70	33.91	15.99	−27.65	25.32	3.74	0.92
	Arb17IC	LinInt	30.29	16.11	−23.61	24.90	34.46	16.61	−29.64	24.19	4.17	0.50
	She90	For18	30.34	16.57	−24.65	24.23	34.50	16.84	−30.31	23.56	4.16	0.28
For18	For18	For18	30.40	24.55	27.45	27.82	25.58	22.05	22.25	25.40	−4.82	−2.51
Van13 (42 ms)	Arb17IC	None	30.66	15.66	−19.79	28.17	34.29	16.28	−26.07	27.59	3.63	0.62
	She90	LinInt	30.66	16.71	−25.12	24.26	34.89	17.03	−30.78	23.66	4.23	0.32
Van13 (38 ms)	Arb17TD	For18	30.88	18.96	−28.77	22.03	36.01	20.89	−34.66	23.06	5.13	1.93
Van13 (42 ms)	Mil22	For18	30.95	53.32	−21.17	57.90	27.64	19.44	−20.98	26.49	−3.31	−33.89
	Loz07QR	For18	31.06	18.81	−28.86	22.05	36.11	20.84	−34.71	23.10	5.04	2.03
Van13 (38 ms)	Arb17TD	LinInt	31.08	19.12	−29.47	21.52	36.35	21.02	−35.30	22.76	5.28	1.90
Van13 (42 ms)	Loz07QR	LinInt	31.15	18.96	−28.97	22.15	36.18	20.90	−34.80	23.12	5.02	1.94
	Mil22	None	31.21	52.54	−16.97	58.71	27.15	19.76	−17.00	28.96	−4.05	−32.79
Van13 (40 ms)	Loz07LR	For18	31.23	19.79	−29.99	21.64	36.35	21.74	−35.79	22.64	5.11	1.95
Van13 (42 ms)	Mil22	LinInt	31.29	53.69	−22.16	58.06	28.15	19.73	−21.83	26.55	−3.15	−33.96
Van13 (40 ms)	Loz07LR	LinInt	31.32	19.92	−30.09	21.74	36.41	21.72	−35.86	22.62	5.08	1.80
	Arb17TD	None	31.37	18.17	−27.38	23.76	36.15	20.50	−33.79	24.19	4.77	2.33
Van13 (42 ms)	Arb17SD	LinInt	31.43	18.78	26.54	25.21	30.00	16.73	20.36	27.67	−1.43	−2.05
For18	For18	None	31.58	24.82	30.29	26.38	26.73	22.43	24.96	24.39	−4.84	−2.39
Van13 (42 ms)	Loz07LR	None	31.72	18.05	−30.55	19.97	37.29	20.72	−36.78	21.62	5.57	2.67
For18	Ste85	LinInt	31.82	27.44	28.75	30.64	26.81	24.88	24.39	27.26	−5.01	−2.56
Van13 (42 ms)	Arb17SD	For18	32.07	18.78	27.27	25.25	30.58	16.67	21.07	27.73	−1.49	−2.11
For18	Ste85	For18	32.36	27.51	29.56	30.50	27.22	25.04	25.14	27.13	−5.14	−2.47
Van13 (40 ms)	Arb17TD	For18	32.78	19.00	−30.70	22.21	37.96	20.92	−36.59	23.22	5.17	1.92
	Arb17SD	LinInt	32.82	19.17	28.48	25.17	31.07	17.22	22.31	27.64	−1.75	−1.95
	Arb17TD	LinInt	33.00	19.18	−31.40	21.71	38.31	21.05	−37.22	22.93	5.31	1.88
Van13 (42 ms)	Loz07LR	For18	33.01	20.06	−31.95	21.70	38.23	21.89	−37.76	22.69	5.22	1.84
		LinInt	33.10	20.18	−32.06	21.80	38.29	21.87	−37.83	22.66	5.19	1.69
	Arb17TD	None	33.30	18.14	−29.16	24.25	38.12	20.48	−35.58	24.63	4.81	2.34
Van13 (40 ms)	Arb17SD	For18	33.47	19.17	29.20	25.20	31.67	17.16	23.01	27.71	−1.80	−2.01
For18	Ste85	None	34.17	28.97	32.17	31.17	28.83	26.98	27.59	28.25	−5.34	−1.99

Continued on next page

Table S12: Error metrics of PEP extraction pipelines on the *EmpkinS Dataset* for different annotators and MAE difference between both annotators. MAE = Mean Absolute Error, ME = Mean Error.

			**Annotator 1**				**Annotator 2**				**Ann. Diff.**	
			**MAE [ms]**		**ME [ms]**		**MAE [ms]**		**ME [ms]**		**MAE [ms]**	
**Q-peak Algorithm**	**B-point Algorithm**	**Outlier Correction Algorithm**	**Mean**	**SD**	**Mean**	**SD**	**Mean**	**SD**	**Mean**	**SD**	**Mean**	**SD**
Van13 (38 ms)	Arb17SD	LinInt	34.23	19.52	30.39	25.08	32.16	17.71	24.25	27.57	−2.07	−1.81
Van13 (42 ms)	Arb17TD	For18	34.70	19.04	−32.56	22.51	39.90	20.92	−38.49	23.43	5.20	1.88
	Arb17SD	None	34.83	19.92	30.20	26.42	33.15	17.48	23.94	28.84	−1.68	−2.44
Van13 (38 ms)	Arb17SD	For18	34.89	19.52	31.11	25.11	32.77	17.65	24.95	27.63	−2.11	−1.88
Van13 (42 ms)	Arb17TD	LinInt	34.95	19.24	−33.28	21.99	40.28	21.08	−39.13	23.14	5.33	1.84
Van13 (36 ms)	Arb17SD	LinInt	35.68	19.92	32.31	25.02	33.28	18.31	26.17	27.53	−2.40	−1.62
Van13 (40 ms)	Arb17SD	None	36.25	20.30	32.11	26.37	34.26	18.00	25.85	28.80	−1.98	−2.31
Van13 (36 ms)	Arb17SD	For18	36.35	19.93	33.02	25.06	33.91	18.25	26.87	27.59	−2.44	−1.68
Mar04	Arb17SD	LinInt	36.62	20.46	31.59	27.60	34.40	19.01	25.36	30.02	−2.22	−1.45
For18	Pal21	LinInt	36.67	48.71	−12.69	59.64	32.59	21.53	−12.53	37.00	−4.08	−27.18
		For18	37.15	48.97	−12.04	60.28	33.02	21.89	−11.80	37.82	−4.13	−27.08
Van13 (34 ms)	Arb17SD	LinInt	37.16	20.33	34.22	24.97	34.45	18.94	28.10	27.50	−2.71	−1.39
Mar04	Arb17SD	For18	37.19	20.50	32.21	27.67	34.94	18.96	25.97	30.09	−2.25	−1.54
For18	Pal21	None	37.28	50.60	−11.52	61.78	32.56	21.78	−10.66	37.70	−4.72	−28.82
Van13 (38 ms)	Arb17SD	None	37.63	20.62	33.95	26.25	35.36	18.51	27.73	28.71	−2.27	−2.12
Van13 (34 ms)	Arb17SD	For18	37.84	20.34	34.92	25.01	35.09	18.89	28.79	27.56	−2.74	−1.45
Van13 (32 ms)	Arb17SD	LinInt	38.69	20.75	36.12	24.95	35.67	19.59	30.01	27.48	−3.02	−1.16
Van13 (36 ms)	Arb17SD	None	39.10	21.03	35.84	26.20	36.50	19.14	29.61	28.66	−2.60	−1.89
Van13 (32 ms)	Arb17SD	For18	39.37	20.76	36.83	24.98	36.31	19.54	30.70	27.54	−3.05	−1.22
Mar04	Arb17SD	None	39.70	21.41	34.88	28.61	37.20	19.67	28.53	30.94	−2.50	−1.74
Van13 (34 ms)	Arb17SD	None	40.49	21.41	37.63	26.12	37.59	19.76	31.41	28.58	−2.91	−1.65
Van13 (32 ms)	Arb17SD	None	41.96	21.82	39.46	26.07	38.74	20.45	33.23	28.55	−3.22	−1.37
	Pal21	LinInt	43.45	49.67	−20.65	62.68	39.44	24.83	−20.39	41.91	−4.01	−24.84
		None	43.72	51.61	−19.15	64.87	39.10	25.21	−18.21	42.82	−4.62	−26.40
		For18	43.74	49.92	−20.06	63.28	39.67	24.97	−19.72	42.53	−4.07	−24.95
Van13 (34 ms)	Pal21	LinInt	44.63	50.04	−22.02	63.33	40.63	25.19	−21.66	42.62	−3.99	−24.85
		None	44.79	51.98	−20.58	65.46	40.23	25.61	−19.49	43.52	−4.57	−26.37
		For18	44.86	50.28	−21.49	63.86	40.82	25.34	−21.05	43.19	−4.04	−24.94
Mar04	Pal21	None	45.12	52.02	−22.30	65.15	41.11	27.22	−21.67	44.30	−4.01	−24.80
		LinInt	45.23	50.11	−23.82	63.17	41.79	27.09	−23.96	43.66	−3.44	−23.02
		For18	45.33	50.35	−23.16	63.67	41.90	27.13	−23.16	44.22	−3.44	−23.22
Van13 (36 ms)	Pal21	LinInt	45.84	50.15	−23.21	63.86	41.93	25.53	−22.94	43.41	−3.90	−24.63
		None	45.94	52.09	−21.73	65.97	41.51	25.98	−20.66	44.41	−4.42	−26.11
		For18	46.01	50.38	−22.72	64.34	42.10	25.68	−22.29	43.99	−3.92	−24.70
Van13 (38 ms)	Pal21	LinInt	47.04	50.19	−24.50	64.28	43.28	25.71	−24.27	44.11	−3.76	−24.48
		None	47.13	52.13	−22.91	66.44	42.86	26.13	−21.87	45.19	−4.26	−26.00
		For18	47.21	50.43	−23.96	64.79	43.40	25.85	−23.59	44.67	−3.81	−24.58
For18	Arb17SD	LinInt	48.03	22.49	46.65	25.22	43.54	22.42	40.63	27.34	−4.49	−0.07

Continued on next page

Table S12: Error metrics of PEP extraction pipelines on the *EmpkinS Dataset* for different annotators and MAE difference between both annotators. MAE = Mean Absolute Error, ME = Mean Error.

			Annotator 1				Annotator 2				Ann. Diff.	
			MAE [ms]		ME [ms]		MAE [ms]		ME [ms]		MAE [ms]	
**Q-peak Algorithm**	**B-point Algorithm**	**Outlier Correction Algorithm**	**Mean**	**SD**	**Mean**	**SD**	**Mean**	**SD**	**Mean**	**SD**	**Mean**	**SD**
Van13 (40 ms)	Pal21	LinInt	48.40	50.22	−25.57	64.90	44.82	25.88	−25.33	45.14	−3.58	−24.35
		None	48.45	52.13	−23.98	67.00	44.36	26.13	−23.00	46.06	−4.09	−26.00
		For18	48.48	50.44	−25.19	65.28	44.84	25.91	−24.82	45.46	−3.64	−24.53
For18	Arb17SD	For18	48.60	22.52	47.23	25.27	44.08	22.47	41.20	27.40	−4.52	−0.05
Van13 (42 ms)	Pal21	LinInt	49.85	50.18	−26.51	65.58	46.48	26.07	−26.25	46.38	−3.37	−24.11
		For18	49.95	50.40	−26.02	66.02	46.52	26.13	−25.63	46.80	−3.42	−24.27
		None	49.98	52.06	−24.68	67.82	46.10	26.30	−23.71	47.49	−3.88	−25.75
For18	Arb17SD	None	50.70	23.55	49.33	26.29	45.93	23.51	43.13	28.32	−4.76	−0.04

3.5.2 Effect of Annotation Agreement on PEP Pipeline Detection Error

Table S13: Effect of annotation agreement on the absolute error (AE) of selected PEP pipeline [For18, Dro22, None] on the *EmpkinS Dataset*, using Q-peak annotations for agreement computation. Annotation agreements: *high*: [0 ms, 4 ms], *medium*: [5 ms, 10 ms], *low*: $\geq$ 11 ms.

	**Annotator 1**		**Annotator 2**	
	**Mean**	**SD**	**Mean**	**SD**
Agreement Bins				
high	9.68	13.71	10.34	14.05
medium	10.71	10.03	13.15	13.74
low	19.21	18.97	13.35	15.31

Table S14: Effect of annotation agreement on the absolute error (AE) of selected PEP pipeline [For18, Dro22, None] on the *EmpkinS Dataset*, using B-point annotations for agreement computation. Annotation agreements: *high*: [0 ms, 4 ms], *medium*: [5 ms, 10 ms], *low*: $\geq$ 11 ms.

	**Annotator 1**		**Annotator 2**	
	**Mean**	**SD**	**Mean**	**SD**
Agreement Bins				
high	8.32	12.46	7.43	10.59
medium	11.34	16.99	10.17	14.22
low	17.90	19.06	21.59	17.79

4 *Guardian Dataset*

4.1 Reference PEP Values for the *Guardian Dataset*

Table S15: Summary of reference pre-ejection period values for the different phases of the *Guardian Dataset*. The range is provided as [min, max].

Phase	M ± SD [ms]	Range [ms]
Pause	132.71 ± 25.41	[36, 208]
Valsalva	134.99 ± 25.28	[42, 200]
HoldingBreath	133.76 ± 30.32	[36, 292]
TiltUp	154.49 ± 22.92	[44, 250]
TiltLevel	134.16 ± 23.87	[48, 216]

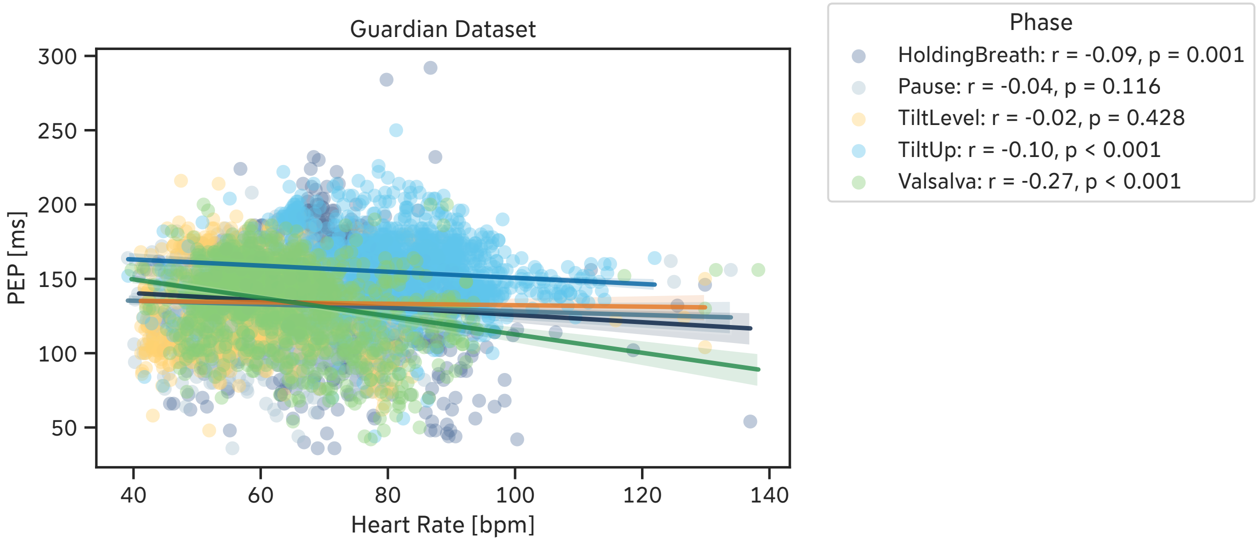

Figure S36: Relationship between Reference PEP and Heart Rate for the *Guardian Dataset*. The black line represents the linear regression fit with the corresponding 95% confidence interval.

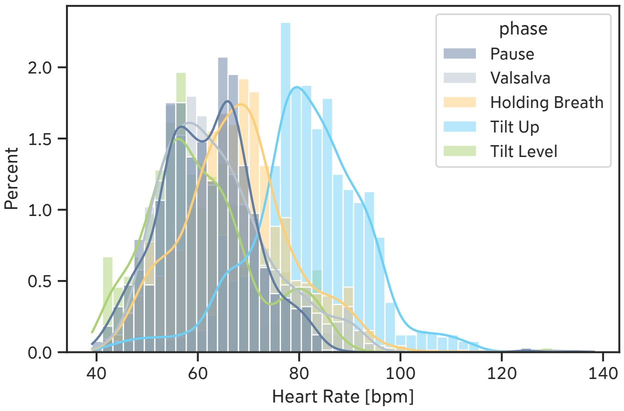

Figure S37: Distribution of heart rate values in the *Guardian Dataset*, divided by phase.

4.2 Q-Peak Detection

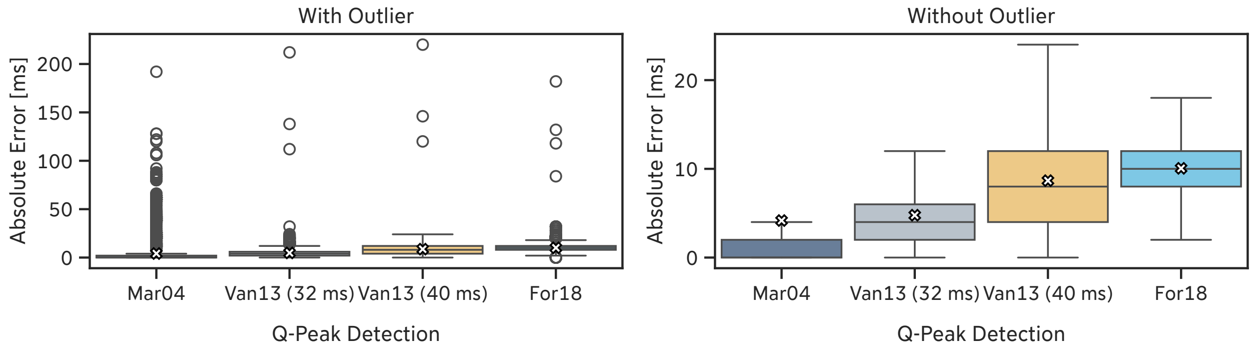

Figure S38: Absolute error of selected Q-peak extraction algorithms on the *Guardian Dataset* with outliers (left) and without outliers (right). Mean values are denoted by the white cross.

Table S16: Mean Abolute Error of selected Q-peak extraction algorithms on the *Guardian Dataset* per participant. The values with the highest errors are highlighted in red.

								Absolute Error [ms]
**Q-peak Algorithm**		**Mar04**	**Van13 (32 ms)**		**Van13 (40 ms)**			**For18**
	**Mean**	**SD**	**Mean**	**SD**	**Mean**	**SD**	**Mean**	**SD**
Participant								
GDN0005	4.99	10.84	7.46	1.98	2.26	4.72	14.21	3.74
GDN0006	1.25	4.48	5.78	1.03	2.29	1.37	13.99	2.25
GDN0007	1.03	1.08	0.79	1.03	7.28	1.08	11.03	1.21
GDN0008	5.45	7.88	4.67	1.34	3.41	1.11	9.73	2.14
GDN0009	45.69	24.99	10.07	1.56	17.33	4.10	6.20	4.40
GDN0010	0.07	0.37	3.26	1.73	11.15	1.93	9.89	1.46
GDN0011	1.21	0.98	0.58	1.02	7.73	1.14	8.37	0.84
GDN0012	1.00	2.69	3.80	1.02	11.75	1.18	7.85	1.03
GDN0013	0.88	0.99	1.08	1.35	8.17	1.73	11.09	1.07
GDN0014	0.48	0.93	0.98	1.31	8.81	1.43	9.33	1.04
GDN0016	0.82	1.00	2.59	1.13	5.41	1.13	10.82	1.19
GDN0017	0.09	0.41	3.32	1.27	11.32	1.27	8.60	0.93
GDN0018	0.90	1.01	14.24	0.80	22.24	0.80	5.65	1.16
GDN0019	0.61	0.92	4.21	0.97	12.21	0.97	8.92	1.01
GDN0020	7.44	9.64	7.50	6.80	13.43	8.56	9.34	6.73
GDN0021	9.44	17.56	12.83	1.27	20.82	1.38	5.17	1.43
GDN0022	15.24	20.29	6.18	3.16	2.62	2.59	11.71	8.80
GDN0023	5.01	11.03	5.10	4.48	4.89	2.65	10.88	2.78
GDN0024	0.99	1.02	1.17	1.09	6.94	1.20	11.61	0.99
GDN0025	1.43	16.17	8.85	17.13	2.14	18.37	21.83	13.53
GDN0027	1.20	0.98	0.79	0.99	7.21	0.99	9.60	0.86
GDN0028	1.21	8.03	4.63	8.95	4.58	9.48	11.37	7.19
GDN0029	0.19	1.30	2.97	1.52	5.10	1.66	11.14	1.18
GDN0030	0.13	0.50	6.48	1.08	1.52	1.08	10.58	1.17

4.2.1 Residual Plots – Overall and Detailed

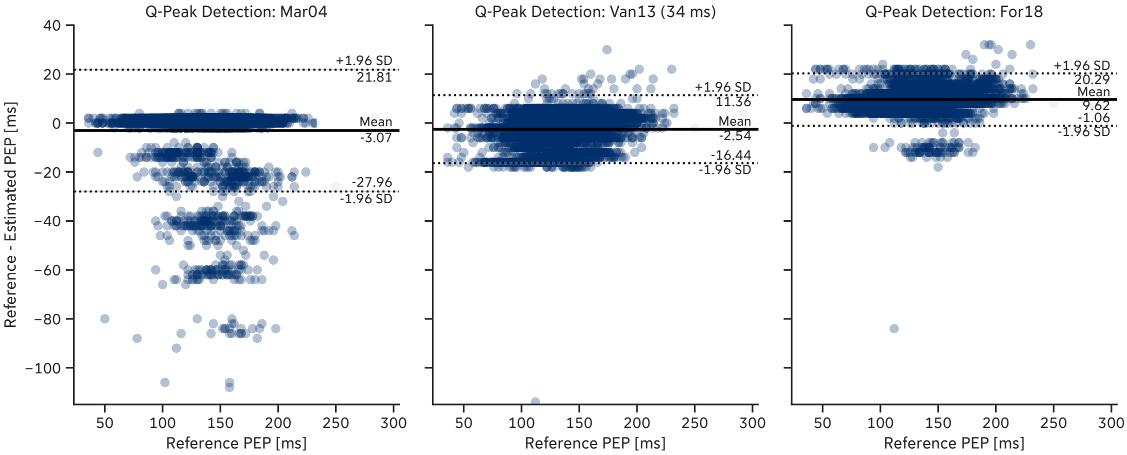

Figure S39: Residual plots of selected Q-peak extraction algorithms on the *Guardian Dataset*.

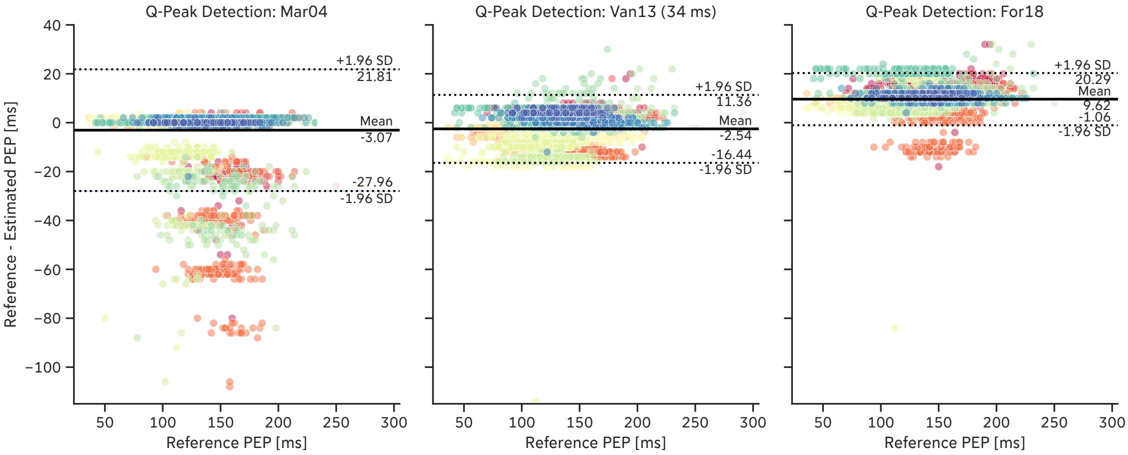

Figure S40: Residual plots of selected Q-peak extraction algorithms on the *Guardian Dataset*. Each participant is represented by a different color.

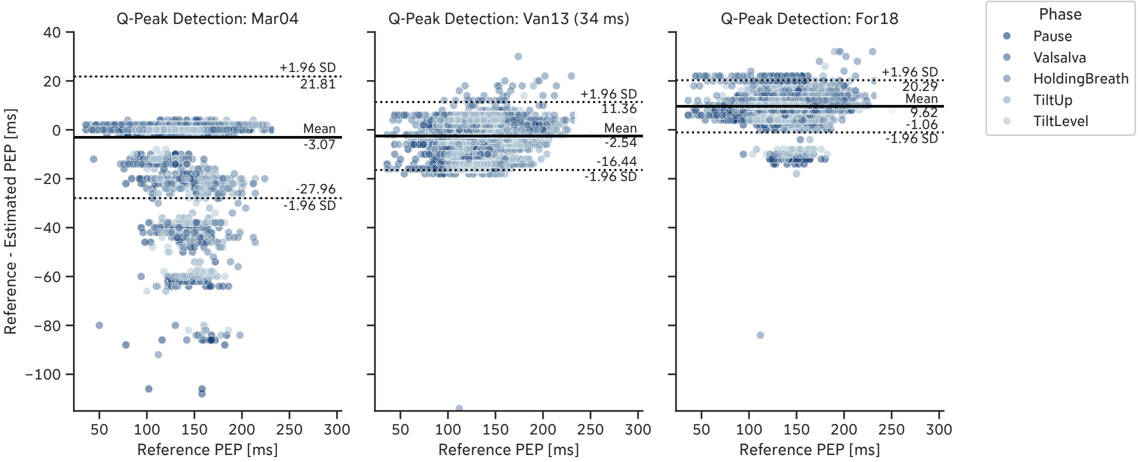

Figure S41: Residual plots of selected Q-peak extraction algorithms on the *Guardian Dataset*. Each phase is represented by a different color.

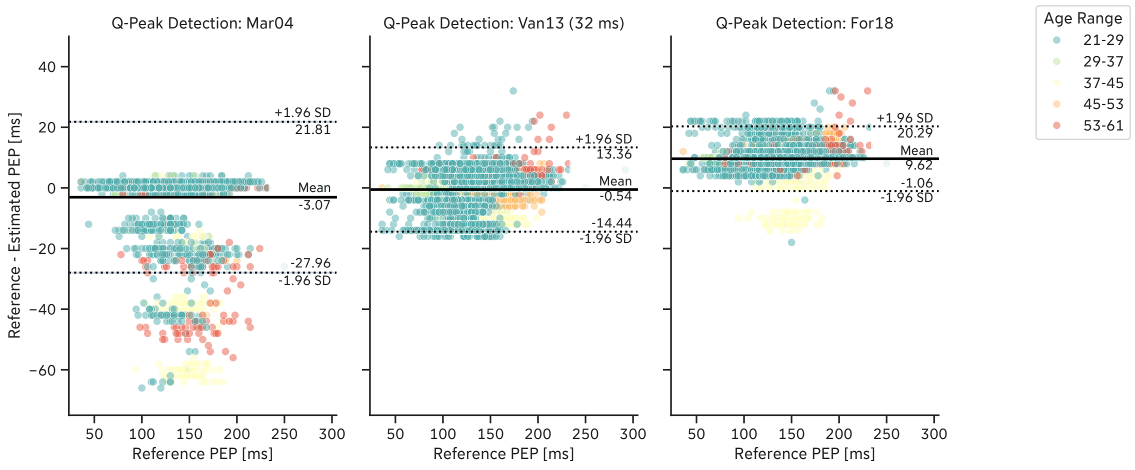

Figure S42: Residual plots of selected Q-peak extraction algorithms on the *Guardian Dataset*. Each age range is represented by a different color.

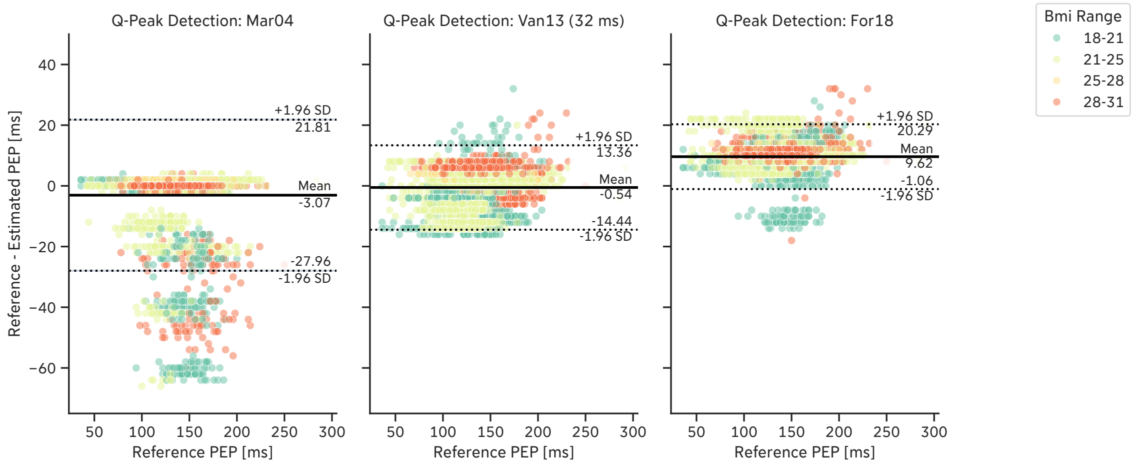

Figure S43: Residual plots of selected Q-peak extraction algorithms on the *Guardian Dataset*. Each BMI range is represented by a different color.

4.2.2 Effect of ECG Waveform on Q-Peak Detection Performance

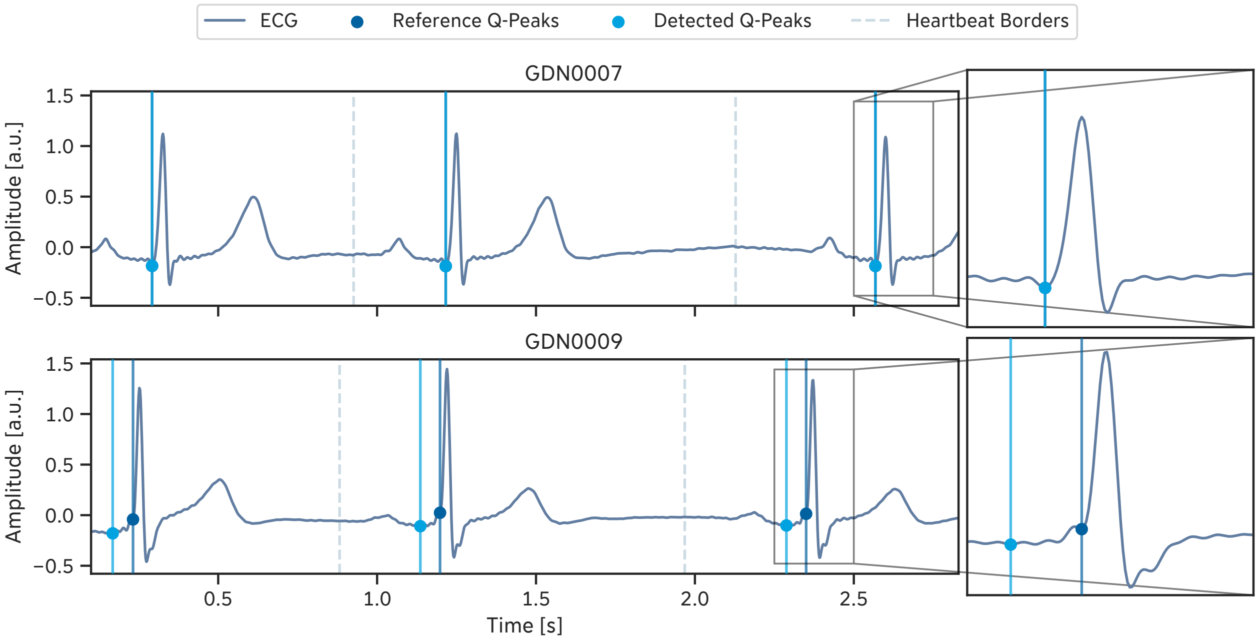

Figure S44: Examples of ECG waveforms from two participants of the *Guardian Dataset* and the effect of the ECG waveform on the Q-peak detection performance. The Q-peak extraction was performed using the *Mar04* algorithm [2].

4.2.3 Effect of Heart Rate on Q-Peak Detection Error

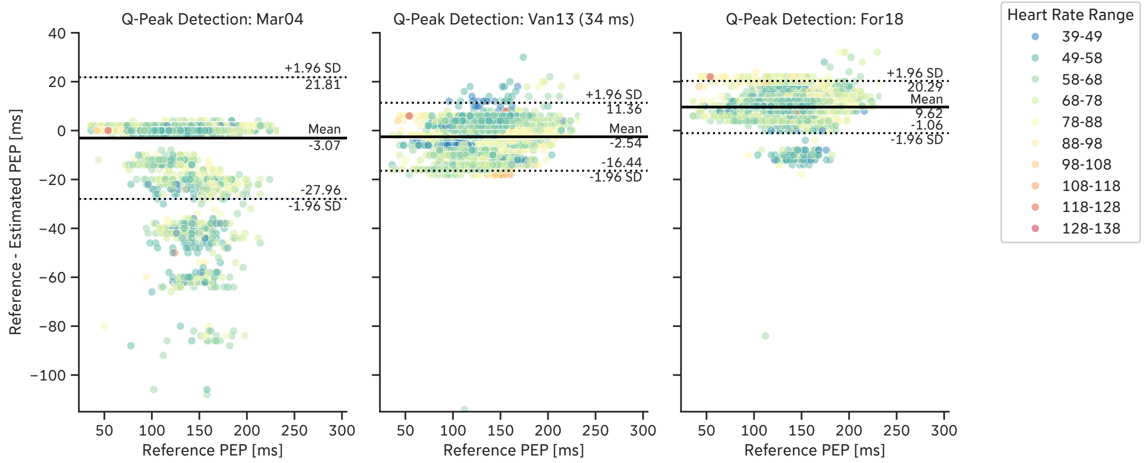

Figure S45: Residual plots of selected Q-peak extraction algorithms on the *Guardian Dataset*. Each heart rate bin is represented by a different color.

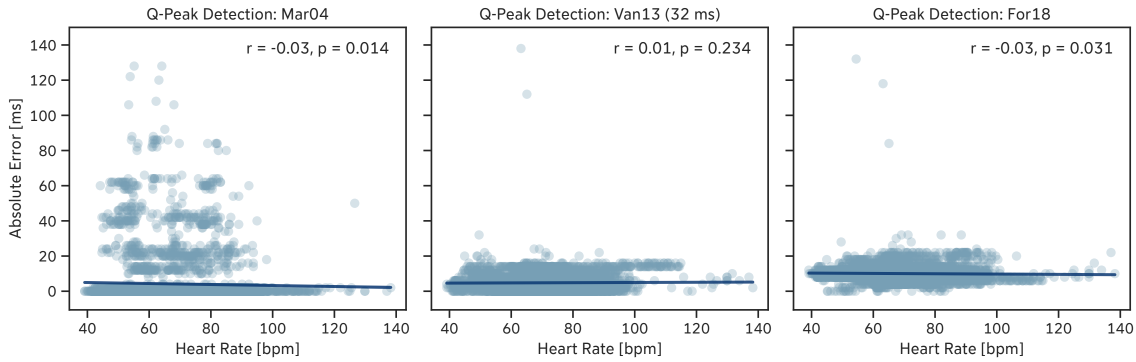

Figure S46: Regression plot between heart rate and absolute error of selected Q-peak extraction algorithms on the *Guardian Dataset*.

4.2.4 Effect of Annotations on Q-Peak Detection Error

Table S17: Error metrics of Q-peak extraction algorithms on the *Guardian Dataset* for different annotators and MAE difference between both annotators. MAE = Mean Absolute Error, ME = Mean Error.

	Annotator 1				Annotator 2				Annotator Difference	
	MAE [ms]		ME [ms]		MAE [ms]		ME [ms]		MAE [ms]	
Q-peak Algorithm	Mean	SD	Mean	SD	Mean	SD	Mean	SD	Mean	SD
Mar04	4.17	12.38	−3.07	12.70	5.11	12.69	−3.53	13.22	0.94	0.31
Van13 (32 ms)	4.77	5.27	−0.54	7.09	4.66	5.72	−0.97	7.32	−0.11	0.45
Van13 (34 ms)	4.96	5.67	−2.54	7.09	4.98	6.13	−2.97	7.32	0.01	0.46
Van13 (36 ms)	5.69	6.21	−4.54	7.09	5.90	6.59	−4.97	7.32	0.21	0.38
Van13 (38 ms)	6.96	6.68	−6.54	7.09	7.33	6.96	−6.97	7.32	0.37	0.27
Van13 (40 ms)	8.67	6.93	−8.54	7.09	9.11	7.14	−8.97	7.32	0.44	0.20
For18	10.06	4.58	9.62	5.45	9.71	4.76	9.18	5.71	−0.34	0.17
Van13 (42 ms)	10.63	6.96	−10.54	7.09	11.05	7.20	−10.97	7.32	0.42	0.24

4.2.5 Effect of Annotation Agreement on Q-Peak Detection Error

Table S18: Effect of annotation agreement on the absolute error (AE) of selected Q-peak extraction algorithm (Mar04) on the *Guardian Dataset*. Annotation agreements: *high*: [0 ms, 4 ms], *medium*: [5 ms, 10 ms], *low*: ≥ 11 ms

	Annotator 1		Annotator 2	
Agreement Bins	Mean	SD	Mean	SD
high	4.10	12.21	4.69	12.31
medium	6.51	10.61	9.16	9.39
low	3.76	9.46	16.67	10.29

4.3 B-Point Detection

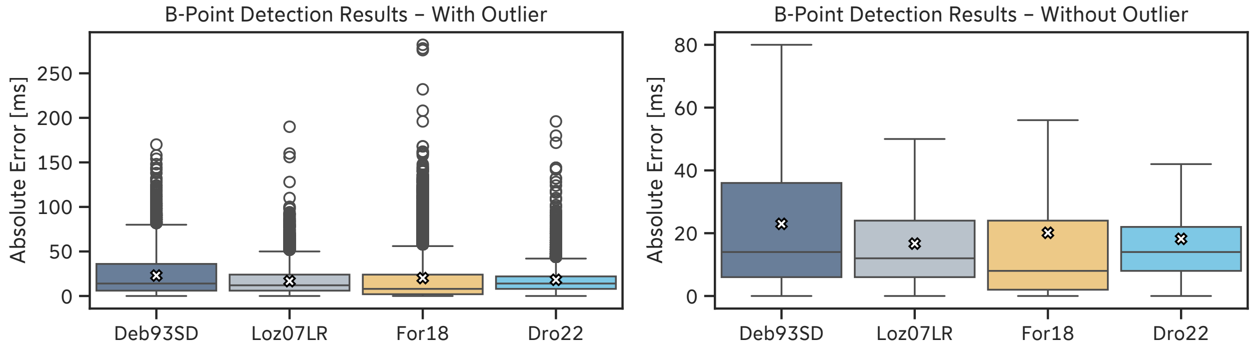

Figure S47: Absolute error of selected B-point extraction algorithms on the *Guardian Dataset* with outliers (left) and without outliers (right). Mean values are denoted by the white cross.

Table S19: Mean Abolute Error of selected B-point extraction algorithms on the *Guardian Dataset* per participant. The values with the highest errors are highlighted in red.

							Absolute Error [ms]	
**B-point Algorithm**		**Deb93SD**		**Dro22**		**For18**		**Loz07LR**
**Outlier Correction Algorithm**		**None**		**None**		**None**		**None**
	**Mean**	**SD**	**Mean**	**SD**	**Mean**	**SD**	**Mean**	**SD**
Participant								
GDN0005	18.87	20.18	16.85	14.04	22.13	27.32	17.03	13.54
GDN0006	10.28	10.75	13.30	8.18	16.78	19.96	13.59	8.58
GDN0007	18.22	19.78	17.93	11.27	31.04	42.81	12.89	11.68
GDN0008	21.17	21.01	13.75	9.63	18.05	22.98	12.85	10.19
GDN0009	24.67	20.83	15.15	11.76	17.70	22.96	14.27	10.96
GDN0010	30.51	29.31	21.77	18.44	25.65	33.14	19.32	17.05
GDN0011	24.06	22.47	16.67	11.70	18.35	32.50	13.60	11.58
GDN0012	28.46	29.38	18.42	15.43	18.49	22.19	17.66	15.92
GDN0013	23.68	20.42	12.99	11.20	10.84	21.28	10.68	9.60
GDN0014	17.37	21.27	19.37	13.60	52.32	45.35	19.72	16.36
GDN0016	28.32	26.64	20.22	13.97	22.33	30.41	19.76	15.22
GDN0017	19.81	16.91	11.85	11.34	10.91	15.79	18.58	11.14
GDN0018	24.21	28.08	21.31	17.46	14.88	24.54	23.54	17.30
GDN0019	19.59	16.29	18.88	10.91	9.35	16.39	17.93	12.26
GDN0020	18.24	20.68	23.11	14.83	19.40	33.11	19.08	14.77
GDN0021	28.74	26.16	14.63	10.35	21.75	34.82	11.25	9.36
GDN0022	25.60	26.52	20.00	17.42	19.22	26.10	21.60	16.87
GDN0023	23.19	20.43	17.64	10.68	9.13	12.67	15.32	9.72
GDN0024	12.33	17.71	17.96	15.60	25.17	31.63	11.54	15.38
GDN0025	24.81	29.00	30.68	22.33	31.39	35.67	26.58	24.31
GDN0027	27.13	27.69	21.39	22.99	15.42	18.45	18.14	20.44
GDN0028	33.62	29.40	25.00	18.96	23.72	27.34	23.46	15.83
GDN0029	31.28	26.42	15.41	19.40	11.62	15.59	13.85	16.58
GDN0030	20.75	23.40	22.27	20.60	23.28	25.54	17.11	14.05

4.3.1 Residual Plots – Overall and Detailed

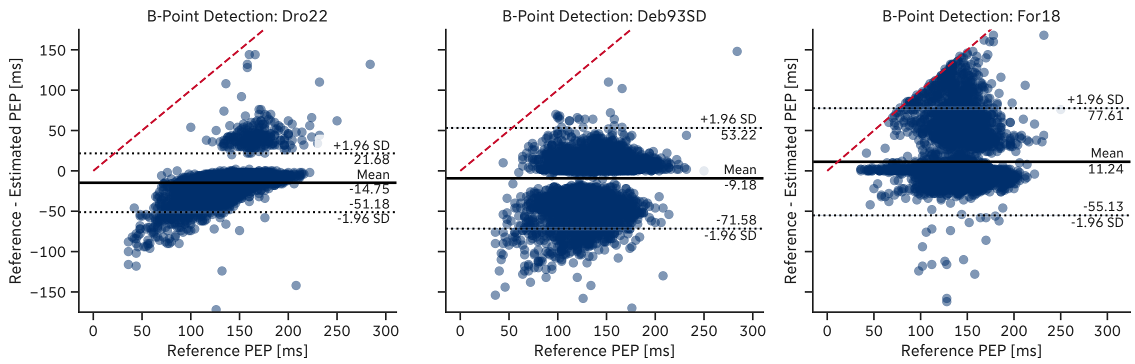

Figure S48: Residual plots of selected B-point extraction algorithms on the *Guardian Dataset*. The red dashed line indicates the upper estimation error limit given by the location of the reference Q-peaks.

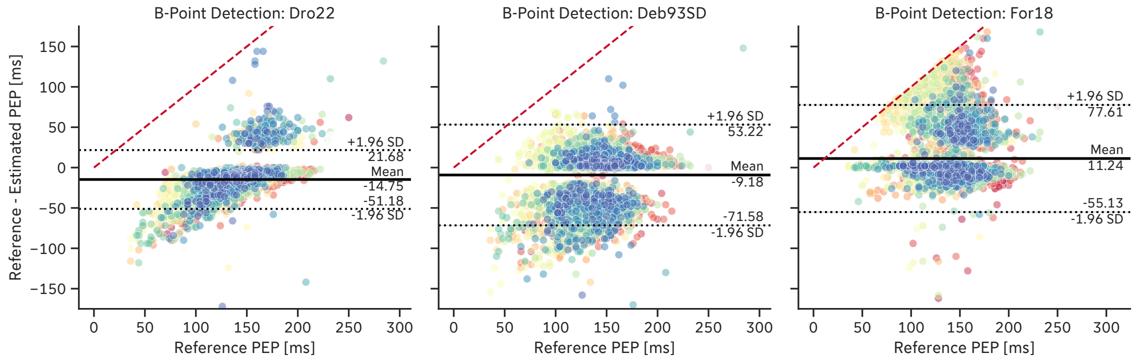

Figure S49: Residual plots of selected B-point extraction algorithms on the *Guardian Dataset*. Each participant is represented by a different color. The red dashed line indicates the upper estimation error limit given by the location of the reference Q-peaks.

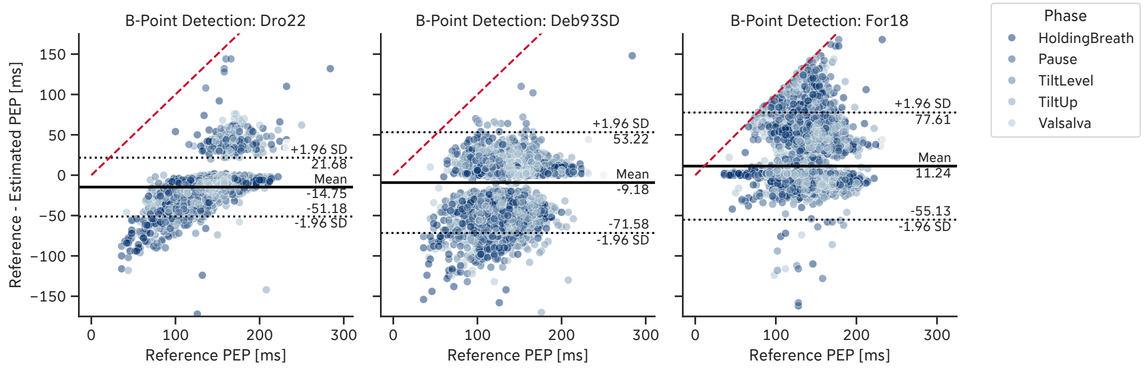

Figure S50: Residual plots of selected B-point extraction algorithms on the *Guardian Dataset*. Each phase is represented by a different color. The red dashed line indicates the upper estimation error limit given by the location of the reference Q-peaks.

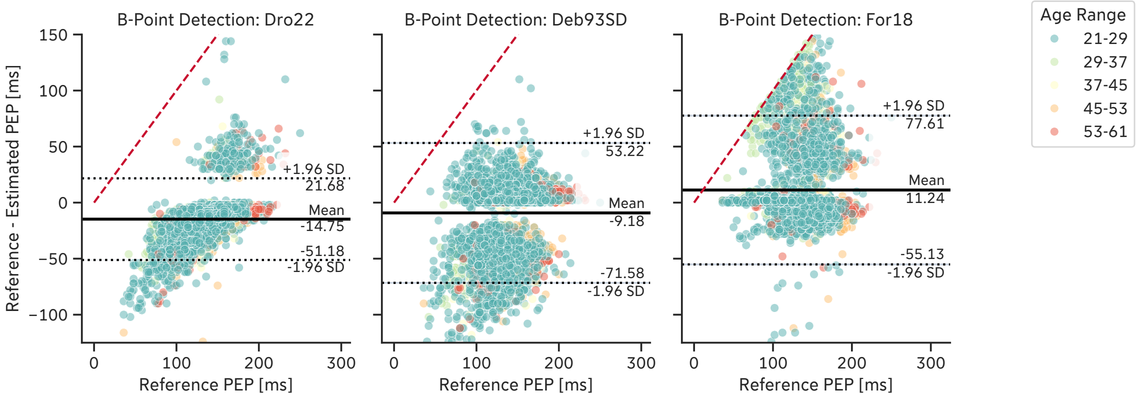

Figure S51: Residual plots of selected B-point extraction algorithms on the *Guardian Dataset*. Each age range is represented by a different color. The red dashed line indicates the upper estimation error limit given by the location of the reference Q-peaks.

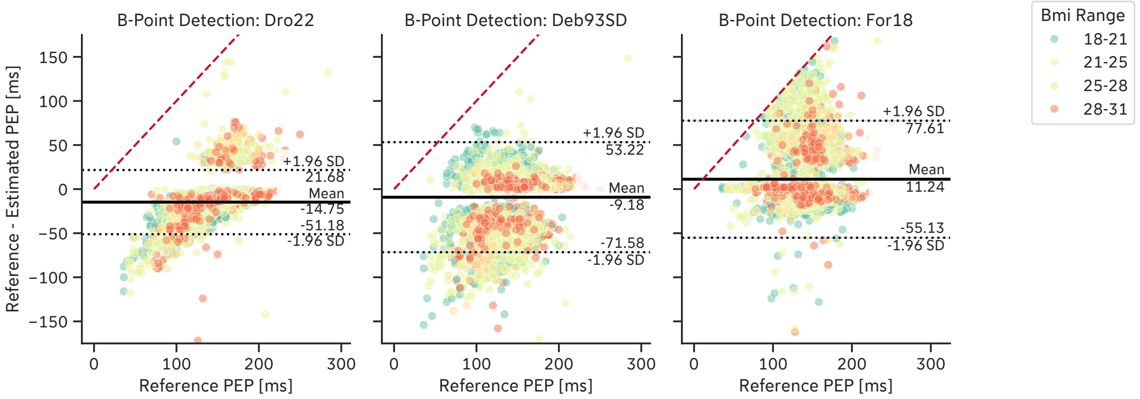

Figure S52: Residual plots of selected B-point extraction algorithms on the *Guardian Dataset*. Each BMI range is represented by a different color. The red dashed line indicates the upper estimation error limit given by the location of the reference Q-peaks.

4.3.2 Effect of Heart Rate on B-Point Detection Error

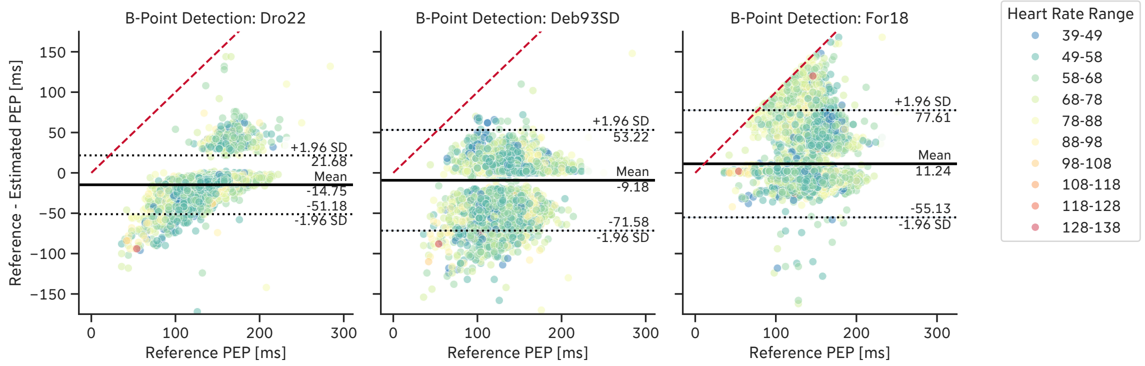

Figure S53: Residual plots of selected B-point extraction algorithms on the *Guardian Dataset*. Each heart rate bin is represented by a different color. The red dashed line indicates the upper estimation error limit given by the location of the reference Q-peaks.

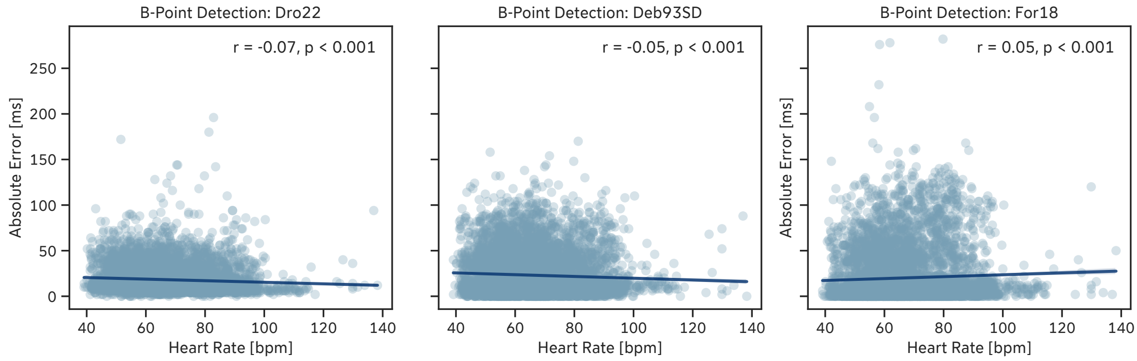

Figure S54: Regression plot between heart rate and absolute error of selected B-point extraction algorithms on the *Guardian Dataset*.

4.3.3 Effect of Outlier Correction on B-Point Detection Performance

Table S20: Effect of Outlier Correction algorithms on the B-point extraction algorithms for the *Guardian Dataset*. The algorithms are sorted by the Mean Absolute Error (MAE) in ascending order. Resuls highlighted in green indicate an improvement of the metric through outlier correction, red indicate no improvement.

		MAE [ms]		ME [ms]		MARE [%]		Valid PEPs	Invalid PEPs	Total PEPs
		Mean	SD	Mean	SD	Mean	SD	Total	Total	Total
B-Point Detection	Outlier Correction									
Loz07LR	None	16.68	14.77	−4.12	21.89	14.36	19.21	6232	379	6611
	For18	17.11	15.82	−4.90	22.79	14.77	20.24	6233	378	6611
	LinInt	17.17	15.88	−5.01	22.85	14.82	20.24	6233	378	6611
Ste85	LinInt	17.64	28.24	12.13	31.01	13.34	21.99	6155	456	6611
	For18	18.26	29.18	13.66	31.60	13.76	22.42	6149	462	6611
	None	21.53	33.68	18.65	35.35	15.95	25.23	6057	554	6611
Dro22	None	18.20	15.23	−14.75	18.59	15.47	19.00	6227	384	6611
	For18	19.15	16.05	−16.34	18.90	16.35	19.93	6230	381	6611
	LinInt	19.39	16.16	−16.78	18.85	16.58	20.17	6229	382	6611
For18	LinInt	18.34	26.14	5.87	31.39	13.77	20.98	6192	419	6611
	For18	18.45	26.38	6.93	31.44	13.80	20.84	6189	422	6611
	None	20.12	29.46	11.24	33.86	14.84	22.46	6039	572	6611
Mil22	LinInt	22.24	26.95	4.00	34.71	16.88	21.32	6229	382	6611
	For18	22.49	27.22	5.30	34.91	16.97	21.14	6229	382	6611
	None	24.28	29.71	9.42	37.19	17.91	22.01	6216	395	6611
Deb93SD	None	22.98	23.87	−9.18	31.84	19.13	24.99	6226	385	6611
	For18	23.58	24.11	−11.65	31.66	19.68	25.43	6228	383	6611
	LinInt	24.27	24.48	−12.92	31.96	20.29	26.07	6228	383	6611
Loz07QR	LinInt	26.38	18.30	18.69	26.10	19.05	15.09	6230	381	6611
	For18	26.43	18.29	18.92	25.99	19.07	15.03	6230	381	6611
	None	27.06	18.66	20.24	25.90	19.38	14.75	6228	383	6611
Arb17TD	For18	29.15	28.18	−6.99	39.94	23.43	25.88	5776	835	6611
	LinInt	29.48	27.89	−8.85	39.61	23.82	25.93	5806	805	6611
	None	33.98	34.63	1.27	48.50	26.66	28.95	5402	1209	6611
She90	For18	31.22	21.53	0.35	37.93	24.35	19.99	6230	381	6611
	LinInt	31.27	21.46	−0.74	37.91	24.44	20.13	6230	381	6611
	None	33.30	22.47	4.44	39.93	25.66	19.77	6187	424	6611
Arb17IC	LinInt	31.30	21.74	0.16	38.11	24.41	20.02	6230	381	6611
	For18	31.42	21.79	1.07	38.23	24.46	19.83	6230	381	6611
	None	32.85	22.52	4.19	39.61	25.34	19.69	6223	388	6611
Arb17SD	LinInt	41.33	22.14	36.06	29.98	30.12	15.73	6220	391	6611
	For18	41.72	21.98	36.51	29.86	30.41	15.59	6220	391	6611
	None	43.12	21.91	37.96	29.97	31.46	15.45	6219	392	6611
Pal21	LinInt	48.08	37.56	−2.17	60.97	38.10	32.93	4535	2076	6611
	For18	49.05	38.50	−0.28	62.36	38.62	33.04	4487	2124	6611
	None	50.65	40.50	3.64	64.75	39.59	33.56	4117	2494	6611

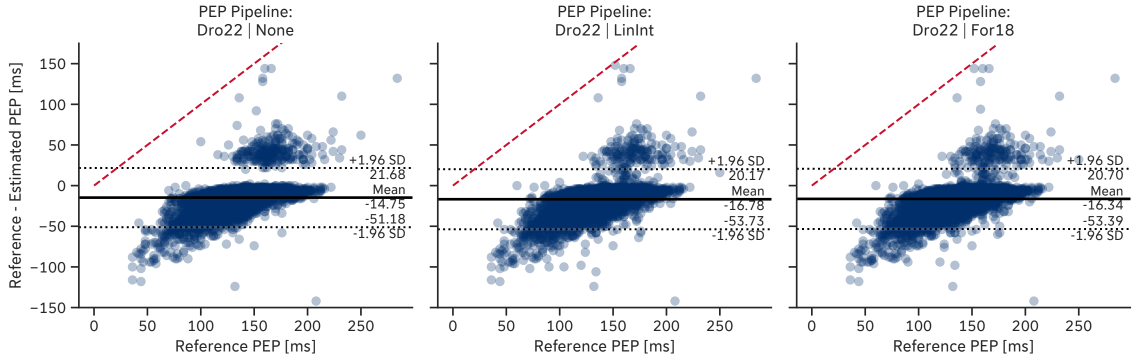

Figure S55: Effect of outlier correction (*LinInt*, *For18*) on the absolute error of the *Dro22* [3] B-point detection algorithm for on the *Guardian Dataset*. The red dashed line indicates the upper estimation error limit given by the location of the reference Q-peaks.

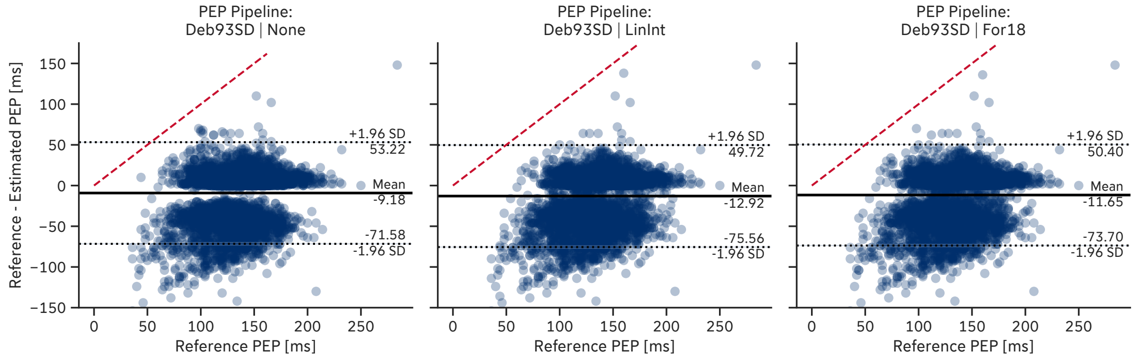

Figure S56: Effect of outlier correction (*LinInt*, *For18*) on the absolute error of the *Deb93SD* [4] B-point detection algorithm for on the *Guardian Dataset*. The red dashed line indicates the upper estimation error limit given by the location of the reference Q-peaks.

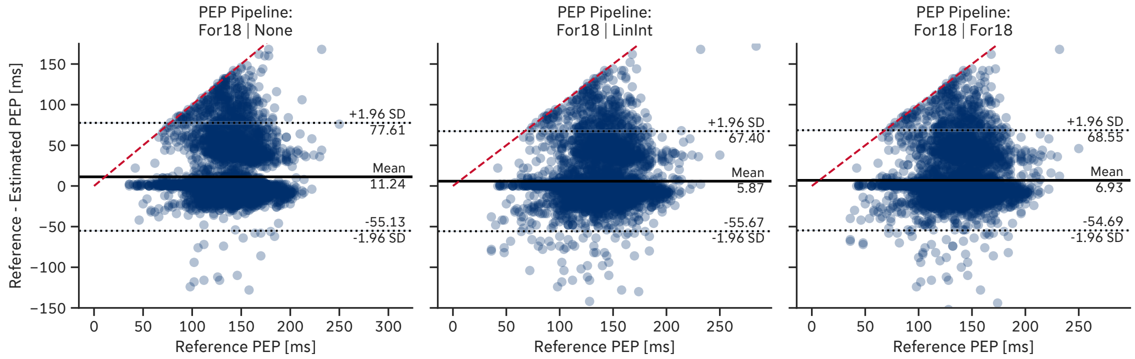

Figure S57: Effect of outlier correction (*LinInt*, *For18*) on the absolute error of the *For18* [5] B-point detection algorithm for on the *Guardian Dataset*. The red dashed line indicates the upper estimation error limit given by the location of the reference Q-peaks.

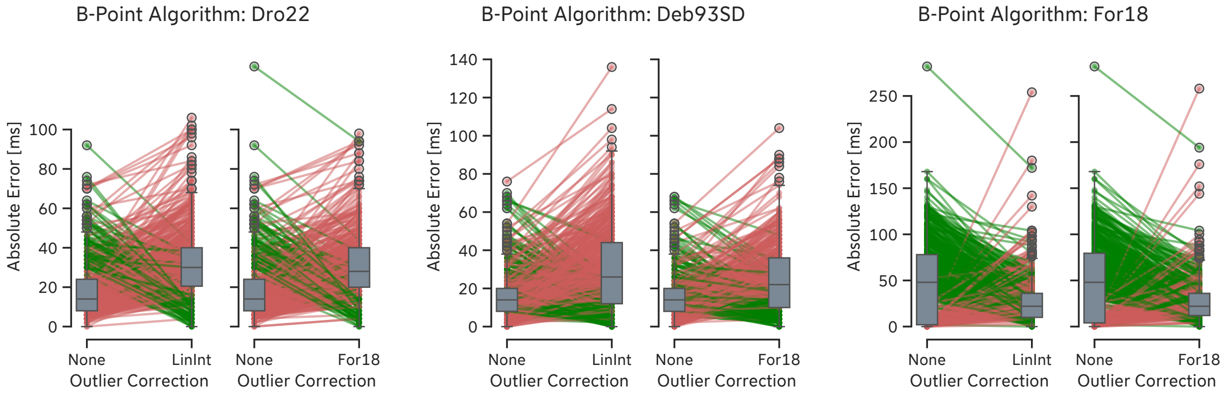

(a) *Dro22* [3]　(b) *Deb93SD* [4]　(c) *For18* [5]

Figure S58: Paired plots of the improvements in the absolute error due to outlier correction for the selected B-point detection algorithms on the *Guardian Dataset*. Green lines indicate an improvement (i.e., reduction in the absolute error) while red lines indicate a deterioration (i.e., increase in the absolute error).

4.3.4 Effect of Annotations on B-Point Detection Error

Table S21: Error metrics of B-point extraction algorithms on the *Guardian Dataset* for different annotators and MAE difference between both annotators. MAE = Mean Absolute Error, ME = Mean Error.

		Annotator 1				Annotator 2				Annotator Difference	
		MAE [ms]		ME [ms]		MAE [ms]		ME [ms]		MAE [ms]	
		Mean	SD	Mean	SD	Mean	SD	Mean	SD	Mean	SD
**B-point Algorithm**	**Outlier Correction Algorithm**										
Loz07LR	None	16.68	14.77	−4.12	21.89	18.93	17.62	−8.21	24.52	2.25	2.85
	For18	17.11	15.82	−4.90	22.79	19.39	18.54	−8.99	25.27	2.28	2.72
	LinInt	17.17	15.88	−5.01	22.85	19.46	18.60	−9.10	25.34	2.29	2.72
Ste85	LinInt	17.64	28.24	12.13	31.01	16.12	27.45	8.66	30.63	−1.52	−0.79
Dro22	None	18.20	15.23	−14.75	18.59	20.43	19.66	−18.82	21.20	2.23	4.43
Ste85	For18	18.26	29.18	13.66	31.60	16.57	28.28	10.18	31.16	−1.69	−0.90
For18	LinInt	18.34	26.14	5.87	31.39	18.22	25.61	2.37	31.34	−0.13	−0.53
	For18	18.45	26.38	6.93	31.44	18.12	25.66	3.50	31.21	−0.34	−0.72
Dro22	For18	19.15	16.05	−16.34	18.90	21.77	20.28	−20.45	21.61	2.62	4.23
	LinInt	19.39	16.16	−16.78	18.85	22.12	20.39	−20.91	21.63	2.73	4.23
For18	None	20.12	29.46	11.24	33.86	19.34	28.48	7.91	33.50	−0.79	−0.98
Ste85	None	21.53	33.68	18.65	35.35	18.97	32.50	15.24	34.41	−2.56	−1.17
Mil22	LinInt	22.24	26.95	4.00	34.71	21.48	27.39	0.07	34.81	−0.77	0.45
	For18	22.49	27.22	5.30	34.91	21.49	27.50	1.37	34.87	−1.00	0.28
Deb93SD	None	22.98	23.87	−9.18	31.84	28.03	25.38	−13.36	35.37	5.05	1.50
	For18	23.58	24.11	−11.65	31.66	28.41	25.68	−15.82	34.88	4.83	1.57
	LinInt	24.27	24.48	−12.92	31.96	29.09	25.98	−17.11	35.04	4.82	1.49
Mil22	None	24.28	29.71	9.42	37.19	22.59	29.73	5.60	36.92	−1.69	0.03
Loz07QR	LinInt	26.38	18.30	18.69	26.10	26.17	18.66	14.52	28.68	−0.20	0.36
	For18	26.43	18.29	18.92	25.99	26.21	18.66	14.76	28.59	−0.22	0.37
	None	27.06	18.66	20.24	25.90	26.74	18.87	16.09	28.50	−0.32	0.21
Arb17TD	For18	29.15	28.18	−6.99	39.94	31.05	29.53	−10.62	41.52	1.90	1.35
	LinInt	29.48	27.89	−8.85	39.61	31.54	29.38	−12.48	41.26	2.06	1.50
She90	For18	31.22	21.53	0.35	37.93	32.16	21.73	−3.49	38.65	0.93	0.20
	LinInt	31.27	21.46	−0.74	37.91	32.22	21.76	−4.62	38.60	0.95	0.31
Arb17IC	LinInt	31.30	21.74	0.16	38.11	32.23	21.90	−3.73	38.79	0.93	0.15
	For18	31.42	21.79	1.07	38.23	32.27	21.90	−2.79	38.90	0.85	0.11
	None	32.85	22.52	4.19	39.61	33.31	22.33	0.38	40.11	0.47	−0.20
She90	None	33.30	22.47	4.44	39.93	33.76	22.42	0.65	40.52	0.45	−0.05
Arb17TD	None	33.98	34.63	1.27	48.50	35.57	35.39	−2.35	50.12	1.59	0.76
Arb17SD	LinInt	41.33	22.14	36.06	29.98	40.14	21.72	32.00	32.54	−1.19	−0.42
	For18	41.72	21.98	36.51	29.86	40.49	21.54	32.45	32.42	−1.23	−0.44
	None	43.12	21.91	37.96	29.97	41.77	21.48	33.90	32.52	−1.34	−0.43
Pal21	LinInt	48.08	37.56	−2.17	60.97	49.14	37.14	−4.77	61.42	1.06	−0.42
	For18	49.05	38.50	−0.28	62.36	49.88	37.83	−2.90	62.54	0.83	−0.67
	None	50.65	40.50	3.64	64.75	50.98	39.52	1.24	64.50	0.33	−0.98

4.3.5 Effect of Annotation Agreement on B-Point Detection Error

Table S22: Effect of annotation agreement on the absolute error (AE) of selected B-point extraction algorithm (Dro22) on the *Guardian Dataset*. Annotation agreements: *high*: [0 ms, 4 ms], *medium*: [5 ms, 10 ms], *low*: $\geq$ 11 ms.

	**Annotator 1**		**Annotator 2**	
Agreement Bins	**Mean**	**SD**	**Mean**	**SD**
high	16.62	14.21	16.10	14.06
medium	23.74	15.73	19.16	15.13
low	21.09	17.95	42.58	27.72

4.4 Combined PEP Pipelines

4.4.1 Results of all PEP Pipelines

Table S23: Results of combined PEP extraction pipelines on the *Guardian Dataset*. The pipelines are sorted by the MAE in ascending order.

Q-Peak Detection	B-Point Detection	Outlier Correction	**MAE [ms]**	**ME [ms]**	**MARE [%]**	**Invalid PEPs**
For18	Dro22	None	11.6±15.9	−5.0±19.0	10.1±17.8	385 (5.8 %)
		For18	12.3±15.9	−6.6±19.0	10.9±18.4	384 (5.8 %)
		LinInt	12.5±16.2	−7.0±19.2	11.1±18.7	384 (5.8 %)
Van13 (32 ms)	Loz07LR	None	17.9±15.4	−4.5±23.2	15.4±20.2	378 (5.7 %)
Van13 (34 ms)	Loz07LR	None	18.1±15.9	−6.5±23.2	15.8±21.0	378 (5.7 %)
Mar04	Loz07LR	None	18.3±16.6	−6.5±23.8	15.7±20.6	481 (7.3 %)
Van13 (32 ms)	Loz07LR	For18	18.3±16.5	−5.3±24.1	15.8±21.3	377 (5.7 %)
		LinInt	18.4±16.6	−5.4±24.2	15.9±21.3	377 (5.7 %)
For18	Loz07LR	None	18.4±14.2	5.7±22.5	14.5±16.1	379 (5.7 %)
Van13 (36 ms)	Loz07LR	None	18.4±16.4	−8.5±23.2	16.3±21.7	378 (5.7 %)
Van13 (34 ms)	Loz07LR	For18	18.6±17.0	−7.3±24.1	16.2±22.1	377 (5.7 %)
For18	Loz07LR	For18	18.6±15.0	4.9±23.4	14.8±17.3	379 (5.7 %)
Van13 (34 ms)	Loz07LR	LinInt	18.6±17.1	−7.4±24.2	16.3±22.1	377 (5.7 %)
For18	Loz07LR	LinInt	18.6±15.0	4.8±23.4	14.8±17.2	379 (5.7 %)
Mar04	Loz07LR	For18	18.8±17.8	−7.3±24.8	16.1±21.8	480 (7.3 %)
		LinInt	18.8±17.9	−7.4±24.9	16.2±21.8	480 (7.3 %)
Van13 (32 ms)	Dro22	None	18.9±16.6	−15.1±20.0	16.3±20.5	383 (5.8 %)
Van13 (36 ms)	Loz07LR	For18	18.9±17.5	−9.3±24.1	16.7±22.8	377 (5.7 %)
Van13 (38 ms)	Loz07LR	None	19.0±17.0	−10.5±23.2	16.9±22.5	378 (5.7 %)
Van13 (36 ms)	Loz07LR	LinInt	19.0±17.6	−9.4±24.2	16.8±22.8	377 (5.7 %)
Van13 (38 ms)	Loz07LR	For18	19.5±18.2	−11.3±24.2	17.4±23.6	376 (5.7 %)
		LinInt	19.6±18.2	−11.4±24.2	17.4±23.6	376 (5.7 %)
Van13 (40 ms)	Loz07LR	None	19.6±17.6	−12.5±23.2	17.6±23.2	378 (5.7 %)
Van13 (32 ms)	Dro22	For18	19.8±17.1	−16.8±20.1	17.1±21.3	381 (5.8 %)
Mar04	Ste85	LinInt	20.0±28.8	9.6±33.7	15.1±22.6	552 (8.3 %)
Van13 (32 ms)	Dro22	LinInt	20.1±17.4	−17.2±20.2	17.4±21.5	381 (5.8 %)
Van13 (36 ms)	Ste85	LinInt	20.1±27.4	8.2±33.0	15.5±21.7	430 (6.5 %)
Van13 (34 ms)	Ste85	LinInt	20.2±27.7	10.1±32.8	15.5±21.9	437 (6.6 %)
Van13 (40 ms)	Loz07LR	For18	20.2±18.7	−13.3±24.2	18.1±24.3	376 (5.7 %)
		LinInt	20.3±18.8	−13.4±24.2	18.2±24.3	376 (5.7 %)
Van13 (32 ms)	For18	LinInt	20.3±26.2	5.7±32.7	15.5±21.4	408 (6.2 %)
Mar04	For18	LinInt	20.3±26.8	3.3±33.5	15.3±21.7	522 (7.9 %)
Van13 (32 ms)	For18	For18	20.4±26.4	6.7±32.7	15.5±21.1	411 (6.2 %)
Mar04	Dro22	None	20.4±18.3	−17.2±21.4	17.1±20.9	486 (7.4 %)
	For18	For18	20.4±26.9	4.4±33.5	15.3±21.4	525 (7.9 %)
Van13 (38 ms)	Ste85	LinInt	20.5±26.9	6.4±33.2	15.8±21.5	422 (6.4 %)
Van13 (42 ms)	Loz07LR	None	20.5±18.1	−14.5±23.2	18.4±23.9	378 (5.7 %)
Van13 (34 ms)	For18	LinInt	20.5±25.7	3.7±32.7	15.7±21.2	407 (6.2 %)
Mar04	Ste85	For18	20.5±29.6	11.2±34.2	15.5±22.9	557 (8.4 %)
Van13 (34 ms)	For18	For18	20.5±25.9	4.7±32.7	15.6±20.8	410 (6.2 %)
Van13 (36 ms)	Ste85	For18	20.6±28.3	9.8±33.6	15.8±22.1	433 (6.5 %)
Van13 (32 ms)	Ste85	LinInt	20.6±27.9	12.0±32.6	15.7±22.0	443 (6.7 %)
Van13 (34 ms)	Dro22	None	20.6±16.4	−17.1±20.0	17.7±20.8	383 (5.8 %)
	Ste85	For18	20.7±28.6	11.7±33.4	15.8±22.3	440 (6.7 %)
Van13 (38 ms)	Ste85	For18	20.9±27.7	7.9±33.8	16.1±21.8	425 (6.4 %)
Van13 (36 ms)	For18	For18	21.1±25.4	2.9±32.9	16.1±20.7	403 (6.1 %)
		LinInt	21.1±25.3	1.8±32.9	16.1±21.0	401 (6.1 %)
Van13 (42 ms)	Loz07LR	For18	21.1±19.3	−15.3±24.2	18.9±25.0	376 (5.7 %)
Van13 (40 ms)	Ste85	LinInt	21.2±26.4	4.6±33.5	16.4±21.3	410 (6.2 %)
For18	For18	LinInt	21.2±27.0	15.0±30.9	16.1±21.1	461 (7.0 %)
Van13 (42 ms)	Loz07LR	LinInt	21.2±19.3	−15.4±24.2	19.0±25.0	376 (5.7 %)

Continued on next page

Table S23: Results of combined PEP extraction pipelines on the *Guardian Dataset*. The pipelines are sorted by the MAE in ascending order.

Q-Peak Detection	B-Point Detection	Outlier Correction	MAE [ms]	ME [ms]	MARE [%]	Invalid PEPs
Van13 (32 ms)	Ste85	For18	21.2±28.9	13.6±33.2	16.1±22.4	447 (6.8 %)
Mar04	Dro22	For18	21.4±18.9	−18.8±21.5	18.1±21.9	484 (7.3 %)
Van13 (40 ms)	Ste85	For18	21.5±27.1	6.1±34.1	16.6±21.5	413 (6.2 %)
For18	For18	For18	21.5±27.3	16.0±30.9	16.3±21.0	467 (7.1 %)
Van13 (34 ms)	Dro22	For18	21.6±17.2	−18.7±20.3	18.6±21.6	380 (5.7 %)
Mar04	Dro22	LinInt	21.7±19.2	−19.2±21.7	18.3±22.1	484 (7.3 %)
Van13 (38 ms)	For18	For18	21.7±24.8	0.9±33.0	16.6±20.4	400 (6.1 %)
		LinInt	21.8±24.7	−0.2±33.0	16.7±20.8	399 (6.0 %)
Van13 (34 ms)	Dro22	LinInt	21.9±17.3	−19.2±20.2	18.8±21.9	381 (5.8 %)
Mar04	For18	None	21.9±29.7	9.1±35.7	16.2±22.8	670 (10.1 %)
Van13 (42 ms)	Ste85	LinInt	22.0±25.5	2.6±33.6	17.1±20.9	406 (6.1 %)
		For18	22.3±26.2	4.2±34.1	17.2±21.0	408 (6.2 %)
Van13 (32 ms)	For18	None	22.3±29.4	11.3±35.1	16.7±22.4	551 (8.3 %)
Van13 (34 ms)	For18	None	22.4±28.7	9.4±35.2	16.8±22.0	545 (8.2 %)
Van13 (36 ms)	Dro22	None	22.5±16.2	−19.1±20.0	19.1±21.1	383 (5.8 %)
Van13 (40 ms)	For18	For18	22.6±24.2	−1.0±33.1	17.3±20.1	396 (6.0 %)
		LinInt	22.7±24.1	−2.1±33.1	17.4±20.5	395 (6.0 %)
Van13 (42 ms)	Loz07QR	For18	22.8±17.6	8.5±27.5	17.6±18.4	379 (5.7 %)
		LinInt	22.8±17.6	8.3±27.6	17.6±18.4	379 (5.7 %)
Van13 (36 ms)	For18	None	22.8±28.1	7.5±35.4	17.2±21.7	537 (8.1 %)
For18	Mil22	LinInt	23.0±29.1	13.5±34.5	17.0±21.6	395 (6.0 %)
Van13 (42 ms)	Loz07QR	None	23.1±17.5	9.9±27.2	17.6±17.6	379 (5.7 %)
Van13 (38 ms)	For18	None	23.4±27.2	5.6±35.5	17.6±21.1	534 (8.1 %)
Van13 (40 ms)	Loz07QR	LinInt	23.4±17.8	10.3±27.6	17.8±17.9	379 (5.7 %)
		For18	23.4±17.8	10.5±27.5	17.8±17.8	379 (5.7 %)
Van13 (36 ms)	Dro22	For18	23.5±17.1	−20.7±20.3	20.0±22.0	380 (5.7 %)
For18	Mil22	For18	23.5±29.5	14.8±34.7	17.3±21.6	395 (6.0 %)
Van13 (32 ms)	Mil22	LinInt	23.5±26.8	3.6±35.5	18.0±21.4	382 (5.8 %)
Van13 (36 ms)	Deb93SD	None	23.6±26.4	−13.6±32.7	19.7±27.7	385 (5.8 %)
Van13 (42 ms)	For18	For18	23.6±23.5	−3.0±33.1	18.0±19.7	394 (6.0 %)
Mar04	Ste85	None	23.6±33.5	16.3±37.6	17.5±25.3	643 (9.7 %)
For18	For18	None	23.6±30.4	20.2±32.8	17.8±22.8	630 (9.5 %)
Van13 (34 ms)	Deb93SD	None	23.7±25.3	−11.6±32.7	19.7±26.8	385 (5.8 %)
Van13 (38 ms)	Deb93SD	None	23.7±27.4	−15.6±32.7	19.9±28.5	385 (5.8 %)
Van13 (32 ms)	Mil22	For18	23.7±27.0	4.9±35.6	18.1±21.0	382 (5.8 %)
Van13 (42 ms)	For18	LinInt	23.7±23.5	−4.1±33.1	18.2±20.2	394 (6.0 %)
Van13 (36 ms)	Dro22	LinInt	23.7±17.4	−21.1±20.5	20.2±22.2	380 (5.7 %)
Van13 (40 ms)	Loz07QR	None	23.8±17.8	11.9±27.2	17.9±17.1	379 (5.7 %)
Mar04	Mil22	LinInt	23.9±27.7	1.5±36.6	18.1±22.3	484 (7.3 %)
Van13 (32 ms)	Deb93SD	None	23.9±24.2	−9.6±32.7	19.8±25.9	385 (5.8 %)
Van13 (36 ms)	Ste85	None	24.0±32.4	15.1±37.3	18.1±24.5	506 (7.7 %)
Van13 (40 ms)	Deb93SD	None	24.0±28.3	−17.6±32.7	20.3±29.3	385 (5.8 %)
For18	Arb17TD	LinInt	24.0±29.1	−0.2±37.7	19.6±25.6	859 (13.0 %)
Mar04	Mil22	For18	24.1±27.9	2.8±36.7	18.2±22.0	484 (7.3 %)
Van13 (34 ms)	Mil22	LinInt	24.1±26.2	1.6±35.6	18.6±21.3	380 (5.7 %)
For18	Arb17TD	For18	24.1±29.6	1.7±38.2	19.5±25.7	889 (13.4 %)
Van13 (34 ms)	Ste85	None	24.1±32.9	16.9±37.2	18.1±24.8	519 (7.9 %)
Van13 (38 ms)	Loz07QR	LinInt	24.2±18.1	12.3±27.6	18.1±17.4	379 (5.7 %)
		For18	24.2±18.1	12.5±27.5	18.1±17.3	379 (5.7 %)
	Ste85	None	24.2±31.8	13.3±37.7	18.4±24.1	491 (7.4 %)
Van13 (34 ms)	Mil22	For18	24.2±26.3	2.9±35.7	18.5±20.9	380 (5.7 %)
Van13 (40 ms)	For18	None	24.3±26.5	3.7±35.8	18.3±20.7	523 (7.9 %)
Van13 (38 ms)	Dro22	None	24.3±16.1	−21.1±20.1	20.5±21.4	382 (5.8 %)
Van13 (34 ms)	Deb93SD	For18	24.4±25.6	−14.1±32.4	20.4±27.2	383 (5.8 %)
Van13 (32 ms)	Deb93SD	For18	24.4±24.4	−12.1±32.4	20.3±26.3	383 (5.8 %)

Continued on next page

Table S23: Results of combined PEP extraction pipelines on the *Guardian Dataset*. The pipelines are sorted by the MAE in ascending order.

Q-Peak Detection	B-Point Detection	Outlier Correction	**MAE [ms]**	**ME [ms]**	**MARE [%]**	**Invalid PEPs**
Van13 (36 ms)	Deb93SD	For18	24.5±26.6	−16.1±32.4	20.6±28.1	383 (5.8 %)
Van13 (38 ms)	Loz07QR	None	24.6±18.2	13.9±27.2	18.3±16.6	379 (5.7 %)
Van13 (42 ms)	Deb93SD	None	24.6±29.1	−19.6±32.7	20.8±30.0	385 (5.8 %)
Van13 (32 ms)	Ste85	None	24.7±33.2	18.7±36.9	18.4±24.9	533 (8.1 %)
Van13 (38 ms)	Deb93SD	For18	24.8±27.6	−18.1±32.4	20.9±28.9	383 (5.8 %)
Mar04	Deb93SD	None	24.9±25.8	−11.6±33.9	20.5±26.5	488 (7.4 %)
Van13 (36 ms)	Mil22	LinInt	24.9±25.5	−0.4±35.6	19.2±21.1	380 (5.7 %)
Van13 (40 ms)	Ste85	None	24.9±31.2	11.7±38.1	19.0±23.8	467 (7.1 %)
Van13 (36 ms)	Mil22	For18	24.9±25.5	0.9±35.7	19.2±20.7	380 (5.7 %)
	Loz07QR	LinInt	25.0±18.5	14.3±27.6	18.5±16.9	379 (5.7 %)
		For18	25.0±18.5	14.5±27.5	18.5±16.9	379 (5.7 %)
Van13 (42 ms)	For18	None	25.2±25.5	1.8±35.8	19.0±20.0	521 (7.9 %)
Van13 (32 ms)	Deb93SD	LinInt	25.2±24.8	−13.3±32.8	21.0±27.0	383 (5.8 %)
Van13 (34 ms)	Deb93SD	LinInt	25.2±25.9	−15.3±32.8	21.2±27.9	383 (5.8 %)
Van13 (38 ms)	Dro22	For18	25.3±17.0	−22.7±20.4	21.4±22.3	379 (5.7 %)
Van13 (40 ms)	Deb93SD	For18	25.3±28.4	−20.1±32.4	21.4±29.7	383 (5.8 %)
Van13 (36 ms)	Deb93SD	LinInt	25.4±27.0	−17.3±32.8	21.4±28.8	383 (5.8 %)
	Loz07QR	None	25.5±18.6	15.9±27.2	18.7±16.2	379 (5.7 %)
For18	Ste85	LinInt	25.6±26.9	21.1±30.5	19.1±20.7	511 (7.7 %)
Van13 (32 ms)	Mil22	None	25.6±29.5	9.1±38.0	19.1±21.8	390 (5.9 %)
Van13 (42 ms)	Ste85	None	25.6±30.1	9.9±38.3	19.5±23.2	458 (6.9 %)
Mar04	Deb93SD	For18	25.6±26.1	−14.2±33.8	21.2±27.0	486 (7.4 %)
Van13 (38 ms)	Dro22	LinInt	25.6±17.3	−23.1±20.6	21.7±22.5	379 (5.7 %)
Mar04	Mil22	None	25.6±30.0	7.0±38.9	19.0±22.7	496 (7.5 %)
Van13 (38 ms)	Mil22	For18	25.8±24.6	−1.1±35.7	19.9±20.4	380 (5.7 %)
		LinInt	25.8±24.6	−2.4±35.6	20.0±20.9	380 (5.7 %)
	Deb93SD	LinInt	25.8±28.1	−19.3±32.9	21.9±29.7	382 (5.8 %)
For18	Mil22	None	25.9±32.5	18.9±37.0	18.8±23.1	410 (6.2 %)
Van13 (34 ms)	Loz07QR	LinInt	25.9±18.8	16.3±27.6	19.0±16.5	379 (5.7 %)
		For18	26.0±18.8	16.5±27.5	19.0±16.5	379 (5.7 %)
	Mil22	None	26.0±28.7	7.1±38.1	19.5±21.5	388 (5.9 %)
Mar04	Loz07QR	LinInt	26.2±18.2	16.2±27.5	19.1±16.1	484 (7.3 %)
Van13 (40 ms)	Dro22	None	26.2±16.0	−23.1±20.1	22.0±21.7	382 (5.8 %)
Van13 (42 ms)	Deb93SD	For18	26.2±29.3	−22.0±32.5	22.1±30.4	382 (5.8 %)
Mar04	Loz07QR	For18	26.2±18.2	16.5±27.4	19.2±16.0	484 (7.3 %)
For18	Ste85	For18	26.3±27.5	22.5±30.7	19.6±21.1	526 (8.0 %)
Mar04	Deb93SD	LinInt	26.4±26.5	−15.5±34.0	21.8±27.6	486 (7.4 %)
Van13 (40 ms)	Deb93SD	LinInt	26.4±29.0	−21.3±32.9	22.4±30.5	382 (5.8 %)
Van13 (34 ms)	Loz07QR	None	26.5±19.0	17.9±27.2	19.2±15.8	379 (5.7 %)
For18	Deb93SD	For18	26.5±18.4	−1.8±32.2	21.3±21.1	383 (5.8 %)
Van13 (36 ms)	Mil22	None	26.6±27.7	5.1±38.1	20.0±21.0	388 (5.9 %)
Mar04	Loz07QR	None	26.7±18.4	17.8±27.1	19.4±15.5	484 (7.3 %)
Van13 (40 ms)	Mil22	For18	26.8±23.8	−3.1±35.7	20.7±20.2	380 (5.7 %)
		LinInt	26.8±23.8	−4.4±35.6	20.9±20.7	380 (5.7 %)
For18	Deb93SD	LinInt	26.9±18.7	−3.1±32.6	21.6±21.6	383 (5.8 %)
Van13 (32 ms)	Loz07QR	LinInt	26.9±19.1	18.3±27.4	19.5±16.1	381 (5.8 %)
		For18	26.9±19.1	18.5±27.3	19.5±16.0	381 (5.8 %)
For18	Deb93SD	None	26.9±18.1	0.6±32.4	21.6±20.6	385 (5.8 %)
Van13 (40 ms)	Dro22	For18	27.2±16.9	−24.7±20.4	22.9±22.6	379 (5.7 %)
Van13 (42 ms)	Deb93SD	LinInt	27.3±29.7	−23.3±32.9	23.1±31.2	382 (5.8 %)
Van13 (38 ms)	Mil22	None	27.4±26.7	3.2±38.1	20.7±20.6	386 (5.8 %)
Van13 (40 ms)	Dro22	LinInt	27.5±17.2	−25.1±20.6	23.2±22.9	379 (5.7 %)
Van13 (32 ms)	Loz07QR	None	27.5±19.3	19.9±27.1	19.7±15.4	381 (5.8 %)
Van13 (42 ms)	Mil22	For18	27.7±23.0	−5.1±35.7	21.5±20.1	380 (5.7 %)
		LinInt	27.9±23.0	−6.4±35.6	21.7±20.6	380 (5.7 %)

Continued on next page

Table S23: Results of combined PEP extraction pipelines on the *Guardian Dataset*. The pipelines are sorted by the MAE in ascending order.

Q-Peak Detection	B-Point Detection	Outlier Correction	MAE [ms]	ME [ms]	MARE [%]	Invalid PEPs
	Dro22	None	28.0±15.9	−25.1±20.1	23.4±22.0	382 (5.8 %)
Van13 (40 ms)	Mil22	None	28.2±25.6	1.2±38.1	21.4±20.2	386 (5.8 %)
For18	Arb17TD	None	28.5±36.4	8.6±45.4	22.5±29.1	1320 (20.0 %)
	She90	LinInt	28.8±26.0	9.1±37.7	22.0±21.1	382 (5.8 %)
	Arb17IC	LinInt	28.9±26.3	9.9±37.8	22.1±21.1	383 (5.8 %)
	She90	For18	29.0±26.1	10.1±37.7	22.1±21.0	382 (5.8 %)
Van13 (42 ms)	Dro22	For18	29.1±16.9	−26.7±20.4	24.3±23.0	379 (5.7 %)
	Mil22	None	29.1±24.6	−0.8±38.1	22.2±19.8	386 (5.8 %)
For18	Arb17IC	For18	29.2±26.4	10.8±37.9	22.3±21.0	383 (5.8 %)
Van13 (42 ms)	Dro22	LinInt	29.4±17.2	−27.1±20.6	24.6±23.2	379 (5.7 %)
For18	Ste85	None	30.0±31.7	27.1±34.2	22.1±23.6	641 (9.7 %)
Van13 (32 ms)	Arb17TD	For18	30.0±28.9	−7.0±41.1	24.4±27.0	817 (12.4 %)
		LinInt	30.3±28.6	−8.9±40.7	24.7±27.0	792 (12.0 %)
Mar04	Arb17TD	For18	30.8±29.1	−9.0±41.4	24.7±26.8	891 (13.5 %)
Van13 (32 ms)	She90	For18	30.9±21.7	0.0±37.8	24.3±20.9	380 (5.7 %)
	Arb17IC	LinInt	31.0±21.9	−0.2±38.0	24.3±20.9	380 (5.7 %)
	She90	LinInt	31.0±21.7	−1.1±37.8	24.4±21.0	380 (5.7 %)
For18	Arb17IC	None	31.0±27.8	13.9±39.2	23.4±21.4	392 (5.9 %)
Van13 (32 ms)	Arb17IC	For18	31.2±21.9	0.7±38.1	24.4±20.6	380 (5.7 %)
Mar04	Arb17TD	LinInt	31.2±28.7	−10.9±41.0	25.1±26.8	862 (13.0 %)
	She90	For18	31.2±21.9	−2.1±38.1	24.5±20.9	484 (7.3 %)
	Arb17IC	LinInt	31.3±22.1	−2.3±38.3	24.5±20.9	483 (7.3 %)
Van13 (34 ms)	Arb17TD	For18	31.3±28.5	−8.7±41.5	25.4±27.0	804 (12.2 %)
Mar04	She90	LinInt	31.3±21.8	−3.2±38.1	24.6±21.0	484 (7.3 %)
	Arb17IC	For18	31.4±22.1	−1.3±38.3	24.5±20.7	483 (7.3 %)
Van13 (34 ms)	She90	For18	31.5±21.0	−2.0±37.8	24.8±21.0	380 (5.7 %)
	Arb17IC	LinInt	31.6±21.2	−2.2±38.0	24.9±20.9	380 (5.7 %)
For18	She90	None	31.6±28.0	14.3±39.7	23.8±21.6	424 (6.4 %)
Van13 (34 ms)	She90	LinInt	31.6±21.0	−3.1±37.8	24.9±21.1	380 (5.7 %)
	Arb17IC	For18	31.7±21.1	−1.3±38.1	24.9±20.7	380 (5.7 %)
	Arb17TD	LinInt	31.7±28.3	−10.7±41.1	25.8±27.1	780 (11.8 %)
Van13 (36 ms)	She90	For18	32.1±20.4	−4.0±37.8	25.3±21.1	380 (5.7 %)
	Arb17IC	LinInt	32.2±20.6	−4.2±38.0	25.4±21.1	380 (5.7 %)
	She90	LinInt	32.2±20.4	−5.1±37.8	25.5±21.3	380 (5.7 %)
	Arb17IC	For18	32.3±20.5	−3.3±38.1	25.4±20.8	380 (5.7 %)
Van13 (32 ms)	Arb17IC	None	32.6±22.7	3.8±39.5	25.3±20.5	387 (5.9 %)
Mar04	Arb17IC	None	32.6±22.6	1.8±39.6	25.3±20.4	491 (7.4 %)
Van13 (38 ms)	She90	For18	32.7±19.9	−6.0±37.8	25.9±21.3	380 (5.7 %)
Van13 (36 ms)	Arb17TD	For18	32.8±28.2	−10.4±42.0	26.5±27.1	791 (12.0 %)
For18	Loz07QR	LinInt	32.8±20.9	28.5±26.6	22.9±14.4	382 (5.8 %)
Van13 (38 ms)	Arb17IC	LinInt	32.9±20.1	−6.2±38.0	26.0±21.3	379 (5.7 %)
		For18	32.9±20.0	−5.3±38.1	26.0±21.1	379 (5.7 %)
	She90	LinInt	32.9±19.9	−7.1±37.8	26.1±21.5	380 (5.7 %)
For18	Loz07QR	For18	33.0±20.9	28.7±26.5	23.0±14.3	382 (5.8 %)
Van13 (32 ms)	She90	None	33.0±22.7	4.0±39.8	25.6±20.6	424 (6.4 %)
Van13 (34 ms)	Arb17IC	None	33.1±21.8	1.8±39.6	25.7±20.4	386 (5.8 %)
Mar04	She90	None	33.2±22.6	2.1±40.1	25.7±20.6	525 (7.9 %)
Van13 (36 ms)	Arb17TD	LinInt	33.2±27.9	−12.4±41.6	27.0±27.2	768 (11.6 %)
Van13 (40 ms)	She90	For18	33.3±19.6	−8.0±37.8	26.5±21.6	380 (5.7 %)
Van13 (34 ms)	She90	None	33.4±21.8	2.0±39.8	26.0±20.6	424 (6.4 %)
Van13 (40 ms)	Arb17IC	For18	33.5±19.6	−7.3±38.1	26.6±21.3	379 (5.7 %)
Van13 (36 ms)	Arb17IC	None	33.5±21.0	−0.2±39.6	26.2±20.5	386 (5.8 %)
Van13 (40 ms)	Arb17IC	LinInt	33.5±19.7	−8.2±38.0	26.7±21.6	379 (5.7 %)
	She90	LinInt	33.6±19.6	−9.1±37.8	26.8±21.8	380 (5.7 %)
For18	Loz07QR	None	33.7±21.3	30.0±26.2	23.4±13.9	382 (5.8 %)

Continued on next page

Table S23: Results of combined PEP extraction pipelines on the *Guardian Dataset*. The pipelines are sorted by the MAE in ascending order.

Q-Peak Detection	B-Point Detection	Outlier Correction	**MAE [ms]**	**ME [ms]**	**MARE [%]**	**Invalid PEPs**
Van13 (36 ms)	She90	None	33.9±21.0	0.0±39.8	26.4±20.6	424 (6.4 %)
Van13 (42 ms)	She90	For18	34.0±19.3	−10.0±37.8	27.1±22.0	380 (5.7 %)
Van13 (38 ms)	Arb17IC	None	34.0±20.4	−2.2±39.6	26.7±20.6	385 (5.8 %)
Van13 (42 ms)	Arb17IC	For18	34.1±19.3	−9.3±38.1	27.2±21.7	379 (5.7 %)
Van13 (38 ms)	Arb17TD	For18	34.2±27.8	−12.1±42.4	27.7±27.2	777 (11.8 %)
Van13 (42 ms)	Arb17IC	LinInt	34.2±19.4	−10.2±38.0	27.3±22.0	379 (5.7 %)
Van13 (38 ms)	She90	None	34.3±20.3	−2.0±39.8	26.9±20.7	424 (6.4 %)
Van13 (42 ms)	She90	LinInt	34.4±19.3	−11.1±37.8	27.4±22.2	380 (5.7 %)
Van13 (40 ms)	Arb17IC	None	34.5±19.8	−4.2±39.6	27.2±20.8	385 (5.8 %)
Van13 (42 ms)	Arb17SD	LinInt	34.5±19.8	25.7±30.5	25.6±16.2	390 (5.9 %)
Van13 (32 ms)	Arb17TD	None	34.7±35.1	1.2±49.3	27.5±29.7	1195 (18.1 %)
Van13 (38 ms)	Arb17TD	LinInt	34.7±27.6	−14.0±42.0	28.2±27.3	754 (11.4 %)
Van13 (40 ms)	She90	None	34.8±19.8	−4.0±39.8	27.4±20.9	424 (6.4 %)
Van13 (42 ms)	Arb17SD	For18	34.8±19.7	26.1±30.3	25.8±16.1	390 (5.9 %)
	Arb17IC	None	35.0±19.4	−6.2±39.6	27.7±21.1	385 (5.8 %)
	She90	None	35.3±19.4	−6.0±39.8	27.9±21.2	424 (6.4 %)
Van13 (40 ms)	Arb17TD	For18	35.7±27.5	−13.7±42.9	28.8±27.3	762 (11.5 %)
	Arb17SD	LinInt	35.8±20.2	27.6±30.4	26.4±16.0	391 (5.9 %)
Mar04	Arb17TD	None	35.8±35.2	0.0±50.2	28.0±29.4	1242 (18.8 %)
Van13 (42 ms)	Arb17SD	None	36.0±19.7	27.6±30.4	26.7±16.0	391 (5.9 %)
Van13 (34 ms)	Arb17TD	None	36.1±34.5	−0.3±49.9	28.6±29.5	1172 (17.7 %)
Van13 (40 ms)	Arb17SD	For18	36.1±20.1	28.1±30.3	26.7±15.9	391 (5.9 %)
	Arb17TD	LinInt	36.3±27.3	−15.7±42.6	29.4±27.4	739 (11.2 %)
Van13 (38 ms)	Arb17SD	LinInt	37.1±20.6	29.6±30.4	27.3±15.9	391 (5.9 %)
Van13 (42 ms)	Arb17TD	For18	37.3±27.2	−15.3±43.5	30.0±27.4	746 (11.3 %)
Van13 (40 ms)	Arb17SD	None	37.4±20.0	29.6±30.4	27.6±15.7	392 (5.9 %)
Van13 (38 ms)	Arb17SD	For18	37.5±20.5	30.1±30.3	27.5±15.8	391 (5.9 %)
Van13 (36 ms)	Arb17TD	None	37.6±34.1	−1.6±50.7	29.7±29.4	1145 (17.3 %)
Van13 (42 ms)	Arb17TD	LinInt	37.9±27.0	−17.3±43.2	30.6±27.5	724 (11.0 %)
Van13 (36 ms)	Arb17SD	LinInt	38.5±21.2	31.6±30.4	28.2±15.9	391 (5.9 %)
Van13 (38 ms)	Arb17SD	None	38.7±20.5	31.6±30.4	28.5±15.6	392 (5.9 %)
Van13 (36 ms)	Arb17SD	For18	38.8±21.0	32.1±30.3	28.4±15.8	391 (5.9 %)
Van13 (38 ms)	Arb17TD	None	39.1±33.5	−3.0±51.4	30.8±29.3	1120 (16.9 %)
Van13 (34 ms)	Arb17SD	LinInt	39.8±21.7	33.6±30.4	29.0±15.9	391 (5.9 %)
Van13 (36 ms)	Arb17SD	None	40.1±21.0	33.6±30.4	29.4±15.6	392 (5.9 %)
Mar04	Arb17SD	LinInt	40.2±22.5	33.7±31.4	29.4±16.6	494 (7.5 %)
Van13 (34 ms)	Arb17SD	For18	40.2±21.6	34.1±30.3	29.3±15.8	391 (5.9 %)
Mar04	Arb17SD	For18	40.5±22.3	34.1±31.3	29.7±16.5	494 (7.5 %)
Van13 (40 ms)	Arb17TD	None	40.6±32.9	−4.4±52.1	32.0±29.2	1093 (16.5 %)
Van13 (32 ms)	Arb17SD	LinInt	41.2±22.3	35.6±30.4	29.9±16.0	391 (5.9 %)
Van13 (34 ms)	Arb17SD	None	41.5±21.5	35.6±30.4	30.3±15.6	392 (5.9 %)
Van13 (32 ms)	Arb17SD	For18	41.6±22.2	36.1±30.3	30.2±15.9	391 (5.9 %)
Mar04	Arb17SD	None	41.9±22.3	35.6±31.4	30.7±16.4	495 (7.5 %)
Van13 (42 ms)	Arb17TD	None	42.1±32.4	−5.7±52.9	33.2±29.1	1064 (16.1 %)
For18	Pal21	LinInt	42.9±35.6	2.4±55.7	33.8±30.6	2261 (34.2 %)
Van13 (32 ms)	Arb17SD	None	42.9±22.1	37.6±30.4	31.2±15.7	392 (5.9 %)
For18	Pal21	For18	44.1±37.0	4.3±57.4	34.4±30.8	2309 (34.9 %)
		None	45.6±39.0	7.3±59.6	35.3±31.5	2701 (40.9 %)
	Arb17SD	LinInt	48.7±25.5	45.9±30.3	35.0±17.3	392 (5.9 %)
		For18	49.1±25.3	46.3±30.2	35.3±17.2	392 (5.9 %)
Van13 (32 ms)	Pal21	LinInt	49.6±37.5	−1.3±62.1	39.5±33.5	2021 (30.6 %)
Mar04	Pal21	LinInt	50.2±37.4	−3.0±62.5	39.7±33.2	2061 (31.2 %)
Van13 (32 ms)	Pal21	For18	50.5±38.5	0.6±63.5	40.0±33.6	2069 (31.3 %)
For18	Arb17SD	None	50.6±25.4	47.8±30.3	36.4±17.2	392 (5.9 %)
Van13 (34 ms)	Pal21	LinInt	50.9±37.5	−2.0±63.2	40.6±33.7	1974 (29.9 %)

Continued on next page

Table S23: Results of combined PEP extraction pipelines on the *Guardian Dataset*. The pipelines are sorted by the MAE in ascending order.

Q-Peak Detection	B-Point Detection	Outlier Correction	**MAE [ms]**	**ME [ms]**	**MARE [%]**	**Invalid PEPs**
Mar04	Pal21	For18	51.1±38.2	−1.0±63.8	40.2±33.2	2105 (31.8 %)
Van13 (34 ms)	Pal21	For18	51.8±38.4	−0.1±64.5	41.1±33.8	2021 (30.6 %)
Van13 (36 ms)	Pal21	LinInt	52.3±37.4	−2.8±64.2	41.7±33.8	1932 (29.2 %)
Van13 (32 ms)	Pal21	None	52.3±40.3	4.9±65.9	41.2±34.1	2427 (36.7 %)
Mar04	Pal21	None	53.0±40.0	3.8±66.3	41.3±33.6	2457 (37.2 %)
Van13 (36 ms)	Pal21	For18	53.1±38.2	−0.9±65.4	42.1±33.8	1978 (29.9 %)
Van13 (34 ms)	Pal21	None	53.8±40.3	4.6±67.0	42.4±34.2	2369 (35.8 %)
Van13 (38 ms)	Pal21	LinInt	53.8±37.2	−3.4±65.4	42.9±33.8	1881 (28.5 %)
		For18	54.6±38.0	−1.5±66.5	43.3±33.9	1926 (29.1 %)
Van13 (36 ms)	Pal21	None	55.2±39.9	4.0±68.0	43.5±34.1	2320 (35.1 %)
Van13 (40 ms)	Pal21	LinInt	55.2±36.8	−4.3±66.2	44.0±33.8	1840 (27.8 %)
		For18	56.0±37.4	−2.4±67.3	44.4±33.8	1886 (28.5 %)
Van13 (42 ms)	Pal21	LinInt	56.6±36.4	−5.1±67.1	45.0±33.8	1798 (27.2 %)
Van13 (38 ms)	Pal21	None	56.7±39.5	3.6±69.0	44.7±34.0	2264 (34.2 %)
Van13 (42 ms)	Pal21	For18	57.4±37.0	−3.2±68.2	45.4±33.7	1842 (27.9 %)
Van13 (40 ms)	Pal21	None	58.1±38.9	2.9±69.9	45.8±33.9	2215 (33.5 %)
Van13 (42 ms)	Pal21	None	59.5±38.3	2.2±70.7	46.9±33.6	2170 (32.8 %)

4.4.2 Absolute Error

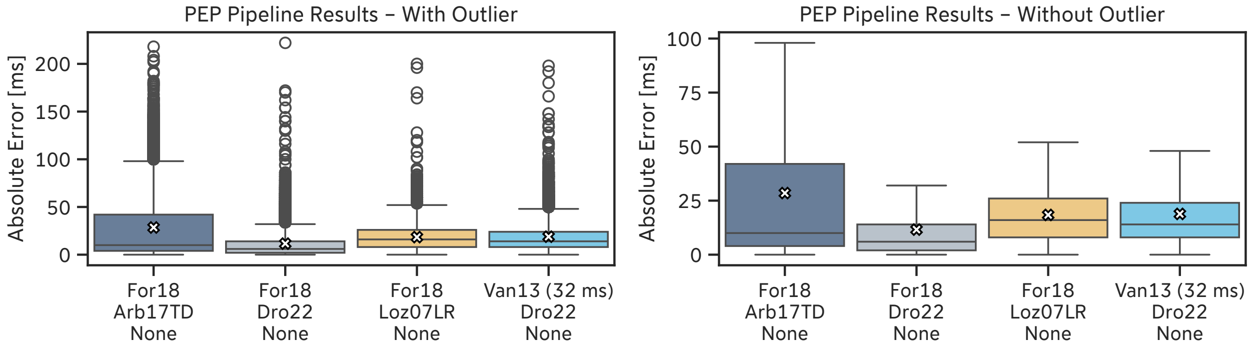

Figure S59: Absolute error of the selected PEP pipelines on the *Guardian Dataset* with outliers (left) and without outliers (right). Mean values are denoted by the white cross.

Table S24: Mean Abolute Error of selected PEP extraction pipelines on the *Guardian Dataset* per participant. The values with the highest errors are highlighted in red.

								Absolute Error [ms]
**Q-peak Algorithm**						**For18**		**Van13 (32 ms)**
**B-point Algorithm**		**Arb17TD**		**Dro22**		**Loz07LR**		**Dro22**
**Outlier Correction Algorithm**		**None**		**None**		**None**		**None**
	**Mean**	**SD**	**Mean**	**SD**	**Mean**	**SD**	**Mean**	**SD**
Participant								
GDN0005	20.63	29.23	9.90	12.39	19.72	13.41	11.70	13.91
GDN0006	13.06	24.30	6.08	6.33	25.83	11.15	7.65	8.02
GDN0007	31.90	40.09	8.93	10.25	16.24	11.11	17.20	11.37
GDN0008	27.44	43.24	7.08	9.01	19.56	12.76	9.41	9.57
GDN0009	42.87	41.07	16.88	11.74	15.52	11.19	21.86	10.47
GDN0010	35.96	37.77	14.32	18.40	17.41	14.40	24.58	18.03
GDN0011	27.38	36.71	9.51	11.36	12.67	9.27	16.43	11.61
GDN0012	26.32	32.01	11.76	15.35	15.27	14.61	22.03	15.37
GDN0013	34.91	44.63	7.18	10.45	17.87	11.07	13.22	11.24
GDN0014	30.24	37.83	11.67	14.28	20.53	12.37	20.36	13.30
GDN0016	30.71	35.18	12.39	15.23	19.38	12.67	17.91	14.34
GDN0017	37.12	49.86	6.54	11.94	26.70	11.73	14.89	11.00
GDN0018	26.19	26.88	16.12	17.32	22.66	15.55	35.46	17.34
GDN0019	18.10	14.69	10.41	10.86	13.35	10.13	23.03	10.78
GDN0020	27.08	23.89	16.07	15.26	16.53	11.63	28.47	17.21
GDN0021	27.01	33.82	9.96	10.60	10.53	8.98	27.08	10.32
GDN0022	36.49	43.62	15.66	23.05	26.13	21.86	15.64	18.67
GDN0023	18.49	22.73	9.23	9.26	15.30	9.50	13.33	10.03
GDN0024	16.85	23.51	8.63	15.57	16.77	13.28	16.90	15.59
GDN0025	39.97	38.67	19.59	22.42	21.73	20.03	24.76	23.96
GDN0027	41.75	46.39	14.67	21.44	20.01	20.75	20.80	23.30
GDN0028	37.91	41.15	21.91	25.57	23.96	15.84	23.33	23.70
GDN0029	35.66	45.35	11.36	22.15	16.61	17.07	12.84	20.80
GDN0030	29.42	32.13	15.74	22.84	18.79	11.87	17.42	22.30

4.4.3 Residual Plots – Overall and Detailed

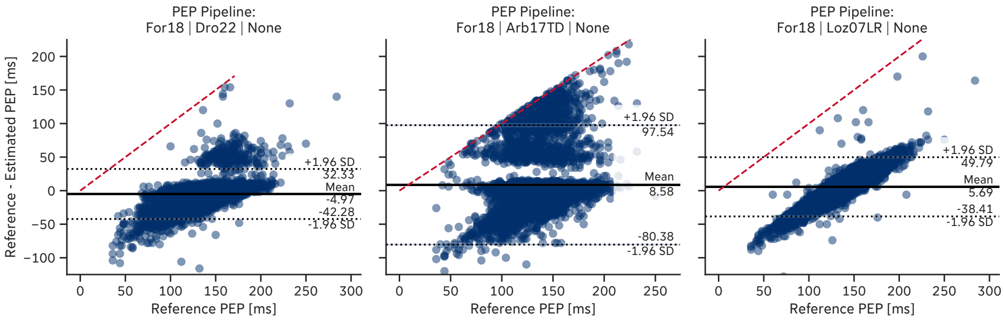

Figure S60: Residual plots of the selected PEP pipelines on the *Guardian Dataset*.

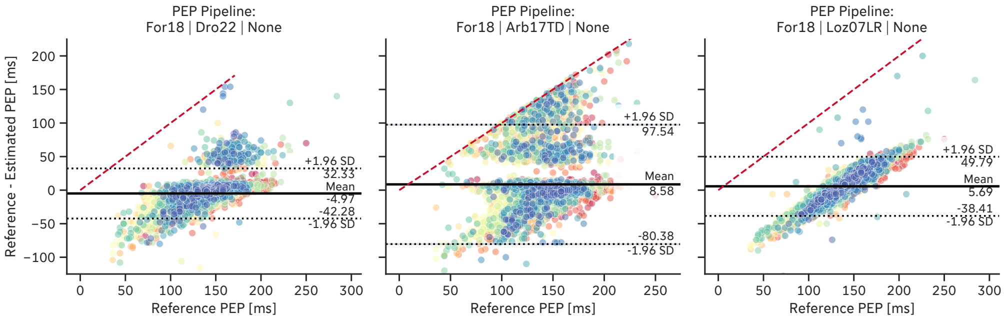

Figure S61: Residual plots of the selected PEP pipelines on the *Guardian Dataset*. Each participant is represented by a different color.

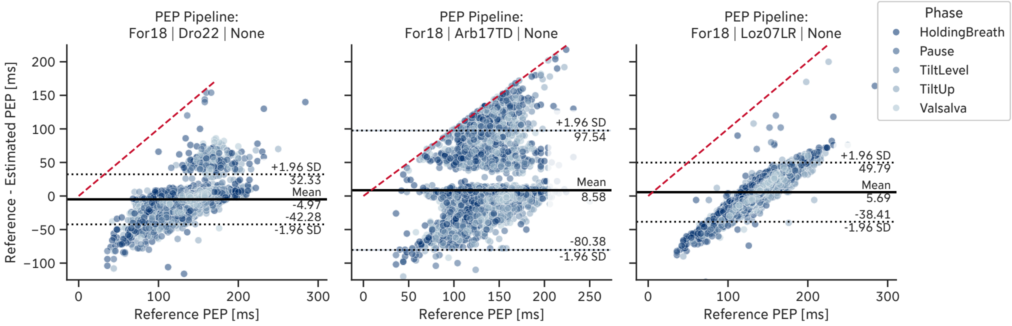

Figure S62: Residual plots of the selected PEP pipelines on the *Guardian Dataset*. Each phase is represented by a different color.

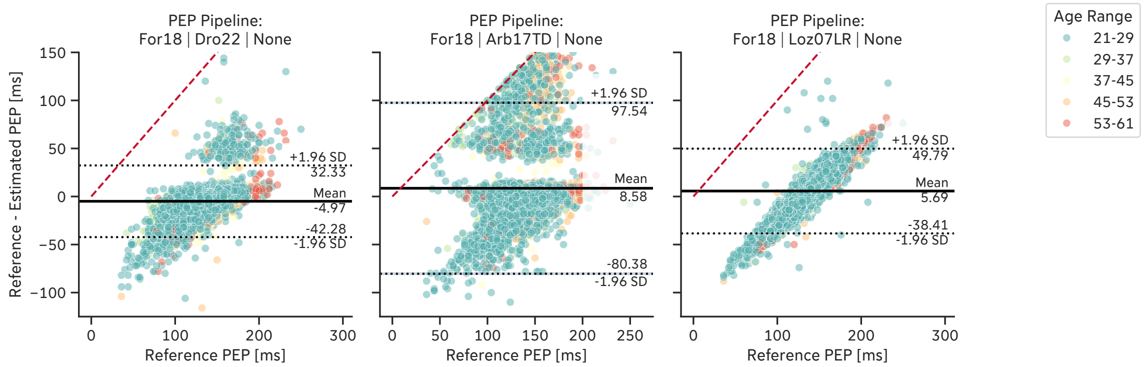

Figure S63: Residual plots of selected PEP pipelines algorithms on the *Guardian Dataset*. Each age range is represented by a different color. The red dashed line indicates the upper estimation error limit given by the location of the reference Q-peaks.

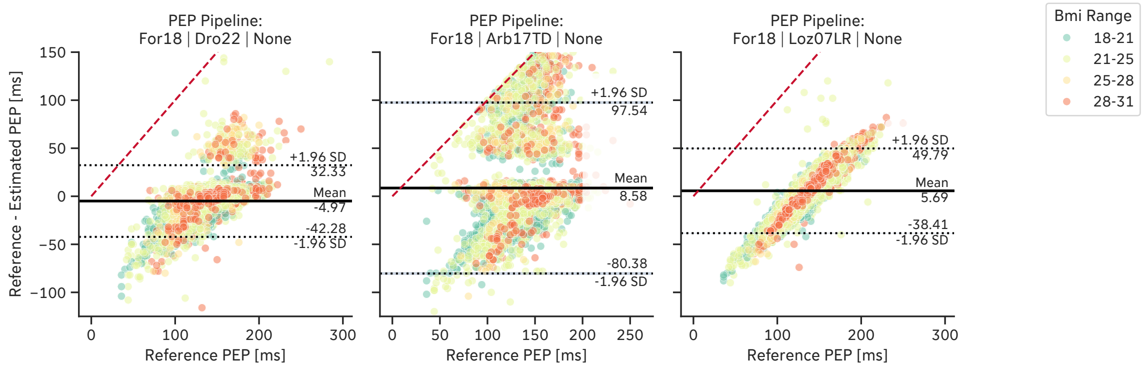

Figure S64: Residual plots of selected PEP pipelines on the *Guardian Dataset*. Each BMI range is represented by a different color. The red dashed line indicates the upper estimation error limit given by the location of the reference Q-peaks.

4.4.4 Effect of Heart Rate on PEP Pipeline Extraction Error

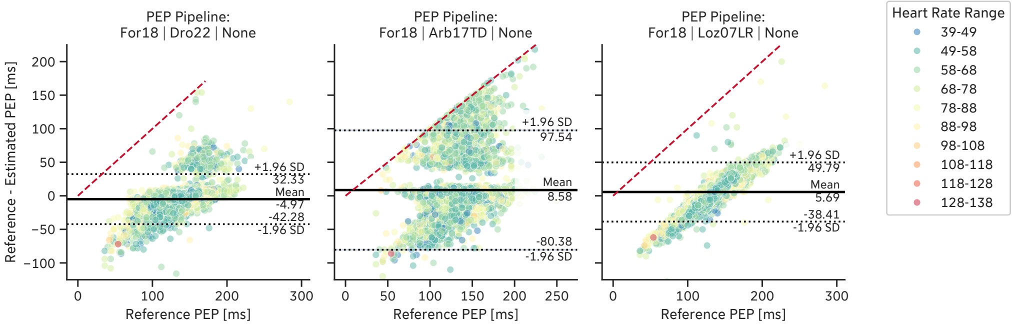

Figure S65: Residual plots of the selected PEP pipelines on the *Guardian Dataset*. Each heart rate bin is represented by a different color.

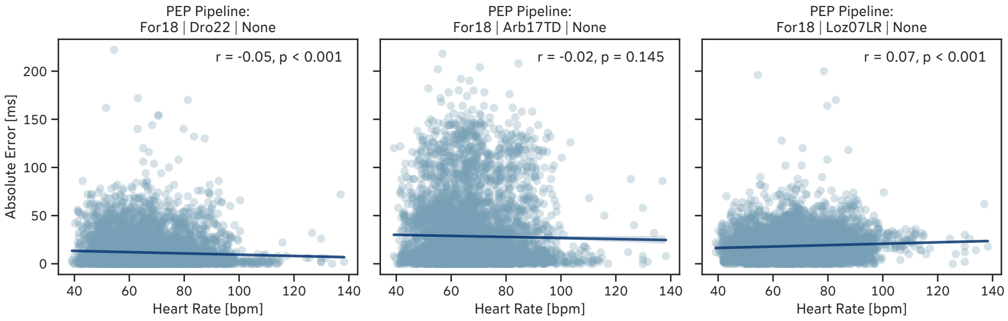

Figure S66: Regression plot between heart rate and absolute error of the selected PEP pipelines on the *Guardian Dataset*.

4.4.5 Effect of Outlier Correction on PEP Pipeline Performance

Table S25: Mean Absolute Error (MAE) of the outlier correction algorithms for the different B-point extraction algorithms on the *Guardian Dataset*. MAE values are provided in milliseconds as ($M \pm SD$).

**Q-peak Algorithm**	**B-point Algorithm**	**Outlier Correction Algorithm** For18	LinInt	None
For18	Arb17IC	29.2±26.4	**28.9±26.3**	31.0±27.8
	Arb17SD	49.1±25.3	**48.7±25.5**	50.6±25.4
	Arb17TD	24.1±29.6	**24.0±29.1**	28.5±36.4
	Deb93SD	**26.5±18.4**	26.9±18.7	26.9±18.1
	Dro22	12.3±15.9	12.5±16.2	**11.6±15.9**
	For18	21.5±27.3	**21.2±27.0**	23.6±30.4
	Loz07LR	18.6±15.0	18.6±15.0	**18.4±14.2**
	Loz07QR	33.0±20.9	**32.8±20.9**	33.7±21.3
	Mil22	23.5±29.5	**23.0±29.1**	25.9±32.5
	Pal21	44.1±37.0	**42.9±35.6**	45.6±39.0
	She90	29.0±26.1	**28.8±26.0**	31.6±28.0
	Ste85	26.3±27.5	**25.6±26.9**	30.0±31.7
Mar04	Arb17IC	31.4±22.1	**31.3±22.1**	32.6±22.6
	Arb17SD	40.5±22.3	**40.2±22.5**	41.9±22.3
	Arb17TD	**30.8±29.1**	31.2±28.7	35.8±35.2
	Deb93SD	25.6±26.1	26.4±26.5	**24.9±25.8**
	Dro22	21.4±18.9	21.7±19.2	**20.4±18.3**
	For18	20.4±26.9	**20.3±26.8**	21.9±29.7
	Loz07LR	18.8±17.8	18.8±17.9	**18.3±16.6**
	Loz07QR	**26.2±18.2**	26.2±18.2	26.7±18.4
	Mil22	24.1±27.9	**23.9±27.7**	25.6±30.0
	Pal21	51.1±38.2	**50.2±37.4**	53.0±40.0
	She90	**31.2±21.9**	31.3±21.8	33.2±22.6
	Ste85	20.5±29.6	**20.0±28.8**	23.6±33.5
Van13 (32 ms)	Arb17IC	31.2±21.9	**31.0±21.9**	32.6±22.7
	Arb17SD	41.6±22.2	**41.2±22.3**	42.9±22.1
	Arb17TD	**30.0±28.9**	30.3±28.6	34.7±35.1
	Deb93SD	24.4±24.4	25.2±24.8	**23.9±24.2**
	Dro22	19.8±17.1	20.1±17.4	**18.9±16.6**
	For18	20.4±26.4	**20.3±26.2**	22.3±29.4
	Loz07LR	18.3±16.5	18.4±16.6	**17.9±15.4**
	Loz07QR	**26.9±19.1**	26.9±19.1	27.5±19.3
	Mil22	23.7±27.0	**23.5±26.8**	25.6±29.5
	Pal21	50.5±38.5	**49.6±37.5**	52.3±40.3
	She90	**30.9±21.7**	31.0±21.7	33.0±22.7
	Ste85	21.2±28.9	**20.6±27.9**	24.7±33.2
Van13 (34 ms)	Arb17IC	31.7±21.1	**31.6±21.2**	33.1±21.8
	Arb17SD	40.2±21.6	**39.8±21.7**	41.5±21.5
	Arb17TD	**31.3±28.5**	31.7±28.3	36.1±34.5
	Deb93SD	24.4±25.6	25.2±25.9	**23.7±25.3**
	Dro22	21.6±17.2	21.9±17.3	**20.6±16.4**
	For18	**20.5±25.9**	20.5±25.7	22.4±28.7
	Loz07LR	18.6±17.0	18.6±17.1	**18.1±15.9**
	Loz07QR	26.0±18.8	**25.9±18.8**	26.5±19.0
	Mil22	24.2±26.3	**24.1±26.2**	26.0±28.7
	Pal21	51.8±38.4	**50.9±37.5**	53.8±40.3
	She90	**31.5±21.0**	31.6±21.0	33.4±21.8
	Ste85	20.7±28.6	**20.2±27.7**	24.1±32.9
	Arb17IC	32.3±20.5	**32.2±20.6**	33.5±21.0

Continued on next page

Van13 (36 ms)

Table S25: Mean Absolute Error (MAE) of the outlier correction algorithms for the different B-point extraction algorithms on the *Guardian Dataset*. MAE values are provided in milliseconds as (M ± SD).

**Q-peak Algorithm**	**B-point Algorithm**	**Outlier Correction Algorithm** For18	**LinInt**	**None**
	Arb17SD	38.8±21.0	**38.5**±**21.2**	40.1±21.0
	Arb17TD	**32.8**±**28.2**	33.2±27.9	37.6±34.1
	Deb93SD	24.5±26.6	25.4±27.0	**23.6**±**26.4**
	Dro22	23.5±17.1	23.7±17.4	**22.5**±**16.2**
	For18	**21.1**±**25.4**	21.1±25.3	22.8±28.1
	Loz07LR	18.9±17.5	19.0±17.6	**18.4**±**16.4**
	Loz07QR	**25.0**±**18.5**	25.0±18.5	25.5±18.6
	Mil22	**24.9**±**25.5**	24.9±25.5	26.6±27.7
	Pal21	53.1±38.2	**52.3**±**37.4**	55.2±39.9
	She90	**32.1**±**20.4**	32.2±20.4	33.9±21.0
	Ste85	20.6±28.3	**20.1**±**27.4**	24.0±32.4
Van13 (38 ms)	Arb17IC	**32.9**±**20.0**	32.9±20.1	34.0±20.4
	Arb17SD	37.5±20.5	**37.1**±**20.6**	38.7±20.5
	Arb17TD	**34.2**±**27.8**	34.7±27.6	39.1±33.5
	Deb93SD	24.8±27.6	25.8±28.1	**23.7**±**27.4**
	Dro22	25.3±17.0	25.6±17.3	**24.3**±**16.1**
	For18	**21.7**±**24.8**	21.8±24.7	23.4±27.2
	Loz07LR	19.5±18.2	19.6±18.2	**19.0**±**17.0**
	Loz07QR	**24.2**±**18.1**	24.2±18.1	24.6±18.2
	Mil22	**25.8**±**24.6**	25.8±24.6	27.4±26.7
	Pal21	54.6±38.0	**53.8**±**37.2**	56.7±39.5
	She90	**32.7**±**19.9**	32.9±19.9	34.3±20.3
	Ste85	20.9±27.7	**20.5**±**26.9**	24.2±31.8
Van13 (40 ms)	Arb17IC	**33.5**±**19.6**	33.5±19.7	34.5±19.8
	Arb17SD	36.1±20.1	**35.8**±**20.2**	37.4±20.0
	Arb17TD	**35.7**±**27.5**	36.3±27.3	40.6±32.9
	Deb93SD	25.3±28.4	26.4±29.0	**24.0**±**28.3**
	Dro22	27.2±16.9	27.5±17.2	**26.2**±**16.0**
	For18	**22.6**±**24.2**	22.7±24.1	24.3±26.5
	Loz07LR	20.2±18.7	20.3±18.8	**19.6**±**17.6**
	Loz07QR	**23.4**±**17.8**	23.4±17.8	23.8±17.8
	Mil22	**26.8**±**23.8**	26.8±23.8	28.2±25.6
	Pal21	56.0±37.4	**55.2**±**36.8**	58.1±38.9
	She90	**33.3**±**19.6**	33.6±19.6	34.8±19.8
	Ste85	21.5±27.1	**21.2**±**26.4**	24.9±31.2
Van13 (42 ms)	Arb17IC	**34.1**±**19.3**	34.2±19.4	35.0±19.4
	Arb17SD	34.8±19.7	**34.5**±**19.8**	36.0±19.7
	Arb17TD	**37.3**±**27.2**	37.9±27.0	42.1±32.4
	Deb93SD	26.2±29.3	27.3±29.7	**24.6**±**29.1**
	Dro22	29.1±16.9	29.4±17.2	**28.0**±**15.9**
	For18	**23.6**±**23.5**	23.7±23.5	25.2±25.5
	Loz07LR	21.1±19.3	21.2±19.3	**20.5**±**18.1**
	Loz07QR	**22.8**±**17.6**	22.8±17.6	23.1±17.5
	Mil22	**27.7**±**23.0**	27.9±23.0	29.1±24.6
	Pal21	57.4±37.0	**56.6**±**36.4**	59.5±38.3
	She90	**34.0**±**19.3**	34.4±19.3	35.3±19.4
	Ste85	22.3±26.2	**22.0**±**25.5**	25.6±30.1

4.5 Individually Lowest vs. Overall Lowest PEP Pipelines

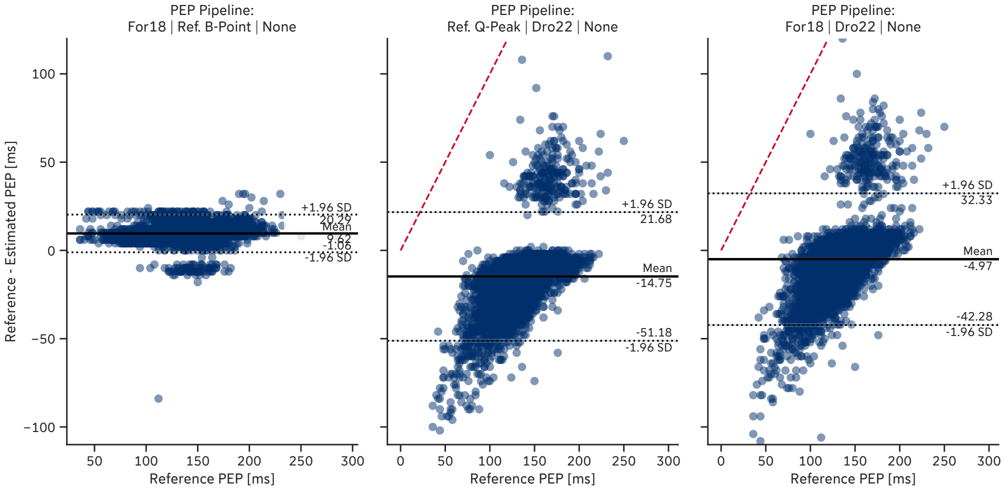

Figure S67: Residual plot of the single PEP pipeline extraction steps of the *individually lowest* PEP pipeline (i.e., the PEP pipeline consisting of the algorithms with the individually lowest MAE) on the *Guardian Dataset*.

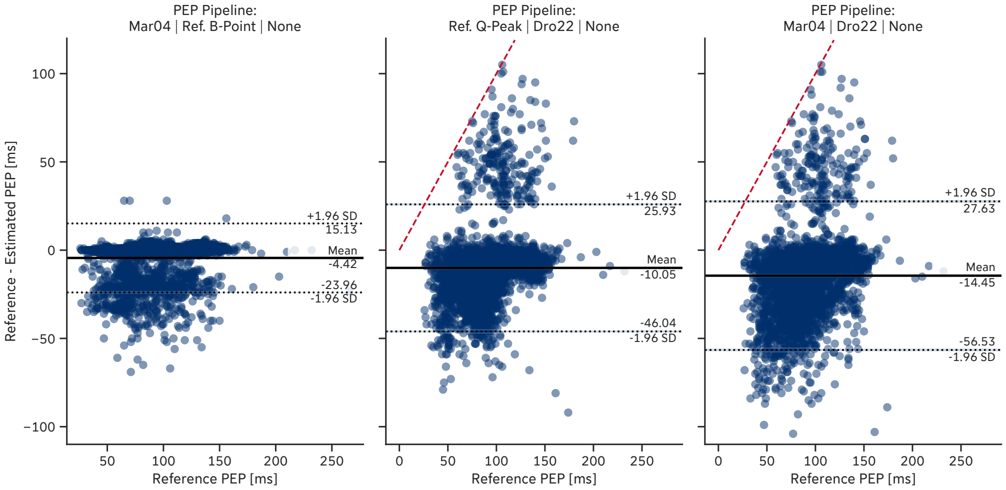

Figure S68: Residual plot of the single PEP pipeline extraction steps of the *overall lowest* PEP pipeline (i.e., the PEP pipeline with the overall lowest MAE) on the *Guardian Dataset*.

4.5.1 Effect of Annotations on B-Point Detection Error

Table S26: Error metrics of PEP extraction pipelines on the *Guardian Dataset* for different annotators and MAE difference between both annotators. MAE = Mean Absolute Error, ME = Mean Error.

			Annotator 1				Annotator 2				Ann. Diff.	
			MAE [ms]		ME [ms]		MAE [ms]		ME [ms]		MAE [ms]	
Q-peak Algorithm	B-point Algorithm	Outlier Correction Algorithm	Mean	SD	Mean	SD	Mean	SD	Mean	SD	Mean	SD
For18	Dro22	None	11.61	15.88	−4.97	19.03	13.47	19.23	−9.45	21.49	1.86	3.35
		For18	12.30	15.92	−6.62	18.99	14.53	19.45	−11.15	21.57	2.24	3.53
		LinInt	12.51	16.15	−7.02	19.18	14.85	19.73	−11.56	21.82	2.34	3.58
Van13 (32 ms)	Loz07LR	None	17.88	15.43	−4.51	23.19	19.98	18.23	−9.01	25.50	2.10	2.80
Van13 (34 ms)	Loz07LR	None	18.08	15.91	−6.51	23.19	20.40	18.85	−11.01	25.50	2.32	2.94
Mar04	Loz07LR	None	18.28	16.64	−6.53	23.84	20.93	19.79	−10.99	26.63	2.64	3.16
Van13 (32 ms)	Loz07LR	For18	18.32	16.51	−5.29	24.10	20.46	19.24	−9.78	26.33	2.14	2.73
		LinInt	18.38	16.57	−5.40	24.15	20.53	19.31	−9.89	26.39	2.15	2.74
For18	Loz07LR	None	18.39	14.16	5.69	22.50	19.37	15.94	1.20	25.06	0.98	1.78
Van13 (36 ms)	Loz07LR	None	18.44	16.43	−8.51	23.19	20.96	19.50	−13.01	25.50	2.52	3.07
Van13 (34 ms)	Loz07LR	For18	18.56	17.01	−7.29	24.10	20.92	19.86	−11.78	26.33	2.36	2.85
For18	Loz07LR	For18	18.62	14.98	4.89	23.40	19.66	16.79	0.40	25.85	1.04	1.81
Van13 (34 ms)	Loz07LR	LinInt	18.62	17.06	−7.40	24.15	20.99	19.93	−11.89	26.39	2.37	2.87
For18	Loz07LR	LinInt	18.65	14.99	4.78	23.45	19.70	16.83	0.29	25.91	1.05	1.84
Mar04	Loz07LR	For18	18.77	17.81	−7.32	24.82	21.45	20.86	−11.79	27.50	2.67	3.06
		LinInt	18.83	17.85	−7.43	24.87	21.52	20.92	−11.90	27.56	2.69	3.07
Van13 (32 ms)	Dro22	None	18.86	16.59	−15.14	20.04	21.41	20.51	−19.61	22.24	2.55	3.92
Van13 (36 ms)	Loz07LR	For18	18.95	17.54	−9.29	24.10	21.51	20.50	−13.78	26.33	2.56	2.96
Van13 (38 ms)	Loz07LR	None	18.95	17.00	−10.51	23.19	21.68	20.14	−15.01	25.50	2.72	3.15
Van13 (36 ms)	Loz07LR	LinInt	19.02	17.59	−9.40	24.15	21.60	20.57	−13.89	26.39	2.57	2.97
Van13 (38 ms)	Loz07LR	For18	19.52	18.18	−11.26	24.18	22.29	21.19	−15.76	26.41	2.77	3.01
		LinInt	19.60	18.24	−11.37	24.23	22.38	21.25	−15.87	26.47	2.78	3.02
Van13 (40 ms)	Loz07LR	None	19.64	17.56	−12.51	23.19	22.55	20.76	−17.01	25.50	2.91	3.20
Van13 (32 ms)	Dro22	For18	19.80	17.08	−16.77	20.06	22.70	20.94	−21.27	22.39	2.90	3.86
Mar04	Ste85	LinInt	20.00	28.77	9.59	33.70	18.71	28.13	5.72	33.30	−1.29	−0.63
Van13 (32 ms)	Dro22	LinInt	20.06	17.39	−17.16	20.25	23.07	21.22	−21.68	22.64	3.01	3.83
Van13 (36 ms)	Ste85	LinInt	20.14	27.39	8.22	32.99	18.12	27.11	4.41	32.31	−2.02	−0.29
Van13 (34 ms)	Ste85	LinInt	20.19	27.72	10.10	32.77	18.03	27.22	6.28	32.04	−2.16	−0.50
Van13 (40 ms)	Loz07LR	For18	20.24	18.73	−13.26	24.18	23.19	21.79	−17.76	26.41	2.96	3.06
		LinInt	20.32	18.79	−13.37	24.23	23.29	21.85	−17.87	26.47	2.97	3.06
Van13 (32 ms)	For18	LinInt	20.32	26.24	5.67	32.70	19.56	25.85	1.84	32.36	−0.77	−0.39
Mar04	For18	LinInt	20.32	26.80	3.28	33.48	20.42	26.57	−0.56	33.51	0.10	−0.23
Van13 (32 ms)	For18	For18	20.39	26.40	6.72	32.68	19.39	25.82	2.94	32.16	−1.00	−0.58

Continued on next page

Table S26: Error metrics of PEP extraction pipelines on the *Guardian Dataset* for different annotators and MAE difference between both annotators. MAE = Mean Absolute Error, ME = Mean Error.

			Annotator 1				Annotator 2				Ann. Diff.	
			MAE [ms]		ME [ms]		MAE [ms]		ME [ms]		MAE [ms]	
**Q-peak Algorithm**	**B-point Algorithm**	**Outlier Correction Algorithm**	**Mean**	**SD**	**Mean**	**SD**	**Mean**	**SD**	**Mean**	**SD**	**Mean**	**SD**
Mar04	Dro22	None	20.41	18.31	−17.17	21.38	23.18	22.58	−21.60	24.10	2.77	4.28
	For18	For18	20.41	26.92	4.35	33.50	20.29	26.50	0.58	33.37	−0.12	−0.42
Van13 (38 ms)	Ste85	LinInt	20.46	26.91	6.36	33.20	18.64	26.77	2.57	32.52	−1.82	−0.14
Van13 (42 ms)	Loz07LR	None	20.50	18.11	−14.51	23.19	23.58	21.34	−19.01	25.50	3.09	3.23
Van13 (34 ms)	For18	LinInt	20.53	25.75	3.68	32.72	19.94	25.55	−0.13	32.41	−0.59	−0.20
Mar04	Ste85	For18	20.53	29.57	11.17	34.22	19.07	28.78	7.29	33.75	−1.46	−0.79
Van13 (34 ms)	For18	For18	20.54	25.87	4.74	32.70	19.72	25.47	0.97	32.20	−0.82	−0.40
Van13 (36 ms)	Ste85	For18	20.61	28.25	9.81	33.57	18.47	27.83	6.02	32.85	−2.15	−0.42
Van13 (32 ms)	Ste85	LinInt	20.64	27.94	11.99	32.60	18.34	27.26	8.15	31.83	−2.29	−0.68
Van13 (34 ms)	Dro22	None	20.65	16.41	−17.14	20.04	23.30	20.46	−21.61	22.24	2.65	4.06
	Ste85	For18	20.73	28.63	11.69	33.36	18.43	28.01	7.89	32.59	−2.29	−0.62
Van13 (38 ms)	Ste85	For18	20.87	27.70	7.94	33.77	18.91	27.42	4.17	33.05	−1.96	−0.29
Van13 (36 ms)	For18	For18	21.06	25.43	2.86	32.89	20.37	25.22	−0.89	32.40	−0.69	−0.21
		LinInt	21.08	25.30	1.78	32.89	20.61	25.30	−2.02	32.57	−0.47	0.00
Van13 (42 ms)	Loz07LR	For18	21.12	19.28	−15.26	24.18	24.25	22.35	−19.76	26.41	3.13	3.08
Van13 (40 ms)	Ste85	LinInt	21.15	26.37	4.56	33.50	19.53	26.37	0.77	32.81	−1.63	0.00
For18	For18	LinInt	21.19	26.97	14.99	30.86	19.49	25.68	10.96	30.32	−1.71	−1.29
Van13 (42 ms)	Loz07LR	LinInt	21.21	19.33	−15.37	24.23	24.36	22.41	−19.87	26.47	3.15	3.08
Van13 (32 ms)	Ste85	For18	21.22	28.89	13.57	33.18	18.79	28.08	9.73	32.36	−2.43	−0.81
Mar04	Dro22	For18	21.42	18.94	−18.82	21.52	24.54	23.08	−23.30	24.33	3.12	4.14
Van13 (40 ms)	Ste85	For18	21.52	27.10	6.14	34.05	19.74	26.95	2.37	33.32	−1.77	−0.15
For18	For18	For18	21.52	27.30	16.02	30.85	19.62	25.85	12.05	30.13	−1.91	−1.45
Van13 (34 ms)	Dro22	For18	21.63	17.17	−18.73	20.30	24.64	21.08	−23.23	22.62	3.01	3.91
Mar04	Dro22	LinInt	21.70	19.20	−19.23	21.68	24.93	23.32	−23.71	24.55	3.23	4.11
Van13 (38 ms)	For18	For18	21.73	24.81	0.91	32.97	21.15	24.75	−2.86	32.44	−0.58	−0.05
		LinInt	21.80	24.72	−0.18	32.95	21.43	24.88	−3.99	32.59	−0.36	0.16
Van13 (34 ms)	Dro22	LinInt	21.87	17.30	−19.16	20.25	24.98	21.20	−23.68	22.64	3.11	3.90
Mar04	For18	None	21.89	29.68	9.11	35.74	21.27	28.91	5.44	35.48	−0.62	−0.77
Van13 (42 ms)	Ste85	LinInt	21.97	25.51	2.61	33.56	20.56	25.74	−1.15	32.92	−1.41	0.23
		For18	22.29	26.21	4.22	34.15	20.73	26.26	0.46	33.45	−1.56	0.05
Van13 (32 ms)	For18	None	22.29	29.37	11.27	35.11	20.79	28.56	7.61	34.50	−1.50	−0.82
Van13 (34 ms)	For18	None	22.40	28.74	9.36	35.21	21.07	28.08	5.72	34.64	−1.33	−0.65
Van13 (36 ms)	Dro22	None	22.47	16.22	−19.14	20.04	25.22	20.39	−23.61	22.24	2.75	4.17
Van13 (40 ms)	For18	For18	22.60	24.18	−1.02	33.08	22.16	24.33	−4.78	32.56	−0.44	0.15

Continued on next page

Table S26: Error metrics of PEP extraction pipelines on the *Guardian Dataset* for different annotators and MAE difference between both annotators. MAE = Mean Absolute Error, ME = Mean Error.

			Annotator 1				Annotator 2				Ann. Diff.	
			MAE [ms]		ME [ms]		MAE [ms]		ME [ms]		MAE [ms]	
			Mean	SD	Mean	SD	Mean	SD	Mean	SD	Mean	SD
**Q-peak Algorithm**	**B-point Algorithm**	**Outlier Correction Algorithm**										
		LinInt	22.70	24.14	−2.11	33.07	22.47	24.50	−5.92	32.72	−0.23	0.37
Van13 (42 ms)	Loz07QR	For18	22.77	17.60	8.54	27.48	23.54	18.78	3.98	29.86	0.78	1.18
		LinInt	22.78	17.63	8.31	27.59	23.57	18.84	3.75	29.94	0.79	1.20
Van13 (36 ms)	For18	None	22.83	28.09	7.50	35.41	21.61	27.60	3.88	34.84	−1.21	−0.49
For18	Mil22	LinInt	22.95	29.09	13.52	34.50	21.86	28.30	9.18	34.56	−1.09	−0.79
Van13 (42 ms)	Loz07QR	None	23.08	17.52	9.91	27.23	23.74	18.47	5.35	29.60	0.67	0.95
Van13 (38 ms)	For18	None	23.40	27.23	5.55	35.47	22.29	26.89	1.93	34.88	−1.11	−0.34
Van13 (40 ms)	Loz07QR	LinInt	23.43	17.85	10.31	27.59	23.97	18.83	5.75	29.94	0.55	0.98
		For18	23.43	17.82	10.54	27.48	23.96	18.79	5.98	29.86	0.53	0.97
Van13 (36 ms)	Dro22	For18	23.47	17.06	−20.73	20.30	26.57	21.04	−25.23	22.62	3.10	3.98
For18	Mil22	For18	23.49	29.50	14.82	34.67	22.14	28.60	10.51	34.61	−1.35	−0.90
Van13 (32 ms)	Mil22	LinInt	23.53	26.84	3.59	35.51	22.83	27.20	−0.73	35.51	−0.70	0.37
Van13 (36 ms)	Deb93SD	None	23.56	26.40	−13.58	32.67	28.28	28.71	−18.17	35.96	4.72	2.30
Van13 (42 ms)	For18	For18	23.58	23.48	−2.98	33.14	23.29	23.82	−6.74	32.63	−0.28	0.34
Mar04	Ste85	None	23.61	33.53	16.31	37.63	21.29	32.39	12.48	36.70	−2.33	−1.13
For18	For18	None	23.64	30.36	20.18	32.77	21.32	28.83	16.32	31.93	−2.32	−1.53
Van13 (34 ms)	Deb93SD	None	23.66	25.34	−11.58	32.67	28.26	27.50	−16.17	35.96	4.61	2.16
Van13 (38 ms)	Deb93SD	None	23.67	27.39	−15.58	32.67	28.44	29.86	−20.17	35.96	4.77	2.47
Van13 (32 ms)	Mil22	For18	23.71	26.98	4.89	35.58	22.75	27.20	0.59	35.46	−0.96	0.22
Van13 (42 ms)	For18	LinInt	23.71	23.47	−4.08	33.12	23.64	24.03	−7.89	32.77	−0.08	0.56
Van13 (36 ms)	Dro22	LinInt	23.75	17.38	−21.12	20.49	26.95	21.32	−25.64	22.87	3.20	3.94
Van13 (40 ms)	Loz07QR	None	23.80	17.80	11.91	27.23	24.22	18.54	7.35	29.60	0.42	0.74
Mar04	Mil22	LinInt	23.88	27.72	1.50	36.56	23.69	28.47	−2.78	36.94	−0.19	0.76
Van13 (32 ms)	Deb93SD	None	23.94	24.21	−9.58	32.67	28.37	26.26	−14.17	35.96	4.43	2.05
Van13 (36 ms)	Ste85	None	23.96	32.37	15.09	37.33	20.92	31.68	11.39	36.21	−3.04	−0.69
Van13 (40 ms)	Deb93SD	None	24.01	28.29	−17.58	32.67	28.80	30.92	−22.17	35.96	4.79	2.63
For18	Arb17TD	LinInt	24.03	29.08	−0.22	37.72	25.93	29.77	−4.35	39.25	1.91	0.70
Mar04	Mil22	For18	24.05	27.93	2.82	36.75	23.64	28.51	−1.44	37.01	−0.41	0.58
Van13 (34 ms)	Mil22	LinInt	24.11	26.24	1.63	35.60	23.60	26.79	−2.68	35.61	−0.51	0.55
For18	Arb17TD	For18	24.13	29.62	1.66	38.17	25.81	30.11	−2.46	39.59	1.68	0.50
Van13 (34 ms)	Ste85	None	24.15	32.94	16.92	37.17	20.97	32.09	13.20	36.00	−3.17	−0.85
Van13 (38 ms)	Loz07QR	LinInt	24.17	18.12	12.31	27.59	24.46	18.91	7.75	29.94	0.29	0.79
		For18	24.19	18.10	12.54	27.48	24.46	18.88	7.98	29.86	0.28	0.78

Continued on next page

Table S26: Error metrics of PEP extraction pipelines on the *Guardian Dataset* for different annotators and MAE difference between both annotators. MAE = Mean Absolute Error, ME = Mean Error.

			Annotator 1				Annotator 2				Ann. Diff.	
			MAE [ms]		ME [ms]		MAE [ms]		ME [ms]		MAE [ms]	
Q-peak Algorithm	B-point Algorithm	Outlier Correction Algorithm	Mean	SD	Mean	SD	Mean	SD	Mean	SD	Mean	SD
	Ste85	None	24.21	31.78	13.34	37.66	21.33	31.18	9.66	36.52	−2.88	−0.60
Van13 (34 ms)	Mil22	For18	24.23	26.34	2.94	35.67	23.46	26.74	−1.36	35.55	−0.77	0.41
Van13 (40 ms)	For18	None	24.28	26.52	3.75	35.76	23.30	26.34	0.13	35.17	−0.98	−0.18
Van13 (38 ms)	Dro22	None	24.32	16.12	−21.11	20.14	27.17	20.36	−25.58	22.32	2.85	4.24
Van13 (34 ms)	Deb93SD	For18	24.37	25.56	−14.05	32.40	28.84	27.76	−18.63	35.43	4.47	2.20
Van13 (32 ms)	Deb93SD	For18	24.44	24.45	−12.05	32.40	28.73	26.57	−16.63	35.43	4.29	2.12
Van13 (36 ms)	Deb93SD	For18	24.48	26.60	−16.05	32.40	29.06	28.91	−20.63	35.43	4.58	2.31
Van13 (38 ms)	Loz07QR	None	24.61	18.15	13.91	27.23	24.77	18.71	9.35	29.60	0.17	0.56
Van13 (42 ms)	Deb93SD	None	24.62	29.07	−19.58	32.67	29.38	31.85	−24.17	35.96	4.76	2.78
Van13 (32 ms)	Ste85	None	24.66	33.17	18.68	36.87	21.37	32.18	14.92	35.63	−3.29	−0.99
Van13 (38 ms)	Deb93SD	For18	24.81	27.57	−18.05	32.40	29.44	30.01	−22.63	35.43	4.63	2.44
Mar04	Deb93SD	None	24.86	25.79	−11.65	33.88	29.96	27.65	−16.21	37.41	5.10	1.86
Van13 (36 ms)	Mil22	LinInt	24.88	25.46	−0.37	35.60	24.55	26.20	−4.68	35.61	−0.33	0.74
Van13 (40 ms)	Ste85	None	24.91	31.15	11.73	38.12	22.19	30.61	8.02	36.94	−2.72	−0.54
Van13 (36 ms)	Mil22	For18	24.94	25.51	0.94	35.67	24.36	26.11	−3.36	35.55	−0.58	0.59
	Loz07QR	LinInt	25.00	18.47	14.31	27.59	25.05	19.07	9.75	29.94	0.06	0.60
		For18	25.02	18.45	14.54	27.48	25.06	19.04	9.98	29.86	0.04	0.59
Van13 (42 ms)	For18	None	25.16	25.54	1.78	35.81	24.33	25.53	−1.82	35.22	−0.83	−0.01
Van13 (32 ms)	Deb93SD	LinInt	25.25	24.81	−13.32	32.79	29.53	26.86	−17.92	35.66	4.28	2.04
Van13 (34 ms)	Deb93SD	LinInt	25.25	25.94	−15.32	32.79	29.70	28.04	−19.92	35.66	4.45	2.11
Van13 (38 ms)	Dro22	For18	25.33	17.01	−22.70	20.39	28.52	21.03	−27.21	22.70	3.19	4.01
Van13 (40 ms)	Deb93SD	For18	25.35	28.45	−20.05	32.40	30.00	31.01	−24.63	35.43	4.65	2.56
Van13 (36 ms)	Deb93SD	LinInt	25.43	27.00	−17.32	32.79	30.00	29.20	−21.92	35.66	4.56	2.20
	Loz07QR	None	25.50	18.56	15.91	27.23	25.42	18.94	11.35	29.60	−0.08	0.39
For18	Ste85	LinInt	25.58	26.85	21.09	30.50	22.85	25.20	17.03	29.46	−2.72	−1.65
Van13 (32 ms)	Mil22	None	25.58	29.52	9.10	37.99	23.95	29.38	4.90	37.59	−1.63	−0.14
Van13 (42 ms)	Ste85	None	25.62	30.10	9.87	38.27	23.10	29.69	6.18	37.11	−2.51	−0.40
Mar04	Deb93SD	For18	25.62	26.15	−14.16	33.76	30.58	28.02	−18.71	37.02	4.96	1.87
Van13 (38 ms)	Dro22	LinInt	25.63	17.33	−23.10	20.58	28.90	21.31	−27.62	22.95	3.28	3.98
Mar04	Mil22	None	25.64	30.04	7.02	38.87	24.51	30.25	2.86	38.82	−1.14	0.20
Van13 (38 ms)	Mil22	For18	25.82	24.63	−1.06	35.67	25.42	25.42	−5.36	35.55	−0.39	0.79

Continued on next page

Table S26: Error metrics of PEP extraction pipelines on the *Guardian Dataset* for different annotators and MAE difference between both annotators. MAE = Mean Absolute Error, ME = Mean Error.

			Annotator 1				Annotator 2				Ann. Diff.	
			MAE [ms]		ME [ms]		MAE [ms]		ME [ms]		MAE [ms]	
**Q-peak Algorithm**	**B-point Algorithm**	**Outlier Correction Algorithm**	**Mean**	**SD**	**Mean**	**SD**	**Mean**	**SD**	**Mean**	**SD**	**Mean**	**SD**
		LinInt	25.82	24.62	−2.37	35.60	25.67	25.56	−6.68	35.61	−0.16	0.95
	Deb93SD	LinInt	25.84	28.09	−19.28	32.94	30.47	30.40	−23.88	35.81	4.63	2.31
For18	Mil22	None	25.90	32.49	18.93	36.99	24.06	31.29	14.71	36.63	−1.84	−1.20
Van13 (34 ms)	Loz07QR	LinInt	25.92	18.84	16.31	27.59	25.74	19.28	11.75	29.94	−0.18	0.44
		For18	25.96	18.84	16.54	27.48	25.76	19.26	11.98	29.86	−0.20	0.42
	Mil22	None	25.99	28.72	7.15	38.06	24.53	28.75	2.95	37.67	−1.46	0.03
Mar04	Loz07QR	LinInt	26.16	18.24	16.22	27.45	26.25	19.05	11.68	30.26	0.10	0.81
Van13 (40 ms)	Dro22	None	26.16	15.98	−23.11	20.14	29.11	20.29	−27.58	22.32	2.95	4.31
Van13 (42 ms)	Deb93SD	For18	26.17	29.31	−22.01	32.55	30.81	31.99	−26.59	35.58	4.64	2.68
Mar04	Loz07QR	For18	26.21	18.23	16.46	27.36	26.29	19.04	11.92	30.19	0.08	0.80
For18	Ste85	For18	26.29	27.54	22.46	30.74	23.44	25.80	18.40	29.61	−2.85	−1.74
Mar04	Deb93SD	LinInt	26.37	26.49	−15.45	34.04	31.30	28.30	−20.02	37.15	4.93	1.81
Van13 (40 ms)	Deb93SD	LinInt	26.43	28.97	−21.28	32.94	31.09	31.40	−25.88	35.81	4.66	2.42
Van13 (34 ms)	Loz07QR	None	26.49	18.99	17.91	27.23	26.17	19.23	13.35	29.60	−0.32	0.24
For18	Deb93SD	For18	26.51	18.43	−1.83	32.23	29.55	20.46	−6.42	35.37	3.05	2.04
Van13 (36 ms)	Mil22	None	26.59	27.71	5.15	38.06	25.31	27.92	0.95	37.67	−1.29	0.21
Mar04	Loz07QR	None	26.69	18.39	17.84	27.06	26.65	19.01	13.31	29.90	−0.05	0.61
Van13 (40 ms)	Mil22	For18	26.76	23.78	−3.06	35.67	26.59	24.71	−7.36	35.55	−0.17	0.94
		LinInt	26.83	23.80	−4.37	35.60	26.88	24.91	−8.68	35.61	0.06	1.10
For18	Deb93SD	LinInt	26.85	18.69	−3.11	32.57	29.90	20.75	−7.70	35.57	3.04	2.06
Van13 (32 ms)	Loz07QR	LinInt	26.88	19.05	18.26	27.42	26.46	19.34	13.69	29.78	−0.42	0.28
		For18	26.93	19.05	18.49	27.32	26.50	19.32	13.92	29.69	−0.43	0.27
For18	Deb93SD	None	26.94	18.05	0.63	32.42	30.05	19.94	−3.95	35.85	3.12	1.89
Van13 (40 ms)	Dro22	For18	27.19	16.93	−24.70	20.39	30.47	20.98	−29.21	22.70	3.28	4.05
Van13 (42 ms)	Deb93SD	LinInt	27.26	29.73	−23.28	32.94	31.92	32.26	−27.88	35.81	4.66	2.53
Van13 (38 ms)	Mil22	None	27.41	26.70	3.19	38.13	26.29	27.08	−1.01	37.73	−1.11	0.38
Van13 (40 ms)	Dro22	LinInt	27.50	17.25	−25.10	20.58	30.85	21.26	−29.62	22.95	3.36	4.01
Van13 (32 ms)	Loz07QR	None	27.50	19.25	19.86	27.07	26.94	19.36	15.30	29.44	−0.56	0.11
Van13 (42 ms)	Mil22	For18	27.73	22.99	−5.06	35.67	27.80	24.05	−9.36	35.55	0.07	1.06
		LinInt	27.87	23.05	−6.37	35.60	28.15	24.28	−10.68	35.61	0.28	1.23
	Dro22	None	28.00	15.88	−25.11	20.14	31.05	20.23	−29.58	22.32	3.05	4.35
Van13 (40 ms)	Mil22	None	28.24	25.64	1.19	38.13	27.35	26.16	−3.01	37.73	−0.89	0.52
For18	Arb17TD	None	28.50	36.35	8.58	45.39	29.99	36.44	4.50	46.99	1.50	0.09
	She90	LinInt	28.76	26.02	9.06	37.71	28.68	25.95	4.76	38.38	−0.08	−0.08
	Arb17IC	LinInt	28.89	26.29	9.92	37.79	28.78	26.09	5.62	38.44	−0.11	−0.21
	She90	For18	29.00	26.15	10.14	37.71	28.83	25.97	5.85	38.36	−0.16	−0.18
Van13 (42 ms)	Dro22	For18	29.05	16.88	−26.70	20.39	32.41	20.94	−31.21	22.70	3.36	4.07
	Mil22	None	29.11	24.64	−0.81	38.13	28.46	25.27	−5.01	37.73	−0.65	0.63
For18	Arb17IC	For18	29.24	26.44	10.83	37.90	28.99	26.22	6.54	38.53	−0.25	−0.22

Continued on next page

Table S26: Error metrics of PEP extraction pipelines on the *Guardian Dataset* for different annotators and MAE difference between both annotators. MAE = Mean Absolute Error, ME = Mean Error.

			Annotator 1				Annotator 2				Ann. Diff.	
			MAE [ms]		ME [ms]		MAE [ms]		ME [ms]		MAE [ms]	
**Q-peak Algorithm**	**B-point Algorithm**	**Outlier Correction Algorithm**	**Mean**	**SD**	**Mean**	**SD**	**Mean**	**SD**	**Mean**	**SD**	**Mean**	**SD**
Van13 (42 ms)	Dro22	LinInt	29.37	17.19	−27.10	20.58	32.80	21.22	−31.62	22.95	3.44	4.03
For18	Ste85	None	29.95	31.74	27.14	34.17	26.38	30.02	23.10	32.61	−3.57	−1.71
Van13 (32 ms)	Arb17TD	For18	29.97	28.90	−6.97	41.05	32.05	29.95	−10.94	42.48	2.07	1.05
		LinInt	30.27	28.59	−8.94	40.67	32.54	29.81	−12.93	42.20	2.27	1.22
Mar04	Arb17TD	For18	30.80	29.06	−9.00	41.38	33.12	30.59	−12.97	43.18	2.32	1.53
Van13 (32 ms)	She90	For18	30.95	21.73	−0.04	37.81	31.77	21.90	−4.34	38.35	0.83	0.17
	Arb17IC	LinInt	31.00	21.91	−0.23	37.97	31.87	22.14	−4.51	38.54	0.87	0.22
	She90	LinInt	31.01	21.67	−1.12	37.82	31.92	22.02	−5.41	38.40	0.91	0.35
For18	Arb17IC	None	31.02	27.77	13.91	39.24	30.38	27.38	9.71	39.73	−0.64	−0.39
Van13 (32 ms)	Arb17IC	For18	31.15	21.89	0.69	38.07	31.91	22.07	−3.59	38.63	0.75	0.17
Mar04	Arb17TD	LinInt	31.18	28.71	−10.89	40.96	33.71	30.45	−14.85	42.93	2.53	1.74
	She90	For18	31.24	21.86	−2.11	38.07	32.38	22.54	−6.38	38.94	1.14	0.68
	Arb17IC	LinInt	31.32	22.10	−2.27	38.27	32.53	22.80	−6.52	39.19	1.21	0.70
Van13 (34 ms)	Arb17TD	For18	31.34	28.52	−8.68	41.48	33.46	29.66	−12.64	42.89	2.12	1.14
Mar04	She90	LinInt	31.35	21.83	−3.21	38.07	32.55	22.65	−7.49	38.94	1.20	0.82
	Arb17IC	For18	31.37	22.10	−1.34	38.35	32.49	22.72	−5.58	39.25	1.12	0.63
Van13 (34 ms)	She90	For18	31.49	21.03	−2.04	37.81	32.53	21.27	−6.34	38.35	1.04	0.23
	Arb17IC	LinInt	31.58	21.19	−2.23	37.97	32.66	21.48	−6.51	38.54	1.08	0.29
For18	She90	None	31.59	27.99	14.27	39.72	30.94	27.73	10.06	40.32	−0.65	−0.26
Van13 (34 ms)	She90	LinInt	31.61	21.00	−3.12	37.82	32.73	21.41	−7.41	38.40	1.12	0.41
	Arb17IC	For18	31.69	21.14	−1.31	38.07	32.66	21.36	−5.59	38.63	0.97	0.23
	Arb17TD	LinInt	31.70	28.25	−10.66	41.10	34.01	29.55	−14.64	42.61	2.32	1.29
Van13 (36 ms)	She90	For18	32.07	20.43	−4.04	37.81	33.32	20.72	−8.34	38.35	1.25	0.29
	Arb17IC	LinInt	32.19	20.56	−4.23	37.97	33.47	20.91	−8.51	38.54	1.28	0.35
	She90	LinInt	32.25	20.41	−5.12	37.82	33.57	20.88	−9.41	38.40	1.32	0.47
	Arb17IC	For18	32.26	20.49	−3.31	38.07	33.45	20.76	−7.59	38.63	1.19	0.27
Van13 (32 ms)	Arb17IC	None	32.60	22.66	3.80	39.52	33.02	22.47	−0.39	39.94	0.42	−0.19
Mar04	Arb17IC	None	32.61	22.61	1.80	39.64	33.37	22.90	−2.35	40.40	0.76	0.29
Van13 (38 ms)	She90	For18	32.69	19.94	−6.04	37.81	34.13	20.30	−10.34	38.35	1.45	0.35
Van13 (36 ms)	Arb17TD	For18	32.77	28.18	−10.37	41.96	34.96	29.43	−14.29	43.40	2.19	1.25
For18	Loz07QR	LinInt	32.84	20.94	28.45	26.59	31.39	20.89	23.89	29.17	−1.45	−0.05
Van13 (38 ms)	Arb17IC	LinInt	32.86	20.08	−6.20	38.01	34.32	20.44	−10.51	38.54	1.46	0.36
		For18	32.88	20.00	−5.29	38.12	34.26	20.26	−9.59	38.63	1.38	0.26
	She90	LinInt	32.93	19.92	−7.12	37.82	34.44	20.46	−11.41	38.40	1.51	0.54
For18	Loz07QR	For18	32.95	20.95	28.69	26.50	31.48	20.91	24.12	29.09	−1.47	−0.04
Van13 (32 ms)	She90	None	33.00	22.67	4.05	39.83	33.43	22.54	−0.16	40.32	0.43	−0.13
Van13 (34 ms)	Arb17IC	None	33.06	21.81	1.82	39.56	33.71	21.63	−2.36	39.98	0.65	−0.18
Mar04	She90	None	33.16	22.64	2.08	40.10	33.88	23.00	−2.10	40.89	0.72	0.36
Van13 (36 ms)	Arb17TD	LinInt	33.18	27.92	−12.37	41.56	35.56	29.32	−16.31	43.11	2.38	1.41
Van13 (40 ms)	She90	For18	33.34	19.57	−8.04	37.81	34.97	19.99	−12.34	38.35	1.63	0.42

Continued on next page

Table S26: Error metrics of PEP extraction pipelines on the *Guardian Dataset* for different annotators and MAE difference between both annotators. MAE = Mean Absolute Error, ME = Mean Error.

Q-peak Algorithm	B-point Algorithm	Outlier Correction Algorithm	Annotator 1 MAE [ms] Mean	Annotator 1 MAE [ms] SD	Annotator 1 ME [ms] Mean	Annotator 1 ME [ms] SD	Annotator 2 MAE [ms] Mean	Annotator 2 MAE [ms] SD	Annotator 2 ME [ms] Mean	Annotator 2 ME [ms] SD	Ann. Diff. MAE [ms] Mean	Ann. Diff. MAE [ms] SD
Van13 (34 ms)	She90	None	33.41	21.78	2.05	39.83	34.07	21.66	−2.16	40.32	0.66	−0.12
Van13 (40 ms)	Arb17IC	For18	33.50	19.59	−7.29	38.12	35.08	19.89	−11.59	38.63	1.58	0.30
Van13 (36 ms)	Arb17IC	None	33.52	21.01	−0.18	39.56	34.40	20.84	−4.36	39.98	0.88	−0.18
Van13 (40 ms)	Arb17IC	LinInt	33.54	19.68	−8.20	38.01	35.19	20.09	−12.51	38.54	1.65	0.41
	She90	LinInt	33.63	19.56	−9.12	37.82	35.33	20.15	−13.41	38.40	1.70	0.59
For18	Loz07QR	None	33.73	21.31	30.05	26.25	32.14	21.18	25.50	28.84	−1.59	−0.13
Van13 (36 ms)	She90	None	33.86	20.98	0.05	39.83	34.74	20.87	−4.16	40.32	0.89	−0.11
Van13 (42 ms)	She90	For18	34.01	19.34	−10.04	37.81	35.84	19.85	−14.31	38.39	1.83	0.51
Van13 (38 ms)	Arb17IC	None	34.02	20.38	−2.15	39.60	35.11	20.16	−6.36	39.98	1.09	−0.22
Van13 (42 ms)	Arb17IC	For18	34.14	19.32	−9.29	38.12	35.92	19.66	−13.59	38.63	1.78	0.34
Van13 (38 ms)	Arb17TD	For18	34.23	27.79	−12.05	42.41	36.48	29.15	−15.95	43.89	2.26	1.35
Van13 (42 ms)	Arb17IC	LinInt	34.23	19.41	−10.20	38.01	36.07	19.87	−14.51	38.54	1.83	0.46
Van13 (38 ms)	She90	None	34.33	20.30	−1.95	39.83	35.43	20.21	−6.16	40.32	1.10	−0.09
Van13 (42 ms)	She90	LinInt	34.35	19.33	−11.12	37.82	36.24	19.96	−15.41	38.40	1.89	0.63
Van13 (40 ms)	Arb17IC	None	34.52	19.84	−4.15	39.60	35.82	19.62	−8.36	39.98	1.30	−0.21
Van13 (42 ms)	Arb17SD	LinInt	34.55	19.82	25.67	30.45	34.40	18.64	21.20	32.88	−0.15	−1.18
Van13 (32 ms)	Arb17TD	None	34.70	35.07	1.17	49.32	36.47	35.50	−2.76	50.82	1.78	0.43
Van13 (38 ms)	Arb17TD	LinInt	34.71	27.57	−14.05	42.04	37.15	29.06	−17.96	43.62	2.44	1.50
Van13 (40 ms)	She90	None	34.81	19.75	−3.95	39.83	36.12	19.68	−8.16	40.32	1.31	−0.08
Van13 (42 ms)	Arb17SD	For18	34.83	19.72	26.12	30.33	34.65	18.48	21.65	32.76	−0.18	−1.24
	Arb17IC	None	35.04	19.45	−6.15	39.60	36.55	19.24	−10.36	39.98	1.51	−0.21
	She90	None	35.31	19.36	−5.95	39.83	36.83	19.30	−10.16	40.32	1.51	−0.07
Van13 (40 ms)	Arb17TD	For18	35.73	27.47	−13.70	42.94	38.04	28.92	−17.57	44.44	2.32	1.44
	Arb17SD	LinInt	35.81	20.16	27.65	30.41	35.41	19.03	23.18	32.84	−0.40	−1.14
Mar04	Arb17TD	None	35.81	35.23	−0.01	50.24	37.73	35.93	−3.96	51.95	1.91	0.70
Van13 (42 ms)	Arb17SD	None	36.02	19.73	27.57	30.43	35.76	18.26	23.10	32.84	−0.26	−1.47
Van13 (34 ms)	Arb17TD	None	36.09	34.52	−0.28	49.95	37.93	35.02	−4.19	51.46	1.83	0.50
Van13 (40 ms)	Arb17SD	For18	36.12	20.05	28.10	30.29	35.68	18.87	23.63	32.72	−0.44	−1.19
	Arb17TD	LinInt	36.28	27.27	−15.69	42.59	38.77	28.86	−19.58	44.19	2.49	1.59
Van13 (38 ms)	Arb17SD	LinInt	37.13	20.63	29.65	30.41	36.46	19.58	25.18	32.84	−0.67	−1.05
Van13 (42 ms)	Arb17TD	For18	37.26	27.24	−15.31	43.54	39.64	28.76	−19.17	45.07	2.38	1.52

Continued on next page

Table S26: Error metrics of PEP extraction pipelines on the *Guardian Dataset* for different annotators and MAE difference between both annotators. MAE = Mean Absolute Error, ME = Mean Error.

			Annotator 1				Annotator 2				Ann. Diff.	
			MAE [ms]		ME [ms]		MAE [ms]		ME [ms]		MAE [ms]	
Q-peak Algorithm	B-point Algorithm	Outlier Correction Algorithm	Mean	SD	Mean	SD	Mean	SD	Mean	SD	Mean	SD
Van13 (40 ms)	Arb17SD	None	37.36	20.03	29.55	30.39	36.83	18.65	25.08	32.80	−0.52	−1.38
Van13 (38 ms)	Arb17SD	For18	37.45	20.50	30.10	30.29	36.75	19.41	25.63	32.72	−0.70	−1.09
Van13 (36 ms)	Arb17TD	None	37.62	34.06	−1.62	50.72	39.52	34.66	−5.48	52.28	1.90	0.60
Van13 (42 ms)	Arb17TD	LinInt	37.86	27.04	−17.33	43.18	40.41	28.69	−21.20	44.80	2.55	1.65
Van13 (36 ms)	Arb17SD	LinInt	38.46	21.15	31.65	30.41	37.53	20.22	27.18	32.84	−0.93	−0.94
Van13 (38 ms)	Arb17SD	None	38.73	20.46	31.55	30.39	37.94	19.22	27.08	32.80	−0.79	−1.24
Van13 (36 ms)	Arb17SD	For18	38.81	21.01	32.10	30.29	37.84	20.05	27.63	32.72	−0.96	−0.97
Van13 (38 ms)	Arb17TD	None	39.09	33.47	−3.03	51.38	41.05	34.15	−6.85	52.96	1.96	0.67
Van13 (34 ms)	Arb17SD	LinInt	39.81	21.72	33.65	30.41	38.63	20.92	29.18	32.84	−1.18	−0.80
Van13 (36 ms)	Arb17SD	None	40.12	20.96	33.55	30.39	39.06	19.88	29.08	32.80	−1.06	−1.07
Mar04	Arb17SD	LinInt	40.15	22.49	33.68	31.37	39.09	22.14	29.24	34.11	−1.06	−0.35
Van13 (34 ms)	Arb17SD	For18	40.18	21.57	34.10	30.29	38.95	20.76	29.63	32.72	−1.23	−0.81
Mar04	Arb17SD	For18	40.53	22.34	34.14	31.26	39.42	21.99	29.69	34.00	−1.11	−0.36
Van13 (40 ms)	Arb17TD	None	40.61	32.94	−4.39	52.11	42.61	33.66	−8.19	53.68	2.00	0.72
Van13 (32 ms)	Arb17SD	LinInt	41.20	22.33	35.65	30.41	39.75	21.69	31.18	32.84	−1.45	−0.63
Van13 (34 ms)	Arb17SD	None	41.53	21.51	35.55	30.39	40.20	20.64	31.08	32.80	−1.33	−0.88
Van13 (32 ms)	Arb17SD	For18	41.58	22.17	36.10	30.29	40.09	21.54	31.63	32.72	−1.49	−0.63
Mar04	Arb17SD	None	41.87	22.34	35.61	31.37	40.61	21.99	31.16	34.09	−1.26	−0.34
Van13 (42 ms)	Arb17TD	None	42.13	32.42	−5.70	52.85	44.19	33.20	−9.48	54.45	2.06	0.78
For18	Pal21	LinInt	42.88	35.59	2.36	55.68	44.34	35.58	−0.60	56.86	1.46	−0.01
Van13 (32 ms)	Arb17SD	None	42.95	22.12	37.55	30.39	41.34	21.46	33.08	32.80	−1.60	−0.66
For18	Pal21	For18	44.11	36.99	4.29	57.41	45.38	36.55	1.32	58.26	1.27	−0.43
		None	45.61	39.00	7.29	59.57	46.34	38.15	4.57	59.85	0.74	−0.85
	Arb17SD	LinInt	48.71	25.47	45.86	30.30	46.07	25.92	41.40	32.87	−2.65	0.45
		For18	49.14	25.32	46.31	30.18	46.44	25.82	41.84	32.75	−2.70	0.50
Van13 (32 ms)	Pal21	LinInt	49.56	37.50	−1.31	62.14	50.93	37.33	−4.14	63.02	1.37	−0.17
Mar04	Pal21	LinInt	50.18	37.41	−2.98	62.52	51.61	37.08	−6.07	63.27	1.44	−0.33
Van13 (32 ms)	Pal21	For18	50.45	38.49	0.58	63.46	51.63	38.05	−2.24	64.10	1.18	−0.43
For18	Arb17SD	None	50.56	25.36	47.77	30.28	47.74	25.96	43.30	32.83	−2.82	0.60
Van13 (34 ms)	Pal21	LinInt	50.91	37.54	−2.00	63.23	52.23	37.34	−4.84	64.02	1.31	−0.20
Mar04	Pal21	For18	51.09	38.16	−0.97	63.76	52.31	37.54	−4.06	64.26	1.22	−0.62

Continued on next page

Table S26: Error metrics of PEP extraction pipelines on the *Guardian Dataset* for different annotators and MAE difference between both annotators. MAE = Mean Absolute Error, ME = Mean Error.

			Annotator 1				Annotator 2				Ann. Diff.	
			MAE [ms]		ME [ms]		MAE [ms]		ME [ms]		MAE [ms]	
Q-peak Algorithm	B-point Algorithm	Outlier Correction Algorithm	Mean	SD	Mean	SD	Mean	SD	Mean	SD	Mean	SD
Van13 (34 ms)	Pal21	For18	51.76	38.42	−0.11	64.46	52.88	37.98	−2.93	65.04	1.12	−0.44
Van13 (36 ms)	Pal21	LinInt	52.30	37.41	−2.80	64.24	53.56	37.19	−5.67	64.96	1.26	−0.22
Van13 (32 ms)	Pal21	None	52.35	40.33	4.91	65.91	52.96	39.59	2.38	66.08	0.61	−0.74
Mar04	Pal21	None	53.01	39.99	3.79	66.29	53.53	38.95	0.94	66.20	0.53	−1.04
Van13 (36 ms)	Pal21	For18	53.11	38.21	−0.90	65.42	54.15	37.74	−3.77	65.90	1.05	−0.47
Van13 (34 ms)	Pal21	None	53.79	40.25	4.57	67.03	54.33	39.53	2.04	67.17	0.55	−0.72
Van13 (38 ms)	Pal21	LinInt	53.84	37.20	−3.39	65.35	55.04	36.97	−6.26	66.01	1.20	−0.23
		For18	54.63	37.96	−1.46	66.51	55.61	37.49	−4.33	66.94	0.99	−0.47
Van13 (36 ms)	Pal21	None	55.20	39.94	4.00	68.02	55.68	39.22	1.45	68.10	0.48	−0.73
Van13 (40 ms)	Pal21	LinInt	55.21	36.77	−4.28	66.20	56.35	36.43	−7.20	66.72	1.13	−0.34
		For18	55.95	37.44	−2.38	67.28	56.87	36.89	−5.31	67.58	0.92	−0.55
Van13 (42 ms)	Pal21	LinInt	56.65	36.37	−5.10	67.13	57.78	36.04	−8.03	67.63	1.13	−0.33
Van13 (38 ms)	Pal21	None	56.71	39.49	3.57	69.02	57.10	38.76	0.99	69.01	0.39	−0.73
Van13 (42 ms)	Pal21	For18	57.37	36.99	−3.16	68.20	58.27	36.43	−6.10	68.46	0.90	−0.56
Van13 (40 ms)	Pal21	None	58.13	38.91	2.93	69.89	58.42	38.08	0.27	69.74	0.29	−0.83
Van13 (42 ms)	Pal21	None	59.51	38.25	2.20	70.72	59.79	37.44	−0.47	70.55	0.27	−0.81

4.5.2 Effect of Annotation Agreement on PEP Pipeline Detection Error

Table S27: Effect of annotation agreement on the absolute error (AE) of selected PEP pipeline [For18, Dro22, None] on the *Guardian Dataset*, using Q-peak annotations for agreement computation. Annotation agreements: *high*: [0 ms, 4 ms], *medium*: [5 ms, 10 ms], *low*: ≥ 11 ms.

	**Annotator 1**		**Annotator 2**	
	**Mean**	**SD**	**Mean**	**SD**
Agreement Bins				
high	11.43	15.76	13.09	18.86
medium	11.05	12.10	16.32	21.80
low	18.73	21.40	26.16	24.58

Table S28: Effect of annotation agreement on the absolute error (AE) of selected PEP pipeline [For18, Dro22, None] on the *Guardian Dataset*, using B-point annotations for agreement computation. Annotation agreements: *high*: [0 ms, 4 ms], *medium*: [5 ms, 10 ms], *low*: ≥ 11 ms.

	**Annotator 1**		**Annotator 2**	
	**Mean**	**SD**	**Mean**	**SD**
Agreement Bins				
high	9.79	14.24	9.50	14.33
medium	15.34	15.81	11.85	15.13
low	17.56	21.17	33.92	26.48

References

[1] M. Ollenschläger, A. Küderle, W. Mehringer, A.-K. Seifer, J. Winkler, H. Gaßner, F. Kluge, and B. M. Eskofier, "MaD GUI: An Open-Source Python Package for Annotation and Analysis of Time-Series Data," *Sensors*, vol. 22, p. 5849, Aug. 2022.

[2] J. Martinez, R. Almeida, S. Olmos, A. Rocha, and P. Laguna, "A Wavelet-Based ECG Delineator: Evaluation on Standard Databases," *IEEE Transactions on Biomedical Engineering*, vol. 51, pp. 570–581, Apr. 2004.

[3] L. Drost, J. B. Finke, J. Port, and H. Schächinger, "Comparison of TWA and PEP as indices of A2- and ß-adrenergic activation," *Psychopharmacology*, Apr. 2022.

[4] T. T. Debski, Y. Zhang, J. Jennings, and T. W. Kamarck, "Stability of cardiac impedance measures: Aortic opening (B-point) detection and scoring," *Biological Psychology*, vol. 36, pp. 63–74, Aug. 1993.

[5] M. Forouzanfar, F. C. Baker, M. De Zambotti, C. McCall, L. Giovangrandi, and G. T. A. Kovacs, "Toward a better noninvasive assessment of preejection period: A novel automatic algorithm for B-point detection and correction on thoracic impedance cardiogram," *Psychophysiology*, vol. 55, p. e13072, Aug. 2018.
